# South Texas Is Home to High Diversity of Cytochrome Oxidase I Haplotypes Compared to Other Invasive Populations of *Aedes aegypti*

**DOI:** 10.3390/insects17090879

**Published:** 2026-08-22

**Authors:** Rachel C. Malampy, Mariana Aguilar, Erin L. Schuenzel, Christopher J. Vitek

**Affiliations:** 1 School of Integrative Biological and Chemical Science, University of Texas Rio Grande Valley, 1201 W. University Drive, Edinburg, TX 78539, USA; r.malampy@northeastern.edu (R.C.M.); mariana.aguilar01@utrgv.edu (M.A.); erin.schuenzel@utrgv.edu (E.L.S.); 2 Center for Vector-Borne, Zoonotic and Emerging Diseases, University of Texas Rio Grande Valley, 1201 W. University Drive, Edinburg, TX 78539, USA

**Keywords:** COI, genetic diversity, *Aedes aegypti*

## Abstract

Understanding population dynamics and genetics can be critical to understanding distribution, spread, and vectorial capacity of disease vectors. One well-studied disease vector, *Aedes aegypti*, has been the focus of research due to the wide range of diseases it vectors and the high transmission capabilities. We examined the genetic diversity of *A. aegypti* utilizing a large number of individual samples from a small geographic range, as opposed to previous studies that focus on a large range and small collection. Using the COI locus, we examined 107 mosquitoes from South Texas, with multiple haplotypes identified. We observed that the samples from South Texas have a unique distribution of haplotypes relative to several invasive global locations from North America, Central America, South America, and Asia. Population measurements suggest a recent population expansion in South Texas, with multiple rare alleles identified. Our intensive sampling strategy using a small geographic scale revealed high levels of diversity not found in non-East African countries and unique haplotypes. We hypothesize that expanding sampling size when studying genetic diversity could help inform movement of mosquitoes. In addition, increased haplotype diversity may be correlated with increased insecticide resistance and differing vector competence.

## Simple Summary

The mosquito *Aedes aegypti* is capable of spreading a wide range of diseases that adversely affect human health. As the mosquito populations expand to new locations and increase in population, the risk to human health increases. To understand how the mosquito is spreading and the genetic composition of the current mosquito population in South Texas, partial DNA sequencing of the cytochrome oxidase I gene was completed for over 100 individuals. The large number of individuals sampled from a small geographic area resulted in new, unique sequences. These new sequences indicate the potential for new responses to pesticides and disease, and the need for increased sampling in other locations due to missing these rarer sequences.

## 1. Introduction

There are approximately 3500 species of mosquitoes around the world that contribute to the spread of many disease-causing viruses and parasites [1,2]. Mosquitoes within the genus *Aedes* belong to the Family Culicidae in the Order Diptera. *Aedes* spp. mosquitoes are known to transmit a wide variety of human pathogens, including dengue virus, LaCrosse encephalitis virus, yellow fever virus, chikungunya virus, and Zika virus [2,3,4,5]. *Aedes* spp. mosquitoes are some of the most widespread species found in Asia, Africa, and the Americas [6,7,8,9]. While some of the species within this genus are more sylvan, a large number have adapted to human habitats, can thrive in urban environments, and have shown a strong ability to invade new habitats [5,6,8,9].

One of the primary vectors of human disease, *Aedes aegypti*, is an invasive mosquito in the US, having originated in Africa and spread to the Americas approximately 500 years ago [7]. *A. aegypti* is adapted to the human environment, is anthropophilic, and is the principal vector for diseases such as dengue, chikungunya, and Zika virus [10,11,12,13,14]. *A. aegypti* has a high vectorial capacity due to a close association with humans and will often bite multiple humans to engorge on blood prior to oviposition, a behavior referred to as sip feeding [12,13,15]. Humans and *A. aegypti* have an intertwined relationship, so it is important to understand dispersal patterns and population dynamics of these vectors because they have a high impact on human health risk [10]. Given the worldwide importance of the genus *Aedes* in human health, understanding the population dynamics of the mosquito can provide insight into viral transmission rates, insecticide resistance, and control strategies. Mitochondrial DNA, and specifically cytochrome oxidase I (COI), has been used to trace evolutionary patterns and population differences in multiple insects [16,17]. COI has been used in multiple studies to analyze different species and different populations of *Aedes* from around the world.

*Aedes aegypti* is a well-studied insect [18,19,20,21,22,23,24,25] with multiple populations sampled genetically around the world. Previous studies [18,19,20,21,22] have focused on sampling a few individuals from a large geographic area versus a more intensive sampling strategy in a smaller range to study genetic differences across a country to large portions of a continent. In this context, we set out to model the genetic diversity of *Aedes aegypti* at varying levels of geographic scale. South Texas may serve as an entry point for *A. aegypti* mosquitoes due to the frequent cross-border travel as well as trade ports located in Brownsville. There were over 40 million border crossings in South Texas in 2025 [26]. We compared the haplotype diversity of *A. aegypti* populations from a small geographic region of South Texas to a haplotype dataset collected across several states in the United States, and across multiple countries in Central and South America, Africa, and Asia.

## 2. Materials and Methods

### 2.1. Sample Collection Methods

**Figure 1:**
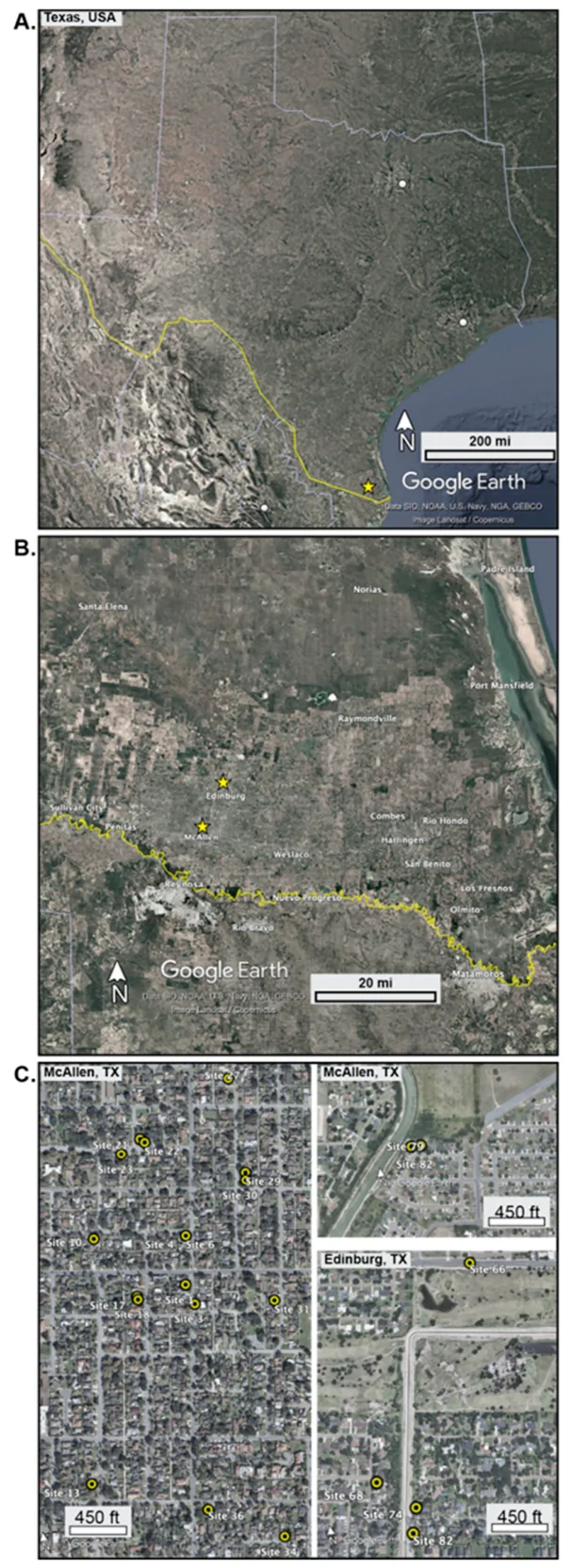
Geographic locations of collection sites in South Texas, USA. (**A**) Star indicates collection area in South Texas. (**B**) Stars indicate collection areas in the two cities, McAllen, TX and Edinburg, TX. (**C**) The twenty-three collection sites, located within McAllen and Edinburg, TX.

Eggs of *A. aegypti* mosquitoes were collected in a residential area in the lower Rio Grande Valley (Figure 1) using field oviposition cups. Egg collection permission was granted by the residents of the collection sites. Eggs were collected for 39 weeks from March 2019 to December 2019. Oviposition cups were lined with seed germination paper, which acted as a substrate for oviposition. The oviposition traps were filled between ½ and ¾ full of water and left outside residential houses for a week prior to collection. No more than three cups were set at a single residence, and no two adjacent residences were used as field sites. Every week, the seed germination paper was removed and replaced, and fresh water was added to the cup. Once collected, the seed germination paper was stored in resealable plastic bags and returned to the laboratory for hatching. Eggs were hatched using a standard operating procedure for mass rearing in an aerated nutrient broth solution for 24 h [27]. After 24 h, larvae were filtered out and placed in a water-filled 34.3 × 25.4 × 3.8 cm larval tray with a plastic cover. Larvae were fed liver powder daily until pupation. Pupae were transferred to mosquito breeders until adult emergence. Adults were fed 10% sugar water until all pupae emerged. The adults were collected for species verification and sex identification. Morphological characteristics were used to determine species and sex using a dichotomous key [28]. Adult female mosquitoes were euthanized and stored at −20° Celsius until they were processed for DNA extraction. All male mosquitoes were counted and discarded.

### 2.2. DNA Processing

Female *A. aegypti* mosquitoes from each site were randomly selected for DNA extraction and sequencing (Table 1). The number of females ranged from 1 individual from sites 3 and 76, up to 16 individuals from sites 22 and 34, with a median of 3 individual samples per site. We attempted to analyze at least three individuals from each site but were limited by oviposition and successful hatching and rearing. Individual mosquitoes were stored in microcentrifuge tubes.

The mosquitoes were surface sterilized before DNA extraction with a 5% bleach solution for five minutes. After a five-minute surface sterilization, the whole mosquito was crushed using a pestle in the microcentrifuge tube. DNA was extracted using the manufacturer’s protocol: purification of Total DNA from Animal Tissue (Spin-Column Protocol) from the DNeasy Blood and Tissue Kit (Qiagen, Valencia, CA, USA). The COI gene was amplified with the HCO2198 (5′-taaacttcagggtgaccaaaaaatca-3′) and LCO4901(5′-ggtcaacaaatcataaagatattgg-3′) primers [29]. The PCR master mix consisted of 12.5 µL of Premix Taq^TM^ DNA Polymerase (Takara, Mountain View, CA, USA), 1 mM final concentration for each of the primers, 3 µL of extracted DNA, and enough molecular-grade water for a reaction size of 25 µL. The thermocycler conditions modified those of Folmer et al. [28]. The thermocycler conditions were as follows: an initial denaturation at 95 °C for 2 min followed by 35 cycles of 95 °C for 1 min, 50 °C for 1 min, 72 °C for 1.5 min, and a final extension step at 72 °C for 10 min. Gel electrophoresis and ultraviolet visualization were used to validate whether the COI gene was amplified. Amplified products were cleaned with Exo-Sap-It (Affymetrix, Santa Clara, CA, USA) per the manufacturer’s protocol and sent to the University of Chicago Cancer Center DNA Sequencing and Genotyping Facility (Chicago, IL, USA) for sequencing on an ABI 3730 using BigDye^TM^ Terminator sequencing chemistry (ABI, ThermoFisher, Waltham, MA, USA).

### 2.3. Data Analysis of Samples Sequenced

The chromatograms were edited using BioEdit 7.2 [30], and each sequence was manually checked for ambiguous base calls. Any ambiguity between the two technical replicates of the sequence was visually inspected. All sequences were aligned, resulting in a 602-base-pair sequence (GenBank accession numbers: PZ507024-PZ507133). A batch file of the 107 sequences was run through GenBank using the megaBLAST algorithm with a limitation for the database search by organism (*A. aegypti*). This analysis was completed to identify novel COI haplotypes in the dataset (Table 1). Polymorphic sites and DNA polymorphism analyses from DNAsp v6.12.03 [31] were used to determine haplotype diversity for the whole population and per site. Haplotype networks were constructed and visualized using PopART [32]. Three network algorithms were used to construct haplotypes: minimum spanning networks [33], median joining networks [32], and statistical parsimony (TCS) networks [34].

### 2.4. Data Analysis of COI Haplotypes from Worldwide Samples

In order to understand how the haplotype diversity determined from our dataset compares to other *A. aegypti* populations, we collected COI sequences of *A. aegypti* from published studies using the PopSet database in GenBank (now BioProjects). We limited our searches to studies that sequenced the same portion of COI and gave geographic data on their samples (Table 2). We also searched the GenBank Nucleotide database for additional population studies with the same limitations (Table 2).

Polymorphic sites and DNA polymorphism analyses from DNAsp v6.12.03 were used to determine haplotype diversity and other related metrics for each additional study and, when possible, from the published data, population subsets (Table 2). Haplotype networks were constructed and visualized using PopART. As with the newly generated COI sequences, a minimum spanning network algorithm was used to construct algorithms for each location at different geographic scales (Table 3).

## 3. Results

### 3.1. Sequencing Results

In this study, we successfully sequenced a partial COI locus from 107 *A. aegypti* individuals from 23 sites located in the McAllen–Edinburg region in South Texas (Table 1, Figure 1). On average, three individuals were sequenced for COI at 19 of the 23 sites. The number of haplotypes recovered at these sites ranged from 1 to 3 haplotypes. Four sites were more heavily sampled, with 13–16 individuals sampled. The number of haplotypes ranged from 2 to 7. A megaBLAST search of the GenBank nucleotide database resulted in 72 exact matches of the COI sequences to previously reported haplotypes. The 72 sequences were split between two known haplotypes previously reported in GenBank. Nine additional haplotypes from 32 individuals had a single base pair difference from reported sequences in GenBank. Finally, three individuals, each with unique haplotypes, differed by two base pairs from known haplotypes. These 14 haplotypes found in South Texas arose from 21 polymorphic sites, which include 6 singletons and 15 parsimony informative sites.

### 3.2. Comparative Population Dynamics

#### 3.2.1. South Texas Population Dynamics Compared to Nationwide Geographic Scale

To better understand the diversity of the COI haplotypes isolated and possible population dynamics represented by the data, we calculated several measures and statistics for the South Texas population and for eight populations of *A. aegypti* from around the world (Table 2). Overall, the 385 sequences represented 54 COI haplotypes. When comparing the South Texas population to the worldwide populations, several major trends were evident. The East African populations from Kenya, Tanzania, and Uganda produced similar results to each other, producing high values for haplotype diversity (Hd), nucleotide diversity (π), θ_W,_ and the number of segregating sites (S) compared to the other populations and negative values for Tajima’s D and Fu’s F. The populations from Benin (West Africa), Thailand, Ecuador, and El Salvador had comparatively lower values for θ_W_ and positive values for Tajima’s D and Fu’s F (except Benin, which had a negative Fu’s F). The South Texas population had a unique pattern that, while having the second largest number of haplotypes, it also had very low values for haplotype diversity, π, and θ_W_, similar to Benin, Thailand, Ecuador, and El Salvador. This pattern is most likely being driven by the high number of singleton haplotypes, creating an excess of rare alleles. Conversely, Tajima’s ^D^ and Fu’s F for the South Texas population were both negative, like the East African populations, and the South Texas population had a large number of segregating sites similar to these same populations. Tajima’s D, Fu’s and Li’s D, and Fu and Li’s F were not significant for the South Texas population. The Florida–Georgia population also had a unique pattern of high values for haplotype diversity, π and θ_W_, and positive values for Tajima’s D and Fu’s F.

#### 3.2.2. South Texas Population Dynamics Compared to Smaller Geographic Scale

Because the geographic scale of sampling in South Texas was local, we also compared the population dynamics of the South Texas mosquitoes to populations sampled at a smaller scale than nationally (Table 3). The regional scale was estimated using Google Earth to create an approximate and conservative size estimate (km^2^) for the local sampling areas. The estimated sampling areas ranged in size from 1450 to 72.25 km^2^ with an average size of 397 km^2^ (Table 3). These populations had between nine and twenty-six individuals sequenced from one to eight sites per geographic region [20,22]. We sequenced a significantly larger number of mosquitoes, 107, in a single geographic location with 23 individual sites. Additionally, Bennett et al. [20] sampled from 4 to 13 individuals per site, and Joyce et al. [22] sampled from 2 to 4 individuals per site. We sampled 1–16 individuals per site, with the median being 3 individuals sampled per site. The diversity measures and statistics of the subdivisions of Uganda, Tanzania, and El Salvador have the same patterns illustrated by the national data. The South Texas *A. aegypti* population is similar to the two Benin subdivisions in all measures except the number of haplotypes and the number of segregating sites. Again, the excess of rare alleles in South Texas is unique compared to other populations.

### 3.3. Haplotype Networks

**Figure 2:**
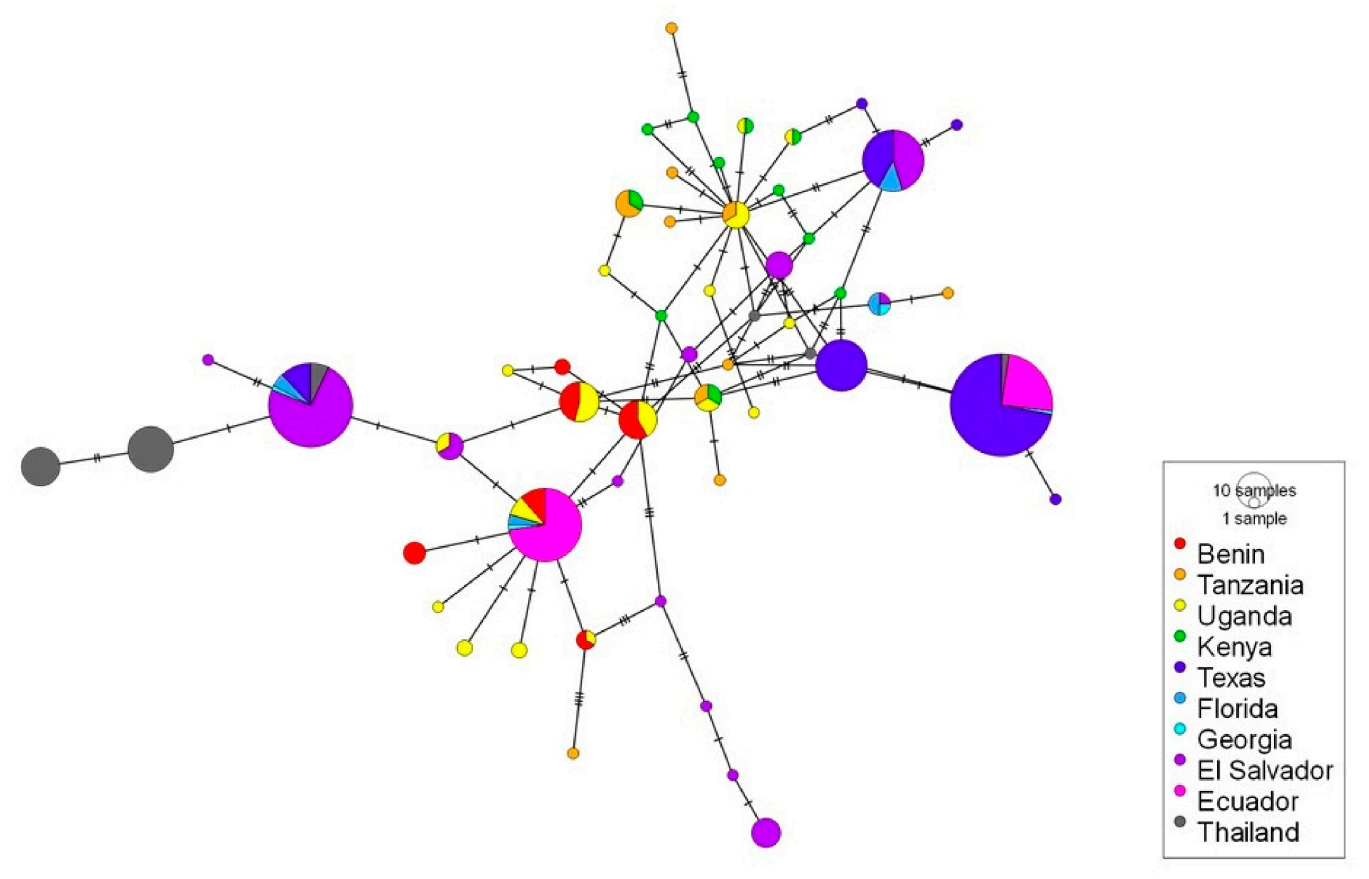
Haplotype network of COI sequences from this study and multiple studies from across the distribution of *A. aegypti.* This minimum spanning haplotype network was constructed with 385 samples from 8 countries and 54 haplotypes.

**Figure 3:**
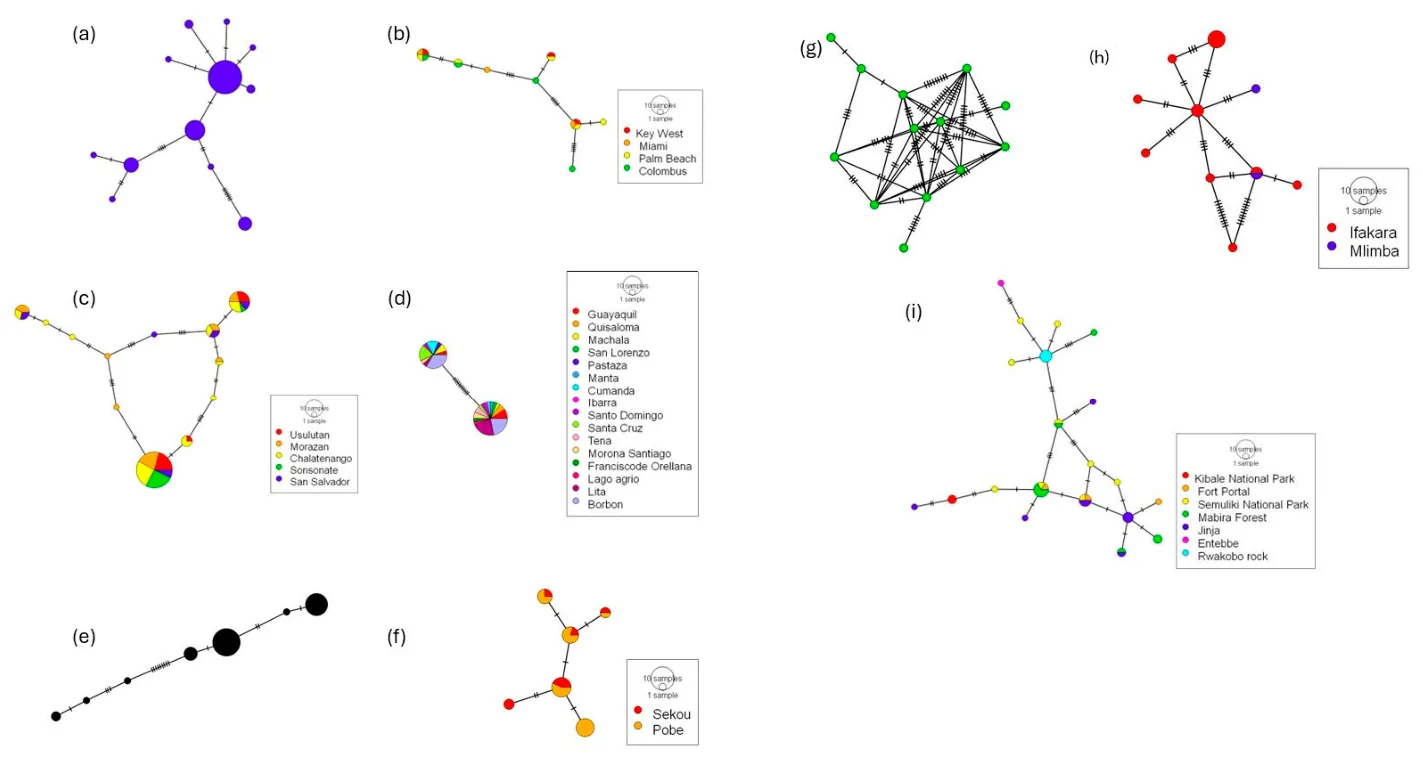
Minimum spanning networks by location with each dash representing a single basepair difference, (**a**) Texas (this study) representing 107 samples and 14 haplotypes, (**b**) Florida and Georgia representing 15 samples and 8 haplotypes, (**c**) El Salvador representing 82 samples and 12 haplotypes, (**d**) Ecuador representing 53 samples and 2 haplotypes, (**e**) Thailand representing 37 samples and 7 haplotypes, (**f**) Benin representing 26 samples and 6 haplotypes, (**g**) Kenya representing 13 samples and 13 haplotypes, (**h**) Tanzania representing 15 samples and 10 haplotypes, (**i**) Uganda representing 37 samples and 20 haplotypes.

**Figure 4:**
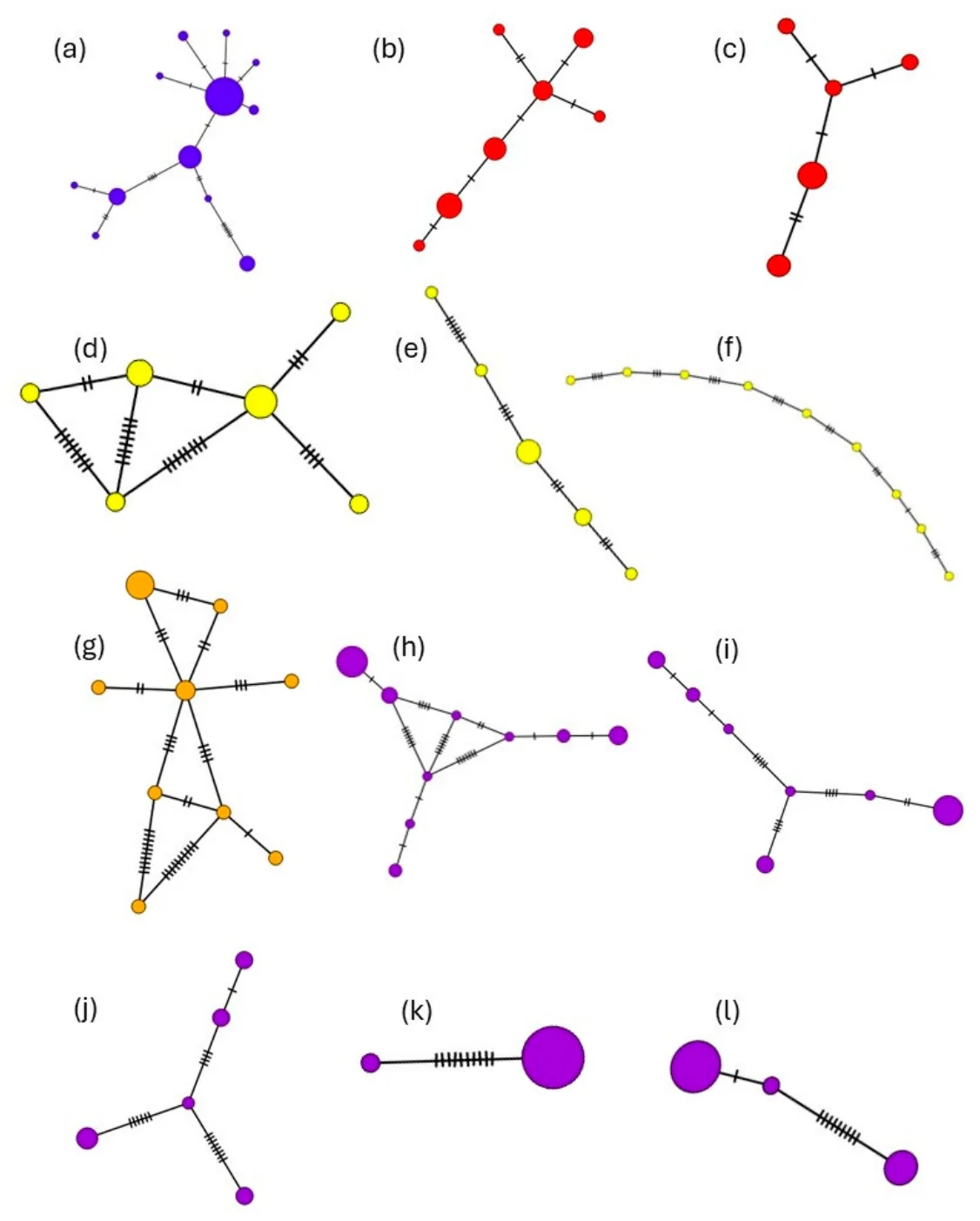
Minimum spanning networks by location with each dash representing a single basepair difference, (**a**) Texas (this study) constructed with 107 samples and 14 haplotypes, (**b**) Pobe, Benin constructed with 18 samples and 7 haplotypes (**c**) Sekou, Benin constructed with 8 samples and 5 haplotypes (**d**) Jinja, Uganda constructed with 9 samples and 6 haplotypes (**e**) Mabira Forest, Uganda constructed with 9 samples and 5 haplotypes (**f**) Semuliki National Park, Uganda constructed with 9 samples and 9 haplotypes (**g**) Ifakara, Tanzania constructed with 13 samples and 9 haplotypes (**h**) Chalatenango, El Salvador constructed with 26 samples and 9 haplotypes (**i**) Morazan, El Salvador constructed with 20 samples and 7 haplotypes (**j**) San Salvador, El Salvador constructed with 10 samples and 5 haplotypes (**k**) Sonsonate, El Salvador constructed with 12 samples and 2 haplotypes (**l**) Usulutan, El Salvador constructed with 14 samples and 3 haplotypes.

Haplotype networks were constructed for all the populations used in Table 2 and Table 3 (Figure 2, Figure 3 and Figure 4 and Supplemental Figures S1 and S2). When possible, site information was also included as a trait to better clarify the distribution of the haplotypes. The minimum spanning haplotype network for all the mosquito COI sequences from South Texas and from other studies (Figure 2) illustrates the presence of several unique alleles found only in South Texas. Three of the South Texas haplotypes are shared with individuals also found in Florida, Georgia, Ecuador, El Salvador, and Thailand. The other four haplotypes from South Texas are unique within this dataset. Three of the unique haplotypes have a single sequence, while the fourth contains 22 sequences from the South Texas sites 1, 21, 22, 27, 29, 36, and 76. This same pattern for the South Texas haplotypes was recovered using the median joining networks (Supplemental Figure S1) and statistical parsimony (TCS) networks (Supplemental Figure S2).

As with the population dynamics, the effects of geographic scale were visualized with minimum spanning networks at the country level (Figure 3) and within-country localities when available (Figure 4). The same patterns exhibited in the population dynamics in Table 2 can be seen in the country-level haplotype networks (Figure 3).

The haplotype networks representing the East African countries of Kenya, Tanzania, and Uganda are the most complex, with many interlinked haplotypes differing by many mutations. The South Texas haplotype network has a similar level of complexity as the Eastern United States (samples from across two states) and El Salvador (samples from across the country) networks. When comparing the small-scale sampling area represented by the South Texas haplotype network to within-country localities (Figure 4), South Texas still exhibits a large number of haplotypes, but with few mutational differences (Figure 4a). This pattern is similar to locations from the two locations in Benin (Figure 4b,c). One notable difference between the South Texas haplotype network and all the other sites (Figure 4d–l) is the number of mutations between the haplotypes. The majority of the South Texas haplotypes vary by a single base pair, compared to multiple base pair mutations in the other sites.

## 4. Discussion

Intensive sampling of the South Texas cities of McAllen and Edinburg revealed an excess of rare haplotypes, evident from comparatively low haplotype diversity, π, and θ, but a comparatively large number of haplotypes and segregating sites and negative Fu’s F. The negative Tajima’s D, though not significant, indicates a possibly expanding population or migration. The haplotype network does not resolve this population dynamic since the unique, rare haplotype found in South Texas may have been missed in previous studies due to low sampling schemes, and there is little sequence data from mosquito populations in the Caribbean or Mexico. However, the low sequence divergence between the most common haplotype found in South Texas and other countries in North and South America, as well as rare, unique haplotypes, could support the creation of new haplotypes in the South Texas region. The importance of the rare alleles is unknown, but these rare haplotype alleles could indicate the need to increase the number of individuals sampled for COI haplotypes to better understand mosquito population structure. Additionally, COI serves as a neutral marker when investigating genetic loci implicated in vector capacity, disease transmission ability, or resistance, such as *kdr* (knockdown resistance mutations) [35,36]; therefore, a robust understanding of the population structure is needed. These haplotypes could also indicate a reservoir for pesticide resistance. Some insecticides, such as pyrethroids, can induce mitochondrial apoptosis, so it is possible that haplotype mutations can be an indicator for insecticide resistance [37,38,39]. Future work linking phenotypic qualities to the haplotype will be needed to understand the role the common and rare alleles have in *A. aegypti* evolution and geographic spread.

In addition to the new COI sequences added from South Texas, we can re-evaluate COI haplotypes from several North and South American populations. Despite a large number of samples over a nationwide geographic scale, only two haplotypes were found in Ecuador populations of *A. aegypti* [21]. One haplotype was shared with the West African population from Benin, and the other is the dominant haplotype found in South Texas. Neither haplotype was found in the El Salvador mosquito population. Limited COI sequencing from Georgia and Florida [18] was also re-evaluated and compared to the South Texas populations. Despite the close geographic distance, only two of the four haplotypes from Florida and Georgia were shared with South Texas. All four haplotypes were also found in El Salvador and Ecuador (Figure 2). The sharing of haplotypes between El Salvador and Georgia and Florida may support the hypothesis of *A. aegypti* entry from Central America through the Caribbean into Florida. More sampling through the Caribbean and Florida is needed, however, to support or refute this hypothesis.

Finally, the El Salvador mosquito population [22] may have had fewer haplotypes than South Texas; however, there was much greater haplotype diversity (Table 2). These haplotypes were also shared across multiple geographic regions, as well as several being unique in this sample set. The haplotype network also supports multiple introductions of mosquitoes from Africa (Uganda, Tanzania, and Benin). Both Tajima’s D and Fu’s F were considerably larger for the El Salvador population than any tested in this study (Table 2). Both statistics support a sudden contraction of alleles or a population bottleneck. This statistical pattern was not recovered in the other three populations from Central and North America. More intensive sampling of Central America will be vital to understanding the movement of *A. aegypti* in North America.

## 5. Conclusions

More rigorous sampling at a small geographic scale revealed increased evolutionary and genetic potential that could counter controlling the spread of *A. aegypti*. More intensive sampling in other locations could inform whether these rare haplotypes are new sequences in South Texas or are found in low frequencies in other locations. Increased sampling at either small or larger geographic scales is also needed to understand the spread of *A. aegypti* into North and Central America.

## Figures and Tables

**Table 1:** Sites and individuals sampled and successfully sequenced for COI haplotype determination.

Site Number	Individuals Sampled	GIS Location	Geographic Location	Number of Haplotypes per Site
1	1 ^a^, 2 ^a^, 3	26.209801, −98.224604	McAllen, TX, USA	2
3	3 ^a^	26.209357, −98.224458	McAllen, TX, USA	1
4	1, 2, 3	26.209219, −98.224434	McAllen, TX, USA	1
6	1, 2, 3	26.210861, −98.224408	McAllen, TX, USA	2
10	1, 2, 3	26.211109, −98.226621	McAllen, TX, USA	1
13	1, 2, 3, 4, 5 ^a^, 6 ^a^, 7 ^b^, 8, 9, 10, 11 ^a^, 15, 16	26.205846, −98.227592	McAllen, TX, USA	7
17	1 ^a^, 2, 3 ^a^	26.209711, −98.225805,26	McAllen, TX, USA	2
18	1, 2, 3	26.209645, −98.225796,	McAllen, TX, USA	1
21	1 ^a^, 2, 3 ^a^, 4 ^a^, 5 ^a^, 6, 7 ^a^, 8 ^a^, 9 ^a^, 10, 13, 14, 15, 16 ^a^	26.213114, −98.225132,	McAllen, TX, USA	5
22	1, 2, 3 ^a^, 4 ^a^, 5 ^a^, 6, 7 ^a^, 8, 9, 10, 11 ^a^, 12, 13 ^a^, 14 ^a^, 15 ^a^, 16	26.213025, −98.225033	McAllen, TX, USA	2
23	1, 2, 3	26.212846, −98.22564	McAllen, TX, USA	1
27	1 ^a^, 2 ^a^, 3 ^a^	26.214131, −98.222776	McAllen, TX, USA	1
29	1 ^a^, 2 ^a^	26.21202, −98.222734	McAllen, TX, USA	1
30	1, 2, 3	26.211854, −98.222754,	McAllen, TX, USA	1
31	1, 2, 3 ^a^	26.20916, −98.222543	McAllen, TX, USA	3
34	1, 2, 3, 4, 5, 6, 7, 8, 9 ^b^, 10, 11, 12, 13, 14, 15, 16	26.204069, −98.223219	McAllen, TX, USA	3
36	1 ^a^, 2, 3	26.204906, −98.224926	McAllen, TX, USA	2
66	1, 2, 3	26.287733, −98.17125	Edinburg, TX, USA	2
68	1, 2, 3	26.28298, −98.174519	Edinburg, TX, USA	2
74	1, 3	26.282316, −98.173618	Edinburg, TX, USA	1
76	1 ^b^	26.281791, −98.173774	Edinburg, TX, USA	1
79	2 ^a^, 3	26.292161, −98.244824	McAllen, TX, USA	2
82	1, 2	26.292151, −98.244571	McAllen, TX, USA	1

^a^ Sequence did not have a 100% match to the GenBank database. The closest alignment in GenBank had a 1 base pair difference from the sequence in this study. ^b^ Sequence did not have a 100% match to the GenBank database. The closest alignment in GenBank had a 2 base pair difference from the sequence in this study.

**Table 2:** Haplotype diversity measures and statistics for *A. aegypti* from South Texas populations and worldwide populations.

Sequence Set	GenBank Accession Numbers	Number of Samples	Nucleotide Sites Used in the Analysis	Number of Haplotypes	Hd	π	θ	Tajima’s D	Fu’s F	S	Study
This Study	PZ507024-133	107	599	14	0.678 ± 0.041	0.00466	0.00668	−0.87731	−1.679	21	This Study
Florida and Georgia, USA	MF371160-74	15	507	8	0.895 ± 0.053	0.01142	0.0091	1.01884	0.24	15	[18]
Thailand	KM613046-82	37	602	7	0.703 ± 0.054	0.00614	0.00597	0.09044	2.12	15	Direct Submission 2015
Kenya	MK300216-29	13	602	13	1 ± 0.03	0.00897	0.01338	−1.54837	−8.936	25	[19]
Benin	KX446392-417	26	452	6	0.834 ± 0.033	0.00352	0.00348	0.03468	−0.492	6	[20]
Tanzania	KX446455-69	15	602	10	0.924 ± 0.053	0.00946	0.01226	−0.94812	−1.688	24	[20]
Uganda	KX446418-54	37	549	20	0.949 ± 0.019	0.0079	0.00916	−0.46306	−8.688	21	[20]
Ecuador	MK890401-53	53	602	2	0.488 ± 0.031	0.00891	0.00403	3.49612	16.091	11	[21]

**Table 3:** Haplotype diversity and statistical metrics for *A. aegypti* populations from South Texas compared to populations at a similar geographic scale.

Sequence Set	Estimated Geographic Range (km^2^)	Number of Samples	Number of Sampling Sites	Nucleotide Sites Used in the Analysis	Number of Haplotypes	Hd	π	θ	Tajima’s D	Fu’s F	S
This Study	278	107	23	599	14	0.678 ± 0.04	0.00466	0.00668	−0.87731	−1.679	21
Pobe, Benin	400	18	2	518	7	0.856 ± 0.04	0.00332	0.00337	−0.04805	−1.906	6
Sekou Benin	381	8	1	452	5	0.857 ± 0.10	0.00419	0.00427	−0.08352	−1.273	5
Ifakara, Tanzania	390	13	1	602	9	0.91 ± 0.068	0.00937	0.01124	−0.71436	−1.406	21
Jinja, Uganda	768	9	1	602	6	0.889 ± 0.09	0.00757	0.01039	−1.3226	−0.209	17
Mabira Forest, Uganda	306	9	2	590	5	0.806 ± 0.12	0.0081	0.00935	−0.6481	1.099	15
Semuliki National Park, Uganda	220	9	1	602	9	1 ± 0.052	0.01089	0.01100	−0.04966	−3.984	18
Chalatenango, El Salvador	132	26	6	488	9	0.797 ± 0.068	0.01112	0.00806	1.32183	1.05	15
Sonsonate, El Salvador	232.5	12	6	498	2	0.167 ± 0.13	0.00335	0.00665	−2.0434	3.755	10
Morazan, El Salvador	1450	20	8	495	7	0.774 ± 0.08	0.01191	0.00854	1.4547	2.269	15
San Salvador, El Salvador	72.25	10	8	480	5	0.867 ± 0.07	0.01398	0.01105	1.22382	2.382	15
Usulutan, El Salvador	140	14	7	516	3	0.538 ± 0.11	0.00863	0.00609	1.61718	5.648	10

## Data Availability

Sequences can be found on the NCBI GenBank accession numbers PZ507024-PZ507133.

## Supplementary Materials

The following supporting information can be downloaded at: https://www.mdpi.com/article/10.3390/insects17090879/s1, Figure S1: Median Joining Network of all haplotypes; Figure S2: TCS Network of all haplotypes, File S1: Sequential FASTA for all sequences used in the analysis.
