# Supplementary material for: South Texas Is Home to High Diversity of Cytochrome Oxidase I Haplotypes Compared to Other Invasive Populations of Aedes aegypti
Source: Insects. 2026 Aug 22;17(9):879. doi: 10.3390/insects17090879 (PMC13607572; doi:10.3390/insects17090879)
Supplement: Supplementary file 1 [file insects-17-00879-s001.zip › insects-4396296-supplementary.pdf]

Supplemental Figures

Figure S1. Median Joining Network of all haplotypes

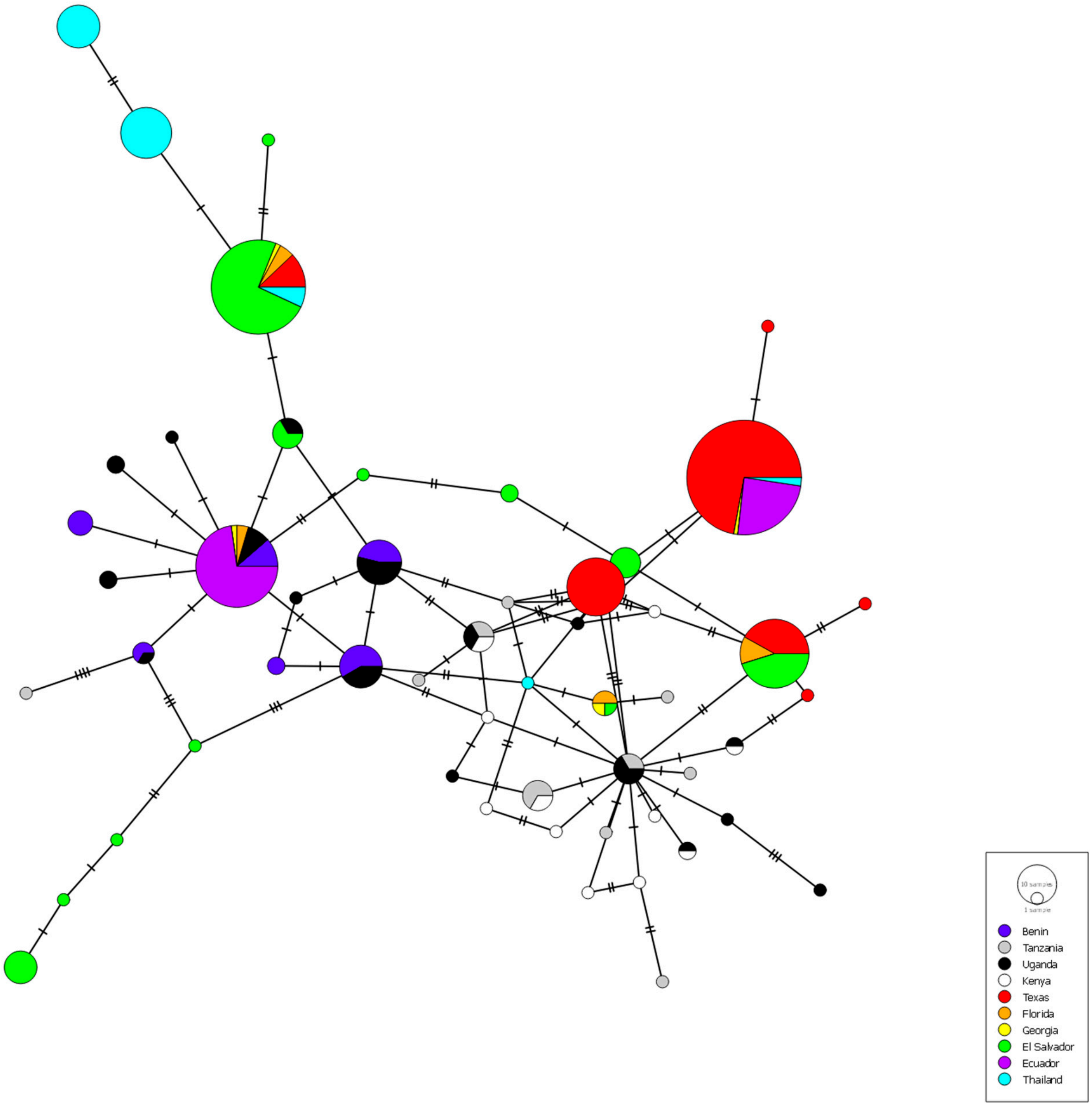

Figure S2. TCS Network of all haplotypes

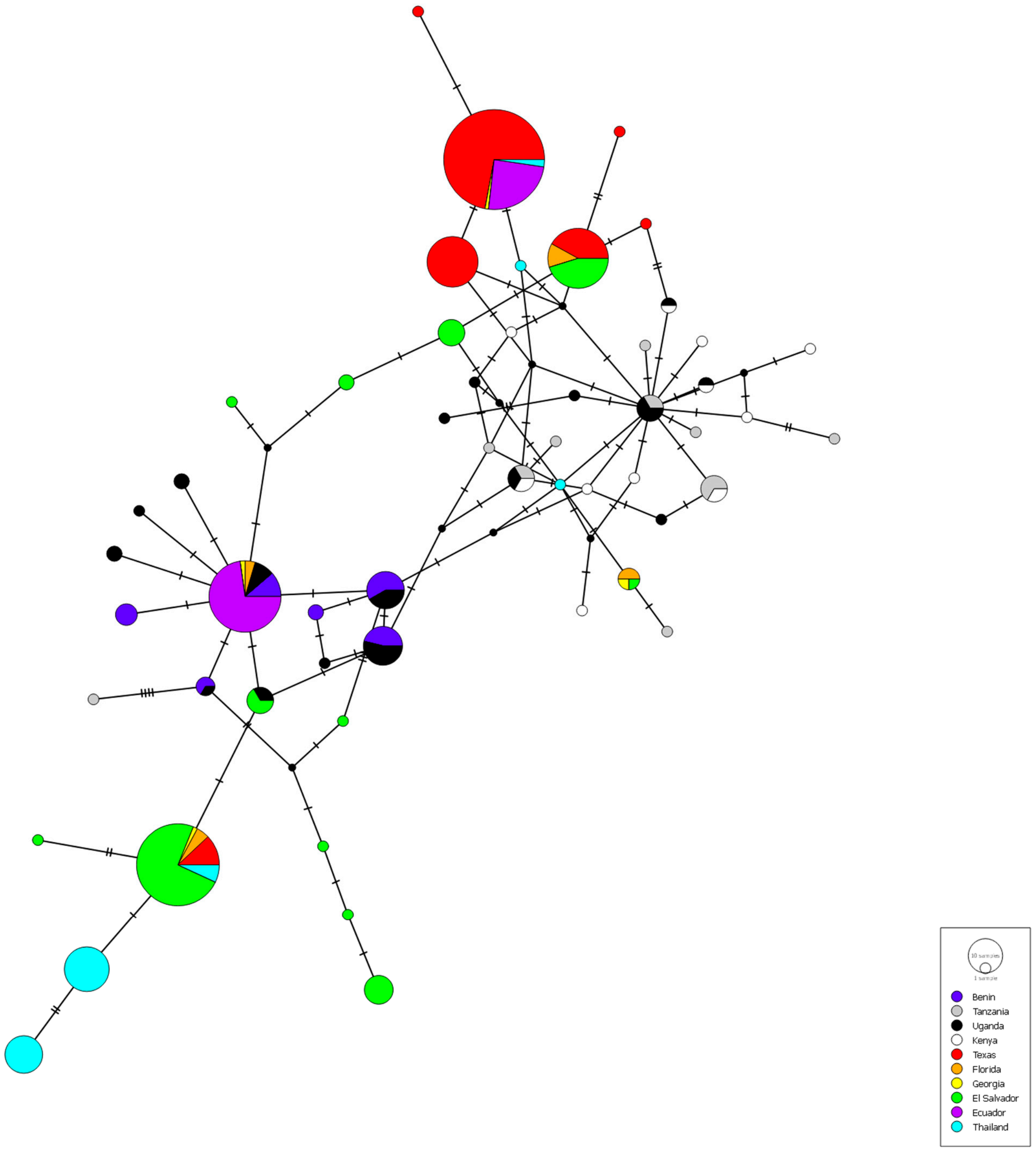

File S1. Sequential FASTA entries for all sequences used in the analysis

```
>KX446392.1
TCGGAACTTCTCTAAGAATTTTAATTCGTGCTGAACTTAGCCACCCTGGTATATTTATTG
GGAATGACCAAATTTATAATGTAATTGTAACAGCTCATGCATTTATTATAATTTTCTTTA
TAGTAATACCAATTATAATTGGAGGATTTGGAAATTGATTAGTTCCTTTAATATTAGGAG
CCCCTGATATAGCCTTTCCTCGAATAAATAATATAAGTTTTTGAATACTACCTCCTTCAT
TGACTCTTCTATTATCAAGCTCAATAGTAGAAAATGGAGCAGGAACTGGGTGAACAGTTT
ATCCTCCTCTCTCTTCAGGAACAGCTCATGCTGGAGCTTCTGTTGATTTAGCTATTTTTT
CTCTTCATTTAGCTGGAATTTCCTCAATTTTAGGAGCAGTAAATTTTATTACAACTGTAA
TTAATATACGATCGTCAGGAATTACTTTAGATCGACTACCCTTATTTGTTTGATCTGTAG
TTATTACAGCTATCTTATTACTTCTTTCTCTTCCTGTTTTAGCTGGAGCTATTACTATGT
TATTAACAGACCGAAACTTAAATACATCTTTCTTTGATCCAATCGGAGGAGGAGATCCTA
TT
>KX446393.1
TCGGAACTTCTTTAAGAATTTTAATTCGTGCTGAACTTAGCCACCCTGGTATATTTATTG
GGAATGACCAAATTTATAATGTAATTGTAACAGCTCATGCATTTATTATAATTTTCTTTA
TAGTAATGCCAATTATAATTGGAGGATTTGGAAATTGATTAGTTCCTTTAATATTAGGAG
CCCCTGATATAGCTTTTCCTCGAATAAATAATATAAGTTTTTGAATACTACCCCCTTCAT
TGACTCTTCTATTATCAAGCTCAATAGTAGAAAATGGAGCAGGAACTGGGTGAACAGTTT
ATCCTCCTCTCTCTTCAGGAACAGCTCATGCTGGAGCTTCTGTTGATTTAGCTATTTTTT
CTCTTCATTTAGCTGGAATTTCCTCAATTTTAGGGGCAGTAAATTTTATTACAACTGTAA
TTAATATACGATCGTCAGGAATTACTTTAGATCGACTACCCTTATTTGTTTGATCTGTAG
TTATTACAGCTATCTTATTACTTCTTTCTCTTCCTGTTTTAGCTGGAGCTATTACTATGT
TATTAACAGACCGAAACTTAAATACATCTTTCTTTGATCCAATCGGAGGAGGAGATCCTA
TT
>KX446394.1
TCGGAACTTCTTTAAGAATTTTAATTCGTGCTGAACTTAGCCACCCTGGTATATTTATTG
GGAATGACCAAATTTATAATGTAATTGTAACAGCTCATGCATTTATTATAATTTTCTTTA
TAGTAATGCCAATTATAATTGGAGGATTTGGAAATTGATTAGTTCCTTTAATATTAGGAG
CCCCTGATATAGCTTTTCCTCGAATAAATAATATAAGTTTTTGAATACTACCCCCTTCAT
TGACTCTTCTATTATCAAGCTCAATAGTAGAAAATGGAGCAGGAACTGGGTGAACAGTTT
ATCCTCCTCTCTCTTCAGGAACAGCTCATGCTGGAGCTTCTGTTGATTTAGCTATTTTTT
CTCTTCATTTAGCTGGAATTTCCTCAATTTTAGGGGCAGTAAATTTTATTACAACTGTAA
TTAATATACGATCGTCAGGAATTACTTTAGATCGACTACCCTTATTTGTTTGATCTGTAG
TTATTACAGCTATCTTATTACTTCTTTCTCTTCCTGTTTTAGCTGGAGCTATTACTATGT
TATTAACAGACCGAAACTTAAATACATCTTTCTTTGATCCAATCGGAGGAGGAGATCCTA
TT
>KX446395.1
------------------------------------------------------------
------------------------TTGTAACAGCTCATGCATTTATTATAATTTTCTTTA
TAGTAATACCAATTATAATTGGAGGATTTGGAAATTGATTAGTTCCTTTAATATTAGGAG
CCCCTGATATAGCCTTTCCTCGAATAAATAATATAAGTTTTTGAATACTACCTCCTTCAT
TGACTCTTCTATTATCAAGCTCAATAGTAGAAAATGGAGCAGGAACTGGGTGAACAGTTT
ATCCTCCTCTCTCTTCAGGAACAGCTCATGCTGGAGCTTCTGTTGATTTAGCTATTTTTT
CTCTTCATTTAGCTGGAATTTCCTCAATTTTAGGGGCAGTAAATTTTATTACAACTGTAA
TTAATATACGATCGTCAGGAATTACTTTAGATCGACTACCCTTATTTGTTTGATCTGTAG
TTATTACAGCTATCTTATTACTTCTTTCTCTTCCTGTTTTAGCTGGAGCTATTACTATGT
TATTAACAGACCGAAACTTAAATACATCTTTCTTTGATCCAATCGGAGGAGGAGATCCTA
TT
>KX446396.1
------------------------------------------------------------
------------------------TTGTAACAGCTCATGCATTTATTATAATTTTCTTTA
TAGTAATACCAATTATAATTGGAGGATTTGGAAATTGATTAGTTCCTTTAATATTAGGAG
```

CCCCTGATATAGCCTTTCCTCGAATAAATAATATAAGTTTTTGAATACTACCTCCTTCAT
TGACTCTTCTATTATCAAGCTCAATAGTAGAAAATGGAGCAGGAACTGGGTGAACAGTTT
ATCCTCCTCTCTCTTCAGGAACAGCTCATGCTGGAGCTTCTGTTGATTTAGCTATTTTTT
CTCTTCATTTAGCTGGAATTTCCTCAATTTTAGGGGCAGTAAATTTTATTACAACTGTAA
TTAATATACGATCATCAGGAATTACTTTAGATCGACTACCCTTATTTGTTTGATCTGTAG
TTATTACAGCTATCTTATTACTTCTTTCTCTTCCTGTTTTAGCTGGAGCTATTACTATGT
TATTAACAGACCGAAACTTAAATACATCTTTCTTTGATCCAATCGGAGGAGGAGATCCTA
TT
>KX446397.1
TCGGAACTTCTTTAAGAATTTTAATTCGTGCTGAACTTAGCCACCCTGGTATATTTATTG
GGAATGACCAAATTTATAATGTAATTGTAACAGCTCATGCATTTATTATAATTTTCTTTA
TAGTAATACCAATTATAATTGGAGGATTTGGAAATTGATTAGTTCCTTTAATATTAGGAG
CCCCTGATATAGCTTTTCCTCGAATAAATAATATAAGTTTTTGAATACTACCTCCTTCAT
TGACTCTTCTATTATCAAGCTCAATAGTAGAAAATGGAGCAGGAACTGGGTGAACAGTTT
ATCCTCCTCTCTCTTCAGGAACAGCTCATGCTGGAGCTTCTGTTGATTTAGCTATTTTTT
CTCTTCATTTAGCTGGAATTTCCTCAATTTTAGGGGCAGTAAATTTTATTACAACTGTAA
TTAATATACGATCGTCAGGAATTACTTTAGATCGACTACCCTTATTTGTTTGATCTGTAG
TTATTACAGCTATCTTATTACTTCTTTCTCTTCCTGTTTTAGCTGGAGCTATTACTATGT
TATTAACAGACCGAAACTTAAATACATCTTTCTTTGATCCAATCGGAGGAGGAGATCCTA
TT
>KX446398.1
TCGGAACTTCTTTAAGAATTTTAATTCGTGCTGAACTTAGCCACCCTGGTATATTTATTG
GGAATGACCAAATTTATAATGTAATTGTAACAGCTCATGCATTTATTATAATTTTCTTTA
TAGTAATACCAATTATAATTGGAGGATTTGGAAATTGATTAGTTCCTTTAATATTAGGAG
CCCCTGATATAGCTTTTCCTCGAATAAATAATATAAGTTTTTGAATACTACCTCCTTCAT
TGACTCTTCTATTATCAAGCTCAATAGTAGAAAATGGAGCAGGAACTGGGTGAACAGTTT
ATCCTCCTCTCTCTTCAGGAACAGCTCATGCTGGAGCTTCTGTTGATTTAGCTATTTTTT
CTCTTCATTTAGCTGGAATTTCCTCAATTTTAGGGGCAGTAAATTTTATTACAACTGTAA
TTAATATACGATCGTCAGGAATTACTTTAGATCGACTACCCTTATTTGTTTGATCTGTAG
TTATTACAGCTATCTTATTACTTCTTTCTCTTCCTGTTTTAGCTGGAGCTATTACT----

--
>KX446399.1
TCGGAACTTCTTTAAGAATTTTAATTCGTGCTGAACTTAGCCACCCTGGTATATTTATTG
GGAATGACCAAATTTATAATGTAATTGTAACAGCTCATGCATTTATTATAATTTTCTTTA
TAGTAATACCAATTATAATTGGAGGATTTGGAAATTGATTAGTTCCTTTAATATTAGGAG
CCCCTGATATAGCTTTTCCTCGAATAAATAATATAAGTTTTTGAATACTACCTCCTTCAT
TGACTCTTCTATTATCAAGCTCAATAGTAGAAAATGGAGCAGGAACTGGGTGAACAGTTT
ATCCTCCTCTCTCTTCAGGAACAGCTCATGCTGGAGCTTCTGTTGATTTAGCTATTTTTT
CTCTTCATTTAGCTGGAATTTCCTCAATTTTAGGGGCAGTAAATTTTATTACAACTGTAA
TTAATATACGATCGTCAGGAATTACTTTAGATCGACTACCCTTATTTGTTTGATCTGTAG
TTATTACAGCTATCTTATTACTTCTTTCTCTTCCTGTTTTAGCTGGAGCTATTACTATGT
TATTAACAGACCGAAACTTAAATACATCTTTCTTTGATCCAATCGGAGGAGGAGATCCTA
TT
>KX446400.1
TCGGAACTTCTTTAAGAATTTTAATTCGTACTGAACTTAGCCACCCTGGTATATTTATTG
GGAATGACCAAATTTATAATGTAATTGTAACAGCTCATGCATTTATTATAATTTTCTTTA
TAGTAATACCAATTATAATTGGAGGATTTGGAAATTGATTAGTTCCTTTAATATTAGGAG
CCCCTGATATAGCTTTTCCTCGAATAAATAATATAAGTTTTTGAATACTACCTCCTTCAT
TGACTCTTCTATTATCAAGCTCAATAGTAGAAAATGGAGCAGGAACTGGGTGAACAGTTT
ATCCTCCTCTCTCTTCAGGAACAGCTCATGCTGGAGCTTCTGTTGATTTAGCTATTTTTT
CTCTTCATTTAGCTGGAATTTCCTCAATTTTAGGGGCAGTAAATTTTATTACAACTGTAA
TTAATATACGATCGTCAGGAATTACTTTAGATCGACTACCCTTATTTGTTTGATCTGTAG
TTATTACAGCTATCTTATTACTTCTTTCTCTTCCTGTTTTAGCTGGAGCTATTACTATGT
TATTAACAGACCGAAACTTAAATACATCTTTCTTTGATCCAATCGGAGGAGGAGATCCTA
TT
>KX446401.1

TCGGAACTTCTTTAAGAATTTTAATTCGTGCTGAACTTAGCCACCCTGGTATATTTATTG
GGAATGACCAAATTTATAATGTAATTGTAACAGCTCATGCATTTATTATAATTTTCTTTA
TAGTAATACCAATTATAATTGGAGGATTTGGAAATTGATTAGTTCCTTTAATATTAGGAG
CCCCTGATATAGCTTTTCCTCGAATAAATAATATAAGTTTTTGAATACTACCTCCTTCAT
TGACTCTTCTATTATCAAGCTCAATAGTAGAAAATGGGGCAGGAACTGGGTGAACAGTTT
ATCCTCCTCTCTCTTCAGGAACAGCTCATGCTGGAGCTTCTGTTGATTTAGCTATTTTTT
CTCTTCATTTAGCTGGAATTTCCTCAATTTTAGGGGCAGTAAATTTTATTACAACTGTAA
TTAATATACGATCGTCAGGAATTACTTTAGATCGACTACCCTTATTTGTTTGATCTGTAG
TTATTACAGCTATCTTATTACTTCTTTCTCTTCCTGTTTTAGCTGGAGCTATTACTATGT
TATTAACAGACCGAAACTTAAATACATCTTTCTTTGATCCAATCGGAGGAGGAGATCCTA
TT
>KX446402.1
TCGGAACTTCTCTAAGAATTTTAATTCGTGCTGAACTTAGCCACCCTGGTATATTTATTG
GGAATGACCAAATTTATAATGTAATTGTAACAGCTCATGCATTTATTATAATTTTCTTTA
TAGTAATACCAATTATAATTGGAGGATTTGGAAATTGATTAGTTCCTTTAATATTAGGAG
CCCCTGATATAGCCTTTCCTCGAATAAATAATATAAGTTTTTGAATACTACCTCCTTCAT
TGACTCTTCTATTATCAAGCTCAATAGTAGAAAATGGAGCAGGAACTGGGTGAACAGTTT
ATCCTCCTCTCTCTTCAGGAACAGCTCATGCTGGAGCTTCTGTTGATTTAGCTATTTTTT
CTCTTCATTTAGCTGGAATTTCCTCAATTTTAGGGGCAGTAAATTTTATTACAACTGTAA
TTAATATACGATCGTCAGGAATTACTTTAGATCGACTACCCTTATTTGTTTGATCTGTAG
TTATTACAGCTATCTTATTACTTCTTTCTCTTCCTGTTTTAGCTGGAGCTATTACTATGT
TATTAACAGACCGAAACTTAAATACATCTTTCTTTGATCCAATCGGAGGAGGAGATCCTA
TT
>KX446403.1
TCGGAACTTCTTTAAGAATTTTAATTCGTGCTGAACTTAGCCACCCTGGTATATTTATTG
GGAATGACCAAATTTATAATGTAATTGTAACAGCTCATGCATTTATTATAATTTTCTTTA
TAGTAATACCAATTATAATTGGAGGATTTGGAAATTGATTAGTTCCTTTAATATTAGGAG
CCCCTGATATAGCTTTTCCTCGAATAAATAATATAAGTTTTTGAATACTACCTCCTTCAT
TGACTCTTCTATTATCAAGCTCAATAGTAGAAAATGGGGCAGGAACTGGGTGAACAGTTT
ATCCTCCTCTCTCTTCAGGAACAGCTCATGCTGGAGCTTCTGTTGATTTAGCTATTTTTT
CTCTTCATTTAGCTGGAATTTCCTCAATTTTAGGGGCAGTAAATTTTATTACAACTGTAA
TTAATATACGATCGTCAGGAATTACTTTAGATCGACTACCCTTATTTGTTTGATCTGTAG
TTATTACAGCTATCTTATTACTTCTTTCTCTTCCTGTTTTAGCTGGAGCTATTACTATGT
TATTAACAGACCGAAACTTAAATACATCTTTCTTTGATCCAATCGGAGGAGGAGATCCTA
TT
>KX446404.1
TCGGAACTTCTTTAAGAATTTTAATTCGTGCTGAACTTAGCCACCCTGGTATATTTATTG
GGAATGACCAAATTTATAATGTAATTGTAACAGCTCATGCATTTATTATAATTTTCTTTA
TAGTAATACCAATTATAATTGGAGGATTTGGAAATTGATTAGTTCCTTTAATATTAGGAG
CCCCTGATATAGCTTTTCCTCGAATAAATAATATAAGTTTTTGAATACTACCTCCTTCAT
TGACTCTTCTATTATCAAGCTCAATAGTAGAAAATGGGGCAGGAACTGGGTGAACAGTTT
ATCCTCCTCTCTCTTCAGGAACAGCTCATGCTGGAGCTTCTGTTGATTTAGCTATTTTTT
CTCTTCATTTAGCTGGAATTTCCTCAATTTTAGGGGCAGTAAATTTTATTACAACTGTAA
TTAATATACGATCGTCAGGAATTACTTTAGATCGACTACCCTTATTTGTTTGATCTGTAG
TTATTACAGCTATCTTATTACTTCTTTCTCTTCCTGTTTTAGCTGGAGCTATTACTATGT
TATTAACAGACCGAAACTTAAATACATCTTTCTTTGATCCAATCGGAGGAGGAGATCCTA
TT
>KX446405.1

------------------------TTGTAACAGCTCATGCATTTATTATAATTTTCTTTA
TAGTAATACCAATTATAATTGGAGGATTTGGAAATTGATTAGTTCCTTTAATATTAGGAG
CCCCTGATATAGCCTTTCCTCGAATAAATAATATAAGTTTTTGAATACTACCTCCTTCAT
TGACTCTTCTATTATCAAGCTCAATAGTAGAAAATGGAGCAGGAACTGGGTGAACAGTTT
ATCCTCCTCTCTCTTCAGGAACAGCTCATGCTGGAGCTTCTGTTGATTTAGCTATTTTTT
CTCTTCATTTAGCTGGAATTTCCTCAATTTTAGGGGCAGTAAATTTTATTACAACTGTAA
TTAATATACGATCGTCAGGAATTACTTTAGATCGACTACCCTTATTTGTTTGATCTGTAG
TTATTACAGCTATCTTATTACTTCTTTCTCTTCCTGTTTTAGCTGGAGCTATTACTATGT

TATTAACAGACCGAAACTTAAATACATCTTTCTTTGATCCAATCGGAGGAGGAGATCCTA
TT
>KX446406.1
---TTTATTG
GGAATGACCAAATTTATAATGTAATTGTAACAGCTCATGCATTTATTATAATTTTCTTTA
TAGTAATACCAATTATAATTGGAGGATTTGGAAATTGATTAGTTCCTTTAATATTAGGAG
CCCCTGATATAGCCTTTCCTCGAATAAATAATATAAGTTTTTGAATACTACCTCCTTCAT
TGACTCTTCTATTATCAAGCTCAATAGTAGAAAATGGAGCAGGAACTGGGTGAACAGTTT
ATCCTCCTCTCTCTTCAGGAACAGCTCATGCTGGAGCTTCTGTTGATTTAGCTATTTTTT
CTCTTCATTTAGCTGGAATTTCCTCAATTTTAGGAGCAGTAAATTTTATTACAACTGTAA
TTAATATACGATCGTCAGGAATTACTTTAGATCGACTACCCTTATTTGTTTGATCTGTAG
TTATTACAGCTATCTTATTACTTCTTTCTCTTCCTGTTTTAGCTGGAGCTATTACTATGT
TATTAACAGACCGAAACTTAAATACATCTTTCTTTGATCCAATCGGAGGAGGAGATCCTA
TT
>KX446407.1
TCGGAACTTCTTTAAGAATTTTAATTCGTGCTGAACTTAGCCACCCTGGTATATTTATTG
GGAATGACCAAATTTATAATGTAATTGTAACAGCTCATGCATTTATTATAATTTTCTTTA
TAGTAATACCAATTATAATTGGAGGATTTGGAAATTGATTAGTTCCTTTAATATTAGGAG
CCCCTGATATAGCTTTTCCTCGAATAAATAATATAAGTTTTTGAATACTACCTCCTTCAT
TGACTCTTCTATTATCAAGCTCAATAGTAGAAAATGGAGCAGGAACTGGGTGAACAGTTT
ATCCTCCTCTCTCTTCAGGAACAGCTCATGCTGGAGCTTCTGTTGATTTAGCTATTTTTT
CTCTTCATTTAGCTGGAATTTCCTCAATTTTAGGGGCAGTAAATTTTATTACAACTGTAA
TTAATATACGATCGTCAGGAATTACTTTAGATCGACTACCCTTATTTGTTTGATCTGTAG
TTATTACAGCTATCTTATTACTTCTTTCTCTTCCTGTTTTAGCTGGAGCTATTACTATGT
TATTAACAGACCGAAACTTAAATACATCTTTCTTTGATCCAATCGGAGGAGGAGATCCTA
TT
>KX446408.1
TCGGAACTTCTTTAAGAATTTTAATTCGTGCTGAACTTAGCCACCCTGGTATATTTATTG
GGAATGACCAAATTTATAATGTAATTGTAACAGCTCATGCATTTATTATAATTTTCTTTA
TAGTAATACCAATTATAATTGGAGGATTTGGAAATTGATTAGTTCCTTTAATATTAGGAG
CCCCTGATATAGCTTTTCCTCGAATAAATAATATAAGTTTTTGAATACTACCTCCTTCAT
TGACTCTTCTATTATCAAGCTCAATAGTAGAAAATGGGGCAGGAACTGGGTGAACAGTTT
ATCCTCCTCTCTCTTCAGGAACAGCTCATGCTGGAGCTTCTGTTGATTTAGCTATTTTTT
CTCTTCATTTAGCTGGAATTTCCTCAATTTTAGGGGCAGTAAATTTTATTACAACTGTAA
TTAATATACGATCGTCAGGAATTACTTTAGATCGACTACCCTTATTTGTTTGATCTGTAG
TTATTACAGCTATCTTATTACTTCTTTCTCTTCCTGTTTTAGCTGGAGCTATTACTATGT
TATTAACAGACCGAAACTTAAATACATCTTTCTTTGATCCAATCGGAGGAGGAGATCCTA
TT
>KX446409.1
TCGGAACTTCTCTAAGAATTTTAATTCGTGCTGAACTTAGCCACCCTGGTATATTTATTG
GGAATGACCAAATTTATAATGTAATTGTAACAGCTCATGCATTTATTATAATTTTCTTTA
TAGTAATACCAATTATAATTGGAGGATTTGGAAATTGATTAGTTCCTTTAATATTAGGAG
CCCCTGATATAGCCTTTCCTCGAATAAATAATATAAGTTTTTGAATACTACCTCCTTCAT
TGACTCTTCTATTATCAAGCTCAATAGTAGAAAATGGAGCAGGAACTGGGTGAACAGTTT
ATCCTCCTCTCTCTTCAGGAACAGCTCATGCTGGAGCTTCTGTTGATTTAGCTATTTTTT
CTCTTCATTTAGCTGGAATTTCCTCAATTTTAGGGGCAGTAAATTTTATTACAACTGTAA
TTAATATACGATCGTCAGGAATTACTTTAGATCGACTACCCTTATTTGTTTGATCTGTAG
TTATTACAGCTATCTTATTACTTCTTTCTCTTCCTGTTTTAGCTGGAGCTATTACTATGT
TATTAACAGACCGAAACTTAAATACATCTTTCTTTGATCCAATCGGAGGAGGAGATCCTA
TT
>KX446410.1
TCGGAACTTCTTTAAGAATTTTAATTCGTGCTGAACTTAGCCACCCTGGTATATTTATTG
GGAATGACCAAATTTATAATGTAATTGTAACAGCTCATGCATTTATTATAATTTTCTTTA
TAGTAATACCAATTATAATTGGAGGATTTGGAAATTGATTAGTTCCTTTAATATTAGGAG
CCCCTGATATAGCTTTTCCTCGAATAAATAATATAAGTTTTTGAATACTACCTCCTTCAT
TGACTCTTCTATTATCAAGCTCAATAGTAGAAAATGGAGCAGGAACTGGGTGAACAGTTT
ATCCTCCTCTCTCTTCAGGAACAGCTCATGCTGGAGCTTCTGTTGATTTAGCTATTTTTT

CTCTTCATTTAGCTGGAATTTCCTCAATTTTAGGGGCAGTAAATTTTATTACAACTGTAA
TTAATATACGATCGTCAGGAATTACTTTAGATCGACTACCCTTATTTGTTTGATCTGTAG
TTATTACAGCTATCTTATTACTTCTTTCTCTTCCTGTTTTAGCTGGAGCTATTACTATGT
TATTAACAGACCGAAACTTAAATACATCTTTCTTTGATCCAATCGGAGGAGGAGATCCTA
TT
>KX446411.1
TCGGAACTTCTTTAAGAATTTTAATTCGTGCTGAACTTAGCCACCCTGGTATATTTATTG
GGAATGACCAAATTTATAATGTAATTGTAACAGCTCATGCATTTATTATAATTTTCTTTA
TAGTAATACCAATTATAATTGGAGGATTTGGAAATTGATTAGTTCCTTTAATATTAGGAG
CCCCTGATATAGCTTTTCCTCGAATAAATAATATAAGTTTTTGAATACTACCTCCTTCAT
TGACTCTTCTATTATCAAGCTCAATAGTAGAAAATGGAGCAGGAACTGGGTGAACAGTTT
ATCCTCCTCTCTCTTCAGGAACAGCTCATGCTGGAGCTTCTGTTGATTTAGCTATTTTTT
CTCTTCATTTAGCTGGAATTTCCTCAATTTTAGGGGCAGTAAATTTTATTACAACTGTAA
TTAATATACGATCGTCAGGAATTACTTTAGATCGACTACCCTTATTTGTTTGATCTGTAG
TTATTACAGCTATCTTATTACTTCTTTCTCTTCCTGTTTTAGCTGGAGCTATTACTATGT
TATTAACAGACCGAAACTTAAATACATCTTTCTTTGATCCAATCGGAGGAGGAGATCCTA
TT
>KX446412.1
TCGGAACTTCTCTAAGAATTTTAATTCGTGCTGAACTTAGCCACCCTGGTATATTTATTG
GGAATGACCAAATTTATAATGTAATTGTAACAGCTCATGCATTTATTATAATTTTCTTTA
TAGTAATACCAATTATAATTGGAGGATTTGGAAATTGATTAGTTCCTTTAATATTAGGAG
CCCCTGATATAGCCTTTCCTCGAATAAATAATATAAGTTTTTGAATACTACCTCCTTCAT
TGACTCTTCTATTATCAAGCTCAATAGTAGAAAATGGAGCAGGAACTGGGTGAACAGTTT
ATCCTCCTCTCTCTTCAGGAACAGCTCATGCTGGAGCTTCTGTTGATTTAGCTATTTTTT
CTCTTCATTTAGCTGGAATTTCCTCAATTTTAGGGGCAGTAAATTTTATTACAACTGTAA
TTAATATACGATCATCAGGAATTACTTTAGATCGACTACCCTTATTTGTTTGATCTGTAG
TTATTACAGCTATCTTATTACTTCTTTCTCTTCCTGTTTTAGCTGGAGCTATTACTATGT
TATTAACAGACCGAAACTTAAATACATCTTTCTTTGATCCAATCGGAGGAGGAGATCCTA
TT
>KX446413.1
TCGGAACTTCTCTAAGAATTTTAATTCGTGCTGAACTTAGCCACCCTGGTATATTTATTG
GGAATGACCAAATTTATAATGTAATTGTAACAGCTCATGCATTTATTATAATTTTCTTTA
TAGTAATACCAATTATAATTGGAGGATTTGGAAATTGATTAGTTCCTTTAATATTAGGAG
CCCCTGATATAGCCTTTCCTCGAATAAATAATATAAGTTTTTGAATACTACCTCCTTCAT
TGACTCTTCTATTATCAAGCTCAATAGTAGAAAATGGAGCAGGAACTGGGTGAACAGTTT
ATCCTCCTCTCTCTTCAGGAACAGCTCATGCTGGAGCTTCTGTTGATTTAGCTATTTTTT
CTCTTCATTTAGCTGGAATTTCCTCAATTTTAGGGGCAGTAAATTTTATTACAACTGTAA
TTAATATACGATCGTCAGGAATTACTTTAGATCGACTACCCTTATTTGTTTGATCTGTAG
TTATTACAGCTATCTTATTACTTCTTTCTCTTCCTGTTTTAGCTGGAGCTATTACTATAT
TATTAACAGACCGAAACTTAAATACATCTTTCTTTGATCCAATCGGAGGAGGAGACCCTA
TT
>KX446414.1
---TTTATTG
GGAATGACCAAATTTATAATGTAATTGTAACAGCTCATGCATTTATTATAATTTTCTTTA
TAGTAATACCAATTATAATTGGAGGATTTGGAAATTGATTAGTTCCTTTAATATTAGGAG
CCCCTGATATAGCTTTTCCTCGAATAAATAATATAAGTTTTTGAATACTACCTCCTTCAT
TGACTCTTCTATTATCAAGCTCAATAGTAGAAAATGGGGCAGGAACTGGGTGAACAGTTT
ATCCTCCTCTCTCTTCAGGAACAGCTCATGCTGGAGCTTCTGTTGATTTAGCTATTTTTT
CTCTTCATTTAGCTGGAATTTCCTCAATTTTAGGGGCAGTAAATTTTATTACAACTGTAA
TTAATATACGATCGTCAGGAATTACTTTAGATCGACTACCCTTATTTGTTTGATCTGTAG
TTATTACAGCTATCTTATTACTTCTTTCTCTTCCTGTTTTAGCTGGAGCTATTACTATGT
TATTAACAGACCGAAACTTAAATACATCTTTCTTTGATCCAATCGGAGGAGGAGATCCTA
TT
>KX446415.1
TCGGAACTTCTTTAAGAATTTTAATTCGTGCTGAACTTAGCCACCCTGGTATATTTATTG
GGAATGACCAAATTTATAATGTAATTGTAACAGCTCATGCATTTATTATAATTTTCTTTA
TAGTAATACCAATTATAATTGGAGGATTTGGAAATTGATTAGTTCCTTTAATATTAGGAG

CCCCTGATATAGCTTTTCCTCGAATAAATAATATAAGTTTTTGAATACTACCTCCTTCAT
TGACTCTTCTATTATCAAGCTCAATAGTAGAAAATGGGGCAGGAACTGGGTGAACAGTTT
ATCCTCCTCTCTCTTCAGGAACAGCTCATGCTGGAGCTTCTGTTGATTTAGCTATTTTTT
CTCTTCATTTAGCTGGAATTTCCTCAATTTTAGGGGCAGTAAATTTTATTACAACTGTAA
TTAATATACGATCGTCAGGAATTACTTTAGATCGACTACCCTTATTTGTTTGATCTGTAG
TTATTACAGCTATCTTATTACTTCTTTCTCTTCCTGTTTTAGCTGGAGCTATTACTATGT
TATTAACAGACCGAAACTTAAATACATCTTTCTTTGATCCAATCGGAGGAGGAGACCCTA
TT
>KX446416.1
--ATTG
GGAATGACCAAATTTATAATGTAATTGTAACAGCTCATGCATTTATTATAATTTTCTTTA
TAGTAATACCAATTATAATTGGAGGATTTGGAAATTGATTAGTTCCTTTAATATTAGGAG
CCCCTGATATAGCCTTTCCTCGAATAAATAATATAAGTTTTTGAATACTACCTCCTTCAT
TGACTCTTCTATTATCAAGCTCAATAGTAGAAAATGGAGCAGGAACTGGGTGAACAGTTT
ATCCTCCTCTCTCTTCAGGAACAGCTCATGCTGGAGCTTCTGTTGATTTAGCTATTTTTT
CTCTTCATTTAGCTGGAATTTCCTCAATTTTAGGGGCAGTAAATTTTATTACAACTGTAA
TTAATATACGATCATCAGGAATTACTTTAGATCGACTACCCTTATTTGTTTGATCTGTAG
TTATTACAGCTATCTTATTACTTCTTTCTCTTCCTGTTTTAGCTGGAGCTATTACTATGT
TATTAACAGACCGAAACTTAAATACATCTTTCTTTGATCCAATCGGAGGAGGAGATCCTA
TT
>KX446417.1
TCGGAACTTCTCTAAGAATTTTAATTCGTGCTGAACTTAGCCACCCTGGTATATTTATTG
GGAATGACCAAATTTATAATGTAATTGTAACAGCTCATGCATTTATTATAATTTTCTTTA
TAGTAATACCAATTATAATTGGAGGATTTGGAAATTGATTAGTTCCTTTAATATTAGGAG
CCCCTGATATAGCCTTTCCTCGAATAAATAATATAAGTTTTTGAATACTACCTCCTTCAT
TGACTCTTCTATTATCAAGCTCAATAGTAGAAAATGGAGCAGGAACTGGGTGAACAGTTT
ATCCTCCTCTCTCTTCAGGAACAGCTCATGCTGGAGCTTCTGTTGATTTAGCTATTTTTT
CTCTTCATTTAGCTGGAATTTCCTCAATTTTAGGGGCAGTAAATTTTATTACAACTGTAA
TTAATATACGATCATCAGGAATTACTTTAGATCGACTACCCTTATTTGTTTGATCTGTAG
TTATTACAGCTATCTTATTACTTCTTTCTCTTCCTGTTTTAGCTGGAGCTATTACTATGT
TATTAACAGACCGAAACTTAAATACATCTTTCTTTGATCCAATCGGAGGAGGAGATCCTA
TT
>KX446418.1
TCGGAACTTCTCTAAGAATTTTAATTCGTGCTGAACTTAGCCACCCTGGTATATTTATTG
GAAATGATCAAATTTATAATGTAATTGTAACAGCTCATGCATTTATTATAATTTTCTTTA
TAGTAATACCAATTATAATTGGAGGATTTGGAAATTGATTAGTTCCTTTAATATTAGGAG
CCCCTGATATAGCCTTTCCTCGAATAAATAATATAAGTTTTTGAATACTACCTCCTTCAT
TGACTCTTCTATTATCAAGCTCAATAGTAGAAAATGGGGCAGGAACTGGGTGAACAGTTT
ATCCTCCTCTCTCTTCAGGAACAGCTCATGCTGGAGCTTCTGTTGATTTAGCTATTTTTT
CTCTTCATTTAGCTGGAATTTCCTCAATTTTAGGGGCAGTAAATTTTATTACAACTGTAA
TTAATATACGATCGTCAGGAATTACTTTAGATCGACTACCCTTATTTGTTTGATCTGTAG
TTATTACAGCTATCTTATTACTTCTTTCTCTTCCTGTTTTAGCTGGAGCTATTACTATGT
TATTAACAGACCGAAACTTAAATACATCTTTCTTTGACCCAATTGGAGGAGGAGATCCTA
TT
>KX446419.1
TCGGAACTTCTCTAAGAATTTTAATTCGTGCTGAACTTAGCCACCCTGGTATATTTATTG
GAAATGATCAAATTTATAATGTAATTGTAACAGCTCATGCATTTATTATAATTTTCTTTA
TAGTAATACCAATTATAATTGGAGGATTTGGAAATTGATTAGTTCCTTTAATATTAGGAG
CCCCTGATATAGCCTTTCCTCGAATAAATAATATAAGTTTTTGAATACTACCTCCTTCAT
TGACTCTTCTATTATCAAGCTCAATAGTAGAAAATGGGGCAGGAACTGGGTGAACAGTTT
ATCCTCCTCTCTCTTCAGGAACAGCTCATGCTGGAGCTTCTGTTGATTTAGCTATTTTTT
CTCTTCATTTAGCTGGAATTTCCTCAATTTTAGGGGCAGTAAATTTTATTACAACTGTAA
TTAATATACGATCGTCAGGAATTACTTTAGATCGACTACCCTTATTTGTTTGATCTGTAG
TTATTACAGCTATCTTATTACTTCTTTCTCTTCCTGTTTTAGCTGGAGCTATTACTATGT
TATTAACAGACCGAAACTTAA---------------------------------------
--
>KX446420.1

TCGGAACTTCTCTAAGAATTTTAATTCGTGCTGAACTTAGCCACCCTGGTATATTTATTG
GGAATGACCAAATTTATAATGTAATTGTAACAGCTCATGCATTTATTATAATTTTCTTTA
TAGTAATACCAATTATAATTGGAGGATTTGGAAATTGATTAGTTCCTTTAATATTAGGAG
CCCCTGATATAGCTTTTCCTCGAATAAATAATATAAGTTTTTGAATACTACCTCCTTCAT
TGACTCTTCTATTATCAAGCTCAATAGTAGAAAATGGAGCAGGAACTGGGTGAACAGTTT
ATCCTCCTCTCTCTTCAGGAACAGCTCATGCTGGAGCTTCTGTTGATTTAGCTATTTTTT
CTCTTCATTTAGCTGGAATTTCCTCAATTTTAGGGGCAGTAAATTTTATTACAACTGTAA
TTAATATACGATCGTCAGGAATTACTTTAGATCGACTACCCTTATTTGTTTGATCTGTAG
TTATTACAGCTATCTTATTACTTCTTTCTCTTCCTGTTTTAGCTGGAGCTATTACTATGT
TATTAACAGACCGAAACTTAAATACATCTTTCTTTGATCCAATCGGAGGAGGAGATCCTA
TT
>KX446421.1
TCGGAACTTCTCTAAGAATTTTAATTCGTGCTGAACTTAGCCACCCTGGTATATTTATTG
GGAATGACCAAATTTATAATGTAATTGTAACAGCTCATGCATTTATTATAATTTTCTTTA
TAGTAATACCAATTATAATTGGAGGATTTGGAAATTGATTAGTTCCTTTAATATTAGGAG
CCCCTGATATAGCCTTTCCTCGAATAAATAATATAAGTTTTTGAATACTACCTCCTTCAT
TGACTCTTCTATTATCAAGCTCAATAGTAGAAAATGGAGCAGGAACTGGGTGAACAGTTT
ATCCTCCTCTCTCTTCAGGAACAGCTCATGCTGGAGCTTCTGTTGATTTAGCTATTTTTT
CTCTTCATTTAGCTGGAATTTCCTCAATTTTAGGAGCAGTAAATTTTATTACAACTGTAA
TTAATATACGATCGTCAGGAATTACTTTAGATCGACTACCCTTATTTGTTTGATCTGTAG
TTATTACAGCTATCTTATTACTTCTTTCTCTTCCTGTTTTAGCTGGAGCTATTACTATGT
TATTAACAGACCGAAACTTAA---------------------------------------
--
>KX446422.1
TCGGAACTTCTTTAAGAATTTTAATTCGTGCTGAACTTAGCCACCCTGGTATATTTATTG
GGAATGACCAAATTTATAATGTAATTGTAACAGCTCATGCATTTATTATAATTTTCTTTA
TAGTAATACCAATTATAATTGGAGGATTTGGAAATTGATTAGTTCCTTTAATATTAGGAG
CCCCTGATATAGCTTTTCCTCGAATAAATAATATAAGTTTTTGAATACTACCTCCTTCAT
TGACTCTTCTATTATCAAGCTCAATAGTAGAAAATGGGGCAGGAACTGGGTGAACAGTTT
ATCCTCCTCTCTCTTCAGGAACAGCTCATGCTGGAGCTTCTGTTGATTTAGCTATTTTTT
CTCTTCATTTAGCTGGAATTTCCTCAATTTTAGGGGCAGTAAATTTTATTACAACTGTAA
TTAATATACGATCGTCAGGAATTACTTTAGATCGACTACCCTTATTTGTTTGATCTGTAG
TTATTACAGCTATCTTATTACTTCTTTCTCTTCCTGTTTTAGCTGGAGCTATTACTATGT
TATTAACAGACCGAAACTTAA---------------------------------------
--
>KX446423.1
TCGGAACTTCTTTAAGAATTTTAATTCGTGCTGAACTTAGCCACCCTGGTATATTTATTG
GGAATGACCAAATTTATAATGTAATTGTAACAGCTCATGCATTTATTATAATTTTCTTTA
TAGTAATACCAATTATAATTGGAGGATTTGGAAATTGATTAGTTCCTTTAATATTAGGAG
CCCCTGATATAGCTTTTCCTCGAATAAATAATATAAGTTTTTGAATACTACCTCCTTCAT
TGACTCTTCTATTATCAAGCTCAATAGTAGAAAATGGGGCAGGAACTGGGTGAACAGTTT
ATCCTCCTCTCTCTTCAGGAACAGCTCATGCTGGAGCTTCTGTTGATTTAGCTATTTTTT
CTCTTCATTTAGCTGGAATTTCCTCAATTTTAGGGGCAGTAAATTTTATTACAACTGTAA
TTAATATACGATCGTCAGGAATTACTTTAGATCGACTACCCTTATTTGTTTGATCTGTAG
TTATTACAGCTATCTTATTACTTCTTTCTCTTCCTGTTTTAGCTGGAGCTATTACTATGT
TATTAACAGACCGAAACTTAAATACATCTTTCTTTGATCCAATCGGAGGAGGAGATCCTA
TT
>KX446424.1
TCGGAACTTCTCTAAGAATTTTAATTCGTGCTGAACTTAGCCACCCTGGTATATTTATTG
GGAATGACCAAATTTATAATGTAATTGTAACAGCTCATGCATTTATTATAATTTTCTTTA
TAGTAATACCAATTATAATTGGAGGATTTGGAAATTGATTAGTTCCTTTAATATTAGGAG
CCCCTGATATAGCTTTTCCTCGAATAAATAATATAAGTTTTTGAATACTACCTCCTTCAT
TGACTCTTCTATTATCAAGCTCAATAGTAGAAAATGGAGCAGGAACTGGGTGAACAGTTT
ATCCTCCTCTCTCTTCAGGAACAGCTCATGCTGGAGCTTCTGTTGATTTAGCTATTTTTT
CTCTTCATTTAGCTGGAATTTCCTCAATTTTAGGGGCAGTAAATTTTATTACAACTGTAA
TTAATATACGATCGTCAGGAATTACTTTAGATCGACTACCCTTATTTGTTTGATCTGTAG
TTATTACAGCTATCTTATTACTTCTTTCTCTTCCTGTTTTAGCTGGAGCTATTACTATAT

TATTAACAGACCGAAACTTAAATACATCTTTCTTTGACCCAATCGGAGGAGGAGATCCTA
TT
>KX446425.1
TCGGAACTTCTCTAAGAATTTTAATTCGTGCTGAACTTAGCCACCCTGGTATATTTATTG
GGAATGACCAAATTTATAATGTAATTGTAACAGCTCATGCATTTATTATAATTTTCTTTA
TAGTAATGCCAATTATAATTGGAGGATTTGGAAATTGATTAGTTCCTTTAATATTAGGAG
CCCCTGATATAGCTTTCCCTCGAATGAATAATATAAGTTTTTGAATACTACCTCCTTCAT
TGACTCTTCTATTATCAAGCTCAATAGTAGAAAATGGAGCAGGAACTGGGTGAACAGTTT
ATCCCCCTCTCTCTTCAGGAACAGCTCATGCTGGAGCTTCTGTTGATTTAGCTATTTTTT
CTCTTCATTTAGCTGGAATTTCCTCAATTTTAGGGGCAGTAAATTTTATTACAACTGTAA
TTAATATACGATCGTCAGGGATTACTTTAGATCGACTACCCTTATTTGTTTGATCTGTAG
TTATTACAGCTATCTTATTACTTCTTTCTCTTCCTGTTTTAGCTGGAGCTATTACTATAT
TATTAACAGACCGAAACTTAAATACATCTTTCTTTGATCCAATCGGAGGGGGAGACCCTA
TT
>KX446426.1
TCGGAACTTCTCTAAGAATTTTAATTCGTGCTGAACTTAGCCACCCTGGTATATTTATTG
GGAATGACCAAATTTATAATGTAATTGTAACAGCTCATGCATTTATTATAATTTTCTTTA
TAGTAATACCAATTATAATTGGAGGATTTGGAAATTGATTAGTTCCTTTAATATTAGGAG
CCCCTGATATAGCTTTCCCTCGAATAAATAATATAAGTTTTTGAATACTACCTCCTTCAT
TGACTCTTCTATTATCAAGCTCAATAGTAGAAAATGGGGCAGGAACTGGGTGAACAGTTT
ATCCTCCTCTCTCTTCAGGAACAGCTCATGCTGGAGCTTCTGTTGATTTAGCTATTTTTT
CTCTTCATTTAGCTGGAATTTCCTCAATTTTAGGGGCAGTAAATTTTATTACAACTGTAA
TTAATATACGATCGTCAGGGATTACTTTAGATCGACTACCCTTATTTGTTTGATCTGTAG
TTATTACAGCTATCTTATTACTTCTTTCTCTTCCTGTTTTAGCTGGAGCTATTACTATAT
TATTAACAGACCGAAACTTAAATACATCTTTCTTTGATCCAATCGGAGGAGGAGACCCTA
TT
>KX446427.1
TCGGAACTTCTCTAAGAATTTTAATTCGTGCTGAACTTAGCCACCCTGGTATATTTATTG
GGAATGACCAAATTTATAATGTAATTGTAACAGCTCATGCATTTATTATAATTTTCTTTA
TAGTAATACCAATTATAATTGGAGGATTTGGAAATTGATTAGTTCCTTTAATATTAGGAG
CCCCTGATATAGCCTTTCCTCGAATAAATAATATAAGTTTTTGAATACTACCTCCTTCAT
TGACTCTTCTATTATCAAGCTCAATAGTAGAAAATGGAGCAGGAACTGGGTGAACAGTTT
ATCCTCCTCTCTCTTCAGGAACAGCTCATGCTGGAGCTTCTGTTGATTTAGCTATTTTTT
CTCTTCATTTAGCTGGAATTTCCTCAATTTTAGGGGCAGTAAATTTTATTACAACTGTAA
TTAATATACGATCGTCAGGAATTACTTTAGATCGACTACCCTTATTTGTTTGATCTGTAG
TTATTACAGCTATCTTATTACTTCTTTCTCTTCCTGTTTTAGCTGGAGCTATTACTATAT
TATTAACAGACCGAAACTTAAATACATCTTTCTTTGATCCAATCGGAGGAGGAGACCCTA
TT
>KX446428.1
TCGGAACTTCTTTAAGAATTTTAATTCGTGCTGAACTTAGCCACCCTGGTATATTTATTG
GGAATGACCAAATTTATAATGTAATTGTAACAGCTCATGCATTTATTATAATTTTCTTTA
TAGTAATACCAATTATAATTGGAGGATTTGGAAATTGATTAGTTCCTTTAATATTAGGAG
CCCCTGATATAGCTTTTCCTCGAATAAATAATATAAGTTTTTGAATACTACCTCCTTCAT
TGACTCTTCTATTATCAAGCTCAATAGTAGAAAATGGAGCAGGAACTGGGTGAACAGTTT
ATCCTCCTCTCTCTTCAGGAACAGCTCATGCTGGAGCTTCTGTTGATTTAGCTATTTTTT
CTCTTCATTTAGCTGGAATTTCCTCAATTTTAGGGGCAGTAAATTTTATTACAACTGTAA
TTAATATACGATCGTCAGGAATTACTTTAGATCGACTACCCTTATTTGTTTGATCTGTAG
TTATTACAGCTATCTTATTACTTCTTTCTCTTCCTGTTTTAGCTGGAGCTATTACTATGT
TATTAACAGACCGAAACTTAAATACATCTTTCTTTGATCCAATCGGAGGAGGAGATCCTA
TT
>KX446429.1
TCGGAACTTCTTTAAGAATTTTAATTCGTGCTGAACTTAGCCACCCTGGTATATTTATTG
GGAATGATCAAATTTATAATGTAATTGTAACAGCTCATGCATTTATTATAATTTTCTTTA
TAGTAATACCAATTATAATTGGAGGATTTGGAAATTGATTAGTTCCTTTAATATTAGGAG
CCCCTGATATAGCTTTTCCTCGAATAAATAATATAAGTTTTTGAATACTACCTCCTTCAT
TGACTCTTCTATTATCAAGCTCAATAGTAGAAAATGGGGCAGGAACTGGGTGAACAGTTT
ATCCTCCTCTCTCTTCAGGAACAGCTCATGCTGGAGCTTCTGTTGATTTAGCTATTTTTT

CTCTTCATTTAGCTGGAATTTCCTCAATTTTAGGGGCAGTAAATTTTATTACAACTGTAA
TTAATATACGATCGTCAGGAATTACTTTAGATCGACTACCCTTATTTGTTTGATCTGTAG
TTATTACAGCTATCTTATTACTTCTTTCTCTTCCTGTTTTAGCTGGAGCTATTACTATGT
TATTAACAGACCGAAACTTAAATTCGTCTTTCTTTGATCCAATCGGAGGAGGAGATCCTA
TT
>KX446430.1
TCGGAACTTCTCTAAGAATTTTAATTCGTGCTGAACTTAGCCACCCTGGTATATTTATTG
GGAATGACCAAATTTATAATGTAATTGTAACAGCTCATGCATTTATTATAATTTTCTTTA
TAGTAATGCCAATTATAATTGGAGGATTTGGAAATTGATTAGTTCCTTTAATATTAGGAG
CCCCTGATATAGCTTTCCCTCGAATGAATAATATAAGTTTTTGAATACTACCTCCTTCAT
TGACTCTTCTATTATCAAGCTCAATAGTAGAAAATGGAGCAGGAACTGGGTGAACAGTTT
ATCCTCCTCTCTCTTCAGGAACAGCTCATGCTGGAGCTTCTGTTGATTTAGCTATTTTTT
CTCTTCATTTAGCTGGAATTTCCTCAATTTTAGGGGCAGTAAATTTTATTACAACTGTAA
TTAATATACGATCGTCAGGGATTACTTTAGATCGACTACCCTTATTTGTTTGATCTGTAG
TTATTACAGCTATCTTATTACTTCTTTCTCTTCCTGTTTTAGCCGGAGCTATTACTATAT
TATTAACAGACCGAAACTTAAATACATCTTTCTTTGATCCAATCGGAGGAGGAGACCCTA
TT
>KX446431.1
TAGGAACTTCTCTAAGAATTTTAATTCGTGCTGAACTTAGCCACCCTGGTATATTTATTG
GGAATGACCAAATTTATAATGTAATTGTAACAGCTCATGCATTTATTATAATTTTCTTTA
TAGTAATGCCAATTATAATTGGAGGATTTGGAAATTGATTAGTTCCTTTAATATTAGGAG
CCCCTGATATAGCTTTCCCTCGAATGAATAATATAAGTTTTTGAATACTACCTCCTTCAT
TGACTCTTCTATTATCAAGCTCAATAGTAGAAAATGGAGCAGGAACTGGGTGAACAGTTT
ATCCTCCTCTCTCTTCAGGAACAGCTCATGCTGGGGCTTCTGTTGATTTAGCTATTTTTT
CTCTTCATTTAGCTGGAATTTCCTCAATTTTAGGGGCAGTAAATTTTATTACAACTGTAA
TTAATATACGATCGTCAGGGATTACTTTAGATCGACTACCCTTATTTGTTTGATCTGTAG
TTATTACAGCTATCTTATTACTTCTTTCTCTTCCTGTTTTAGCTGGAGCTATTACTATAT
TATTAACAGACCGAAACTTAAATACATCTTTCTTTGATCCAATCGGAGGGGGAGATCCTA
TT
>KX446432.1
------------TAAGAATTTTAATTCGTGCTGAACTTAGCCACCCTGGTATATTTATTG
GGAATGACCAAATTTATAATGTAATTGTAACAGCTCATGCATTTATTATAATTTTCTTTA
TAGTAATACCAATTATAATTGGAGGATTTGGAAATTGATTAGTTCCTTTAATATTAGGAG
CCCCTGATATAGCTTTCCCTCGAATAAATAATATAAGTTTTTGAATACTACCTCCTTCAT
TGACTCTTCTATTATCAAGCTCAATAGTAGAAAATGGGGCAGGAACTGGGTGAACAGTTT
ATCCTCCTCTCTCTTCAGGAACAGCTCATGCTGGAGCTTCTGTTGATTTAGCTATTTTTT
CTCTTCATTTAGCTGGAATTTCCTCAATTTTAGGGGCAGTAAATTTTATTACAACTGTAA
TTAATATACGATCGTCAGGGATTACTTTAGATCGACTACCCTTATTTGTTTGATCTGTAG
TTATTACAGCTATCTTATTACTTCTTTCTCTTCCTGTTTTAGCTGGAGCTATTACTATAT
TATTAACAGACCGAAACTTAAATACATCTTTCTTTGATCCAATCGGAGGAGGAGACCCTA
TT
>KX446433.1
TCGGAACTTCTTTAAGAATTTTAATTCGTGCTGAACTTAGCCACCCTGGTATATTTATTG
GGAATGACCAAATTTATAATGTAATTGTAACAGCTCATGCATTTATTATAATTTTCTTTA
TAGTAATACCAATTATAATTGGAGGATTTGGAAATTGATTAGTTCCTTTAATATTAGGAG
CCCCTGATATAGCTTTTCCTCGAATAAATAATATAAGTTTTTGAATACTACCTCCTTCAT
TGACTCTTCTATTATCAAGCTCAATAGTAGAAAATGGGGCAGGAACTGGGTGAACAGTTT
ATCCTCCTCTCTCTTCAGGAACAGCTCATGCTGGAGCTTCTGTTGATTTAGCTATTTTTT
CTCTTCATTTAGCTGGAATTTCCTCAATTTTAGGGGCAGTAAATTTTATTACAACTGTAA
TTAATATACGATCGTCAGGAATTACTTTAGATCGACTACCCTTATTTGTTTGATCTGTAG
TTATTACAGCTATCTTATTACTTCTTTCTCTTCCTGTTTTAGCTGGAGCTATTACTATGT
TATTAACAGACCGAAACTTAAATACATCTTTCTTTGATCCAATCGGAGGAGGAGATCCTA
TT
>KX446434.1
TCGGAACTTCTTTAAGAATTTTAATTCGTGCTGAACTTAGCCACCCTGGTATATTTATTG
GGAATGACCAAATTTATAATGTAATTGTAACAGCTCATGCATTTATTATAATTTTCTTTA
TAGTAATACCAATTATAATTGGAGGATTTGGAAATTGATTAGTTCCTTTAATATTAGGAG

CCCCTGATATAGCTTTTCCTCGAATAAATAATATAAGTTTTTGAATACTACCTCCTTCAT
TGACTCTTCTATTATCAAGCTCAATAGTAGAAAATGGGGCAGGAACTGGGTGAACAGTTT
ATCCTCCTCTCTCTTCAGGAACAGCTCATGCTGGAGCTTCTGTTGATTTAGCTATTTTTT
CTCTTCATTTAGCTGGAATTTCCTCAATTTTAGGGGCAGTAAATTTTATTACAACTGTAA
TTAATATACGATCGTCAGGAATTACTTTAGATCGACTACCCTTATTTGTTTGATCTGTAG
TTATTACAGCTATCTTATTACTTCTTTCTCTTCCTGTTTTAGCTGGAGCTATTACTATGT
TATTAACAGACCGAAACTTAAATACATCTTTCTTTGATCCAATCGGAGGAGGAGATCCTA
TT
>KX446435.1
TCGGAACTTCTTTAAGAATTTTAATTCGTGCTGAACTTAGCCACCCTGGTATATTTATTG
GGAATGACCAAATTTATAATGTAATTGTAACAGCTCATGCATTTATTATAATTTTCTTTA
TAGTAATACCAATTATAATTGGAGGATTTGGAAATTGATTAGTTCCTTTAATATTAGGAG
CCCCTGATATAGCTTTTCCTCGAATAAATAATATAAGTTTTTGAATACTACCTCCTTCAT
TGACTCTTCTATTATCAAGCTCAATAGTAGAAAATGGGGCAGGAACTGGGTGAACAGTTT
ATCCTCCTCTCTCTTCAGGAACAGCTCATGCTGGAGCTTCTGTTGATTTAGCTATTTTTT
CTCTTCATTTAGCTGGAATTTCCTCAATTTTAGGGGCAGTAAATTTTATTACAACTGTAA
TTAATATACGATCGTCAGGAATTACTTTAGATCGACTACCCTTATTTGTTTGATCTGTAG
TTATTACAGCTATCTTATTACTTCTTTCTCTTCCTGTTTTAGCTGGAGCTATTACTATGT
TATTAACAGACCGAAACTTAAATACATCTTTCTTTGATCCAATCGGAGGAGGAGATCCTA
TT
>KX446436.1
TCGGAACTTCTCTAAGAATTTTAATTCGTGCTGAACTTAGCCACCCTGGTATATTTATTG
GAAATGACCAAATTTATAATGTAATTGTAACAGCTCATGCATTTATTATAATTTTCTTTA
TAGTAATGCCAATTATAATTGGAGGATTTGGAAATTGATTAGTTCCTTTAATATTAGGAG
CCCCTGATATAGCTTTCCCTCGAATAAATAATATAAGTTTTTGAATACTACCTCCTTCAT
TGACTCTTCTATTATCAAGCTCAATAGTAGAAAATGGAGCAGGAACTGGGTGAACAGTTT
ATCCTCCTCTCTCTTCAGGAACAGCTCATGCTGGAGCTTCTGTTGATTTAGCTATTTTTT
CTCTTCATTTAGCTGGAATTTCCTCAATTTTAGGGGCAGTAAATTTTATTACAACTGTAA
TTAATATACGATCGTCAGGGATTACTTTAGATCGACTACCTTTATTTGTTTGATCTGTAG
TTATTACAGCTATCTTATTACTTCTTTCTCTTCCTGTTTTAGCTGGAGCTATTACTATAT
TATTAACAGACCGAAATTTAAATACATCTTTCTTTGATCCAATTGGAGGGGGAGACCCTA
TT
>KX446437.1
TTGGAACTTCTCTAAGAATTTTAATTCGTGCTGAACTTAGCCACCCTGGTATATTTATTG
GGAATGACCAAATTTATAATGTAATTGTAACAGCTCATGCATTTATTATAATTTTCTTTA
TAGTAATACCAATTATAATTGGAGGATTTGGAAATTGATTAGTTCCTTTAATATTAGGAG
CCCCTGATATAGCCTTTCCTCGAATAAATAATATAAGTTTTTGAATACTACCTCCTTCAT
TGACTCTTCTATTATCAAGCTCAATAGTAGAAAATGGAGCAGGAACTGGGTGAACAGTTT
ATCCTCCTCTCTCTTCAGGAACAGCTCATGCTGGAGCTTCTGTTGATTTAGCTATTTTTT
CTCTTCATTTAGCTGGAATTTCCTCAATTTTAGGGGCAGTAAATTTTATTACAACTGTAA
TTAATATACGATCGTCAGGAATTACTTTAGATCGACTACCCTTATTTGTTTGATCTGTAG
TTATTACAGCTATCTTACTACTTCTTTCTCTTCCTGTTTTAGCTGGAGCTATTACTATGT
TATTAACAGACCGAAACTTAAATACATCTTTCTTTGACCCAATCGGAGGAGGAGATCCTA
TT
>KX446438.1
TCGGAACTTCTCTAAGAATTTTAATTCGTGCTGAACTTAGCCACCCTGGTATATTTATTG
GGAATGACCAAATTTATAATGTAATTGTAACAGCTCATGCATTTATTATAATTTTCTTTA
TAGTGATACCAATTATAATTGGAGGATTTGGAAATTGATTAGTTCCTTTAATATTAGGAG
CCCCTGATATAGCCTTTCCTCGAATAAATAATATAAGTTTTTGAATACTACCTCCTTCAT
TGACTCTTCTATTATCAAGCTCAATAGTAGAAAATGGAGCAGGAACTGGGTGAACAGTTT
ATCCTCCTCTCTCTTCAGGAACAGCTCATGCTGGAGCTTCTGTTGATTTAGCTATTTTTT
CTCTTCATTTAGCTGGAATTTCCTCAATTTTAGGGGCAGTAAATTTTATTACAACTGTAA
TTAATATACGATCGTCAGGAATTACTTTAGATCGACTACCCTTATTTGTTTGATCTGTAG
TTATTACAGCTATCTTATTACTTCTTTCTCTTCCTGTTTTAGCTGGAGCTATTACTATGT
TATTAACAGACCGAAACTTAAATACATCTTTCTTTGATCCAATCGGAGGAGGAGATCCTA
TT
>KX446439.1

TCGGAACTTCTCTAAGAATTTTAATTCGTGCTGAACTTAGCCACCCTGGTATATTTATTG
GGAATGACCAAATTTATAATGTAATTGTAACAGCTCATGCATTTATTATAATTTTCTTTA
TAGTGATACCAATTATAATTGGAGGATTTGGAAATTGATTAGTTCCTTTAATATTAGGAG
CCCCTGATATAGCCTTTCCTCGAATAAATAATATAAGTTTTTGAATACTACCTCCTTCAT
TGACTCTTCTATTATCAAGCTCAATAGTAGAAAATGGAGCAGGAACTGGGTGAACAGTTT
ATCCTCCTCTCTCTTCAGGAACAGCTCATGCTGGAGCTTCTGTTGATTTAGCTATTTTTT
CTCTTCATTTAGCTGGAATTTCCTCAATTTTAGGGGCAGTAAATTTTATTACAACTGTAA
TTAATATACGATCGTCAGGAATTACTTTAGATCGACTACCCTTATTTGTTTGATCTGTAG
TTATTACAGCTATCTTATTACTTCTTTCTCTTCCTGTTTTAGCTGGAGCTATTACTATGT
TATTAACAGACCGAAACTTAAATACATCTTTCTTTGATCCAATCGGAGGAGGAGATCCTA
TT
>KX446440.1
TCGGAACTTCTTTAAGAATTTTAATTCGTGCTGAACTTAGCCACCCTGGTATATTTATTG
GGAATGACCAAATTTATAATGTAATTGTAACAGCTCATGCATTTATTATAATTTTCTTTA
TAGTAATACCAATTATAATTGGAGGATTTGGAAATTGATTAGTTCCTTTAATATTAGGAG
CCCCTGATATAGCTTTTCCTCGAATAAATAATATAAGTTTTTGAATACTACCTCCTTCAT
TGACTCTTCTATTATCAAGCTCAATAGTAGAAAATGGGGCAGGAACTGGGTGAACAGTTT
ATCCTCCTCTCTCTTCAGGAACAGCTCATGCTGGAGCTTCTGTTGATTTAGCTATTTTTT
CTCTTCATTTAGCTGGAATTTCCTCAATTTTAGGGGCAGTAAATTTTATTACAACTGTAA
TTAATATACGATCGTCAGGAATTACTTTAGATCGACTACCCTTATTTGTTTGATCTGTAG
TTATTACAGCTATCTTATTACTTCTTTCTCTTCCTGTTTTAGCTGGAGCTATTACTATGT
TATTAACAGACCGAAACTTAAATACATCTTTCTTTGATCCAATCGGAGGAGGAGATCCTA
TT
>KX446441.1
TTGGAACTTCTCTAAGAATTTTAATTCGTGCTGAACTTAGCCACCCTGGTATATTTATTG
GGAATGACCAAATTTATAATGTAATTGTAACAGCTCATGCATTTATTATAATTTTCTTTA
TAGTAATACCAATTATAATTGGAGGATTTGGAAATTGATTAGTTCCTTTAATATTAGGAG
CCCCTGATATAGCCTTTCCTCGAATAAATAATATAAGTTTTTGAATACTACCTCCTTCAT
TGACTCTTCTATTATCAAGCTCAATAGTAGAAAATGGAGCAGGAACTGGGTGAACAGTTT
ATCCTCCTCTCTCTTCAGGAACAGCTCATGCTGGAGCTTCTGTTGATTTAGCTATTTTTT
CTCTTCATTTAGCTGGAATTTCCTCAATTTTAGGGGCAGTAAATTTTATTACAACTGTAA
TTAATATACGATCGTCAGGAATTACTTTAGATCGACTACCCTTATTTGTTTGATCTGTAG
TTATTACAGCTATCTTACTACTTCTTTCTCTTCCTGTTTTAGCTGGAGCTATTACTATGT
TATTAACAGACCGAAACTTAAATACATCTTTCTTTGACCCAATCGGAGGAGGAGATCCTA
TT
>KX446442.1
TCGGAACTTCTTTAAGAATTTTAATTCGTGCTGAACTTAGCCACCCTGGTATATTTATTG
GGAATGACCAAATTTATAATGTAATTGTAACAGCTCATGCATTTATTATAATTTTCTTTA
TAGTAATACCAATTATAATTGGAGGATTTGGAAATTGATTAGTTCCTTTAATATTAGGAG
CCCCTGATATAGCTTTTCCTCGAATAAATAATATAAGTTTTTGAATACTACCCCCTTCAT
TGACTCTTCTATTATCAAGCTCAATAGTAGAAAATGGGGCAGGAACTGGGTGAACAGTTT
ATCCTCCTCTCTCTTCAGGAACAGCTCATGCTGGAGCTTCTGTTGATTTAGCTATTTTTT
CTCTTCATTTAGCTGGAATTTCCTCAATTTTAGGGGCAGTAAATTTTATTACAACTGTAA
TTAATATACGATCGTCAGGAATTACTTTAGATCGACTACCCTTATTTGTTTGATCTGTAG
TTATTACAGCTATCTTATTACTTCTTTCTCTTCCTGTTTTAGCTGGAGCTATTACTATGT
TATTAACAGACCGAAACTTAAATACATCTTTCTTTGATCCAATCGGAGGAGGAGATCCTA
TT
>KX446443.1
TCGGAACTTCTCTAAGAATTTTAATTCGTGCTGAACTTAGCCACCCTGGTATATTTATTG
GGAATGACCAAATTTATAATGTAATTGTAACAGCTCATGCATTTATTATAATTTTCTTTA
TAGTAATACCAATTATAATTGGAGGATTTGGAAATTGATTAGTTCCTTTAATATTAGGAG
CCCCTGATATAGCCTTTCCTCGAATAAATAATATAAGTTTTTGAATACTACCTCCTTCAT
TGACTCTTCTATTATCAAGCTCAATAGTAGAAAATGGAGCAGGAACTGGGTGAACAGTTT
ATCCTCCTCTCTCTTCAGGAACAGCTCATGCTGGAGCTTCTGTTGATTTAGCTATTTTTT
CTCTTCATTTAGCTGGAATTTCCTCAATTTTAGGGGCAGTAAATTTTATTACAACTGTAA
TTAATATACGATCGTCAGGAATTACTTTAGATCGACTACCCTTATTTGTTTGATCTGTAG
TTATTACAGCTATCTTATTACTTCTTTCTCTTCCTGTTTTAGCTGGAGCTATTACTATGT

TATTAACAGACCGAAACTTAAATACATCTTTCTTTGATCCAATCGGAGGAGGAGATCCTA
TT
>KX446444.1
TCGGAACTTCTCTAAGAATTTTAATTCGTGCTGAACTTAGCCACCCTGGTATATTTATTG
GAAATGATCAAATTTATAATGTAATTGTAACAGCTCATGCATTTATTATAATTTTCTTTA
TAGTAATACCAATTATAATTGGAGGATTTGGAAATTGATTAGTTCCTTTAATATTAGGAG
CCCCTGATATAGCCTTTCCTCGAATAAATAATATAAGTTTTTGAATACTACCTCCTTCAT
TGACTCTTCTATTATCAAGCTCAATAGTAGAAAATGGAGCAGGAACTGGGTGAACAGTTT
ATCCTCCTCTCTCTTCAGGAACAGCTCATGCTGGAGCTTCTGTTGATTTAGCTATTTTTT
CTCTTCATTTAGCTGGAATTTCCTCAATTTTAGGGGCCGTAAATTTTATTACAACTGTAA
TTAATATACGATCGTCAGGAATTACTTTAGATCGACTACCCTTATTTGTTTGATCTGTAG
TTATTACAGCTATCTTATTACTTCTTTCTCTTCCTGTTTTAGCTGGAGCTATTACTATGT
TATTAACAGACCGAAACTTAAATACATCTTTCTTTGATCCAATTGGAGGAGGAGATCCTA
TT
>KX446445.1
TCGGAACTTCTTTAAGAATTTTAATTCGTGCTGAACTTAGCCACCCTGGTATATTTATTG
GGAATGACCAAATTTATAATGTAATTGTAACAGCTCATGCATTTATTATAATTTTCTTTA
TAGTAATACCAATTATAATTGGAGGATTTGGAAATTGATTAGTTCCTTTAATATTAGGAG
CCCCTGATATAGCTTTTCCTCGAATAAATAATATAAGTTTTTGAATACTACCTCCTTCAT
TGACTCTTCTATTATCAAGCTCAATAGTAGAAAATGGAGCAGGAACTGGGTGAACAGTTT
ATCCTCCTCTCTCTTCAGGAACAGCTCATGCTGGAGCTTCTGTTGATTTAGCTATTTTTT
CTCTTCATTTAGCTGGAATTTCCTCAATTTTAGGGGCAGTAAATTTTATTACAACTGTAA
TTAATATACGATCGTCAGGAATTACTTTAGATCGACTACCCTTATTTGTTTGATCTGTAG
TTATTACAGCTATCTTATTACTTCTTTCTCTTCCTGTTTTAGCTGGAGCTATTACTATGT
TATTAACAGACCGAAACTTAAATACATCTTTCTTTGATCCAATCGGAGGAGGAGATCCTA
TT
>KX446446.1
TCGGAACTTCTCTAAGAATTTTAATTCGTGCTGAACTTAGCCACCCTGGTATATTTATTG
GGAATGACCAAATTTATAATGTAATTGTAACAGCTCATGCATTTATTATAATTTTCTTTA
TAGTAATACCAATTATAATTGGAGGATTTGGAAATTGATTAGTTCCTTTAATATTAGGAG
CCCCTGATATAGCCTTTCCTCGAATAAATAATATAAGTTTTTGAATACTACCTCCTTCAT
TGACTCTTCTATTATCAAGCTCAATAGTAGAAAATGGAGCAGGAACTGGGTGAACAGTTT
ATCCTCCTCTCTCTTCAGGAACAGCTCATGCTGGAGCTTCTGTTGATTTAGCTATTTTTT
CTCTTCATTTAGCTGGAATTTCCTCAATTTTAGGGGCAGTAAATTTTATTACAACTGTAA
TTAATATACGATCGTCAGGAATTACTTTAGATCGACTACCCTTATTTGTTTGATCTGTAG
TTATTACAGCTATCTTATTACTTCTTTCTCTTCCTGTTTTAGCTGGAGCTATTACTATGT
TATTAACAGACCGAAACTTAAATACATCTTTCTTTGATCCAATCGGAGGAGGAGATCCTA
TT
>KX446447.1
TCGGAACTTCTCTAAGAATTTTAATTCGTGCTGAACTTAGCCACCCTGGTATATTTATTG
GGAATGACCAAATTTATAATGTAATTGTAACAGCTCATGCATTTATTATAATTTTCTTTA
TAGTAATACCAATTATAATTGGAGGATTTGGAAATTGATTAGTTCCTTTAATATTAGGAG
CCCCTGATATAGCTTTCCCTCGAATGAATAATATAAGTTTTTGAATACTACCTCCTTCAT
TGACTCTTCTATTATCAAGCTCAATAGTAGAAAATGGGGCAGGAACTGGATGAACAGTTT
ATCCTCCTCTCTCTTCAGGAACAGCTCATGCTGGAGCTTCTGTTGATTTAGCTATTTTTT
CTCTTCATTTAGCTGGAATTTCCTCAATTTTAGGGGCAGTAAATTTTATTACAACTGTAA
TTAATATACGATCGTCAGGAATTACTTTAGATCGACTACCCTTATTTGTTTGATCTGTAG
TTATTACAGCTATCTTATTACTTCTTTCTCTTCCTGTTTTAGCTGGAGCTATTACTATAT
TATTAACAGACCGAAACTTAAATACATCTTTCTTTGATCCAATCGGAGGGGGAGACCCTA
TT
>KX446448.1
TCGGAACTTCTCTAAGAATTTTAATTCGTGCTGAACTTAGCCACCCTGGTATATTTATTG
GGAATGACCAAATTTATAATGTAATTGTAACAGCTCATGCATTTATTATAATTTTCTTTA
TAGTAATACCAATTATAATTGGAGGATTTGGAAATTGATTAGTTCCTTTAATATTAGGAG
CCCCTGATATAGCCTTTCCTCGAATAAATAATATAAGTTTTTGAATACTACCTCCTTCAT
TGACTCTTCTATTATCAAGCTCAATAGTAGAAAATGGAGCAGGAACTGGGTGAACAGTTT
ATCCTCCTCTCTCTTCAGGAACAGCTCATGCTGGAGCTTCTGTTGATTTAGCTATTTTTT

CTCTTCATTTAGCTGGAATTTCCTCAATTTTAGGGGCAGTAAATTTTATTACAACTGTAA
TTAATATACGATCGTCAGGAATTACTTTAGATCGACTACCCTTATTTGTTTGATCTGTAG
TTATTACAGCTATCTTATTACTTCTTTCTCTTCCTGTTTTAGCTGGAGCTATTACTATGT
TATTAACAGACCGAAACTTAAATACATCTTTCTTTGATCCAATCGGAGGAGGAGATCCTA
TT
>KX446449.1
TCGGAACTTCTTTAAGAATTTTAATTCGTGCTGAACTTAGCCACCCTGGTATATTTATTG
GGAATGACCAAATTTATAATGTAATTGTAACAGCTCATGCATTTATTATAATTTTCTTTA
TAGTAATACCAATTATAATTGGAGGATTTGGAAATTGATTAGTTCCTTTAATATTAGGAG
CCCCTGATATAGCTTTTCCTCGAATAAATAATATAAGTTTTTGAATACTACCTCCTTCAT
TGACTCTTCTATTATCAAGCTCAATAGTAGAAAATGGAGCAGGAACTGGGTGAACAGTTT
ATCCTCCTCTCTCTTCAGGAACAGCTCATGCTGGAGCTTCTGTTGATTTAGCTATTTTTT
CTCTTCATTTAGCTGGAATTTCCTCAATTTTAGGGGCAGTAAATTTTATTACAACTGTAA
TTAATATACGATCGTCAGGAATTACTTTAGATCGACTACCCTTATTTGTTTGATCTGTAG
TTATTACAGCTATCTTATTACTTCTTTCTCTTCCTGTTTTAGCTGGAGCTATTACTATGT
TATTAACAGACCGAAACTTAAATACATCTTTCTTTGATCCAATCGGAGGAGGAGATCCTA
TT
>KX446450.1
TCGGAACTTCTCTAAGAATTTTAATTCGTGCTGAACTTAGCCACCCTGGTATATTTATTG
GGAATGACCAAATTTATAATGTAATTGTAACAGCTCATGCATTTATTATAATTTTCTTTA
TAGTAATGCCAATTATAATTGGAGGATTTGGAAATTGATTAGTTCCTTTAATATTAGGAG
CCCCTGATATAGCTTTCCCTCGAATGAATAATATAAGTTTTTGAATACTACCTCCTTCAT
TGACTCTTCTATTATCAAGCTCAATAGTAGAAAATGGAGCAGGAACTGGATGAACAGTTT
ATCCCCCTCTCTCTTCAGGAACAGCTCATGCTGGAGCTTCTGTTGATTTAGCTATTTTTT
CTCTTCATTTAGCTGGAATTTCCTCAATTTTAGGAGCAGTAAATTTTATTACAACTGTAA
TTAATATACGATCGTCAGGGATTACTTTAGATCGACTACCCTTATTTGTTTGATCTGTAG
TTATTACAGCTATCTTATTACTTCTTTCCCTTCCTGTTTTAGCTGGAGCTATTACTATAT
TATTAACAGACCGAAACTTAAATACATCTTTCTTTGATCCAATCGGAGGGGGAGACCCTA
TT
>KX446451.1
TCGGAACTTCTCTAAGAATTTTAATTCGTGCTGAACTTAGCCACCCTGGTATATTTATTG
GGAATGACCAAATTTATAATGTAATTGTAACAGCTCATGCATTTATTATAATTTTCTTTA
TAGTAATGCCAATTATAATTGGAGGATTTGGAAATTGATTAGTTCCTTTAATATTAGGAG
CCCCTGATATAGCTTTCCCTCGAATGAATAATATAAGTTTTTGAATACTACCTCCTTCAT
TGACTCTTCTATTATCAAGCTCAATAGTAGAAAATGGAGCAGGAACTGGGTGAACAGTTT
ATCCTCCTCTCTCTTCAGGAACAGCTCATGCTGGAGCTTCTGTTGATTTAGCTATTTTTT
CTCTTCATTTAGCTGGAATTTCCTCAATTTTAGGGGCAGTAAATTTTATTACAACTGTAA
TTAATATACGATCGTCAGGGATTACTTTAGATCGACTACCCTTATTTGTTTGATCTGTAG
TTATTACAGCTATCTTATTACTTCTTTCTCTTCCTGTTTTAGCTGGAGCTATTACTATAT
TATTAACAGACCGAAACTTAAATACATCTTTCTTTGATCCAATCGGAGGGGGAGACCCTA
TT
>KX446452.1
TCGGAACTTCTCTAAGAATTTTAATTCGTGCTGAACTTAGCCACCCTGGTATATTTATTG
GGAATGACCAAATTTATAATGTAATTGTAACAGCTCATGCATTTATTATAATTTTCTTTA
TAGTAATGCCAATTATAATTGGAGGATTTGGAAATTGATTAGTTCCTTTAATATTAGGAG
CCCCTGATATAGCTTTCCCTCGAATGAATAATATAAGTTTTTGAATACTACCTCCTTCAT
TGACTCTTCTATTATCAAGCTCAATAGTAGAAAATGGAGCAGGAACTGGGTGAACAGTTT
ATCCTCCTCTCTCTTCAGGAACAGCTCATGCTGGAGCTTCTGTTGATTTAGCTATTTTTT
CTCTTCATTTAGCTGGAATTTCCTCAATTTTAGGGGCAGTAAATTTTATTACAACTGTAA
TTAATATACGATCGTCAGGGATTACTTTAGATCGACTACCCTTATTTGTTTGATCTGTAG
TTATTACAGCTATCTTATTACTTCTTTCTCTTCCTGTTTTAGCTGGAGCTATTACTATAT
TATTAACAGACCGAAACTTAAATACATCTTTCTTTGATCCAATCGGAGGGGGAGACCCTA
TT
>KX446453.1
TCGGAACTTCTCTAAGAATTTTAATTCGTGCTGAACTTAGCCACCCTGGTATATTTATTG
GGAATGACCAAATTTATAATGTAATTGTAACAGCTCATGCATTTATTATAATTTTCTTTA
TAGTAATGCCAATTATAATTGGAGGATTTGGAAATTGATTAGTTCCTTTAATATTAGGAG

CCCCTGATATAGCTTTCCCTCGAATGAATAATATAAGTTTTTGAATACTACCTCCTTCAT
TGACTCTTCTATTATCAAGCTCAATAGTAGAAAATGGAGCAGGAACTGGGTGAACAGTTT
ATCCTCCTCTCTCTTCAGGAACAGCTCATGCTGGAGCTTCTGTTGATTTAGCTATTTTTT
CTCTTCATTTAGCTGGAATTTCCTCAATTTTAGGGGCAGTAAATTTTATTACAACTGTAA
TTAATATACGATCGTCAGGGATTACTTTAGATCGACTACCCTTATTTGTTTGATCTGTAG
TTATTACAGCTATCTTATTACTTCTTTCTCTTCCTGTTTTAGCTGGAGCTATTACTATAT
TATTAACAGACCGAAACTTAAATACATCTTTCTTTGATCCAATCGGAGGGGGAGACCCTA
TT
>KX446454.1
TCGGAACTTCTCTAAGAATTTTAATTCGTGCTGAACTTAGCCACCCTGGTATATTTATTG
GGAATGACCAAATTTATAATGTAATTGTAACAGCTCATGCATTTATTATAATTTTCTTTA
TAGTAATGCCAATTATAATTGGAGGATTTGGAAATTGATTAGTTCCTTTAATATTAGGAG
CCCCTGATATAGCTTTCCCTCGAATGAATAATATAAGTTTTTGAATACTACCTCCTTCAT
TGACTCTTCTATTATCAAGCTCAATAGTAGAAAATGGAGCAGGAACTGGGTGAACAGTTT
ATCCTCCTCTCTCTTCAGGAACAGCTCATGCTGGAGCTTCTGTTGATTTAGCTATTTTTT
CTCTTCATTTAGCTGGAATTTCCTCAATTTTAGGGGCAGTAAATTTTATTACAACTGTAA
TTAATATACGATCGTCAGGGATTACTTTAGATCGACTACCCTTATTTGTTTGATCTGTAG
TTATTACAGCTATCTTATTACTTCTTTCTCTTCCTGTTTTAGCTGGAGCTATTACTATAT
TATTAACAGACCGAAACTTAAATACATCTTTCTTTGATCCAATCGGAGGGGGAGACCCTA
TT
>KX446455.1
TCGGAACTTCTCTAAGAATTTTAATTCGTGCTGAACTTAGCCACCCTGGTATATTTATTG
GGAATGACCAAATTTATAATGTAATTGTAACAGCTCATGCATTTATTATAATTTTCTTTA
TAGTAATGCCAATTATAATTGGAGGATTTGGAAATTGATTAGTTCCTTTAATATTAGGAG
CCCCTGATATAGCTTTCCCTCGAATGAATAATATAAGTTTTTGAATACTACCTCCTTCAT
TGACTCTTCTATTATCAAGCTCAATAGTAGAAAATGGAGCAGGAACTGGGTGAACAGTTT
ATCCTCCTCTCTCTTCAGGAACAGCTCATGCTGGAGCTTCTGTTGATTTAGCTATTTTTT
CTCTTCATTTAGCTGGAATTTCCTCAATTTTAGGGGCAGTAAATTTTATTACAACTGTAA
TTAATATACGATCGTCAGGGATTACTTTAGATCGACTACCCTTATTTGTTTGATCTGTAG
TTATTACAGCTATCTTATTACTTCTTTCTCTTCCTGTTTTAGCTGGAGCTATTACTATAT
TATTAACAGACCGAAACTTAAATACATCTTTCTTTGATCCAATCGGAGGGGGAGACCCTA
TT
>KX446456.1
TCGGAACTTCTCTAAGAATTTTAATTCGTGCTGAACTTAGCCACCCTGGTATATTTATTG
GGAATGACCAAATTTATAATGTAATTGTAACAGCTCATGCATTTATTATAATTTTCTTTA
TAGTAATACCAATTATAATTGGAGGATTTGGAAATTGATTAGTTCCTTTAATATTAGGAG
CCCCTGATATAGCTTTCCCTCGAATAAATAATATAAGTTTTTGAATACTACCTCCTTCAT
TGACTCTTCTATTATCAAGCTCAATAGTAGAAAATGGGGCAGGAACTGGGTGAACAGTTT
ATCCTCCTCTCTCTTCAGGAACAGCTCATGCTGGAGCTTCTGTTGATTTAGCTATTTTTT
CTCTTCATTTAGCTGGAATTTCCTCAATTTTAGGGGCAGTAAATTTTATTACAACTGTAA
TTAATATACGATCGTCAGGGATTACTTTAGATCGACTACCCTTATTTGTTTGATCTGTAG
TTATTACAGCTATCTTATTACTTCTTTCTCTTCCTGTTTTAGCCGGAGCTATTACTATAT
TATTAACAGACCGAAACTTAAATACATCTTTCTTTGATCCAATCGGAGGAGGAGACCCTA
TT
>KX446457.1
TCGGAACTTCTCTAAGAATTTTAATTCGTGCTGAACTTAGCCACCCTGGTATATTTATTG
GGAATGACCAAATTTATAATGTAATTGTAACAGCTCATGCATTTATTATAATTTTCTTTA
TAGTAATGCCAATTATAATTGGAGGATTTGGAAATTGATTAGTTCCTTTAATATTAGGAG
CCCCTGATATAGCTTTCCCTCGAATGAATAATATAAGTTTTTGAATACTACCTCCTTCAT
TGACTCTTCTATTATCAAGCTCAATAGTAGAAAATGGAGCAGGAACTGGGTGAACAGTTT
ATCCTCCTCTCTCTTCAGGAACAGCTCATGCTGGAGCTTCTGTTGATTTAGCTATTTTTT
CTCTTCATTTAGCTGGAATTTCCTCAATTTTAGGGGCAGTAAATTTTATTACAACTGTAA
TTAATATACGATCGTCAGGGATTACTTTAGATCGACTACCCTTATTTGTTTGATCTGTAG
TTATTACAGCTATCTTATTACTTCTTTCTCTTCCTGTTTTAGCTGGAGCTATTACTATAT
TATTAACAGACCGAAACTTAAATACATCTTTCTTTGATCCAATCGGAGGGGGAGACCCTA
TT
>KX446458.1

TCGGAACTTCTCTAAGAATTTTAATTCGTGCTGAACTTAGCCACCCTGGTATATTTATTG
GGAATGACCAAATTTATAATGTAATTGTAACAGCTCATGCATTTATTATAATTTTCTTTA
TAGTAATGCCAATTATAATTGGAGGATTTGGAAATTGATTAGTTCCTTTAATATTAGGAG
CCCCTGATATAGCTTTCCCTCGAATGAATAATATAAGTTTTTGAATACTACCTCCTTCAT
TGACTCTTCTATTATCAAGCTCAATAGTAGAAAATGGAGCAGGAACTGGGTGAACAGTTT
ATCCTCCTCTCTCTTCAGGAACAGCTCATGCTGGAGCTTCTGTTGATTTAGCTATTTTTT
CTCTTCATTTAGCTGGAATTTCCTCAATTTTAGGGGCAGTAAATTTTATTACAACTGTAA
TTAATATACGATCGTCAGGGATTACTTTAGATCGACTACCTTTATTTGTTTGATCTGTAG
TTATTACAGCTATCTTATTACTTCTTTCTCTTCCTGTTTTAGCTGGAGCTATTACTATAT
TATTAACAGACCGAAACTTAAATACATCTTTCTTTGACCCAATTGGAGGGGGAGACCCTA
TT
>KX446459.1
TCGGAACTTCTCTAAGAATTTTAATTCGTGCTGAACTTAGCCACCCTGGTATATTTATTG
GGAATGACCAAATTTATAATGTAATTGTAACAGCTCATGCATTTATTATAATTTTCTTTA
TAGTAATACCAATTATAATTGGAGGATTTGGAAATTGATTAGTTCCTTTAATATTAGGAG
CCCCTGATATAGCTTTCCCTCGAATAAATAATATAAGTTTTTGAATACTACCTCCTTCAT
TGACTCTTCTATTATCAAGCTCAATAGTAGAAAATGGGGCAGGAACTGGGTGAACAGTTT
ATCCTCCTCTCTCTTCAGGAACAGCTCATGCTGGAGCTTCTGTTGATTTAGCTATTTTTT
CTCTTCATTTAGCTGGAATTTCCTCAATTTTAGGGGCAGTAAATTTTATTACAACTGTAA
TTAATATACGATCGTCAGGGATTACTTTAGATCGACTACCCTTATTTGTTTGATCTGTAG
TTATTACAGCTATCTTATTACTTCTTTCTCTTCCTGTTTTAGCTGGAGCTATTACTATAT
TATTAACAGACCGAAACTTAAATACATCTTTCTTTGATCCAATCGGAGGAGGAGACCCTA
TT
>KX446460.1
TCGGAACTTCTCTAAGAATTTTAATTCGTGCTGAACTTAGCCACCCTGGTATATTTATTG
GGAATGACCAAATTTATAATGTAATTGTAACAGCTCATGCATTTATTATAATTTTCTTTA
TAGTAATACCAATTATAATTGGAGGATTTGGAAATTGATTAGTTCCTTTAATATTAGGAG
CCCCTGATATAGCTTTCCCTCGAATGAATAATATAAGTTTTTGAATACTACCTCCTTCAT
TGACTCTTCTATTATCAAGCTCAATAGTAGAAAATGGGGCAGGAACTGGGTGAACAGTTT
ATCCTCCTCTCTCTTCAGGAACAGCTCATGCTGGAGCTTCTGTTGATTTAGCTATTTTTT
CTCTTCATTTAGCTGGAATTTCCTCAATTTTAGGGGCAGTAAATTTTATTACAACTGTAA
TTAATATACGATCGTCAGGAATTACTTTAGATCGACTACCCTTATTTGTTTGATCTGTAG
TTATTACAGCTATCTTATTACTTCTTTCTCTTCCTGTTTTAGCTGGAGCTATTACTATAT
TATTAACAGACCGAAACTTAAATACATCTTTCTTTGATCCAATCGGAGGAGGAGACCCTA
TT
>KX446461.1
TCGGAACTTCTCTAAGAATTTTAATTCGTGCTGAACTTAGCCACCCTGGTATATTTATTG
GGAATGACCAAATTTATAATGTAATTGTAACAGCTCATGCATTTATTATAATTTTCTTTA
TAGTAATGCCAATTATAATTGGAGGATTTGGAAATTGATTAGTTCCTTTAATATTAGGAG
CCCCTGATATAGCTTTCCCTCGAATGAATAATATAAGTTTTTGAATACTACCTCCTTCAT
TGACTCTTCTATTATCAAGCTCAATAGTAGAAAATGGAGCAGGAACTGGGTGAACAGTTT
ATCCTCCTCTCTCTTCAGGAACAGCTCATGCTGGAGCTTCTGTTGATTTAGCTATTTTTT
CTCTTCATTTAGCTGGAATTTCCTCAATTTTAGGGGCAGTAAATTTTATTACAACTGTAA
TTAATATACGATCGTCAGGGATTACTTTAGATCGACTACCTTTATTTGTTTGATCTGTAG
TTATTACAGCTATCTTATTACTTCTTTCTCTTCCTGTTTTAGCTGGAGCTATTACTATAT
TATTAACAGACCGAAACTTAAATACATCTTTCTTTGACCCAATTGGAGGGGGAGACCCTA
TT
>KX446462.1
TCGGAACTTCTCTAAGAATTTTAATTCGTGCTGAACTTAGCCACCCTGGTATATTTATTG
GGAATGACCAAATTTATAATGTAATTGTAACAGCTCATGCATTTATTATAATTTTCTTTA
TAGTAATGCCAATTATAATTGGAGGATTTGGAAATTGATTAGTTCCTTTAATATTAGGAG
CCCCTGATATAGCTTTCCCTCGAATGAATAATATAAGTTTTTGAATACTACCTCCTTCAT
TGACTCTTCTATTATCAAGCTCAATAGTAGAAAATGGAGCAGGAACTGGGTGAACAGTTT
ATCCTCCTCTCTCTTCAGGAACAGCTCATGCTGGAGCTTCTGTTGATTTAGCTATTTTTT
CTCTTCATTTAGCTGGAATTTCCTCAATTTTAGGGGCAGTAAATTTTATTACAACTGTAA
TTAATATACGATCGTCAGGGATTACTTTAGATCGACTACCTTTATTTGTTTGATCTGTAG
TTATTACAGCTATCTTATTACTTCTTTCTCTTCCTGTTTTAGCTGGAGCTATTACTATAT

TATTAACAGACCGAAACTTAAATACATCTTTCTTTGACCCAATTGGAGGGGGAGACCCTA
TT
>KX446463.1
TCGGAACTTCTCTAAGAATTTTAATTCGTGCTGAACTTAGCCACCCTGGTATATTTATTG
GGAATGATCAAATTTATAATGTAATTGTAACAGCTCATGCATTTATTATAATTTTCTTTA
TAGTAATGCCAATTATAATTGGAGGATTTGGAAATTGATTAGTTCCTTTAATATTAGGAG
CCCCTGATATAGCTTTCCCTCGAATGAATAATATAAGTTTTTGAATACTACCTCCTTCAT
TGACTCTTCTATTATCAAGCTCAATAGTAGAAAATGGAGCAGGAACTGGGTGAACAGTTT
ATCCTCCTCTCTCTTCAGGAACAGCTCATGCTGGAGCTTCTGTTGATTTAGCTATTTTTT
CTCTTCATTTAGCTGGAATTTCCTCAATTTTAGGGGCAGTAAATTTTATTACAACTGTAA
TTAATATACGATCGTCAGGGATTACTTTAGATCGACTACCCTTATTTGTTTGATCTGTAG
TTATTACGGCTATCTTATTACTTCTTTCTCTTCCTGTTTTAGCTGGAGCTATTACTATAT
TATTAACAGACCGAAACTTAAATACATCTTTCTTTGATCCAATCGGAGGGGGAGACCCTA
TT
>KX446464.1
TCGGAACTTCTCTAAGAATTTTAATTCGTGCTGAACTTAGCCACCCTGGTATATTTATTG
GGAATGACCAAATTTATAATGTAATTGTAACAGCTCATGCATTTATTATAATTTTTTTTA
TAGTAATGCCAATTATAATTGGAGGATTTGGAAATTGATTAGTTCCTTTAATATTAGGAG
CCCCTGATATAGCTTTCCCTCGAATGAATAATATAAGTTTTTGAATACTACCTCCTTCAT
TGACTCTTCTATTATCAAGCTCAATAGTAGAAAATGGAGCAGGAACTGGGTGAACAGTTT
ATCCTCCTCTCTCTTCAGGAACAGCTCATGCTGGAGCTTCTGTTGATTTAGCTATTTTTT
CTCTTCATTTAGCTGGAATTTCCTCAATTTTAGGAGCAGTAAATTTTATTACAACTGTAA
TTAATATACGATCGTCAGGAATTACTTTAGATCGACTACCCTTATTTGTTTGATCTGTAG
TTATTACAGCTATCTTATTACTTCTTTCTCTTCCTGTTTTAGCTGGAGCTATTACTATAT
TATTAACAGACCGAAACTTAAATACATCTTTCTTTGATCCAATCGGAGGGGGAGACCCTA
TT
>KX446465.1
TCGGAACTTCTCTAAGAATTTTAATTCGTGCTGAACTTAGCCACCCTGGTATATTTATTG
GGAATGACCAAATTTATAATGTAATTGTAACAGCTCATGCATTTATTATAATTTTCTTTA
TAGTAATACCAATTATAATTGGAGGATTTGGAAATTGATTAGTTCCTTTAATATTAGGAG
CCCCTGATATAGCCTTTCCTCGAATAAATAATATAAGTTTTTGAATACTACCTCCTTCAT
TGACTCTTCTATTATCAAGCTCAATAGTAGAAAATGGAGCAGGAACTGGGTGAACAGTTT
ATCCTCCTCTCTCTTCAGGAACAGCTCATGCTGGGGCTTCTGTTGATTTAGCTATTTTTT
CTCTTCATTTAGCTGGGATTTCTTCAATTTTAGGAGCAGTAAATTTTATTACAACTGTAA
TTAATATACGATCGTCAGGAATTACTTTAGATCGACTACCCTTATTTGTTTGATCTGTGG
TTATTACAGCTATCTTATTACTTCTTTCTCTTCCTGTTTTAGCTGGAGCTATTACTATAT
TATTAACAGACCGAAACTTAAATACCTCTTTCTTTGATCCAATCGGAGGAGGAGACCCTA
TT
>KX446466.1
TCGGAACTTCTCTAAGAATTTTAATTCGTGCTGAACTTAGCCACCCTGGTATATTTATTG
GGAATGACCAAATTTATAATGTAATTGTAACAGCTCATGCATTTATTATAATTTTCTTTA
TAGTAATGCCAATTATAATTGGAGGATTTGGAAATTGATTAGTTCCTTTAATATTAGGAG
CCCCTGATATAGCTTTCCCTCGAATGAATAATATAAGTTTTTGAATACTACCTCCTTCAT
TGACTCTTCTATTATCAAGCTCAATAGTAGAAAATGGAGCAGGAACTGGGTGAACAGTTT
ATCCTCCTCTCTCTTCAGGAACAGCTCATGCTGGAGCTTCTGTTGATTTAGCTATTTTTT
CTCTTCATTTAGCTGGAATTTCCTCAATTTTAGGGGCAGTAAATTTTATTACAACTGTAA
TTAATATACGATCGTCAGGGATTACTTTAGATCGACTACCTTTATTTGTTTGATCTGTAG
TTATTACAGCTATCTTATTACTTCTTTCTCTTCCTGTTTTAGCTGGAGCTATTACTATAT
TATTAACAGACCGAAACTTAAATACATCTTTCTTTGACCCAATTGGAGGGGGAGACCCTA
TT
>KX446467.1
TCGGAACTTCTCTAAGAATTTTAATTCGTGCTGAACTTAGCCACCCTGGTATATTTATTG
GGAATGACCAAATTTATAATGTAATTGTAACAGCTCATGCATTTATTATAATTTTCTTTA
TAGTAATGCCAATTATAATTGGAGGATTTGGAAATTGATTAGTTCCTTTAATATTAGGAG
CCCCTGATATAGCTTTCCCTCGAATGAATAATATAAGTTTTTGAATACTACCTCCTTCAT
TGACTCTTCTATTATCAAGCTCAATAGTAGAAAATGGAGCAGGAACTGGGTGAACAGTTT
ACCCTCCTCTCTCTTCAGGAACAGCTCATGCTGGAGCTTCTGTTGATTTAGCTATTTTTT

CTCTTCATTTAGCTGGAATTTCCTCAATTTTAGGGGCAGTAAATTTTATTACAACTGTAA
TTAATATACGATCGTCAGGGATTACTTTAGATCGACTACCCTTATTTGTTTGATCTGTAG
TTATTACAGCTATCTTATTACTTCTTTCTCTTCCTGTTTTAGCTGGAGCTATTACTATAT
TATTAACAGACCGAAACTTAAATACATCTTTCTTTGATCCAATTGGAGGGGGAGACCCTA
TT
>KX446468.1
TCGGAACTTCTCTAAGAATTTTAATTCGTGCTGAACTTAGCCACCCTGGTATATTTATTG
GGAATGACCAAATTTATAATGTAATTGTAACAGCTCATGCATTTATTATAATTTTCTTTA
TAGTAATACCAATTATAATTGGAGGATTTGGAAATTGATTAGTTCCTTTAATATTAGGAG
CCCCTGATATAGCTTTCCCTCGAATAAATAATATAAGTTTTTGAATACTACCTCCTTCAT
TGACTCTTCTATTATCAAGCTCAATAGTAGAAAATGGGGCAGGAACTGGGTGAACAGTTT
ATCCTCCTCTCTCTTCAGGAACAGCTCATGCTGGAGCTTCTGTTGATTTAGCTATTTTTT
CTCTTCATTTAGCTGGAATTTCCTCAATTTTAGGGGCAGTAAATTTTATTACAACTGTAA
TTAATATACGATCGTCAGGGATTACTTTAGATCGACTACCCTTATTTGTTTGATCTGTAG
TTATTACAGCTATCTTATTACTTCTTTCTCTTCCTGTTTTAGCTGGAGCTATTACTATAT
TATTAACAGACCGAAACTTAAATACATCTTTCTTTGATCCAATCGGAGGAGGAGACCCTA
TT
>KX446469.1
TCGGAACTTCTCTAAGAATTTTAATTCGTGCTGAACTTAGCCACCCTGGTATATTTATTG
GGAATGACCAAATTTATAATGTAATTGTAACAGCTCATGCATTTATTATAATTTTCTTTA
TAGTAATGCCAATTATAATTGGAGGATTTGGAAATTGATTAGTTCCTTTAATATTAGGAG
CCCCTGATATAGCTTTCCCTCGAATGAATAATATAAGTTTTTGAATACTACCTCCTTCAT
TGACTCTTCTATTATCAAGCTCAATAGTAGAAAATGGAGCAGGAACTGGATGAACAGTTT
ATCCTCCTCTCTCTTCAGGAACAGCTCATGCTGGAGCTTCTGTTGATTTAGCTATTTTTT
CTCTTCATTTAGCTGGAATTTCCTCAATTTTAGGGGCAGTAAATTTTATTACAACTGTAA
TTAATATACGATCATCAGGGATTACTTTAGATCGACTACCCTTATTTGTATGATCTGTAG
TTATTACAGCTATCTTATTACTTCTTTCTCTTCCTGTTTTAGCTGGAGCTATTACTATAT
TATTAACAGACCGAAACTTAAATACATCTTTCTTTGATCCAATCGGAGGGGGAGACCCTA
TT
>MK890401.1
TCGGAACTTCTCTAAGAATTTTAATTCGTGCTGAACTTAGCCACCCTGGTATATTTATTG
GGAATGACCAAATTTATAATGTAATTGTAACAGCTCATGCATTTATTATAATTTTCTTTA
TAGTAATACCAATTATAATTGGAGGATTTGGAAATTGATTAGTTCCTTTAATATTAGGAG
CCCCTGATATAGCCTTTCCTCGAATAAATAATATAAGTTTTTGAATACTACCTCCTTCAT
TGACTCTTCTATTATCAAGCTCAATAGTAGAAAATGGAGCAGGAACTGGGTGAACAGTTT
ATCCTCCTCTCTCTTCAGGAACAGCTCATGCTGGAGCTTCTGTTGATTTAGCTATTTTTT
CTCTTCATTTAGCTGGAATTTCCTCAATTTTAGGGGCAGTAAATTTTATTACAACTGTAA
TTAATATACGATCGTCAGGAATTACTTTAGATCGACTACCCTTATTTGTTTGATCTGTAG
TTATTACAGCTATCTTATTACTTCTTTCTCTTCCTGTTTTAGCTGGAGCTATTACTATGT
TATTAACAGACCGAAACTTAAATACATCTTTCTTTGATCCAATCGGAGGAGGAGATCCTA
TT
>MK890402.1
TCGGAACTTCTCTAAGAATTTTAATTCGTGCTGAACTTAGCCACCCTGGTATATTTATTG
GGAATGACCAAATTTATAATGTAATTGTAACAGCTCATGCATTTATTATAATTTTCTTTA
TAGTAATGCCAATTATAATTGGAGGATTTGGAAATTGATTAGTTCCTTTAATATTAGGAG
CCCCTGATATAGCTTTCCCTCGAATGAATAATATAAGTTTTTGAATACTACCTCCTTCAT
TGACTCTTCTATTATCAAGCTCAATAGTAGAAAATGGGGCAGGAACTGGGTGAACAGTTT
ATCCTCCTCTCTCTTCAGGAACAGCTCATGCTGGAGCTTCTGTTGATTTAGCTATTTTTT
CTCTTCATTTAGCTGGAATTTCCTCAATTTTAGGGGCAGTAAATTTTATTACAACTGTGA
TTAATATGCGATCGTCAGGGATTACTTTAGATCGACTACCCTTATTTGTTTGATCTGTAG
TTATTACAGCTATCTTATTACTTCTTTCTCTTCCTGTTTTAGCTGGAGCTATTACTATAT
TATTAACAGACCGAAACTTAAATACATCTTTCTTTGATCCAATCGGAGGGGGAGACCCTA
TT
>MK890403.1
TCGGAACTTCTCTAAGAATTTTAATTCGTGCTGAACTTAGCCACCCTGGTATATTTATTG
GGAATGACCAAATTTATAATGTAATTGTAACAGCTCATGCATTTATTATAATTTTCTTTA
TAGTAATACCAATTATAATTGGAGGATTTGGAAATTGATTAGTTCCTTTAATATTAGGAG

CCCCTGATATAGCCTTTCCTCGAATAAATAATATAAGTTTTTGAATACTACCTCCTTCAT
TGACTCTTCTATTATCAAGCTCAATAGTAGAAAATGGAGCAGGAACTGGGTGAACAGTTT
ATCCTCCTCTCTCTTCAGGAACAGCTCATGCTGGAGCTTCTGTTGATTTAGCTATTTTTT
CTCTTCATTTAGCTGGAATTTCCTCAATTTTAGGGGCAGTAAATTTTATTACAACTGTAA
TTAATATACGATCGTCAGGAATTACTTTAGATCGACTACCCTTATTTGTTTGATCTGTAG
TTATTACAGCTATCTTATTACTTCTTTCTCTTCCTGTTTTAGCTGGAGCTATTACTATGT
TATTAACAGACCGAAACTTAAATACATCTTTCTTTGATCCAATCGGAGGAGGAGATCCTA
TT
>MK890404.1
TCGGAACTTCTCTAAGAATTTTAATTCGTGCTGAACTTAGCCACCCTGGTATATTTATTG
GGAATGACCAAATTTATAATGTAATTGTAACAGCTCATGCATTTATTATAATTTTCTTTA
TAGTAATACCAATTATAATTGGAGGATTTGGAAATTGATTAGTTCCTTTAATATTAGGAG
CCCCTGATATAGCCTTTCCTCGAATAAATAATATAAGTTTTTGAATACTACCTCCTTCAT
TGACTCTTCTATTATCAAGCTCAATAGTAGAAAATGGAGCAGGAACTGGGTGAACAGTTT
ATCCTCCTCTCTCTTCAGGAACAGCTCATGCTGGAGCTTCTGTTGATTTAGCTATTTTTT
CTCTTCATTTAGCTGGAATTTCCTCAATTTTAGGGGCAGTAAATTTTATTACAACTGTAA
TTAATATACGATCGTCAGGAATTACTTTAGATCGACTACCCTTATTTGTTTGATCTGTAG
TTATTACAGCTATCTTATTACTTCTTTCTCTTCCTGTTTTAGCTGGAGCTATTACTATGT
TATTAACAGACCGAAACTTAAATACATCTTTCTTTGATCCAATCGGAGGAGGAGATCCTA
TT
>MK890405.1
TCGGAACTTCTCTAAGAATTTTAATTCGTGCTGAACTTAGCCACCCTGGTATATTTATTG
GGAATGACCAAATTTATAATGTAATTGTAACAGCTCATGCATTTATTATAATTTTCTTTA
TAGTAATGCCAATTATAATTGGAGGATTTGGAAATTGATTAGTTCCTTTAATATTAGGAG
CCCCTGATATAGCTTTCCCTCGAATGAATAATATAAGTTTTTGAATACTACCTCCTTCAT
TGACTCTTCTATTATCAAGCTCAATAGTAGAAAATGGGGCAGGAACTGGGTGAACAGTTT
ATCCTCCTCTCTCTTCAGGAACAGCTCATGCTGGAGCTTCTGTTGATTTAGCTATTTTTT
CTCTTCATTTAGCTGGAATTTCCTCAATTTTAGGGGCAGTAAATTTTATTACAACTGTGA
TTAATATGCGATCGTCAGGGATTACTTTAGATCGACTACCCTTATTTGTTTGATCTGTAG
TTATTACAGCTATCTTATTACTTCTTTCTCTTCCTGTTTTAGCTGGAGCTATTACTATAT
TATTAACAGACCGAAACTTAAATACATCTTTCTTTGATCCAATCGGAGGGGGAGACCCTA
TT
>MK890406.1
TCGGAACTTCTCTAAGAATTTTAATTCGTGCTGAACTTAGCCACCCTGGTATATTTATTG
GGAATGACCAAATTTATAATGTAATTGTAACAGCTCATGCATTTATTATAATTTTCTTTA
TAGTAATGCCAATTATAATTGGAGGATTTGGAAATTGATTAGTTCCTTTAATATTAGGAG
CCCCTGATATAGCTTTCCCTCGAATGAATAATATAAGTTTTTGAATACTACCTCCTTCAT
TGACTCTTCTATTATCAAGCTCAATAGTAGAAAATGGGGCAGGAACTGGGTGAACAGTTT
ATCCTCCTCTCTCTTCAGGAACAGCTCATGCTGGAGCTTCTGTTGATTTAGCTATTTTTT
CTCTTCATTTAGCTGGAATTTCCTCAATTTTAGGGGCAGTAAATTTTATTACAACTGTGA
TTAATATGCGATCGTCAGGGATTACTTTAGATCGACTACCCTTATTTGTTTGATCTGTAG
TTATTACAGCTATCTTATTACTTCTTTCTCTTCCTGTTTTAGCTGGAGCTATTACTATAT
TATTAACAGACCGAAACTTAAATACATCTTTCTTTGATCCAATCGGAGGGGGAGACCCTA
TT
>MK890407.1
TCGGAACTTCTCTAAGAATTTTAATTCGTGCTGAACTTAGCCACCCTGGTATATTTATTG
GGAATGACCAAATTTATAATGTAATTGTAACAGCTCATGCATTTATTATAATTTTCTTTA
TAGTAATACCAATTATAATTGGAGGATTTGGAAATTGATTAGTTCCTTTAATATTAGGAG
CCCCTGATATAGCCTTTCCTCGAATAAATAATATAAGTTTTTGAATACTACCTCCTTCAT
TGACTCTTCTATTATCAAGCTCAATAGTAGAAAATGGAGCAGGAACTGGGTGAACAGTTT
ATCCTCCTCTCTCTTCAGGAACAGCTCATGCTGGAGCTTCTGTTGATTTAGCTATTTTTT
CTCTTCATTTAGCTGGAATTTCCTCAATTTTAGGGGCAGTAAATTTTATTACAACTGTAA
TTAATATACGATCGTCAGGAATTACTTTAGATCGACTACCCTTATTTGTTTGATCTGTAG
TTATTACAGCTATCTTATTACTTCTTTCTCTTCCTGTTTTAGCTGGAGCTATTACTATGT
TATTAACAGACCGAAACTTAAATACATCTTTCTTTGATCCAATCGGAGGAGGAGATCCTA
TT
>MK890408.1

TCGGAACTTCTCTAAGAATTTTAATTCGTGCTGAACTTAGCCACCCTGGTATATTTATTG
GGAATGACCAAATTTATAATGTAATTGTAACAGCTCATGCATTTATTATAATTTTCTTTA
TAGTAATACCAATTATAATTGGAGGATTTGGAAATTGATTAGTTCCTTTAATATTAGGAG
CCCCTGATATAGCCTTTCCTCGAATAAATAATATAAGTTTTTGAATACTACCTCCTTCAT
TGACTCTTCTATTATCAAGCTCAATAGTAGAAAATGGAGCAGGAACTGGGTGAACAGTTT
ATCCTCCTCTCTCTTCAGGAACAGCTCATGCTGGAGCTTCTGTTGATTTAGCTATTTTTT
CTCTTCATTTAGCTGGAATTTCCTCAATTTTAGGGGCAGTAAATTTTATTACAACTGTAA
TTAATATACGATCGTCAGGAATTACTTTAGATCGACTACCCTTATTTGTTTGATCTGTAG
TTATTACAGCTATCTTATTACTTCTTTCTCTTCCTGTTTTAGCTGGAGCTATTACTATGT
TATTAACAGACCGAAACTTAAATACATCTTTCTTTGATCCAATCGGAGGAGGAGATCCTA
TT
>MK890409.1
TCGGAACTTCTCTAAGAATTTTAATTCGTGCTGAACTTAGCCACCCTGGTATATTTATTG
GGAATGACCAAATTTATAATGTAATTGTAACAGCTCATGCATTTATTATAATTTTCTTTA
TAGTAATACCAATTATAATTGGAGGATTTGGAAATTGATTAGTTCCTTTAATATTAGGAG
CCCCTGATATAGCCTTTCCTCGAATAAATAATATAAGTTTTTGAATACTACCTCCTTCAT
TGACTCTTCTATTATCAAGCTCAATAGTAGAAAATGGAGCAGGAACTGGGTGAACAGTTT
ATCCTCCTCTCTCTTCAGGAACAGCTCATGCTGGAGCTTCTGTTGATTTAGCTATTTTTT
CTCTTCATTTAGCTGGAATTTCCTCAATTTTAGGGGCAGTAAATTTTATTACAACTGTAA
TTAATATACGATCGTCAGGAATTACTTTAGATCGACTACCCTTATTTGTTTGATCTGTAG
TTATTACAGCTATCTTATTACTTCTTTCTCTTCCTGTTTTAGCTGGAGCTATTACTATGT
TATTAACAGACCGAAACTTAAATACATCTTTCTTTGATCCAATCGGAGGAGGAGATCCTA
TT
>MK890410.1
TCGGAACTTCTCTAAGAATTTTAATTCGTGCTGAACTTAGCCACCCTGGTATATTTATTG
GGAATGACCAAATTTATAATGTAATTGTAACAGCTCATGCATTTATTATAATTTTCTTTA
TAGTAATACCAATTATAATTGGAGGATTTGGAAATTGATTAGTTCCTTTAATATTAGGAG
CCCCTGATATAGCCTTTCCTCGAATAAATAATATAAGTTTTTGAATACTACCTCCTTCAT
TGACTCTTCTATTATCAAGCTCAATAGTAGAAAATGGAGCAGGAACTGGGTGAACAGTTT
ATCCTCCTCTCTCTTCAGGAACAGCTCATGCTGGAGCTTCTGTTGATTTAGCTATTTTTT
CTCTTCATTTAGCTGGAATTTCCTCAATTTTAGGGGCAGTAAATTTTATTACAACTGTAA
TTAATATACGATCGTCAGGAATTACTTTAGATCGACTACCCTTATTTGTTTGATCTGTAG
TTATTACAGCTATCTTATTACTTCTTTCTCTTCCTGTTTTAGCTGGAGCTATTACTATGT
TATTAACAGACCGAAACTTAAATACATCTTTCTTTGATCCAATCGGAGGAGGAGATCCTA
TT
>MK890411.1
TCGGAACTTCTCTAAGAATTTTAATTCGTGCTGAACTTAGCCACCCTGGTATATTTATTG
GGAATGACCAAATTTATAATGTAATTGTAACAGCTCATGCATTTATTATAATTTTCTTTA
TAGTAATGCCAATTATAATTGGAGGATTTGGAAATTGATTAGTTCCTTTAATATTAGGAG
CCCCTGATATAGCTTTCCCTCGAATGAATAATATAAGTTTTTGAATACTACCTCCTTCAT
TGACTCTTCTATTATCAAGCTCAATAGTAGAAAATGGGGCAGGAACTGGGTGAACAGTTT
ATCCTCCTCTCTCTTCAGGAACAGCTCATGCTGGAGCTTCTGTTGATTTAGCTATTTTTT
CTCTTCATTTAGCTGGAATTTCCTCAATTTTAGGGGCAGTAAATTTTATTACAACTGTGA
TTAATATGCGATCGTCAGGGATTACTTTAGATCGACTACCCTTATTTGTTTGATCTGTAG
TTATTACAGCTATCTTATTACTTCTTTCTCTTCCTGTTTTAGCTGGAGCTATTACTATAT
TATTAACAGACCGAAACTTAAATACATCTTTCTTTGATCCAATCGGAGGGGGAGACCCTA
TT
>MK890412.1
TCGGAACTTCTCTAAGAATTTTAATTCGTGCTGAACTTAGCCACCCTGGTATATTTATTG
GGAATGACCAAATTTATAATGTAATTGTAACAGCTCATGCATTTATTATAATTTTCTTTA
TAGTAATACCAATTATAATTGGAGGATTTGGAAATTGATTAGTTCCTTTAATATTAGGAG
CCCCTGATATAGCCTTTCCTCGAATAAATAATATAAGTTTTTGAATACTACCTCCTTCAT
TGACTCTTCTATTATCAAGCTCAATAGTAGAAAATGGAGCAGGAACTGGGTGAACAGTTT
ATCCTCCTCTCTCTTCAGGAACAGCTCATGCTGGAGCTTCTGTTGATTTAGCTATTTTTT
CTCTTCATTTAGCTGGAATTTCCTCAATTTTAGGGGCAGTAAATTTTATTACAACTGTAA
TTAATATACGATCGTCAGGAATTACTTTAGATCGACTACCCTTATTTGTTTGATCTGTAG
TTATTACAGCTATCTTATTACTTCTTTCTCTTCCTGTTTTAGCTGGAGCTATTACTATGT

TATTAACAGACCGAAACTTAAATACATCTTTCTTTGATCCAATCGGAGGAGGAGATCCTA
TT
>MK890413.1
TCGGAACTTCTCTAAGAATTTTAATTCGTGCTGAACTTAGCCACCCTGGTATATTTATTG
GGAATGACCAAATTTATAATGTAATTGTAACAGCTCATGCATTTATTATAATTTTCTTTA
TAGTAATGCCAATTATAATTGGAGGATTTGGAAATTGATTAGTTCCTTTAATATTAGGAG
CCCCTGATATAGCTTTCCCTCGAATGAATAATATAAGTTTTTGAATACTACCTCCTTCAT
TGACTCTTCTATTATCAAGCTCAATAGTAGAAAATGGGGCAGGAACTGGGTGAACAGTTT
ATCCTCCTCTCTCTTCAGGAACAGCTCATGCTGGAGCTTCTGTTGATTTAGCTATTTTTT
CTCTTCATTTAGCTGGAATTTCCTCAATTTTAGGGGCAGTAAATTTTATTACAACTGTGA
TTAATATGCGATCGTCAGGGATTACTTTAGATCGACTACCCTTATTTGTTTGATCTGTAG
TTATTACAGCTATCTTATTACTTCTTTCTCTTCCTGTTTTAGCTGGAGCTATTACTATAT
TATTAACAGACCGAAACTTAAATACATCTTTCTTTGATCCAATCGGAGGGGGAGACCCTA
TT
>MK890414.1
TCGGAACTTCTCTAAGAATTTTAATTCGTGCTGAACTTAGCCACCCTGGTATATTTATTG
GGAATGACCAAATTTATAATGTAATTGTAACAGCTCATGCATTTATTATAATTTTCTTTA
TAGTAATGCCAATTATAATTGGAGGATTTGGAAATTGATTAGTTCCTTTAATATTAGGAG
CCCCTGATATAGCTTTCCCTCGAATGAATAATATAAGTTTTTGAATACTACCTCCTTCAT
TGACTCTTCTATTATCAAGCTCAATAGTAGAAAATGGGGCAGGAACTGGGTGAACAGTTT
ATCCTCCTCTCTCTTCAGGAACAGCTCATGCTGGAGCTTCTGTTGATTTAGCTATTTTTT
CTCTTCATTTAGCTGGAATTTCCTCAATTTTAGGGGCAGTAAATTTTATTACAACTGTGA
TTAATATGCGATCGTCAGGGATTACTTTAGATCGACTACCCTTATTTGTTTGATCTGTAG
TTATTACAGCTATCTTATTACTTCTTTCTCTTCCTGTTTTAGCTGGAGCTATTACTATAT
TATTAACAGACCGAAACTTAAATACATCTTTCTTTGATCCAATCGGAGGGGGAGACCCTA
TT
>MK890415.1
TCGGAACTTCTCTAAGAATTTTAATTCGTGCTGAACTTAGCCACCCTGGTATATTTATTG
GGAATGACCAAATTTATAATGTAATTGTAACAGCTCATGCATTTATTATAATTTTCTTTA
TAGTAATACCAATTATAATTGGAGGATTTGGAAATTGATTAGTTCCTTTAATATTAGGAG
CCCCTGATATAGCCTTTCCTCGAATAAATAATATAAGTTTTTGAATACTACCTCCTTCAT
TGACTCTTCTATTATCAAGCTCAATAGTAGAAAATGGAGCAGGAACTGGGTGAACAGTTT
ATCCTCCTCTCTCTTCAGGAACAGCTCATGCTGGAGCTTCTGTTGATTTAGCTATTTTTT
CTCTTCATTTAGCTGGAATTTCCTCAATTTTAGGGGCAGTAAATTTTATTACAACTGTAA
TTAATATACGATCGTCAGGAATTACTTTAGATCGACTACCCTTATTTGTTTGATCTGTAG
TTATTACAGCTATCTTATTACTTCTTTCTCTTCCTGTTTTAGCTGGAGCTATTACTATGT
TATTAACAGACCGAAACTTAAATACATCTTTCTTTGATCCAATCGGAGGAGGAGATCCTA
TT
>MK890416.1
TCGGAACTTCTCTAAGAATTTTAATTCGTGCTGAACTTAGCCACCCTGGTATATTTATTG
GGAATGACCAAATTTATAATGTAATTGTAACAGCTCATGCATTTATTATAATTTTCTTTA
TAGTAATGCCAATTATAATTGGAGGATTTGGAAATTGATTAGTTCCTTTAATATTAGGAG
CCCCTGATATAGCTTTCCCTCGAATGAATAATATAAGTTTTTGAATACTACCTCCTTCAT
TGACTCTTCTATTATCAAGCTCAATAGTAGAAAATGGGGCAGGAACTGGGTGAACAGTTT
ATCCTCCTCTCTCTTCAGGAACAGCTCATGCTGGAGCTTCTGTTGATTTAGCTATTTTTT
CTCTTCATTTAGCTGGAATTTCCTCAATTTTAGGGGCAGTAAATTTTATTACAACTGTGA
TTAATATGCGATCGTCAGGGATTACTTTAGATCGACTACCCTTATTTGTTTGATCTGTAG
TTATTACAGCTATCTTATTACTTCTTTCTCTTCCTGTTTTAGCTGGAGCTATTACTATAT
TATTAACAGACCGAAACTTAAATACATCTTTCTTTGATCCAATCGGAGGGGGAGACCCTA
TT
>MK890417.1
TCGGAACTTCTCTAAGAATTTTAATTCGTGCTGAACTTAGCCACCCTGGTATATTTATTG
GGAATGACCAAATTTATAATGTAATTGTAACAGCTCATGCATTTATTATAATTTTCTTTA
TAGTAATACCAATTATAATTGGAGGATTTGGAAATTGATTAGTTCCTTTAATATTAGGAG
CCCCTGATATAGCCTTTCCTCGAATAAATAATATAAGTTTTTGAATACTACCTCCTTCAT
TGACTCTTCTATTATCAAGCTCAATAGTAGAAAATGGAGCAGGAACTGGGTGAACAGTTT
ATCCTCCTCTCTCTTCAGGAACAGCTCATGCTGGAGCTTCTGTTGATTTAGCTATTTTTT

CTCTTCATTTAGCTGGAATTTCCTCAATTTTAGGGGCAGTAAATTTTATTACAACTGTAA
TTAATATACGATCGTCAGGAATTACTTTAGATCGACTACCCTTATTTGTTTGATCTGTAG
TTATTACAGCTATCTTATTACTTCTTTCTCTTCCTGTTTTAGCTGGAGCTATTACTATGT
TATTAACAGACCGAAACTTAAATACATCTTTCTTTGATCCAATCGGAGGAGGAGATCCTA
TT
>MK890418.1
TCGGAACTTCTCTAAGAATTTTAATTCGTGCTGAACTTAGCCACCCTGGTATATTTATTG
GGAATGACCAAATTTATAATGTAATTGTAACAGCTCATGCATTTATTATAATTTTCTTTA
TAGTAATGCCAATTATAATTGGAGGATTTGGAAATTGATTAGTTCCTTTAATATTAGGAG
CCCCTGATATAGCTTTCCCTCGAATGAATAATATAAGTTTTTGAATACTACCTCCTTCAT
TGACTCTTCTATTATCAAGCTCAATAGTAGAAAATGGGGCAGGAACTGGGTGAACAGTTT
ATCCTCCTCTCTCTTCAGGAACAGCTCATGCTGGAGCTTCTGTTGATTTAGCTATTTTTT
CTCTTCATTTAGCTGGAATTTCCTCAATTTTAGGGGCAGTAAATTTTATTACAACTGTGA
TTAATATGCGATCGTCAGGGATTACTTTAGATCGACTACCCTTATTTGTTTGATCTGTAG
TTATTACAGCTATCTTATTACTTCTTTCTCTTCCTGTTTTAGCTGGAGCTATTACTATAT
TATTAACAGACCGAAACTTAAATACATCTTTCTTTGATCCAATCGGAGGGGGAGACCCTA
TT
>MK890419.1
TCGGAACTTCTCTAAGAATTTTAATTCGTGCTGAACTTAGCCACCCTGGTATATTTATTG
GGAATGACCAAATTTATAATGTAATTGTAACAGCTCATGCATTTATTATAATTTTCTTTA
TAGTAATACCAATTATAATTGGAGGATTTGGAAATTGATTAGTTCCTTTAATATTAGGAG
CCCCTGATATAGCCTTTCCTCGAATAAATAATATAAGTTTTTGAATACTACCTCCTTCAT
TGACTCTTCTATTATCAAGCTCAATAGTAGAAAATGGAGCAGGAACTGGGTGAACAGTTT
ATCCTCCTCTCTCTTCAGGAACAGCTCATGCTGGAGCTTCTGTTGATTTAGCTATTTTTT
CTCTTCATTTAGCTGGAATTTCCTCAATTTTAGGGGCAGTAAATTTTATTACAACTGTAA
TTAATATACGATCGTCAGGAATTACTTTAGATCGACTACCCTTATTTGTTTGATCTGTAG
TTATTACAGCTATCTTATTACTTCTTTCTCTTCCTGTTTTAGCTGGAGCTATTACTATGT
TATTAACAGACCGAAACTTAAATACATCTTTCTTTGATCCAATCGGAGGAGGAGATCCTA
TT
>MK890420.1
TCGGAACTTCTCTAAGAATTTTAATTCGTGCTGAACTTAGCCACCCTGGTATATTTATTG
GGAATGACCAAATTTATAATGTAATTGTAACAGCTCATGCATTTATTATAATTTTCTTTA
TAGTAATACCAATTATAATTGGAGGATTTGGAAATTGATTAGTTCCTTTAATATTAGGAG
CCCCTGATATAGCCTTTCCTCGAATAAATAATATAAGTTTTTGAATACTACCTCCTTCAT
TGACTCTTCTATTATCAAGCTCAATAGTAGAAAATGGAGCAGGAACTGGGTGAACAGTTT
ATCCTCCTCTCTCTTCAGGAACAGCTCATGCTGGAGCTTCTGTTGATTTAGCTATTTTTT
CTCTTCATTTAGCTGGAATTTCCTCAATTTTAGGGGCAGTAAATTTTATTACAACTGTAA
TTAATATACGATCGTCAGGAATTACTTTAGATCGACTACCCTTATTTGTTTGATCTGTAG
TTATTACAGCTATCTTATTACTTCTTTCTCTTCCTGTTTTAGCTGGAGCTATTACTATGT
TATTAACAGACCGAAACTTAAATACATCTTTCTTTGATCCAATCGGAGGAGGAGATCCTA
TT
>MK890421.1
TCGGAACTTCTCTAAGAATTTTAATTCGTGCTGAACTTAGCCACCCTGGTATATTTATTG
GGAATGACCAAATTTATAATGTAATTGTAACAGCTCATGCATTTATTATAATTTTCTTTA
TAGTAATGCCAATTATAATTGGAGGATTTGGAAATTGATTAGTTCCTTTAATATTAGGAG
CCCCTGATATAGCTTTCCCTCGAATGAATAATATAAGTTTTTGAATACTACCTCCTTCAT
TGACTCTTCTATTATCAAGCTCAATAGTAGAAAATGGGGCAGGAACTGGGTGAACAGTTT
ATCCTCCTCTCTCTTCAGGAACAGCTCATGCTGGAGCTTCTGTTGATTTAGCTATTTTTT
CTCTTCATTTAGCTGGAATTTCCTCAATTTTAGGGGCAGTAAATTTTATTACAACTGTGA
TTAATATGCGATCGTCAGGGATTACTTTAGATCGACTACCCTTATTTGTTTGATCTGTAG
TTATTACAGCTATCTTATTACTTCTTTCTCTTCCTGTTTTAGCTGGAGCTATTACTATAT
TATTAACAGACCGAAACTTAAATACATCTTTCTTTGATCCAATCGGAGGGGGAGACCCTA
TT
>MK890422.1
TCGGAACTTCTCTAAGAATTTTAATTCGTGCTGAACTTAGCCACCCTGGTATATTTATTG
GGAATGACCAAATTTATAATGTAATTGTAACAGCTCATGCATTTATTATAATTTTCTTTA
TAGTAATGCCAATTATAATTGGAGGATTTGGAAATTGATTAGTTCCTTTAATATTAGGAG

CCCCTGATATAGCTTTCCCTCGAATGAATAATATAAGTTTTTGAATACTACCTCCTTCAT
TGACTCTTCTATTATCAAGCTCAATAGTAGAAAATGGGGCAGGAACTGGGTGAACAGTTT
ATCCTCCTCTCTCTTCAGGAACAGCTCATGCTGGAGCTTCTGTTGATTTAGCTATTTTTT
CTCTTCATTTAGCTGGAATTTCCTCAATTTTAGGGGCAGTAAATTTTATTACAACTGTGA
TTAATATGCGATCGTCAGGGATTACTTTAGATCGACTACCCTTATTTGTTTGATCTGTAG
TTATTACAGCTATCTTATTACTTCTTTCTCTTCCTGTTTTAGCTGGAGCTATTACTATAT
TATTAACAGACCGAAACTTAAATACATCTTTCTTTGATCCAATCGGAGGGGGAGACCCTA
TT
>MK890423.1
TCGGAACTTCTCTAAGAATTTTAATTCGTGCTGAACTTAGCCACCCTGGTATATTTATTG
GGAATGACCAAATTTATAATGTAATTGTAACAGCTCATGCATTTATTATAATTTTCTTTA
TAGTAATGCCAATTATAATTGGAGGATTTGGAAATTGATTAGTTCCTTTAATATTAGGAG
CCCCTGATATAGCTTTCCCTCGAATGAATAATATAAGTTTTTGAATACTACCTCCTTCAT
TGACTCTTCTATTATCAAGCTCAATAGTAGAAAATGGGGCAGGAACTGGGTGAACAGTTT
ATCCTCCTCTCTCTTCAGGAACAGCTCATGCTGGAGCTTCTGTTGATTTAGCTATTTTTT
CTCTTCATTTAGCTGGAATTTCCTCAATTTTAGGGGCAGTAAATTTTATTACAACTGTGA
TTAATATGCGATCGTCAGGGATTACTTTAGATCGACTACCCTTATTTGTTTGATCTGTAG
TTATTACAGCTATCTTATTACTTCTTTCTCTTCCTGTTTTAGCTGGAGCTATTACTATAT
TATTAACAGACCGAAACTTAAATACATCTTTCTTTGATCCAATCGGAGGGGGAGACCCTA
TT
>MK890424.1
TCGGAACTTCTCTAAGAATTTTAATTCGTGCTGAACTTAGCCACCCTGGTATATTTATTG
GGAATGACCAAATTTATAATGTAATTGTAACAGCTCATGCATTTATTATAATTTTCTTTA
TAGTAATGCCAATTATAATTGGAGGATTTGGAAATTGATTAGTTCCTTTAATATTAGGAG
CCCCTGATATAGCTTTCCCTCGAATGAATAATATAAGTTTTTGAATACTACCTCCTTCAT
TGACTCTTCTATTATCAAGCTCAATAGTAGAAAATGGGGCAGGAACTGGGTGAACAGTTT
ATCCTCCTCTCTCTTCAGGAACAGCTCATGCTGGAGCTTCTGTTGATTTAGCTATTTTTT
CTCTTCATTTAGCTGGAATTTCCTCAATTTTAGGGGCAGTAAATTTTATTACAACTGTGA
TTAATATGCGATCGTCAGGGATTACTTTAGATCGACTACCCTTATTTGTTTGATCTGTAG
TTATTACAGCTATCTTATTACTTCTTTCTCTTCCTGTTTTAGCTGGAGCTATTACTATAT
TATTAACAGACCGAAACTTAAATACATCTTTCTTTGATCCAATCGGAGGGGGAGACCCTA
TT
>MK890425.1
TCGGAACTTCTCTAAGAATTTTAATTCGTGCTGAACTTAGCCACCCTGGTATATTTATTG
GGAATGACCAAATTTATAATGTAATTGTAACAGCTCATGCATTTATTATAATTTTCTTTA
TAGTAATACCAATTATAATTGGAGGATTTGGAAATTGATTAGTTCCTTTAATATTAGGAG
CCCCTGATATAGCCTTTCCTCGAATAAATAATATAAGTTTTTGAATACTACCTCCTTCAT
TGACTCTTCTATTATCAAGCTCAATAGTAGAAAATGGAGCAGGAACTGGGTGAACAGTTT
ATCCTCCTCTCTCTTCAGGAACAGCTCATGCTGGAGCTTCTGTTGATTTAGCTATTTTTT
CTCTTCATTTAGCTGGAATTTCCTCAATTTTAGGGGCAGTAAATTTTATTACAACTGTAA
TTAATATACGATCGTCAGGAATTACTTTAGATCGACTACCCTTATTTGTTTGATCTGTAG
TTATTACAGCTATCTTATTACTTCTTTCTCTTCCTGTTTTAGCTGGAGCTATTACTATGT
TATTAACAGACCGAAACTTAAATACATCTTTCTTTGATCCAATCGGAGGAGGAGATCCTA
TT
>MK890426.1
TCGGAACTTCTCTAAGAATTTTAATTCGTGCTGAACTTAGCCACCCTGGTATATTTATTG
GGAATGACCAAATTTATAATGTAATTGTAACAGCTCATGCATTTATTATAATTTTCTTTA
TAGTAATACCAATTATAATTGGAGGATTTGGAAATTGATTAGTTCCTTTAATATTAGGAG
CCCCTGATATAGCCTTTCCTCGAATAAATAATATAAGTTTTTGAATACTACCTCCTTCAT
TGACTCTTCTATTATCAAGCTCAATAGTAGAAAATGGAGCAGGAACTGGGTGAACAGTTT
ATCCTCCTCTCTCTTCAGGAACAGCTCATGCTGGAGCTTCTGTTGATTTAGCTATTTTTT
CTCTTCATTTAGCTGGAATTTCCTCAATTTTAGGGGCAGTAAATTTTATTACAACTGTAA
TTAATATACGATCGTCAGGAATTACTTTAGATCGACTACCCTTATTTGTTTGATCTGTAG
TTATTACAGCTATCTTATTACTTCTTTCTCTTCCTGTTTTAGCTGGAGCTATTACTATGT
TATTAACAGACCGAAACTTAAATACATCTTTCTTTGATCCAATCGGAGGAGGAGATCCTA
TT
>MK890427.1

TCGGAACTTCTCTAAGAATTTTAATTCGTGCTGAACTTAGCCACCCTGGTATATTTATTG
GGAATGACCAAATTTATAATGTAATTGTAACAGCTCATGCATTTATTATAATTTTCTTTA
TAGTAATACCAATTATAATTGGAGGATTTGGAAATTGATTAGTTCCTTTAATATTAGGAG
CCCCTGATATAGCCTTTCCTCGAATAAATAATATAAGTTTTTGAATACTACCTCCTTCAT
TGACTCTTCTATTATCAAGCTCAATAGTAGAAAATGGAGCAGGAACTGGGTGAACAGTTT
ATCCTCCTCTCTCTTCAGGAACAGCTCATGCTGGAGCTTCTGTTGATTTAGCTATTTTTT
CTCTTCATTTAGCTGGAATTTCCTCAATTTTAGGGGCAGTAAATTTTATTACAACTGTAA
TTAATATACGATCGTCAGGAATTACTTTAGATCGACTACCCTTATTTGTTTGATCTGTAG
TTATTACAGCTATCTTATTACTTCTTTCTCTTCCTGTTTTAGCTGGAGCTATTACTATGT
TATTAACAGACCGAAACTTAAATACATCTTTCTTTGATCCAATCGGAGGAGGAGATCCTA
TT
>MK890428.1
TCGGAACTTCTCTAAGAATTTTAATTCGTGCTGAACTTAGCCACCCTGGTATATTTATTG
GGAATGACCAAATTTATAATGTAATTGTAACAGCTCATGCATTTATTATAATTTTCTTTA
TAGTAATACCAATTATAATTGGAGGATTTGGAAATTGATTAGTTCCTTTAATATTAGGAG
CCCCTGATATAGCCTTTCCTCGAATAAATAATATAAGTTTTTGAATACTACCTCCTTCAT
TGACTCTTCTATTATCAAGCTCAATAGTAGAAAATGGAGCAGGAACTGGGTGAACAGTTT
ATCCTCCTCTCTCTTCAGGAACAGCTCATGCTGGAGCTTCTGTTGATTTAGCTATTTTTT
CTCTTCATTTAGCTGGAATTTCCTCAATTTTAGGGGCAGTAAATTTTATTACAACTGTAA
TTAATATACGATCGTCAGGAATTACTTTAGATCGACTACCCTTATTTGTTTGATCTGTAG
TTATTACAGCTATCTTATTACTTCTTTCTCTTCCTGTTTTAGCTGGAGCTATTACTATGT
TATTAACAGACCGAAACTTAAATACATCTTTCTTTGATCCAATCGGAGGAGGAGATCCTA
TT
>MK890429.1
TCGGAACTTCTCTAAGAATTTTAATTCGTGCTGAACTTAGCCACCCTGGTATATTTATTG
GGAATGACCAAATTTATAATGTAATTGTAACAGCTCATGCATTTATTATAATTTTCTTTA
TAGTAATGCCAATTATAATTGGAGGATTTGGAAATTGATTAGTTCCTTTAATATTAGGAG
CCCCTGATATAGCTTTCCCTCGAATGAATAATATAAGTTTTTGAATACTACCTCCTTCAT
TGACTCTTCTATTATCAAGCTCAATAGTAGAAAATGGGGCAGGAACTGGGTGAACAGTTT
ATCCTCCTCTCTCTTCAGGAACAGCTCATGCTGGAGCTTCTGTTGATTTAGCTATTTTTT
CTCTTCATTTAGCTGGAATTTCCTCAATTTTAGGGGCAGTAAATTTTATTACAACTGTGA
TTAATATGCGATCGTCAGGGATTACTTTAGATCGACTACCCTTATTTGTTTGATCTGTAG
TTATTACAGCTATCTTATTACTTCTTTCTCTTCCTGTTTTAGCTGGAGCTATTACTATAT
TATTAACAGACCGAAACTTAAATACATCTTTCTTTGATCCAATCGGAGGGGGAGACCCTA
TT
>MK890430.1
TCGGAACTTCTCTAAGAATTTTAATTCGTGCTGAACTTAGCCACCCTGGTATATTTATTG
GGAATGACCAAATTTATAATGTAATTGTAACAGCTCATGCATTTATTATAATTTTCTTTA
TAGTAATACCAATTATAATTGGAGGATTTGGAAATTGATTAGTTCCTTTAATATTAGGAG
CCCCTGATATAGCCTTTCCTCGAATAAATAATATAAGTTTTTGAATACTACCTCCTTCAT
TGACTCTTCTATTATCAAGCTCAATAGTAGAAAATGGAGCAGGAACTGGGTGAACAGTTT
ATCCTCCTCTCTCTTCAGGAACAGCTCATGCTGGAGCTTCTGTTGATTTAGCTATTTTTT
CTCTTCATTTAGCTGGAATTTCCTCAATTTTAGGGGCAGTAAATTTTATTACAACTGTAA
TTAATATACGATCGTCAGGAATTACTTTAGATCGACTACCCTTATTTGTTTGATCTGTAG
TTATTACAGCTATCTTATTACTTCTTTCTCTTCCTGTTTTAGCTGGAGCTATTACTATGT
TATTAACAGACCGAAACTTAAATACATCTTTCTTTGATCCAATCGGAGGAGGAGATCCTA
TT
>MK890431.1
TCGGAACTTCTCTAAGAATTTTAATTCGTGCTGAACTTAGCCACCCTGGTATATTTATTG
GGAATGACCAAATTTATAATGTAATTGTAACAGCTCATGCATTTATTATAATTTTCTTTA
TAGTAATACCAATTATAATTGGAGGATTTGGAAATTGATTAGTTCCTTTAATATTAGGAG
CCCCTGATATAGCCTTTCCTCGAATAAATAATATAAGTTTTTGAATACTACCTCCTTCAT
TGACTCTTCTATTATCAAGCTCAATAGTAGAAAATGGAGCAGGAACTGGGTGAACAGTTT
ATCCTCCTCTCTCTTCAGGAACAGCTCATGCTGGAGCTTCTGTTGATTTAGCTATTTTTT
CTCTTCATTTAGCTGGAATTTCCTCAATTTTAGGGGCAGTAAATTTTATTACAACTGTAA
TTAATATACGATCGTCAGGAATTACTTTAGATCGACTACCCTTATTTGTTTGATCTGTAG
TTATTACAGCTATCTTATTACTTCTTTCTCTTCCTGTTTTAGCTGGAGCTATTACTATGT

TATTAACAGACCGAAACTTAAATACATCTTTCTTTGATCCAATCGGAGGAGGAGATCCTA
TT
>MK890432.1
TCGGAACTTCTCTAAGAATTTTAATTCGTGCTGAACTTAGCCACCCTGGTATATTTATTG
GGAATGACCAAATTTATAATGTAATTGTAACAGCTCATGCATTTATTATAATTTTCTTTA
TAGTAATGCCAATTATAATTGGAGGATTTGGAAATTGATTAGTTCCTTTAATATTAGGAG
CCCCTGATATAGCTTTCCCTCGAATGAATAATATAAGTTTTTGAATACTACCTCCTTCAT
TGACTCTTCTATTATCAAGCTCAATAGTAGAAAATGGGGCAGGAACTGGGTGAACAGTTT
ATCCTCCTCTCTCTTCAGGAACAGCTCATGCTGGAGCTTCTGTTGATTTAGCTATTTTTT
CTCTTCATTTAGCTGGAATTTCCTCAATTTTAGGGGCAGTAAATTTTATTACAACTGTGA
TTAATATGCGATCGTCAGGGATTACTTTAGATCGACTACCCTTATTTGTTTGATCTGTAG
TTATTACAGCTATCTTATTACTTCTTTCTCTTCCTGTTTTAGCTGGAGCTATTACTATAT
TATTAACAGACCGAAACTTAAATACATCTTTCTTTGATCCAATCGGAGGGGGAGACCCTA
TT
>MK890433.1
TCGGAACTTCTCTAAGAATTTTAATTCGTGCTGAACTTAGCCACCCTGGTATATTTATTG
GGAATGACCAAATTTATAATGTAATTGTAACAGCTCATGCATTTATTATAATTTTCTTTA
TAGTAATACCAATTATAATTGGAGGATTTGGAAATTGATTAGTTCCTTTAATATTAGGAG
CCCCTGATATAGCCTTTCCTCGAATAAATAATATAAGTTTTTGAATACTACCTCCTTCAT
TGACTCTTCTATTATCAAGCTCAATAGTAGAAAATGGAGCAGGAACTGGGTGAACAGTTT
ATCCTCCTCTCTCTTCAGGAACAGCTCATGCTGGAGCTTCTGTTGATTTAGCTATTTTTT
CTCTTCATTTAGCTGGAATTTCCTCAATTTTAGGGGCAGTAAATTTTATTACAACTGTAA
TTAATATACGATCGTCAGGAATTACTTTAGATCGACTACCCTTATTTGTTTGATCTGTAG
TTATTACAGCTATCTTATTACTTCTTTCTCTTCCTGTTTTAGCTGGAGCTATTACTATGT
TATTAACAGACCGAAACTTAAATACATCTTTCTTTGATCCAATCGGAGGAGGAGATCCTA
TT
>MK890434.1
TCGGAACTTCTCTAAGAATTTTAATTCGTGCTGAACTTAGCCACCCTGGTATATTTATTG
GGAATGACCAAATTTATAATGTAATTGTAACAGCTCATGCATTTATTATAATTTTCTTTA
TAGTAATACCAATTATAATTGGAGGATTTGGAAATTGATTAGTTCCTTTAATATTAGGAG
CCCCTGATATAGCCTTTCCTCGAATAAATAATATAAGTTTTTGAATACTACCTCCTTCAT
TGACTCTTCTATTATCAAGCTCAATAGTAGAAAATGGAGCAGGAACTGGGTGAACAGTTT
ATCCTCCTCTCTCTTCAGGAACAGCTCATGCTGGAGCTTCTGTTGATTTAGCTATTTTTT
CTCTTCATTTAGCTGGAATTTCCTCAATTTTAGGGGCAGTAAATTTTATTACAACTGTAA
TTAATATACGATCGTCAGGAATTACTTTAGATCGACTACCCTTATTTGTTTGATCTGTAG
TTATTACAGCTATCTTATTACTTCTTTCTCTTCCTGTTTTAGCTGGAGCTATTACTATGT
TATTAACAGACCGAAACTTAAATACATCTTTCTTTGATCCAATCGGAGGAGGAGATCCTA
TT
>MK890435.1
TCGGAACTTCTCTAAGAATTTTAATTCGTGCTGAACTTAGCCACCCTGGTATATTTATTG
GGAATGACCAAATTTATAATGTAATTGTAACAGCTCATGCATTTATTATAATTTTCTTTA
TAGTAATACCAATTATAATTGGAGGATTTGGAAATTGATTAGTTCCTTTAATATTAGGAG
CCCCTGATATAGCCTTTCCTCGAATAAATAATATAAGTTTTTGAATACTACCTCCTTCAT
TGACTCTTCTATTATCAAGCTCAATAGTAGAAAATGGAGCAGGAACTGGGTGAACAGTTT
ATCCTCCTCTCTCTTCAGGAACAGCTCATGCTGGAGCTTCTGTTGATTTAGCTATTTTTT
CTCTTCATTTAGCTGGAATTTCCTCAATTTTAGGGGCAGTAAATTTTATTACAACTGTAA
TTAATATACGATCGTCAGGAATTACTTTAGATCGACTACCCTTATTTGTTTGATCTGTAG
TTATTACAGCTATCTTATTACTTCTTTCTCTTCCTGTTTTAGCTGGAGCTATTACTATGT
TATTAACAGACCGAAACTTAAATACATCTTTCTTTGATCCAATCGGAGGAGGAGATCCTA
TT
>MK890436.1
TCGGAACTTCTCTAAGAATTTTAATTCGTGCTGAACTTAGCCACCCTGGTATATTTATTG
GGAATGACCAAATTTATAATGTAATTGTAACAGCTCATGCATTTATTATAATTTTCTTTA
TAGTAATACCAATTATAATTGGAGGATTTGGAAATTGATTAGTTCCTTTAATATTAGGAG
CCCCTGATATAGCCTTTCCTCGAATAAATAATATAAGTTTTTGAATACTACCTCCTTCAT
TGACTCTTCTATTATCAAGCTCAATAGTAGAAAATGGAGCAGGAACTGGGTGAACAGTTT
ATCCTCCTCTCTCTTCAGGAACAGCTCATGCTGGAGCTTCTGTTGATTTAGCTATTTTTT

CTCTTCATTTAGCTGGAATTTCCTCAATTTTAGGGGCAGTAAATTTTATTACAACTGTAA
TTAATATACGATCGTCAGGAATTACTTTAGATCGACTACCCTTATTTGTTTGATCTGTAG
TTATTACAGCTATCTTATTACTTCTTTCTCTTCCTGTTTTAGCTGGAGCTATTACTATGT
TATTAACAGACCGAAACTTAAATACATCTTTCTTTGATCCAATCGGAGGAGGAGATCCTA
TT
>MK890437.1
TCGGAACTTCTCTAAGAATTTTAATTCGTGCTGAACTTAGCCACCCTGGTATATTTATTG
GGAATGACCAAATTTATAATGTAATTGTAACAGCTCATGCATTTATTATAATTTTCTTTA
TAGTAATACCAATTATAATTGGAGGATTTGGAAATTGATTAGTTCCTTTAATATTAGGAG
CCCCTGATATAGCCTTTCCTCGAATAAATAATATAAGTTTTTGAATACTACCTCCTTCAT
TGACTCTTCTATTATCAAGCTCAATAGTAGAAAATGGAGCAGGAACTGGGTGAACAGTTT
ATCCTCCTCTCTCTTCAGGAACAGCTCATGCTGGAGCTTCTGTTGATTTAGCTATTTTTT
CTCTTCATTTAGCTGGAATTTCCTCAATTTTAGGGGCAGTAAATTTTATTACAACTGTAA
TTAATATACGATCGTCAGGAATTACTTTAGATCGACTACCCTTATTTGTTTGATCTGTAG
TTATTACAGCTATCTTATTACTTCTTTCTCTTCCTGTTTTAGCTGGAGCTATTACTATGT
TATTAACAGACCGAAACTTAAATACATCTTTCTTTGATCCAATCGGAGGAGGAGATCCTA
TT
>MK890438.1
TCGGAACTTCTCTAAGAATTTTAATTCGTGCTGAACTTAGCCACCCTGGTATATTTATTG
GGAATGACCAAATTTATAATGTAATTGTAACAGCTCATGCATTTATTATAATTTTCTTTA
TAGTAATACCAATTATAATTGGAGGATTTGGAAATTGATTAGTTCCTTTAATATTAGGAG
CCCCTGATATAGCCTTTCCTCGAATAAATAATATAAGTTTTTGAATACTACCTCCTTCAT
TGACTCTTCTATTATCAAGCTCAATAGTAGAAAATGGAGCAGGAACTGGGTGAACAGTTT
ATCCTCCTCTCTCTTCAGGAACAGCTCATGCTGGAGCTTCTGTTGATTTAGCTATTTTTT
CTCTTCATTTAGCTGGAATTTCCTCAATTTTAGGGGCAGTAAATTTTATTACAACTGTAA
TTAATATACGATCGTCAGGAATTACTTTAGATCGACTACCCTTATTTGTTTGATCTGTAG
TTATTACAGCTATCTTATTACTTCTTTCTCTTCCTGTTTTAGCTGGAGCTATTACTATGT
TATTAACAGACCGAAACTTAAATACATCTTTCTTTGATCCAATCGGAGGAGGAGATCCTA
TT
>MK890439.1
TCGGAACTTCTCTAAGAATTTTAATTCGTGCTGAACTTAGCCACCCTGGTATATTTATTG
GGAATGACCAAATTTATAATGTAATTGTAACAGCTCATGCATTTATTATAATTTTCTTTA
TAGTAATACCAATTATAATTGGAGGATTTGGAAATTGATTAGTTCCTTTAATATTAGGAG
CCCCTGATATAGCCTTTCCTCGAATAAATAATATAAGTTTTTGAATACTACCTCCTTCAT
TGACTCTTCTATTATCAAGCTCAATAGTAGAAAATGGAGCAGGAACTGGGTGAACAGTTT
ATCCTCCTCTCTCTTCAGGAACAGCTCATGCTGGAGCTTCTGTTGATTTAGCTATTTTTT
CTCTTCATTTAGCTGGAATTTCCTCAATTTTAGGGGCAGTAAATTTTATTACAACTGTAA
TTAATATACGATCGTCAGGAATTACTTTAGATCGACTACCCTTATTTGTTTGATCTGTAG
TTATTACAGCTATCTTATTACTTCTTTCTCTTCCTGTTTTAGCTGGAGCTATTACTATGT
TATTAACAGACCGAAACTTAAATACATCTTTCTTTGATCCAATCGGAGGAGGAGATCCTA
TT
>MK890440.1
TCGGAACTTCTCTAAGAATTTTAATTCGTGCTGAACTTAGCCACCCTGGTATATTTATTG
GGAATGACCAAATTTATAATGTAATTGTAACAGCTCATGCATTTATTATAATTTTCTTTA
TAGTAATGCCAATTATAATTGGAGGATTTGGAAATTGATTAGTTCCTTTAATATTAGGAG
CCCCTGATATAGCTTTCCCTCGAATGAATAATATAAGTTTTTGAATACTACCTCCTTCAT
TGACTCTTCTATTATCAAGCTCAATAGTAGAAAATGGGGCAGGAACTGGGTGAACAGTTT
ATCCTCCTCTCTCTTCAGGAACAGCTCATGCTGGAGCTTCTGTTGATTTAGCTATTTTTT
CTCTTCATTTAGCTGGAATTTCCTCAATTTTAGGGGCAGTAAATTTTATTACAACTGTGA
TTAATATGCGATCGTCAGGGATTACTTTAGATCGACTACCCTTATTTGTTTGATCTGTAG
TTATTACAGCTATCTTATTACTTCTTTCTCTTCCTGTTTTAGCTGGAGCTATTACTATAT
TATTAACAGACCGAAACTTAAATACATCTTTCTTTGATCCAATCGGAGGGGGAGACCCTA
TT
>MK890441.1
TCGGAACTTCTCTAAGAATTTTAATTCGTGCTGAACTTAGCCACCCTGGTATATTTATTG
GGAATGACCAAATTTATAATGTAATTGTAACAGCTCATGCATTTATTATAATTTTCTTTA
TAGTAATGCCAATTATAATTGGAGGATTTGGAAATTGATTAGTTCCTTTAATATTAGGAG

CCCCTGATATAGCTTTCCCTCGAATGAATAATATAAGTTTTTGAATACTACCTCCTTCAT
TGACTCTTCTATTATCAAGCTCAATAGTAGAAAATGGGGCAGGAACTGGGTGAACAGTTT
ATCCTCCTCTCTCTTCAGGAACAGCTCATGCTGGAGCTTCTGTTGATTTAGCTATTTTTT
CTCTTCATTTAGCTGGAATTTCCTCAATTTTAGGGGCAGTAAATTTTATTACAACTGTGA
TTAATATGCGATCGTCAGGGATTACTTTAGATCGACTACCCTTATTTGTTTGATCTGTAG
TTATTACAGCTATCTTATTACTTCTTTCTCTTCCTGTTTTAGCTGGAGCTATTACTATAT
TATTAACAGACCGAAACTTAAATACATCTTTCTTTGATCCAATCGGAGGGGGAGACCCTA
TT
>MK890442.1
TCGGAACTTCTCTAAGAATTTTAATTCGTGCTGAACTTAGCCACCCTGGTATATTTATTG
GGAATGACCAAATTTATAATGTAATTGTAACAGCTCATGCATTTATTATAATTTTCTTTA
TAGTAATGCCAATTATAATTGGAGGATTTGGAAATTGATTAGTTCCTTTAATATTAGGAG
CCCCTGATATAGCTTTCCCTCGAATGAATAATATAAGTTTTTGAATACTACCTCCTTCAT
TGACTCTTCTATTATCAAGCTCAATAGTAGAAAATGGGGCAGGAACTGGGTGAACAGTTT
ATCCTCCTCTCTCTTCAGGAACAGCTCATGCTGGAGCTTCTGTTGATTTAGCTATTTTTT
CTCTTCATTTAGCTGGAATTTCCTCAATTTTAGGGGCAGTAAATTTTATTACAACTGTGA
TTAATATGCGATCGTCAGGGATTACTTTAGATCGACTACCCTTATTTGTTTGATCTGTAG
TTATTACAGCTATCTTATTACTTCTTTCTCTTCCTGTTTTAGCTGGAGCTATTACTATAT
TATTAACAGACCGAAACTTAAATACATCTTTCTTTGATCCAATCGGAGGGGGAGACCCTA
TT
>MK890443.1
TCGGAACTTCTCTAAGAATTTTAATTCGTGCTGAACTTAGCCACCCTGGTATATTTATTG
GGAATGACCAAATTTATAATGTAATTGTAACAGCTCATGCATTTATTATAATTTTCTTTA
TAGTAATACCAATTATAATTGGAGGATTTGGAAATTGATTAGTTCCTTTAATATTAGGAG
CCCCTGATATAGCCTTTCCTCGAATAAATAATATAAGTTTTTGAATACTACCTCCTTCAT
TGACTCTTCTATTATCAAGCTCAATAGTAGAAAATGGAGCAGGAACTGGGTGAACAGTTT
ATCCTCCTCTCTCTTCAGGAACAGCTCATGCTGGAGCTTCTGTTGATTTAGCTATTTTTT
CTCTTCATTTAGCTGGAATTTCCTCAATTTTAGGGGCAGTAAATTTTATTACAACTGTAA
TTAATATACGATCGTCAGGAATTACTTTAGATCGACTACCCTTATTTGTTTGATCTGTAG
TTATTACAGCTATCTTATTACTTCTTTCTCTTCCTGTTTTAGCTGGAGCTATTACTATGT
TATTAACAGACCGAAACTTAAATACATCTTTCTTTGATCCAATCGGAGGAGGAGATCCTA
TT
>MK890444.1
TCGGAACTTCTCTAAGAATTTTAATTCGTGCTGAACTTAGCCACCCTGGTATATTTATTG
GGAATGACCAAATTTATAATGTAATTGTAACAGCTCATGCATTTATTATAATTTTCTTTA
TAGTAATGCCAATTATAATTGGAGGATTTGGAAATTGATTAGTTCCTTTAATATTAGGAG
CCCCTGATATAGCTTTCCCTCGAATGAATAATATAAGTTTTTGAATACTACCTCCTTCAT
TGACTCTTCTATTATCAAGCTCAATAGTAGAAAATGGGGCAGGAACTGGGTGAACAGTTT
ATCCTCCTCTCTCTTCAGGAACAGCTCATGCTGGAGCTTCTGTTGATTTAGCTATTTTTT
CTCTTCATTTAGCTGGAATTTCCTCAATTTTAGGGGCAGTAAATTTTATTACAACTGTGA
TTAATATGCGATCGTCAGGGATTACTTTAGATCGACTACCCTTATTTGTTTGATCTGTAG
TTATTACAGCTATCTTATTACTTCTTTCTCTTCCTGTTTTAGCTGGAGCTATTACTATAT
TATTAACAGACCGAAACTTAAATACATCTTTCTTTGATCCAATCGGAGGGGGAGACCCTA
TT
>MK890445.1
TCGGAACTTCTCTAAGAATTTTAATTCGTGCTGAACTTAGCCACCCTGGTATATTTATTG
GGAATGACCAAATTTATAATGTAATTGTAACAGCTCATGCATTTATTATAATTTTCTTTA
TAGTAATGCCAATTATAATTGGAGGATTTGGAAATTGATTAGTTCCTTTAATATTAGGAG
CCCCTGATATAGCTTTCCCTCGAATGAATAATATAAGTTTTTGAATACTACCTCCTTCAT
TGACTCTTCTATTATCAAGCTCAATAGTAGAAAATGGGGCAGGAACTGGGTGAACAGTTT
ATCCTCCTCTCTCTTCAGGAACAGCTCATGCTGGAGCTTCTGTTGATTTAGCTATTTTTT
CTCTTCATTTAGCTGGAATTTCCTCAATTTTAGGGGCAGTAAATTTTATTACAACTGTGA
TTAATATGCGATCGTCAGGGATTACTTTAGATCGACTACCCTTATTTGTTTGATCTGTAG
TTATTACAGCTATCTTATTACTTCTTTCTCTTCCTGTTTTAGCTGGAGCTATTACTATAT
TATTAACAGACCGAAACTTAAATACATCTTTCTTTGATCCAATCGGAGGGGGAGACCCTA
TT
>MK890446.1

TCGGAACTTCTCTAAGAATTTTAATTCGTGCTGAACTTAGCCACCCTGGTATATTTATTG
GGAATGACCAAATTTATAATGTAATTGTAACAGCTCATGCATTTATTATAATTTTCTTTA
TAGTAATACCAATTATAATTGGAGGATTTGGAAATTGATTAGTTCCTTTAATATTAGGAG
CCCCTGATATAGCCTTTCCTCGAATAAATAATATAAGTTTTTGAATACTACCTCCTTCAT
TGACTCTTCTATTATCAAGCTCAATAGTAGAAAATGGAGCAGGAACTGGGTGAACAGTTT
ATCCTCCTCTCTCTTCAGGAACAGCTCATGCTGGAGCTTCTGTTGATTTAGCTATTTTTT
CTCTTCATTTAGCTGGAATTTCCTCAATTTTAGGGGCAGTAAATTTTATTACAACTGTAA
TTAATATACGATCGTCAGGAATTACTTTAGATCGACTACCCTTATTTGTTTGATCTGTAG
TTATTACAGCTATCTTATTACTTCTTTCTCTTCCTGTTTTAGCTGGAGCTATTACTATGT
TATTAACAGACCGAAACTTAAATACATCTTTCTTTGATCCAATCGGAGGAGGAGATCCTA
TT
>MK890447.1
TCGGAACTTCTCTAAGAATTTTAATTCGTGCTGAACTTAGCCACCCTGGTATATTTATTG
GGAATGACCAAATTTATAATGTAATTGTAACAGCTCATGCATTTATTATAATTTTCTTTA
TAGTAATACCAATTATAATTGGAGGATTTGGAAATTGATTAGTTCCTTTAATATTAGGAG
CCCCTGATATAGCCTTTCCTCGAATAAATAATATAAGTTTTTGAATACTACCTCCTTCAT
TGACTCTTCTATTATCAAGCTCAATAGTAGAAAATGGAGCAGGAACTGGGTGAACAGTTT
ATCCTCCTCTCTCTTCAGGAACAGCTCATGCTGGAGCTTCTGTTGATTTAGCTATTTTTT
CTCTTCATTTAGCTGGAATTTCCTCAATTTTAGGGGCAGTAAATTTTATTACAACTGTAA
TTAATATACGATCGTCAGGAATTACTTTAGATCGACTACCCTTATTTGTTTGATCTGTAG
TTATTACAGCTATCTTATTACTTCTTTCTCTTCCTGTTTTAGCTGGAGCTATTACTATGT
TATTAACAGACCGAAACTTAAATACATCTTTCTTTGATCCAATCGGAGGAGGAGATCCTA
TT
>MK890448.1
TCGGAACTTCTCTAAGAATTTTAATTCGTGCTGAACTTAGCCACCCTGGTATATTTATTG
GGAATGACCAAATTTATAATGTAATTGTAACAGCTCATGCATTTATTATAATTTTCTTTA
TAGTAATGCCAATTATAATTGGAGGATTTGGAAATTGATTAGTTCCTTTAATATTAGGAG
CCCCTGATATAGCTTTCCCTCGAATGAATAATATAAGTTTTTGAATACTACCTCCTTCAT
TGACTCTTCTATTATCAAGCTCAATAGTAGAAAATGGGGCAGGAACTGGGTGAACAGTTT
ATCCTCCTCTCTCTTCAGGAACAGCTCATGCTGGAGCTTCTGTTGATTTAGCTATTTTTT
CTCTTCATTTAGCTGGAATTTCCTCAATTTTAGGGGCAGTAAATTTTATTACAACTGTGA
TTAATATGCGATCGTCAGGGATTACTTTAGATCGACTACCCTTATTTGTTTGATCTGTAG
TTATTACAGCTATCTTATTACTTCTTTCTCTTCCTGTTTTAGCTGGAGCTATTACTATAT
TATTAACAGACCGAAACTTAAATACATCTTTCTTTGATCCAATCGGAGGGGGAGACCCTA
TT
>MK890449.1
TCGGAACTTCTCTAAGAATTTTAATTCGTGCTGAACTTAGCCACCCTGGTATATTTATTG
GGAATGACCAAATTTATAATGTAATTGTAACAGCTCATGCATTTATTATAATTTTCTTTA
TAGTAATACCAATTATAATTGGAGGATTTGGAAATTGATTAGTTCCTTTAATATTAGGAG
CCCCTGATATAGCCTTTCCTCGAATAAATAATATAAGTTTTTGAATACTACCTCCTTCAT
TGACTCTTCTATTATCAAGCTCAATAGTAGAAAATGGAGCAGGAACTGGGTGAACAGTTT
ATCCTCCTCTCTCTTCAGGAACAGCTCATGCTGGAGCTTCTGTTGATTTAGCTATTTTTT
CTCTTCATTTAGCTGGAATTTCCTCAATTTTAGGGGCAGTAAATTTTATTACAACTGTAA
TTAATATACGATCGTCAGGAATTACTTTAGATCGACTACCCTTATTTGTTTGATCTGTAG
TTATTACAGCTATCTTATTACTTCTTTCTCTTCCTGTTTTAGCTGGAGCTATTACTATGT
TATTAACAGACCGAAACTTAAATACATCTTTCTTTGATCCAATCGGAGGAGGAGATCCTA
TT
>MK890450.1
TCGGAACTTCTCTAAGAATTTTAATTCGTGCTGAACTTAGCCACCCTGGTATATTTATTG
GGAATGACCAAATTTATAATGTAATTGTAACAGCTCATGCATTTATTATAATTTTCTTTA
TAGTAATACCAATTATAATTGGAGGATTTGGAAATTGATTAGTTCCTTTAATATTAGGAG
CCCCTGATATAGCCTTTCCTCGAATAAATAATATAAGTTTTTGAATACTACCTCCTTCAT
TGACTCTTCTATTATCAAGCTCAATAGTAGAAAATGGAGCAGGAACTGGGTGAACAGTTT
ATCCTCCTCTCTCTTCAGGAACAGCTCATGCTGGAGCTTCTGTTGATTTAGCTATTTTTT
CTCTTCATTTAGCTGGAATTTCCTCAATTTTAGGGGCAGTAAATTTTATTACAACTGTAA
TTAATATACGATCGTCAGGAATTACTTTAGATCGACTACCCTTATTTGTTTGATCTGTAG
TTATTACAGCTATCTTATTACTTCTTTCTCTTCCTGTTTTAGCTGGAGCTATTACTATGT

TATTAACAGACCGAAACTTAAATACATCTTTCTTTGATCCAATCGGAGGAGGAGATCCTA
TT
>MK890451.1
TCGGAACTTCTCTAAGAATTTTAATTCGTGCTGAACTTAGCCACCCTGGTATATTTATTG
GGAATGACCAAATTTATAATGTAATTGTAACAGCTCATGCATTTATTATAATTTTCTTTA
TAGTAATACCAATTATAATTGGAGGATTTGGAAATTGATTAGTTCCTTTAATATTAGGAG
CCCCTGATATAGCCTTTCCTCGAATAAATAATATAAGTTTTTGAATACTACCTCCTTCAT
TGACTCTTCTATTATCAAGCTCAATAGTAGAAAATGGAGCAGGAACTGGGTGAACAGTTT
ATCCTCCTCTCTCTTCAGGAACAGCTCATGCTGGAGCTTCTGTTGATTTAGCTATTTTTT
CTCTTCATTTAGCTGGAATTTCCTCAATTTTAGGGGCAGTAAATTTTATTACAACTGTAA
TTAATATACGATCGTCAGGAATTACTTTAGATCGACTACCCTTATTTGTTTGATCTGTAG
TTATTACAGCTATCTTATTACTTCTTTCTCTTCCTGTTTTAGCTGGAGCTATTACTATGT
TATTAACAGACCGAAACTTAAATACATCTTTCTTTGATCCAATCGGAGGAGGAGATCCTA
TT
>MK890452.1
TCGGAACTTCTCTAAGAATTTTAATTCGTGCTGAACTTAGCCACCCTGGTATATTTATTG
GGAATGACCAAATTTATAATGTAATTGTAACAGCTCATGCATTTATTATAATTTTCTTTA
TAGTAATACCAATTATAATTGGAGGATTTGGAAATTGATTAGTTCCTTTAATATTAGGAG
CCCCTGATATAGCCTTTCCTCGAATAAATAATATAAGTTTTTGAATACTACCTCCTTCAT
TGACTCTTCTATTATCAAGCTCAATAGTAGAAAATGGAGCAGGAACTGGGTGAACAGTTT
ATCCTCCTCTCTCTTCAGGAACAGCTCATGCTGGAGCTTCTGTTGATTTAGCTATTTTTT
CTCTTCATTTAGCTGGAATTTCCTCAATTTTAGGGGCAGTAAATTTTATTACAACTGTAA
TTAATATACGATCGTCAGGAATTACTTTAGATCGACTACCCTTATTTGTTTGATCTGTAG
TTATTACAGCTATCTTATTACTTCTTTCTCTTCCTGTTTTAGCTGGAGCTATTACTATGT
TATTAACAGACCGAAACTTAAATACATCTTTCTTTGATCCAATCGGAGGAGGAGATCCTA
TT
>MK890453.1
TCGGAACTTCTCTAAGAATTTTAATTCGTGCTGAACTTAGCCACCCTGGTATATTTATTG
GGAATGACCAAATTTATAATGTAATTGTAACAGCTCATGCATTTATTATAATTTTCTTTA
TAGTAATGCCAATTATAATTGGAGGATTTGGAAATTGATTAGTTCCTTTAATATTAGGAG
CCCCTGATATAGCTTTCCCTCGAATGAATAATATAAGTTTTTGAATACTACCTCCTTCAT
TGACTCTTCTATTATCAAGCTCAATAGTAGAAAATGGGGCAGGAACTGGGTGAACAGTTT
ATCCTCCTCTCTCTTCAGGAACAGCTCATGCTGGAGCTTCTGTTGATTTAGCTATTTTTT
CTCTTCATTTAGCTGGAATTTCCTCAATTTTAGGGGCAGTAAATTTTATTACAACTGTGA
TTAATATGCGATCGTCAGGGATTACTTTAGATCGACTACCCTTATTTGTTTGATCTGTAG
TTATTACAGCTATCTTATTACTTCTTTCTCTTCCTGTTTTAGCTGGAGCTATTACTATAT
TATTAACAGACCGAAACTTAAATACATCTTTCTTTGATCCAATCGGAGGGGGAGACCCTA
TT
>MK028219.1
-----------CTAAGAATTTTAATTCGTGCTGAACTTAGCCACCCTGGTATATTTATTG
GGAATGACCAAATTTATAATGTAATTGTAACAGCTCATGCATTTATTATAATTTTCTTTA
TAGTAATACCAATTATAATTGGAGGATTTGGAAATTGATTAGTTCCTTTAATATTAGGAG
CCCCTGATATAGCCTTTCCTCGAATAAATAATATAAGTTTTTGAATACTACCTCCTTCAT
TGACTCTTCTATTATCAAGCTCAATAGTAGAAAATGGGGCAGGAACTGGGTGAACAGTTT
ATCCTCCTCTCTCTTCAGGAACAGCTCATGCTGGAGCTTCTGTTGATTTAGCTATTTTTT
CTCTTCATTTAGCTGGAATTTCCTCAATTTTAGGGGCAGTAAATTTTATTACAACTGTAA
TTAATATACGATCGTCAGGAATTACTTTAGATCGACTACCTTTATTTGTTTGATCTGTAG
TTATTACAGCTATCTTATTACTTCTTTCTCTTCCTGTTTTAGCTGGAGCTATTACTATGT
TATTA---
--
>MK028220.1
-----------------TTTTAATTCGTGCTGAACTTAGCCACCCTGGTATATTTATTG
GGAATGACCAAATTTATAATGTAATTGTAACAGCTCATGCATTTATTATAATTTTCTTTA
TAGTAATACCAATTATAATTGGAGGATTTGGAAATTGATTAGTTCCTTTAATATTAGGAG
CCCCTGATATAGCCTTTCCTCGAATAAATAATATAAGTTTTTGAATACTACCTCCTTCAT
TGACTCTTCTATTATCAAGCTCAATAGTAGAAAATGGGGCAGGAACTGGGTGAACAGTTT
ATCCTCCTCTCTCTTCAGGAACAGCTCATGCTGGAGCTTCTGTTGATTTAGCTATTTTTT

CTCTTCATTTAGCTGGAATTTCCTCAATTTTAGGGGCAGTAAATTTTATTACAACTGTAA
TTAATATACGATCGTCAGGAATTACTTTAGATCGACTACCTTTATTTGTTTGATCTGTAG
TTATTACAGCTATCTTATTACTTCTTTCTCTTCCTGTTTTAGCTGGAGCTATTACTATGT
TATTAACAGACCGAAACTTAAATACAT---------------------------------
--
>MK028221.1
------------------TTTTAATTCGTGCTGAACTTAGCCACCCTGGTATATTTATTG
GGAATGACCAAATTTATAATGTAATTGTAACAGCTCATGCATTTATTATAATTTTCTTTA
TAGTAATACCAATTATAATTGGAGGATTTGGAAATTGATTAGTTCCTTTAATATTAGGAG
CCCCTGATATAGCCTTTCCTCGAATAAATAATATAAGTTTTTGAATACTACCTCCTTCAT
TGACTCTTCTATTATCAAGCTCAATAGTAGAAAATGGGGCAGGAACTGGGTGAACAGTTT
ATCCTCCTCTCTCTTCAGGAACAGCTCATGCTGGAGCTTCTGTTGATTTAGCTATTTTTT
CTCTTCATTTAGCTGGAATTTCCTCAATTTTAGGGGCAGTAAATTTTATTACAACTGTAA
TTAATATACGATCGTCAGGAATTACTTTAGATCGACTACCCTTATTTGTTTGATCTGTAG
TTATTACAGCTATCTTATTACTTCTTTCTCTTCCTGTTTTAGCTGGAGCTATTACTATGT
TATTA---
--
>MK028222.1
----------------------------TGCTGAACTTAGCCACCCTGGTATATTTATTG
GGAATGACCAAATTTATAATGTAATTGTAACAGCTCATGCATTTATTATAATTTTCTTTA
TAGTAATACCAATTATAATTGGAGGATTTGGAAATTGATTAGTTCCTTTAATATTAGGAG
CCCCTGATATAGCCTTTCCTCGAATAAATAATATAAGTTTTTGAATACTACCTCCTTCAT
TGACTCTTCTATTATCAAGCTCAATAGTAGAAAATGGGGCAGGAACTGGGTGAACAGTTT
ATCCTCCTCTCTCTTCAGGAACAGCTCATGCTGGAGCTTCTGTTGATTTAGCTATTTTTT
CTCTTCATTTAGCTGGAATTTCCTCAATTTTAGGGGCAGTAAATTTTATTACAACTGTAA
TTAATATACGATCGTCAGGAATTACTTTAGATCGACTACCTTTATTTGTTTGATCTGTAG
TTATTACAGCTATCTTATTACTTCTTTCTCTTCCTGTTTTAGCTGGAGCTATTACTATGT
TATTAA--
--
>MK028223.1
-------------------TTTAATTCGTGCTGAACTTAGCCACCCTGGTATATTTATTG
GGAATGACCAAATTTATAATGTAATTGTAACAGCTCATGCATTTATTATAATTTTCTTTA
TAGTAATACCAATTATAATTGGAGGATTTGGAAATTGATTAGTTCCTTTAATATTAGGAG
CCCCTGATATAGCCTTTCCTCGAATAAATAATATAAGTTTTTGAATACTACCTCCTTCAT
TGACTCTTCTATTATCAAGCTCAATAGTAGAAAATGGGGCAGGAACTGGGTGAACAGTTT
ATCCTCCTCTCTCTTCAGGAACAGCTCATGCTGGAGCTTCTGTTGATTTAGCTATTTTTT
CTCTTCATTTAGCTGGAATTTCCTCAATTTTAGGGGCAGTAAATTTTATTACAACTGTAA
TTAATATACGATCGTCAGGAATTACTTTAGATCGACTACCTTTATTTGTTTGATCTGTAG
TTATTACAGCTATCTTATTACTTCTTTCTCTTCCTGTTTTAGCTGGAGCTATTACTATGT
TATTAACAGACCGAAACTTAAATAC-----------------------------------
--
>MK028224.1
-------TTCTCTAAGAATTTTAATTCGTGCTGAACTTAGCCACCCTGGTATATTTATTG
GGAATGACCAAATTTATAATGTAATTGTAACAGCTCATGCATTTATTATAATTTTCTTTA
TAGTAATACCAATTATAATTGGAGGATTTGGAAATTGATTAGTTCCTTTAATATTAGGAG
CCCCTGATATAGCCTTTCCTCGAATAAATAATATAAGTTTTTGAATACTACCTCCTTCAT
TGACTCTTCTATTATCAAGCTCAATAGTAGAAAATGGGGCAGGAACTGGGTGAACAGTTT
ATCCTCCTCTCTCTTCAGGAACAGCTCATGCTGGAGCTTCTGTTGATTTAGCTATTTTTT
CTCTTCATTTAGCTGGAATTTCCTCAATTTTAGGGGCAGTAAATTTTATTACAACTGTAA
TTAATATACGATCGTCAGGAATTACTTTAGATCGACTACCTTTATTTGTTTGATCTGTAG
TTATTACAGCTATCTTATTACTTCTTTCTCTTCCTGTTTTAGCTGGAGCTATTACTATGT
TATTAACAGACCGAAACTTAAATACATCTTT-----------------------------
--
>MK028225.1
--------------------------CGTGCTGAACTTAGCCACCCTGGTATATTTATTG
GGAATGACCAAATTTATAATGTAATTGTAACAGCTCATGCATTTATTATAATTTTCTTTA
TAGTAATACCAATTATAATTGGAGGATTTGGAAATTGATTAGTTCCTTTAATATTAGGAG

CCCCTGATATAGCCTTTCCTCGAATAAATAATATAAGTTTTTGAATACTACCTCCTTCAT
TGACTCTTCTATTATCAAGCTCAATAGTAGAAAATGGGGCAGGAACTGGGTGAACAGTTT
ATCCTCCTCTCTCTTCAGGAACAGCTCATGCTGGAGCTTCTGTTGATTTAGCTATTTTTT
CTCTTCATTTAGCTGGAATTTCCTCAATTTTAGGGGCAGTAAATTTTATTACAACTGTAA
TTAATATACGATCGTCAGGAATTACTTTAGATCGACTACCTTTATTTGTTTGATCTGTAG
TTATTACAGCTATCTTATTACTTCTTTCTCTTCCTGTTTTAGCTGGAGCTATTACTATGT
TATTAAC---
--
>MK028226.1
------------------TTTTAATTCGTGCTGAACTTAGCCACCCTGGTATATTTATTG
GGAATGACCAAATTTATAATGTAATTGTAACAGCTCATGCATTTATTATAATTTTCTTTA
TAGTAATACCAATTATAATTGGAGGATTTGGAAATTGATTAGTTCCTTTAATATTAGGAG
CCCCTGATATAGCCTTTCCTCGAATAAATAATATAAGTTTTTGAATACTACCTCCTTCAT
TGACTCTTCTATTATCAAGCTCAATAGTAGAAAATGGGGCAGGAACTGGGTGAACAGTTT
ATCCTCCTCTCTCTTCAGGAACAGCTCATGCTGGAGCTTCTGTTGATTTAGCTATTTTTT
CTCTTCATTTAGCTGGAATTTCCTCAATTTTAGGGGCAGTAAATTTTATTACAACTGTAA
TTAATATACGATCGTCAGGAATTACTTTAGATCGACTACCTTTATTTGTTTGATCTGTAG
TTATTACAGCTATCTTATTACTTCTTTCTCTTCCTGTTTTAGCTGGAGCTATTACTATGT
TATTAAC---
--
>MK028227.1
--------------------------CGTGCTGAACTTAGCCACCCTGGTATATTTATTG
GGAATGACCAAATTTATAATGTAATTGTAACAGCTCATGCATTTATTATAATTTTCTTTA
TAGTAATGCCAATTATAATTGGAGGATTTGGAAATTGATTAGTTCCTTTAATATTAGGAG
CCCCTGATATAGCTTTCCCTCGAATGAATAATATAAGTTTTTGAATACTACCTCCTTCAT
TGACTCTTCTATTATCAAGCTCAATAGTAGAAAATGGAGCAGGAACTGGATGAACAGTTT
ATCCTCCTCTTTCTTCAGGAACAGCTCATGCTGGAGCTTCTGTTGATTTAGCTATTTTTT
CTCTTCATTTAGCTGGAATTTCCTCAATTTTAGGGGCAGTAAATTTTATTACAACTGTAA
TTAATATACGATCGTCAGGGATTACTTTAGATCGACTACCCTTATTTGTTTGATCTGTAG
TTATTACAGCTATCTTATTACTTCTTTCTCTTCCTGTTTTAGCTGGAGCTATTACTATAT
TATT--
--
>MK028228.1
------------------TTTTAATTCGTGCTGAACTTAGCCACCCTGGTATATTTATTG
GGAATGACCAAATTTATAATGTAATTGTAACAGCTCATGCATTTATTATAATTTTCTTTA
TAGTAATGCCAATTATAATTGGAGGATTTGGAAATTGATTAGTTCCTTTAATATTAGGAG
CCCCTGATATAGCTTTCCCTCGAATGAATAATATAAGTTTTTGAATACTACCTCCTTCAT
TGACTCTTCTATTATCAAGCTCAATAGTAGAAAATGGAGCAGGAACTGGATGAACAGTTT
ATCCTCCTCTTTCTTCAGGAACAGCTCATGCTGGAGCTTCTGTTGATTTAGCTATTTTTT
CTCTTCATTTAGCTGGAATTTCCTCAATTTTAGGGGCAGTAAATTTTATTACAACTGTAA
TTAATATACGATCGTCAGGGATTACTTTAGATCGACTACCCTTATTTGTTTGATCTGTAG
TTATTACAGCTATCTTATTACTTCTTTCTCTTCCTGTTTTAGCTGGAGCTATTACTATAT
TATTAACAGACCGAAACTTAAATAC-----------------------------------
--
>MK028229.1
------------------TTTTAATTCGTGCTGAACTTAGCCACCCTGGTATATTTATTG
GGAATGACCAAATTTATAATGTAATTGTAACAGCTCATGCATTTATTATAATTTTCTTTA
TAGTAATACCAATTATAATTGGAGGATTTGGAAATTGATTAGTTCCTTTAATATTAGGAG
CCCCTGATATAGCCTTTCCTCGAATAAATAATATAAGTTTTTGAATACTACCTCCTTCAT
TGACTCTTCTATTATCAAGCTCAATAGTAGAAAATGGGGCAGGAACTGGGTGAACAGTTT
ATCCTCCTCTCTCTTCAGGAACAGCTCATGCTGGAGCTTCTGTTGATTTAGCTATTTTTT
CTCTTCATTTAGCTGGAATTTCCTCAATTTTAGGGGCAGTAAATTTTATTACAACTGTAA
TTAATATACGATCGTCAGGAATTACTTTAGATCGACTACCTTTATTTGTTTGATCTGTAG
TTATTACAGCTATCTTATTACTTCTTTCTCTTCCTGTTTTAGCTGGAGCTATTACTATGT
TATTAACAGACCGAAACTTAAATAC-----------------------------------
--
>MK028230.1

------------------TTTTAATTCGTGCTGAACTTAGCCACCCTGGTATATTTATTG
GGAATGACCAAATTTATAATGTAATTGTAACAGCTCATGCATTTATTATAATTTTCTTTA
TAGTAATGCCAATTATAATTGGAGGATTTGGAAATTGATTAGTTCCTTTAATATTAGGAG
CCCCTGATATAGCTTTCCCTCGAATGAATAATATAAGTTTTTGAATACTACCTCCTTCAT
TGACTCTTCTATTATCAAGCTCAATAGTAGAAAATGGAGCAGGAACTGGATGAACAGTTT
ATCCTCCTCTTTCTTCAGGAACAGCTCATGCTGGAGCTTCTGTTGATTTAGCTATTTTTT
CTCTTCATTTAGCTGGAATTTCCTCAATTTTAGGGGCAGTAAATTTTATTACAACTGTAA
TTAATATACGATCGTCAGGGATTACTTTAGATCGACTACCCTTATTTGTTTGATCTGTAG
TTATTACAGCTATCTTATTACTTCTTTCTCTTCCTGTTTTAGCTGGAGCTATTACTATAT
TATTAACAGACCGAAACTTAAATAC-----------------------------------
--
>MK028231.1
------------------TTTTAATTCGTGCTGAACTTAGCCACCCTGGTATATTTATTG
GGAATGACCAAATTTATAATGTAATTGTAACAGCTCATGCATTTATTATAATTTTCTTTA
TAGTAATACCAATTATAATTGGAGGATTTGGAAATTGATTAGTTCCTTTAATATTAGGAG
CCCCTGATATAGCCTTTCCTCGAATAAATAATATAAGTTTTTGAATACTACCTCCTTCAT
TGACTCTTCTATTATCAAGCTCAATAGTAGAAAATGGGGCAGGAACTGGGTGAACAGTTT
ATCCTCCTCTCTCTTCAGGAACAGCTCATGCTGGAGCTTCTGTTGATTTAGCTATTTTTT
CTCTTCATTTAGCTGGAATTTCCTCAATTTTAGGGGCAGTAAATTTTATTACAACTGTAA
TTAATATACGATCGTCAGGAATTACTTTAGATCGACTACCTTTATTTGTTTGATCTGTAG
TTATTACAGCTATCTTATTACTTCTTTCTCTTCCTGTTTTAGCTGGAGCTATTACTATGT
TATTAACAGACCGAAACTTAA---------------------------------------
--
>MK028232.1
------------------TTTTAATTCGTGCTGAACTTAGCCACCCTGGTATATTTATTG
GGAATGACCAAATTTATAATGTAATTGTAACAGCTCATGCATTTATTATAATTTTCTTTA
TAGTAATGCCAATTATAATTGGAGGATTTGGAAATTGATTAGTTCCTTTAATATTAGGAG
CCCCTGATATAGCTTTCCCTCGAATGAATAATATAAGTTTTTGAATACTACCTCCTTCAT
TGACTCTTCTATTATCAAGCTCAATAGTAGAAAATGGAGCAGGAACTGGATGAACAGTTT
ATCCTCCTCTTTCTTCAGGAACAGCTCATGCTGGAGCTTCTGTTGATTTAGCTATTTTTT
CTCTTCATTTAGCTGGAATTTCCTCAATTTTAGGGGCAGTAAATTTTATTACAACTGTAA
TTAATATACGATCGTCAGGGATTACTTTAGATCGACTACCCTTATTTGTTTGATCTGTAG
TTATTACAGCTATCTTATTACTTCTTTCTCTTCCTGTTTTAGCTGGAGCTATTACTATAT
TATTAACAGACCGAAACTTAAATAC-----------------------------------
--
>MK028233.1
--ACCCTGGTATATTTATTG
GGAATGACCAAATTTATAATGTAATTGTAACAGCTCATGCATTTATTATAATTTTCTTTA
TAGTAATGCCAATTATAATTGGAGGATTTGGAAATTGATTAGTTCCTTTAATATTAGGAG
CCCCTGATATAGCTTTCCCTCGAATGAATAATATAAGTTTTTGAATACTACCTCCTTCAT
TGACTCTTCTATTATCAAGCTCAATAGTAGAAAATGGAGCAGGAACTGGATGAACAGTTT
ATCCTCCTCTTTCTTCAGGAACAGCTCATGCTGGAGCTTCTGTTGATTTAGCTATTTTTT
CTCTTCATTTAGCTGGAATTTCCTCAATTTTAGGGGCAGTAAATTTTATTACAACTGTAA
TTAATATACGATCGTCAGGAATTACTTTAGATCGACTACCCTTATTTGTTTGATCTGTAG
TTATTACAGCTATCTTATTACTTCTTTCTCTTCCTGTTTTAGCTGGAGCTATTACTATAT
TATTA---
--
>MK028234.1
--------------------------------------AGCCACCCTGGTATATTTATTG
GGAATGACCAAATTTATAATGTAATTGTAACAGCTCATGCATTTATTATAATTTTCTTTA
TAGTAATGCCAATTATAATTGGAGGATTTGGAAATTGATTAGTTCCTTTAATATTAGGAG
CCCCTGATATAGCTTTTCCTCGAATGAATAATATAAGTTTTTGAATACTACCTCCTTCAT
TGACTCTTCTATTATCAAGCTCAATAGTAGAAAATGGAGCAGGAACTGGATGAACAGTTT
ATCCTCCTCTTTCTTCAGGAACAGCTCATGCTGGAGCTTCTGTTGATTTAGCTATTTTTT
CTCTTCATTTAGCTGGAATTTCCTCAATTTTAGGGGCAGTAAATTTTATTACAACTGTAA
TTAATATACGATCGTCAGGAATTACTTTAGATCGACTACCCTTATTTGTTTGATCTGTAG
TTATTACAGCTATCTTATTACTTCTTTCTCTTCCTGTTTTAGCTGGAGCTATTACTATAT

TAT---
--
>MK028235.1
--------------------------------------AGCCACCCTGGTATATTTATTG
GGAATGACCAAATTTATAATGTAATTGTAACAGCTCATGCATTTATTATAATTTTCTTTA
TAGTAATACCAATTATAATTGGAGGATTTGGAAATTGATTAGTTCCTTTAATATTAGGAG
CCCCTGATATAGCCTTTCCTCGAATAAATAATATAAGTTTTTGAATACTACCTCCTTCAT
TGACTCTTCTATTATCAAGCTCAATAGTAGAAAATGGGGCAGGAACTGGGTGAACAGTTT
ATCCTCCTCTCTCTTCAGGAACAGCTCATGCTGGAGCTTCTGTTGATTTAGCTATTTTTT
CTCTTCATTTAGCTGGAATTTCCTCAATTTTAGGGGCAGTAAATTTTATTACAACTGTAA
TTAATATACGATCGTCAGGAATTACTTTAGATCGACTACCTTTATTTGTTTGATCTGTAG
TTATTACAGCTATCTTATTACTTCTTTCTCTTCCTGTTTTAGCTGGAGCTATTACTATGT
TATTA---
--
>MK028236.1
---------------------------------------GCCACCCTGGTATATTTATTG
GGAATGACCAAATTTATAATGTAATTGTAACAGCTCATGCATTTATTATAATTTTCTTTA
TAGTAATACCAATTATAATTGGAGGATTTGGAAATTGATTAGTTCCTTTAATATTAGGAG
CCCCTGATATAGCCTTTCCTCGAATAAATAATATAAGTTTTTGAATACTACCTCCTTCAT
TGACTCTTCTATTATCAAGCTCAATAGTAGAAAATGGGGCAGGAACTGGGTGAACAGTTT
ATCCTCCTCTCTCTTCAGGAACAGCTCATGCTGGAGCTTCTGTTGATTTAGCTATTTTTT
CTCTTCATTTAGCTGGAATTTCCTCAATTTTAGGGGCAGTAAATTTTATTACAACTGTAA
TTAATATACGATCGTCAGGAATTACTTTAGATCGACTACCTTTATTTGTTTGATCTGTAG
TTATTACAGCTATCTTATTACTTCTTTCTCTTCCTGTTTTAGCTGGAGCTATTACTATGT
TATT--
--
>MK028237.1
-------------------------------------TAGCCACCCTGGTATATTTATTG
GGAATGACCAAATTTATAATGTAATTGTAACAGCTCATGCATTTATTATAATTTTCTTTA
TAGTAATACCAATTATAATTGGAGGATTTGGAAATTGATTAGTTCCTTTAATATTAGGAG
CCCCTGATATAGCCTTTCCTCGAATAAATAATATAAGTTTTTGAATACTACCTCCTTCAT
TGACTCTTCTATTATCAAGCTCAATAGTAGAAAATGGGGCAGGAACTGGGTGAACAGTTT
ATCCTCCTCTCTCTTCAGGAACAGCTCATGCTGGAGCTTCTGTTGATTTAGCTATTTTTT
CTCTTCATTTAGCTGGAATTTCCTCAATTTTAGGGGCAGTAAATTTTATTACAACTGTAA
TTAATATACGATCGTCAGGAATTACTTTAGATCGACTACCTTTATTTGTTTGATCTGTAG
TTATTACAGCTATCTTATTACTTCTTTCTCTTCCTGTTTTAGCTGGAGCTATTACTATGT
TATTA---
--
>MK028238.1
------------------TTTTAATTCGTGCTGAACTTAGCCACCCTGGTATATTTATTG
GGAATGACCAAATTTATAATGTAATTGTAACAGCTCATGCATTTATTATAATTTTCTTTA
TAGTAATACCAATTATAATTGGAGGATTTGGAAATTGATTAGTTCCTTTAATATTAGGAG
CCCCTGATATAGCCTTTCCTCGAATAAATAATATAAGTTTTTGAATACTACCTCCTTCAT
TGACTCTTCTATTATCAAGCTCAATAGTAGAAAATGGGGCAGGAACTGGGTGAACAGTTT
ATCCTCCTCTCTCTTCAGGAACAGCTCATGCTGGAGCTTCTGTTGATTTAGCTATTTTTT
CTCTTCATTTAGCTGGAATTTCCTCAATTTTAGGGGCAGTAAATTTTATTACAACTGTAA
TTAATATACGATCGTCAGGAATTACTTTAGATCGACTACCTTTATTTGTTTGATCTGTAG
TTATTACAGCTATCTTATTACTTCTTTCTCTTCCTGTTTTAGCTGGAGCTATTACTATGT
TATTAA--
--
>MK028239.1
------------------TTTTAATTCGTGCTGAACTTAGCCACCCTGGTATATTTATTG
GGAATGACCAAATTTATAATGTAATTGTAACAGCTCATGCATTTATTATAATTTTCTTTA
TAGTAATACCAATTATAATTGGAGGATTTGGAAATTGATTAGTTCCTTTAATATTAGGAG
CCCCTGATATAGCCTTTCCTCGAATAAATAATATAAGTTTTTGAATACTACCTCCTTCAT
TGACTCTTCTATTATCAAGCTCAATAGTAGAAAATGGGGCAGGAACTGGGTGAACAGTTT
ATCCTCCTCTCTCTTCAGGAACAGCTCATGCTGGAGCTTCTGTTGATTTAGCTATTTTTT

```
CTCTTCATTTAGCTGGAATTTCCTCAATTTTAGGGGCAGTAAATTTTATTACAACTGTAA
TTAATATACGATCGTCAGGAATTACTTTAGATCGACTACCTTTATTTGTTTGATCTGTAG
TTATTACAGCTATCTTATTACTTCTTTCTCTTCCTGTTTTAGCTGGAGCTATTACTATGT
TATTAACAGACCGAAACTTAAATA------------------------------------
--
>MK028240.1
--------------------------CGTGCTGAACTTAGCCACCCTGGTATATTTATTG
GGAATGACCAAATTTATAATGTAATTGTAACAGCTCATGCATTTATTATAATTTTCTTTA
TAGTAATGCCAATTATAATTGGAGGATTTGGAAATTGATTAGTTCCTTTAATATTAGGAG
CCCCTGATATAGCTTTCCCTCGAATGAATAATATAAGTTTTTGAATACTACCTCCTTCAT
TGACTCTTCTATTATCAAGCTCAATAGTAGAAAATGGAGCAGGAACTGGATGAACAGTTT
ATCCTCCTCTTTCTTCAGGAACAGCTCATGCTGGAGCTTCTGTTGATTTAGCTATTTTTT
CTCTTCATTTAGCTGGAATTTCCTCAATTTTAGGGGCAGTAAATTTTATTACAACTGTAA
TTAATATACGATCGTCAGGGATTACTTTAGATCGACTACCCTTATTTGTTTGATCTGTAG
TTATTACAGCTATCTTATTACTTCTTTCTCTTCCTGTTTTAGCTGGAGCTATTACTATAT
TAT---------------------------------------------------------
--
>MK028241.1
------------------TTTTAATTCGTGCTGAACTTAGCCACCCTGGTATATTTATTG
GGAATGACCAAATTTATAATGTAATTGTAACAGCTCATGCATTTATTATAATTTTCTTTA
TAGTAATGCCAATTATAATTGGAGGATTTGGAAATTGATTAGTTCCTTTAATATTAGGAG
CCCCTGATATAGCTTTCCCTCGAATGAATAATATAAGTTTTTGAATACTACCTCCTTCAT
TGACTCTTCTATTATCAAGCTCAATAGTAGAAAATGGAGCAGGAACTGGATGAACAGTTT
ATCCTCCTCTTTCTTCAGGAACAGCTCATGCTGGAGCTTCTGTTGATTTAGCTATTTTTT
CTCTTCATTTAGCTGGAATTTCCTCAATTTTAGGGGCAGTAAATTTTATTACAACTGTAA
TTAATATACGATCGTCAGGGATTACTTTAGATCGACTACCCTTATTTGTTTGATCTGTAG
TTATTACAGCTATCTTATTACTTCTTTCTCTTCCTGTTTTAGCTGGAGCTATTACTATAT
TA----------------------------------------------------------
--
>MK028242.1
-------------------TTTAATTCGTGCTGAACTTAGCCACCCTGGTATATTTATTG
GGAATGACCAAATTTATAATGTAATTGTAACAGCTCATGCATTTATTATAATTTTCTTTA
TAGTAATGCCAATTATAATTGGAGGATTTGGAAATTGATTAGTTCCTTTAATATTAGGAG
CCCCTGATATAGCTTTCCCTCGAATGAATAATATAAGTTTTTGAATACTACCTCCTTCAT
TGACTCTTCTATTATCAAGCTCAATAGTAGAAAATGGAGCAGGAACTGGATGAACAGTTT
ATCCTCCTCTTTCTTCAGGAACAGCTCATGCTGGAGCTTCTGTTGATTTAGCTATTTTTT
CTCTTCATTTAGCTGGAATTTCCTCAATTTTAGGGGCAGTAAATTTTATTACAACTGTAA
TTAATATACGATCGTCAGGGATTACTTTAGATCGACTACCCTTATTTGTTTGATCTGTAG
TTATTACAGCTATCTTATTACTTCTTTCTCTTCCTGTTTTAGCTGGAGCTATTACTATAT
TATTAA------------------------------------------------------
--
>MK028243.1
------------------------------CTGAACTTAGCCACCCTGGTATATTTATTG
GGAATGACCAAATTTATAATGTAATTGTAACAGCTCATGCATTTATTATAATTTTCTTTA
TAGTAATACCAATTATAATTGGAGGATTTGGAAATTGATTAGTTCCTTTAATATTAGGAG
CCCCTGATATAGCTTTTCCTCGAATAAATAATATAAGTTTCTGAATACTACCTCCTTCAT
TAACTCTACTATTATCAAGCTCAATAGTAGAAAATGGAGCAGGAACTGGATGAACAGTTT
ATCCTCCTCTCTCTTCAGGAACAGCTCATGCTGGGGCTTCTGTTGATTTAGCTATTTTTT
CTCTTCATTTAGCTGGAATTTCCTCAATTTTAGGAGCAGTAAATTTTATTACAACTGTAA
TTAATATACGATCATCAGGAATTACTTTAGATCGACTACCCTTATTTGTTTGATCTGTAG
TTATTACAGCTATCTTATTACTTCTTTCTCTTCCTGTTTTAGCTGGAGCTATTACTATAT
TAT---------------------------------------------------------
--
>MK028244.1
-----------------------------GCTGAACTTAGCCACCCTGGTATATTTATTG
GGAATGACCAAATTTATAATGTAATTGTAACAGCTCATGCATTTATTATAATTTTCTTTA
TAGTAATACCAATTATAATTGGAGGATTTGGAAATTGATTAGTTCCTTTAATATTAGGAG
```

CCCCTGATATAGCCTTTCCTCGAATAAATAATATAAGTTTTTGAATACTACCTCCTTCAT
TGACTCTTCTATTATCAAGCTCAATAGTAGAAAATGGGGCAGGAACTGGGTGAACAGTTT
ATCCTCCTCTCTCTTCAGGAACAGCTCATGCTGGAGCTTCTGTTGATTTAGCTATTTTTT
CTCTTCATTTAGCTGGAATTTCCTCAATTTTAGGGGCAGTAAATTTTATTACAACTGTAA
TTAATATACGATCGTCAGGAATTACTTTAGATCGACTACCTTTATTTGTTTGATCTGTAG
TTATTACAGCTATCTTATTACTTCTTTCTCTTCCTGTTTTAGCTGGAGCTATTACTATGT
TATTAACAGACCG---
--
>MK028245.1
-------------------------------TGAACTTAGCCACCCTGGTATATTTATTG
GGAATGACCAAATTTATAATGTAATTGTAACAGCTCATGCATTTATTATAATTTTCTTTA
TAGTAATACCAATTATAATTGGAGGATTTGGAAATTGATTAGTTCCTTTAATATTAGGAG
CCCCTGATATAGCCTTTCCTCGAATAAATAATATAAGTTTTTGAATACTACCTCCTTCAT
TGACTCTTCTATTATCAAGCTCAATAGTAGAAAATGGGGCAGGAACTGGGTGAACAGTTT
ATCCTCCTCTCTCTTCAGGAACAGCTCATGCTGGAGCTTCTGTTGATTTAGCTATTTTTT
CTCTTCATTTAGCTGGAATTTCCTCAATTTTAGGGGCAGTAAATTTTATTACAACTGTAA
TTAATATACGATCGTCAGGAATTACTTTAGATCGACTACCTTTATTTGTTTGATCTGTAG
TTATTACAGCTATCTTATTACTTCTTTCTCTTCCTGTTTTAGCTGGAGCTATTACTATGT
TATTAACAGACC--
--
>MK028246.1
-------------------TTTAATTCGTGCTGAACTTAGCCACCCTGGTATATTTATTG
GGAATGACCAAATTTATAATGTAATTGTAACAGCTCATGCATTTATTATAATTTTCTTTA
TAGTAATACCAATTATAATTGGAGGATTTGGAAATTGATTAGTTCCTTTAATATTAGGAG
CCCCTGATATAGCTTTTCCTCGAATAAATAATATAAGTTTCTGAATACTACCTCCTTCAT
TAACTCTACTATTATCAAGCTCAATAGTAGAAAATGGAGCAGGAACTGGATGAACAGTTT
ATCCTCCTCTCTCTTCAGGAACAGCTCATGCTGGGGCTTCTGTTGATTTAGCTATTTTTT
CTCTTCATTTAGCTGGAATTTCCTCAATTTTAGGAGCAGTAAATTTTATTACAACTGTAA
TTAATATACGATCATCAGGAATTACTTTAGATCGACTACCCTTATTTGTTTGATCTGTAG
TTATTACAGCTATCTTATTACTTCTTTCTCTTCCTGTTTTAGCTGGAGCTATTACTATAT
TAT---
--
>MK028247.1
-------------------------------TGAACTTAGCCACCCTGGTATATTTATTG
GGAATGACCAAATTTATAATGTAATTGTAACAGCTCATGCATTTATTATAATTTTCTTTA
TAGTAATACCAATTATAATTGGAGGATTTGGAAATTGATTAGTTCCTTTAATATTAGGAG
CCCCTGATATAGCTTTTCCTCGAATAAATAATATAAGTTTTTGAATACTACCTCCTTCAT
TGACTCTACTATTATCAAGCTCAATAGTAGAAAATGGAGCAGGAACTGGATGAACAGTTT
ATCCTCCTCTCTCTTCAGGAACAGCTCATGCTGGAGCTTCTGTTGATTTAGCTATTTTTT
CTCTTCATTTAGCTGGAATTTCCTCAATTTTAGGAGCAGTAAATTTTATTACAACTGTAA
TTAATATACGATCGTCAGGAATTACTTTAGATCGACTACCCTTATTTGTTTGATCTGTAG
TTATTACAGCTATCTTATTACTTCTTTCTCTTCCTGTTTTAGCTGGAGCTATTACTATAT
TAT---
--
>MK028248.1
----------------------------TGCTGAACTTAGCCACCCTGGTATATTTATTG
GGAATGACCAAATTTATAATGTAATTGTAACAGCTCATGCATTTATTATAATTTTCTTTA
TAGTAATACCAATTATAATTGGAGGATTTGGAAATTGATTAGTTCCTTTAATATTAGGAG
CCCCTGATATAGCCTTTCCTCGAATAAATAATATAAGTTTTTGAATACTACCTCCTTCAT
TGACTCTTCTATTATCAAGCTCAATAGTAGAAAATGGGGCAGGAACTGGGTGAACAGTTT
ATCCTCCTCTCTCTTCAGGAACAGCTCATGCTGGAGCTTCTGTTGATTTAGCTATTTTTT
CTCTTCATTTAGCTGGAATTTCCTCAATTTTAGGGGCAGTAAATTTTATTACAACTGTAA
TTAATATACGATCGTCAGGAATTACTTTAGATCGACTACCTTTATTTGTTTGATCTGTAG
TTATTACAGCTATCTTATTACTTCTTTCTCTTCCTGTTTTAGCTGGAGCTATTACTATGT
TATTAACAGACCGA--
--
>MK028249.1

------------------TTTTAATTCGTGCTGAACTTAGCCACCCTGGTATATTTATTG
GGAATGACCAAATTTATAATGTAATTGTAACAGCTCATGCATTTATTATAATTTTCTTTA
TAGTAATACCAATTATAATTGGAGGATTTGGAAATTGATTAGTTCCTTTAATATTAGGAG
CCCCTGATATAGCTTTTCCTCGAATAAATAATATAAGTTTCTGAATACTACCTCCTTCAT
TAACTCTACTATTATCAAGCTCAATAGTAGAAAATGGAGCAGGAACTGGATGAACAGTTT
ATCCTCCTCTCTCTTCAGGAACAGCTCATGCTGGGGCTTCTGTTGATTTAGCTATTTTTT
CTCTTCATTTAGCTGGAATTTCCTCAATTTTAGGAGCAGTAAATTTTATTACAACTGTAA
TTAATATACGATCATCAGGAATTACTTTAGATCGACTACCCTTATTTGTTTGATCTGTAG
TTATTACAGCTATCTTATTACTTCTTTCTCTTCCTGTTTTAGCTGGAGCTATTACTATAT
TAT---
--
>MK028250.1
-------TTCTCTAAGAATTTTAATTCGTGCTGAACTTAGCCACCCTGGTATATTTATTG
GGAATGACCAAATTTATAATGTAATTGTAACAGCTCATGCATTTATTATAATTTTCTTTA
TAGTAATACCAATTATAATTGGAGGATTTGGAAATTGATTAGTTCCTTTAATATTAGGAG
CCCCTGATATAGCCTTTCCTCGAATAAATAATATAAGTTTTTGAATACTACCTCCTTCAT
TGACTCTTCTATTATCAAGCTCAATAGTAGAAAATGGGGCAGGAACTGGGTGAACAGTTT
ATCCTCCTCTCTCTTCAGGAACAGCTCATGCTGGAGCTTCTGTTGATTTAGCTATTTTTT
CTCTTCATTTAGCTGGAATTTCCTCAATTTTAGGGGCAGTAAATTTTATTACAACTGTAA
TTAATATACGATCGTCAGGAATTACTTTAGATCGACTACCTTTATTTGTTTGATCTGTAG
TTATTACAGCTATCTTATTACTTCTTTCTCTTCCTGTTTTAGCTGGAGCTATTACTATGT
TATTAACAGACCGAAACTTAAAT-------------------------------------
--
>MK028251.1
----------------------------TGCTGAACTTAGCCACCCTGGTATATTTATTG
GGAATGACCAAATTTATAATGTAATTGTAACAGCTCATGCATTTATTATAATTTTCTTTA
TAGTAATGCCAATTATAATTGGAGGATTTGGAAATTGATTAGTTCCTTTAATATTAGGAG
CCCCTGATATAGCTTTCCCTCGAATGAATAATATAAGTTTTTGAATACTACCTCCTTCAT
TGACTCTTCTATTATCAAGCTCAATAGTAGAAAATGGAGCAGGAACTGGATGAACAGTTT
ATCCTCCTCTTTCTTCAGGAACAGCTCATGCTGGAGCTTCTGTTGATTTAGCTATTTTTT
CTCTTCATTTAGCTGGAATTTCCTCAATTTTAGGGGCAGTAAATTTTATTACAACTGTAA
TTAATATACGATCGTCAGGAATTACTTTAGATCGACTACCCTTATTTGTTTGATCTGTAG
TTATTACAGCTATCTTATTACTTCTTTCTCTTCCTGTTTTAGCTGGAGCTATTACTATAT
TATTAACAGACCGA--
--
>MK028252.1
-----------------ATTTTAATTCGTGCTGAACTTAGCCACCCTGGTATATTTATTG
GGAATGACCAAATTTATAATGTAATTGTAACAGCTCATGCATTTATTATAATTTTCTTTA
TAGTAATACCAATTATAATTGGAGGATTTGGAAATTGATTAGTTCCTTTAATATTAGGAG
CCCCTGATATAGCCTTTCCTCGAATAAATAATATAAGTTTTTGAATACTACCTCCTTCAT
TGACTCTTCTATTATCAAGCTCAATAGTAGAAAATGGGGCAGGAACTGGATGAACAGTTT
ATCCTCCTCTCTCTTCAGGAACAGCTCATGCTGGAGCTTCTGTTGATTTAGCTATTTTTT
CTCTTCATTTAGCTGGAATTTCCTCAATTTTAGGAGCAGTAAATTTTATTACAACTGTAA
TTAATATACGATCGTCAGGAATTACTTTAGATCGACTACCTTTATTTGTTTGATCTGTAG
TTATTACAGCTATCTTATTACTTCTTTCTCTTCCTGTTTTAGCTGGAGCTATTACTA---

--
>MK028253.1
-----ACTTCTCTAAGAATTTTAATTCGTGCTGAACTTAGCCACCCTGGTATATTTATTG
GGAATGACCAAATTTATAATGTAATTGTAACAGCTCATGCATTTATTATAATTTTCTTTA
TAGTAATGCCAATTATAATTGGAGGATTTGGAAATTGATTAGTTCCTTTAATATTAGGAG
CCCCTGATATAGCTTTCCCTCGAATGAATAATATAAGTTTTTGAATACTACCTCCTTCAT
TGACTCTTCTATTATCAAGCTCAATAGTAGAAAATGGAGCAGGAACTGGATGAACAGTTT
ATCCTCCTCTTTCTTCAGGAACAGCTCATGCTGGAGCTTCTGTTGATTTAGCTATTTTTT
CTCTTCATTTAGCTGGAATTTCCTCAATTTTAGGGGCAGTAAATTTTATTACAACTGTAA
TTAATATACGATCGTCAGGAATTACTTTAGATCGACTACCCTTATTTGTTTGATCTGTAG
TTATTACAGCTATCTTATTACTTCTTTCTCTTCCTGTTTTAGCTGGAGCTATTACTATAT

TATTAACAGACCGAAACTTAAATACATCTT------------------------------
--
>MK028254.1
--------------------TTAATTCGTGCTGAACTTAGCCACCCTGGTATATTTATTG
GGAATGACCAAATTTATAATGTAATTGTAACAGCTCATGCATTTATTATAATTTTCTTTA
TAGTAATACCAATTATAATTGGAGGATTTGGAAATTGATTAGTTCCTTTAATATTAGGAG
CCCCTGATATAGCCTTTCCTCGAATAAATAATATAAGTTTTTGAATACTACCTCCTTCAT
TGACTCTTCTATTATCAAGCTCAATAGTAGAAAATGGGGCAGGAACTGGGTGAACAGTTT
ATCCTCCTCTCTCTTCAGGAACAGCTCATGCTGGAGCTTCTGTTGATTTAGCTATTTTTT
CTCTTCATTTAGCTGGAATTTCCTCAATTTTAGGGGCAGTAAATTTTATTACAACTGTAA
TTAATATACGATCGTCAGGAATTACTTTAGATCGACTACCTTTATTTGTTTGATCTGTAG
TTATTACAGCTATCTTATTACTTCTTTCTCTTCCTGTTTTAGCTGGAGCTATTACTATGT
TATTAACAGACCGAAACTTAAATA------------------------------------
--
>MK028255.1
-------------------TTTAATTCGTGCTGAACTTAGCCACCCTGGTATATTTATTG
GGAATGACCAAATTTATAATGTAATTGTAACAGCTCATGCATTTATTATAATTTTCTTTA
TAGTAATGCCAATTATAATTGGAGGATTTGGAAATTGATTAGTTCCTTTAATATTAGGAG
CCCCTGATATAGCTTTTCCTCGAATGAATAATATAAGTTTTTGAATACTACCTCCTTCAT
TGACTCTTCTATTATCAAGCTCAATAGTAGAAAATGGAGCAGGAACTGGATGAACAGTTT
ATCCTCCTCTTTCTTCAGGAACAGCTCATGCTGGAGCTTCTGTTGATTTAGCTATTTTTT
CTCTTCATTTAGCTGGAATTTCCTCAATTTTAGGGGCAGTAAATTTTATTACAACTGTAA
TTAATATACGATCGTCAGGAATTACTTTAGATCGACTACCCTTATTTGTTTGATCTGTAG
TTATTACAGCTATCTTATTACTTCTTTCTCTTCCTGTTTTAGCTGGAGCTATTACTATAT
TATTAACAGACCGAAACTTAAATA------------------------------------
--
>MK028256.1
------------------TTTTAATTCGTGCTGAACTTAGCCACCCTGGTATATTTATTG
GGAATGACCAAATTTATAATGTAATTGTAACAGCTCATGCATTTATTATAATTTTCTTTA
TAGTAATGCCAATTATAATTGGAGGATTTGGAAATTGATTAGTTCCTTTAATATTAGGAG
CCCCTGATATAGCTTTCCCTCGAATGAATAATATAAGTTTTTGAATACTACCTCCTTCAT
TGACTCTTCTATTATCAAGCTCAATAGTAGAAAATGGAGCAGGAACTGGATGAACAGTTT
ATCCTCCTCTTTCTTCAGGAACAGCTCATGCTGGAGCTTCTGTTGATTTAGCTATTTTTT
CTCTTCATTTAGCTGGAATTTCCTCAATTTTAGGGGCAGTAAATTTTATTACAACTGTAA
TTAATATACGATCGTCAGGGATTACTTTAGATCGACTACCCTTATTTGTTTGATCTGTAG
TTATTACAGCTATCTTATTACTTCTTTCTCTTCCTGTTTTAGCTG---------------

--
>MK028257.1
-------------------TTTAATTCGTGCTGAACTTAGCCACCCTGGTATATTTATTG
GGAATGACCAAATTTATAATGTAATTGTAACAGCTCATGCATTTATTATAATTTTCTTTA
TAGTAATGCCAATTATAATTGGAGGATTTGGAAATTGATTAGTTCCTTTAATATTAGGAG
CCCCTGATATAGCTTTCCCTCGAATGAATAATATAAGTTTTTGAATACTACCTCCTTCAT
TGACTCTTCTATTATCAAGCTCAATAGTAGAAAATGGAGCAGGAACTGGATGAACAGTTT
ATCCTCCTCTTTCTTCAGGAACAGCTCATGCTGGAGCTTCTGTTGATTTAGCTATTTTTT
CTCTTCATTTAGCTGGAATTTCCTCAATTTTAGGGGCAGTAAATTTTATTACAACTGTAA
TTAATATACGATCGTCAGGAATTACTTTAGATCGACTACCCTTATTTGTTTGATCTGTAG
TTATTACAGCTATCTTATTACTTCTTTCTCTTCCTGTTTTAGCTGGAGCTATTACTATAT
TATTAACAGACCGAAACTTAAATA------------------------------------
--
>MK028258.1
-----------------ATTTTAATTCGTGCTGAACTTAGCCACCCTGGTATATTTATTG
GGAATGACCAAATTTATAATGTAATTGTAACAGCTCATGCATTTATTATAATTTTCTTTA
TAGTAATGCCAATTATAATTGGAGGATTTGGAAATTGATTAGTTCCTTTAATATTAGGAG
CCCCTGATATAGCTTTCCCTCGAATGAATAATATAAGTTTTTGAATACTACCTCCTTCAT
TGACTCTTCTATTATCAAGCTCAATAGTAGAAAATGGAGCAGGAACTGGATGAACAGTTT
ATCCTCCTCTTTCTTCAGGAACAGCTCATGCTGGAGCTTCTGTTGATTTAGCTATTTTTT

CTCTTCATTTAGCTGGAATTTCCTCAATTTTAGGGGCAGTAAATTTTATTACAACTGTAA
TTAATATACGATCGTCAGGGATTACTTTAGATCGACTACCCTTATTTGTTTGATCTGTAG
TTATTACAGCTATCTTATTACTTCTTTCTCTTCCTGTTTTAGCTGGAGCTATTACTATAT
TATTAACAGACCGAAACTTAAATACA----------------------------------
--
>MK028259.1
------------------------------------TTAGCCACCCTGGTATATTTATTG
GGAATGACCAAATTTATAATGTAATTGTAACAGCTCATGCATTTATTATAATTTTCTTTA
TAGTAATGCCAATTATAATTGGAGGATTTGGAAATTGATTAGTTCCTTTAATATTAGGAG
CCCCTGATATAGCTTTCCCTCGAATGAATAATATAAGTTTTTGAATACTACCTCCTTCAT
TGACTCTTCTATTATCAAGCTCAATAGTAGAAAATGGAGCAGGAACTGGATGAACAGTTT
ATCCTCCTCTTTCTTCAGGAACAGCTCATGCTGGAGCTTCTGTTGATTTAGCTATTTTTT
CTCTTCATTTAGCTGGAATTTCCTCAATTTTAGGGGCAGTAAATTTTATTACAACTGTAA
TTAATATACGATCGTCAGGGATTACTTTAGATCGACTACCCTTATTTGTTTGATCTGTAG
TTATTACAGCTATCTTATTACTTCTTTCTCTTCCTGTTTTAGCTGGAGCTATTACTATAT
TATT--
--
>MK028260.1
-----------------------------------CTTAGCCACCCTGGTATATTTATTG
GGAATGACCAAATTTATAATGTAATTGTAACAGCTCATGCATTTATTATAATTTTCTTTA
TAGTAATGCCAATTATAATTGGAGGATTTGGAAATTGATTAGTTCCTTTAATATTAGGAG
CCCCTGATATAGCTTTCCCTCGAATGAATAATATAAGTTTTTGAATACTACCTCCTTCAT
TGACTCTTCTATTATCAAGCTCAATAGTAGAAAATGGAGCAGGAACTGGATGAACAGTTT
ATCCTCCTCTTTCTTCAGGAACAGCTCATGCTGGAGCTTCTGTTGATTTAGCTATTTTTT
CTCTTCATTTAGCTGGAATTTCCTCAATTTTAGGGGCAGTAAATTTTATTACAACTGTAA
TTAATATACGATCGTCAGGGATTACTTTAGATCGACTACCCTTATTTGTTTGATCTGTAG
TTATTACAGCTATCTTATTACTTCTTTCTCTTCCTGTTTTAGCTGGAGCTATTACTATAT
TAT---
--
>MK028261.1
-------------------------------------TAGCCACCCTGGTATATTTATTG
GGAATGACCAAATTTATAATGTAATTGTAACAGCTCATGCATTTATTATAATTTTCTTTA
TAGTAATACCAATTATAATTGGAGGATTTGGAAATTGATTAGTTCCTTTAATATTAGGAG
CCCCTGATATAGCCTTTCCTCGAATAAATAATATAAGTTTTTGAATACTACCTCCTTCAT
TGACTCTTCTATTATCAAGCTCAATAGTAGAAAATGGGGCAGGAACTGGGTGAACAGTTT
ATCCTCCTCTCTCTTCAGGAACAGCTCATGCTGGAGCTTCTGTTGATTTAGCTATTTTTT
CTCTTCATTTAGCTGGAATTTCCTCAATTTTAGGGGCAGTAAATTTTATTACAACTGTAA
TTAATATACGATCGTCAGGAATTACTTTAGATCGACTACCTTTATTTGTTTGATCTGTAG
TTATTACAGCTATCTTATTACTTCTTTCTCTTCCTGTTTTAGCTGGAGCTATTACTATGT
TATTA---
--
>MK028262.1
------------------------------------TTAGCCACCCTGGTATATTTATTG
GGAATGACCAAATTTATAATGTAATTGTAACAGCTCATGCATTTATTATAATTTTCTTTA
TAGTAATACCAATTATAATTGGAGGATTTGGAAATTGATTAGTTCCTTTAATATTAGGAG
CCCCTGATATAGCCTTTCCTCGAATAAATAATATAAGTTTTTGAATACTACCTCCTTCAT
TGACTCTTCTATTATCAAGCTCAATAGTAGAAAATGGGGCAGGAACTGGGTGAACAGTTT
ATCCTCCTCTCTCTTCAGGAACAGCTCATGCTGGAGCTTCTGTTGATTTAGCTATTTTTT
CTCTTCATTTAGCTGGAATTTCCTCAATTTTAGGGGCAGTAAATTTTATTACAACTGTAA
TTAATATACGATCGTCAGGAATTACTTTAGATCGACTACCTTTATTTGTTTGATCTGTAG
TTATTACAGCTATCTTATTACTTCTTTCTCTTCCTGTTTTAGCTGGAGCTATTACTATGT
TAT---
--
>MK028263.1
------------------------------------TTAGCCACCCTGGTATATTTATTG
GGAATGACCAAATTTATAATGTAATTGTAACAGCTCATGCATTTATTATAATTTTCTTTA
TAGTAATACCAATTATAATTGGAGGATTTGGAAATTGATTAGTTCCTTTAATATTAGGAG

CCCCTGATATAGCCTTTCCTCGAATAAATAATATAAGTTTTTGAATACTACCTCCTTCAT
TGACTCTTCTATTATCAAGCTCAATAGTAGAAAATGGGGCAGGAACTGGGTGAACAGTTT
ATCCTCCTCTCTCTTCAGGAACAGCTCATGCTGGAGCTTCTGTTGATTTAGCTATTTTTT
CTCTTCATTTAGCTGGAATTTCCTCAATTTTAGGGGCAGTAAATTTTATTACAACTGTAA
TTAATATACGATCGTCAGGAATTACTTTAGATCGACTACCTTTATTTGTTTGATCTGTAG
TTATTACAGCTATCTTATTACTTCTTTCTCTTCCTGTTTTAGCTGGAGCTATTACTATGT
TATTAA--
--
>MK028264.1
------------------------------------TTAGCCACCCTGGTATATTTATTG
GGAATGACCAAATTTATAATGTAATTGTAACAGCTCATGCATTTATTATAATTTTCTTTA
TAGTAATACCAATTATAATTGGAGGATTTGGAAATTGATTAGTTCCTTTAATATTAGGAG
CCCCTGATATAGCCTTTCCTCGAATAAATAATATAAGTTTTTGAATACTACCTCCTTCAT
TGACTCTTCTATTATCAAGCTCAATAGTAGAAAATGGGGCAGGAACTGGGTGAACAGTTT
ATCCTCCTCTCTCTTCAGGAACAGCTCATGCTGGAGCTTCTGTTGATTTAGCTATTTTTT
CTCTTCATTTAGCTGGAATTTCCTCAATTTTAGGGGCAGTAAATTTTATTACAACTGTAA
TTAATATACGATCGTCAGGAATTACTTTAGATCGACTACCTTTATTTGTTTGATCTGTAG
TTATTACAGCTATCTTATTACTTCTTTCTCTTCCTGTTTTAGCTGGAGCTATTACTATGT
TATTA---
--
>MK028265.1
-------------------------------------TAGCCACCCTGGTATATTTATTG
GGAATGACCAAATTTATAATGTAATTGTAACAGCTCATGCATTTATTATAATTTTCTTTA
TAGTAATACCAATTATAATTGGAGGATTTGGAAATTGATTAGTTCCTTTAATATTAGGAG
CCCCTGATATAGCCTTTCCTCGAATAAATAATATAAGTTTTTGAATACTACCTCCTTCAT
TGACTCTTCTATTATCAAGCTCAATAGTAGAAAATGGGGCAGGAACTGGGTGAACAGTTT
ATCCTCCTCTCTCTTCAGGAACAGCTCATGCTGGAGCTTCTGTTGATTTAGCTATTTTTT
CTCTTCATTTAGCTGGAATTTCCTCAATTTTAGGGGCAGTAAATTTTATTACAACTGTAA
TTAATATACGATCGTCAGGAATTACTTTAGATCGACTACCTTTATTTGTTTGATCTGTAG
TTATTACAGCTATCTTATTACTTCTTTCTCTTCCTGTTTTAGCTGGAGCTATTACTATGT
TATTA---
--
>MK028266.1
----AACTTCTCTAAGAATTTTAATTCGTGCTGAACTTAGCCACCCTGGTATATTTATTG
GGAATGACCAAATTTATAATGTAATTGTAACAGCTCATGCATTTATTATAATTTTCTTTA
TAGTAATACCAATTATAATTGGAGGATTTGGAAATTGATTAGTTCCTTTAATATTAGGAG
CCCCTGATATAGCTTTTCCTCGAATAAATAATATAAGTTTCTGAATACTACCTCCTTCAT
TAACTCTACTATTATCAAGCTCAATAGTAGAAAATGGAGCAGGAACTGGATGAACAGTTT
ATCCTCCTCTCTCTTCAGGAACAGCTCATGCTGGGGCTTCTGTTGATTTAGCTATTTTTT
CTCTTCATTTAGCTGGAATTTCCTCAATTTTAGGAGCAGTAAATTTTATTACAACTGTAA
TTAATATACGATCATCAGGAATTACTTTAGATCGACTACCCTTATTTGTTTGATCTGTAG
TTATTACAGCTATCTTATTACTTCTTTCTCTTCCTGTTTTAGCTGGAGCTATTACTATAT
TAT---
--
>MK028267.1
--GGAACTTCTCTAAGAATTTTAATTCGTGCTGAACTTAGCCACCCTGGTATATTTATTG
GGAATGACCAAATTTATAATGTAATTGTAACAGCTCATGCATTTATTATAATTTTCTTTA
TAGTAATACCAATTATAATTGGAGGATTTGGAAATTGATTAGTTCCTTTAATATTAGGAG
CCCCTGATATAGCTTTTCCTCGAATAAATAATATAAGTTTCTGAATACTACCTCCTTCAT
TAACTCTACTATTATCAAGCTCAATAGTAGAAAATGGAGCAGGAACTGGATGAACAGTTT
ATCCTCCTCTCTCTTCAGGAACAGCTCATGCTGGAGCTTCTGTTGATTTAGCTATTTTTT
CTCTTCATTTAGCTGGAATTTCCTCAATTTTAGGAGCAGTAAATTTTATTACAACTGTAA
TTAATATACGATCATCAGGAATTACTTTAGATCGACTACCCTTATTTGTTTGATCTGTAG
TTATTACAGCTATCTTATTACTTCTTTCTCTTCCTGTTTTAGCTGGAGCTATTACTATAT
TAT---
--
>MK028268.1

------CTTCTCTAAGAATTTTAATTCGTGCTGAACTTAGCCACCCTGGTATATTTATTG
GGAATGACCAAATTTATAATGTAATTGTAACAGCTCATGCATTTATTATAATTTTCTTTA
TAGTAATACCAATTATAATTGGAGGATTTGGAAATTGATTAGTTCCTTTAATATTAGGAG
CCCCTGATATAGCTTTTCCTCGAATAAATAATATAAGTTTCTGAATACTACCTCCTTCAT
TAACTCTACTATTATCAAGCTCAATAGTAGAAAATGGAGCAGGAACTGGATGAACAGTTT
ATCCTCCTCTCTCTTCAGGAACAGCTCATGCTGGGGCTTCTGTTGATTTAGCTATTTTTT
CTCTTCATTTAGCTGGAATTTCCTCAATTTTAGGAGCAGTAAATTTTATTACAACTGTAA
TTAATATACGATCATCAGGAATTACTTTAGATCGACTACCCTTATTTGTTTGATCTGTAG
TTATTACAGCTATCTTATTACTTCTTTCTCTTCCTGTTTTAGCTGGAGCTATTACTATAT
TATA--
--
>MK028269.1
TCGGAACTTCTCTAAGAATTTTAATTCGTGCTGAACTTAGCCACCCTGGTATATTTATTG
GGAATGACCAAATTTATAATGTAATTGTAACAGCTCATGCATTTATTATAATTTTCTTTA
TAGTAATACCAATTATAATTGGAGGATTTGGAAATTGATTAGTTCCTTTAATATTAGGAG
CCCCTGATATAGCCTTTCCTCGAATAAATAATATAAGTTTTTGAATACTACCTCCTTCAT
TGACTCTTCTATTATCAAGCTCAATAGTAGAAAATGGGGCAGGAACTGGGTGAACAGTTT
ATCCTCCTCTCTCTTCAGGAACAGCTCATGCTGGAGCTTCTGTTGATTTAGCTATTTTTT
CTCTTCATTTAGCTGGAATTTCCTCAATTTTAGGGGCAGTAAATTTTATTACAACTGTAA
TTAATATACGATCGTCAGGAATTACTTTAGATCGACTACCCTTATTTGTTTGATCTGTAG
TTATTACAGCTATCTTATTACTTCTTTCTCTTCCTGTTTTAGCTGGAGCTATTACTATGT
TATTAACAGACCGAAACTTAAATA------------------------------------
--
>MK028270.1
------------------------------------CTTAGCCACCCTGGTATATTTATTG
GGAATGACCAAATTTATAATGTAATTGTAACAGCTCATGCATTTATTATAATTTTCTTTA
TAGTAATACCAATTATAATTGGAGGATTTGGAAATTGATTAGTTCCTTTAATATTAGGAG
CCCCTGATATAGCCTTTCCTCGAATAAATAATATAAGTTTTTGAATACTACCTCCTTCAT
TGACTCTTCTATTATCAAGCTCAATAGTAGAAAATGGGGCAGGAACTGGGTGAACAGTTT
ATCCTCCTCTCTCTTCAGGAACAGCTCATGCTGGAGCTTCTGTTGATTTAGCTATTTTTT
CTCTTCATTTAGCTGGAATTTCCTCAATTTTAGGGGCAGTAAATTTTATTACAACTGTAA
TTAATATACGATCGTCAGGAATTACTTTAGATCGACTACCTTTATTTGTTTGATCTGTAG
TTATTACAGCTATCTTATTACTTCTTTCTCTTCCTGTTTTAGCTGGAGCTATTACTATGT
TATTA---
--
>MK028271.1
------------------------------------CTTAGCCACCCTGGTATATTTATTG
GGAATGACCAAATTTATAATGTAATTGTAACAGCTCATGCATTTATTATAATTTTCTTTA
TAGTAATACCAATTATAATTGGAGGATTTGGAAATTGATTAGTTCCTTTAATATTAGGAG
CCCCTGATATAGCCTTTCCTCGAATAAATAATATAAGTTTTTGAATACTACCTCCTTCAT
TGACTCTTCTATTATCAAGCTCAATAGTAGAAAATGGGGCAGGAACTGGGTGAACAGTTT
ATCCTCCTCTCTCTTCAGGAACAGCTCATGCTGGAGCTTCTGTTGATTTAGCTATTTTTT
CTCTTCATTTAGCTGGAATTTCCTCAATTTTAGGGGCAGTAAATTTTATTACAACTGTAA
TTAATATACGATCGTCAGGAATTACTTTAGATCGACTACCTTTATTTGTTTGATCTGTAG
TTATTACAGCTATCTTATTACTTCTTTCTCTTCCTGTTTTAGCTGGAGCTATTACTATGT
TATTA---
--
>MK028272.1
--------TCTCTAAGAATTTTAATTCGTGCTGAACTTAGCCACCCTGGTATATTTATTG
GGAATGACCAAATTTATAATGTAATTGTAACAGCTCATGCATTTATTATAATTTTCTTTA
TAGTAATACCAATTATAATTGGAGGATTTGGAAATTGATTAGTTCCTTTAATATTAGGAG
CCCCTGATATAGCTTTTCCTCGAATAAATAATATAAGTTTTTGAATACTACCTCCTTCAT
TAACTCTACTATTATCAAGCTCAATAGTAGAAAATGGAGCAGGAACTGGATGAACAGTTT
ATCCTCCTCTCTCTTCAGGAACAGCTCATGCTGGAGCTTCTGTTGATTTAGCTATTTTTT
CTCTTCATTTAGCTGGAATTTCCTCAATTTTAGGAGCAGTAAATTTTATTACAACTGTAA
TTAATATACGATCATCAGGAATTACTTTAGATCGACTACCCTTATTTGTTTGATCTGTAG
TTATTACAGCTATCTTATTACTTCTTTCTCTTCCTGTTTTAGCTGGAGCTATT-------

--
>MK028273.1
------------------TTTTAATTCGTGCTGAACTTAGCCACCCTGGTATATTTATTG
GGAATGACCAAATTTATAATGTAATTGTAACAGCTCATGCATTTATTATAATTTTCTTTA
TAGTAATACCAATTATAATTGGAGGATTTGGAAATTGATTAGTTCCTTTAATATTAGGAG
CCCCTGATATAGCCTTTCCTCGAATAAATAATATAAGTTTTTGAATACTACCTCCTTCAT
TGACTCTTCTATTATCAAGCTCAATAGTAGAAAATGGGGCAGGAACTGGGTGAACAGTTT
ATCCTCCTCTCTCTTCAGGAACAGCTCATGCTGGAGCTTCTGTTGATTTAGCTATTTTTT
CTCTTCATTTAGCTGGAATTTCCTCAATTTTAGGGGCAGTAAATTTTATTACAACTGTAA
TTAATATACGATCGTCAGGAATTACTTTAGATCGACTACCCTTATTTGTTTGATCTGTAG
TTATTACAGCTATCTTATTACTTCTTTCTCTTCCTGTTTTAGCTGGAGCTATTACTATGT
TATTAACAGACCGAAACT--
--
>MK028274.1
------------------TTTTAATTCGTGCTGAACTTAGCCACCCTGGTATATTTATTG
GGAATGACCAAATTTATAATGTAATTGTAACAGCTCATGCATTTATTATAATTTTCTTTA
TAGTAATACCAATTATAATTGGAGGATTTGGAAATTGATTAGTTCCTTTAATATTAGGAG
CCCCTGATATAGCCTTTCCTCGAATAAATAATATAAGTTTTTGAATACTACCTCCTTCAT
TGACTCTTCTATTATCAAGCTCAATAGTAGAAAATGGGGCAGGAACTGGGTGAACAGTTT
ATCCTCCTCTCTCTTCAGGAACAGCTCATGCTGGAGCTTCTGTTGATTTAGCTATTTTTT
CTCTTCATTTAGCTGGAATTTCCTCAATTTTAGGGGCAGTAAATTTTATTACAACTGTAA
TTAATATACGATCGTCAGGAATTACTTTAGATCGACTACCTTTATTTGTTTGATCTGTAG
TTATTACAGCTATCTTATTACTTCTTTCTCTTCCTGTTTTAGCTGGAGCTATTACTATGT
TATTAACAGACCGAAACTTAA---------------------------------------
--
>MK028275.1
-------------------TTTAATTCGTGCTGAACTTAGCCACCCTGGTATATTTATTG
GGAATGACCAAATTTATAATGTAATTGTAACAGCTCATGCATTTATTATAATTTTCTTTA
TAGTAATGCCAATTATAATTGGAGGATTTGGAAATTGATTAGTTCCTTTAATATTAGGAG
CCCCTGATATAGCCTTTCCTCGAATGAATAATATAAGTTTTTGAATACTACCTCCTTCAT
TGACTCTTCTATTATCAAGCTCAATAGTAGAAAATGGAGCAGGAACTGGATGAACAGTTT
ATCCTCCTCTCTCTTCAGGAACAGCTCATGCTGGAGCTTCTGTTGATTTAGCTATTTTTT
CTCTTCATTTAGCTGGAATTTCCTCAATTTTAGGGGCAGTAAATTTTATTACAACTGTAA
TTAATATACGATCGTCAGGAATTACTTTAGATCGACTACCCTTATTTGTTTGATCTGTAG
TTATTACAGCTATCTTATTACTTCTTTCTCTTCCTGTTTTAGCTGGAGCTATTACT----

--
>MK028276.1
--------------------TTAATTCGTGCTGAACTTAGCCACCCTGGTATATTTATTG
GGAATGACCAAATTTATAATGTAATTGTAACAGCTCATGCATTTATTATAATTTTCTTTA
TAGTAATACCAATTATAATTGGAGGATTTGGAAATTGATTAGTTCCTTTAATATTAGGAG
CCCCTGATATAGCCTTTCCTCGAATAAATAATATAAGTTTTTGAATACTACCTCCTTCAT
TGACTCTTCTATTATCAAGCTCAATAGTAGAAAATGGGGCAGGAACTGGGTGAACAGTTT
ATCCTCCTCTCTCTTCAGGAACAGCTCATGCTGGAGCTTCTGTTGATTTAGCTATTTTTT
CTCTTCATTTAGCTGGAATTTCCTCAATTTTAGGGGCAGTAAATTTTATTACAACTGTAA
TTAATATACGATCGTCAGGAATTACTTTAGATCGACTACCTTTATTTGTTTGATCTGTAG
TTATTACAGCTATCTTATTACTTCTTTCTCTTCCTGTTTTAGCTGGAGCTATTACTATGT
TATTAACAGACCGAAACTT---
--
>MK028277.1
-------------------TTTAATTCGTGCTGAACTTAGCCACCCTGGTATATTTATTG
GGAATGACCAAATTTATAATGTAATTGTAACAGCTCATGCATTTATTATAATTTTCTTTA
TAGTAATACCAATTATAATTGGAGGATTTGGAAATTGATTAGTTCCTTTAATATTAGGAG
CCCCTGATATAGCCTTTCCTCGAATAAATAATATAAGTTTTTGAATACTACCTCCTTCAT
TGACTCTTCTATTATCAAGCTCAATAGTAGAAAATGGGGCAGGAACTGGGTGAACAGTTT
ATCCTCCTCTCTCTTCAGGAACAGCTCATGCTGGAGCTTCTGTTGATTTAGCTATTTTTT

CTCTTCATTTAGCTGGAATTTCCTCAATTTTAGGGGCAGTAAATTTTATTACAACTGTAA
TTAATATACGATCGTCAGGAATTACTTTAGATCGACTACCCTTATTTGTTTGATCTGTAG
TTATTACAGCTATCTTATTACTTCTTTCTCTTCCTGTTTTAGCTGGAGCTATTACTATGT
TATTAACAGACCGAAACTTAAAT-------------------------------------
--
>MK028278.1
-------------------TTTAATTCGTGCTGAACTTAGCCACCCTGGTATATTTATTG
GGAATGACCAAATTTATAATGTAATTGTAACAGCTCATGCATTTATTATAATTTTCTTTA
TAGTAATACCAATTATAATTGGAGGATTTGGAAATTGATTAGTTCCTTTAATATTAGGAG
CCCCTGATATAGCCTTTCCTCGAATAAATAATATAAGTTTTTGAATACTACCTCCTTCAT
TGACTCTTCTATTATCAAGCTCAATAGTAGAAAATGGGGCAGGAACTGGGTGAACAGTTT
ATCCTCCTCTCTCTTCAGGAACAGCTCATGCTGGAGCTTCTGTTGATTTAGCTATTTTTT
CTCTTCATTTAGCTGGAATTTCCTCAATTTTAGGGGCAGTAAATTTTATTACAACTGTAA
TTAATATACGATCGTCAGGAATTACTTTAGATCGACTACCTTTATTTGTTTGATCTGTAG
TTATTACAGCTATCTTATTACTTCTTTCTCTTCCTGTTTTAGCTGGAGCTATTACT----

--
>MK028279.1
-----------------ATTTTAATTCGTGCTGAACTTAGCCACCCTGGTATATTTATTG
GGAATGACCAAATTTATAATGTAATTGTAACAGCTCATGCATTTATTATAATTTTCTTTA
TAGTAATGCCAATTATAATTGGAGGATTTGGAAATTGATTAGTTCCTTTAATATTAGGAG
CCCCTGATATAGCTTTCCCTCGAATGAATAATATAAGTTTTTGAATACTACCTCCTTCAT
TGACTCTTCTATTATCAAGCTCAATAGTAGAAAATGGAGCAGGAACTGGATGAACAGTTT
ATCCTCCTCTTTCTTCAGGAACAGCTCATGCTGGAGCTTCTGTTGATTTAGCTATTTTTT
CTCTTCATTTAGCTGGAATTTCCTCAATTTTAGGGGCAGTAAATTTTATTACAACTGTAA
TTAATATACGATCGTCAGGGATTACTTTAGATCGACTACCCTTATTTGTTTGATCTGTAG
TTATTACAGCTATCTTATTACTTCTTTCTCTTCCTGTTTTAGCTGGAGCTATTACTATAT
TATTAACAGACCGAAACTTAAATACATCTTTCTTT-------------------------
--
>MK028280.1
----------------AATTTTAATTCGTGCTGAACTTAGCCACCCTGGTATATTTATTG
GGAATGACCAAATTTATAATGTAATTGTAACAGCTCATGCATTTATTATAATTTTCTTTA
TAGTAATACCAATTATAATTGGAGGATTTGGAAATTGATTAGTTCCTTTAATATTAGGAG
CCCCTGATATAGCCTTTCCTCGAATAAATAATATAAGTTTTTGAATACTACCTCCTTCAT
TGACTCTTCTATTATCAAGCTCAATAGTAGAAAATGGGGCAGGAACTGGGTGAACAGTTT
ATCCTCCTCTCTCTTCAGGAACAGCTCATGCTGGAGCTTCTGTTGATTTAGCTATTTTTT
CTCTTCATTTAGCTGGAATTTCCTCAATTTTAGGGGCAGTAAATTTTATTACAACTGTAA
TTAATATACGATCGTCAGGAATTACTTTAGATCGACTACCTTTATTTGTTTGATCTGTAG
TTATTACAGCTATCTTATTACTTCTTTCTCTTCCTGTTTTAGCTGGAGCTATTACTATGT
TATTAACAGACCGAAACTTAAATACATCTTTCTTTGATCCAATCGGAGGA----------
--
>MK028281.1
----------------AATTTTAATTCGTGCTGAACTTAGCCACCCTGGTATATTTATTG
GGAATGACCAAATTTATAATGTAATTGTAACAGCTCATGCATTTATTATAATTTTCTTTA
TAGTAATACCAATTATAATTGGAGGATTTGGAAATTGATTAGTTCCTTTAATATTAGGAG
CCCCTGATATAGCCTTTCCTCGAATAAATAATATAAGTTTTTGAATACTACCTCCTTCAT
TGACTCTTCTATTATCAAGCTCAATAGTAGAAAATGGGGCAGGAACTGGGTGAACAGTTT
ATCCTCCTCTCTCTTCAGGAACAGCTCATGCTGGAGCTTCTGTTGATTTAGCTATTTTTT
CTCTTCATTTAGCTGGAATTTCCTCAATTTTAGGGGCAGTAAATTTTATTACAACTGTAA
TTAATATACGATCGTCAGGAATTACTTTAGATCGACTACCTTTATTTGTTTGATCTGTAG
TTATTACAGCTATCTTATTACTTCTTTCTCTTCCTGTTTTAGCTGGAGCTATTACTATGT
TATTAACAGACCGAAACTTAAATACAT---------------------------------
--
>MK028282.1
----------------AATTTTAATTCGTGCTGAACTTAGCCACCCTGGTATATTTATTG
GGAATGACCAAATTTATAATGTAATTGTAACAGCTCATGCATTTATTATAATTTTCTTTA
TAGTAATACCAATTATAATTGGAGGATTTGGAAATTGATTAGTTCCTTTAATATTAGGAG

CCCCTGATATAGCCTTTCCTCGAATAAATAATATAAGTTTTTGAATACTACCTCCTTCAT
TGACTCTTCTATTATCAAGCTCAATAGTAGAAAATGGGGCAGGAACTGGGTGAACAGTTT
ATCCTCCTCTCTCTTCAGGAACAGCTCATGCTGGAGCTTCTGTTGATTTAGCTATTTTTT
CTCTTCATTTAGCTGGAATTTCCTCAATTTTAGGGGCAGTAAATTTTATTACAACTGTAA
TTAATATACGATCGTCAGGAATTACTTTAGATCGACTACCTTTATTTGTTTGATCTGTAG
TTATTACAGCTATCTTATTACTTCTTTCTCTTCCTGTTTTAGCTGGAGCTATTACTATGT
TATTAACAGACCGAAACTTAAATACATCTTTCTTTGATCCAATCGGAGG-----------
--
>MK028283.1
----------------AATTTTAATTCGTGCTGAACTTAGCCACCCTGGTATATTTATTG
GGAATGACCAAATTTATAATGTAATTGTAACAGCTCATGCATTTATTATAATTTTCTTTA
TAGTAATACCAATTATAATTGGAGGATTTGGAAATTGATTAGTTCCTTTAATATTAGGAG
CCCCTGATATAGCCTTTCCTCGAATAAATAATATAAGTTTTTGAATACTACCTCCTTCAT
TGACTCTTCTATTATCAAGCTCAATAGTAGAAAATGGGGCAGGAACTGGGTGAACAGTTT
ATCCTCCTCTCTCTTCAGGAACAGCTCATGCTGGAGCTTCTGTTGATTTAGCTATTTTTT
CTCTTCATTTAGCTGGAATTTCCTCAATTTTAGGGGCAGTAAATTTTATTACAACTGTAA
TTAATATACGATCGTCAGGAATTACTTTAGATCGACTACCTTTATTTGTTTGATCTGTAG
TTATTACAGCTATCTTATTACTTCTTTCTCTTCCTGTTTTAGCTGGAGCTATTACTATGT
TATTAACAGACCGAAACTTAAATACAT---------------------------------
--
>MK028284.1
-------TTCTCTAAGAATTTTAATTCGTGCTGAACTTAGCCACCCTGGTATATTTATTG
GGAATGACCAAATTTATAATGTAATTGTAACAGCTCATGCATTTATTATAATTTTCTTTA
TAGTAATACCAATTATAATTGGAGGATTTGGAAATTGATTAGTTCCTTTAATATTAGGAG
CCCCTGATATAGCCTTTCCTCGAATAAATAATATAAGTTTTTGAATACTACCTCCTTCAT
TGACTCTTCTATTATCAAGCTCAATAGTAGAAAATGGGGCAGGAACTGGGTGAACAGTTT
ATCCTCCTCTCTCTTCAGGAACAGCTCATGCTGGAGCTTCTGTTGATTTAGCTATTTTTT
CTCTTCATTTAGCTGGAATTTCCTCAATTTTAGGGGCAGTAAATTTTATTACAACTGTAA
TTAATATACGATCGTCAGGAATTACTTTAGATCGACTACCTTTATTTGTTTGATCTGTAG
TTATTACAGCTATCTTATTACTTCTTTCTCTTCCTGTTTTAGCTGGAGCTATTACTATGT
TATTAACAGACCGAAACTTAAATACATCTTTCTTTGATCCAATCG---------------
--
>MK028285.1
-----------------------------GCTGAACTTAGCCACCCTGGTATATTTATTG
GGAATGACCAAATTTATAATGTAATTGTAACAGCTCATGCATTTATTATAATTTTCTTTA
TAGTAATACCAATTATAATTGGAGGATTTGGAAATTGATTAGTTCCTTTAATATTAGGAG
CCCCTGATATAGCCTTTCCTCGAATAAATAATATAAGTTTTTGAATACTACCTCCTTCAT
TGACTCTTCTATTATCAAGCTCAATAGTAGAAAATGGGGCAGGAACTGGGTGAACAGTTT
ATCCTCCTCTCTCTTCAGGAACAGCTCATGCTGGAGCTTCTGTTGATTTAGCTATTTTTT
CTCTTCATTTAGCTGGAATTTCCTCAATTTTAGGGGCAGTAAATTTTATTACAACTGTAA
TTAATATACGATCGTCAGGAATTACTTTAGATCGACTACCTTTATTTGTTTGATCTGTAG
TTATTACAGCTATCTTATTACTTCTTTCTCTTCCTGTTTTAGCTGGAGCTATTACTATGT

--
>MK028286.1
-----------------------------------CTTAGCCACCCTGGTATATTTATTG
GGAATGACCAAATTTATAATGTAATTGTAACAGCTCATGCATTTATTATAATTTTCTTTA
TAGTAATACCAATTATAATTGGAGGATTTGGAAATTGATTAGTTCCTTTAATATTAGGAG
CCCCTGATATAGCCTTTCCTCGAATAAATAATATAAGTTTTTGAATACTACCTCCTTCAT
TGACTCTTCTATTATCAAGCTCAATAGTAGAAAATGGGGCAGGAACTGGGTGAACAGTTT
ATCCTCCTCTCTCTTCAGGAACAGCTCATGCTGGAGCTTCTGTTGATTTAGCTATTTTTT
CTCTTCATTTAGCTGGAATTTCCTCAATTTTAGGGGCAGTAAATTTTATTACAACTGTAA
TTAATATACGATCGTCAGGAATTACTTTAGATCGACTACCTTTATTTGTTTGATCTGTAG
TTATTACAGCTATCTTATTACTTCTTTCTCTTCCTGTTTTAGCTGGAGCTATTACTATGT
TATTA---
--
>MK028287.1

--ACCCTGGTATATTTATTG
GGAATGACCAAATTTATAATGTAATTGTAACAGCTCATGCATTTATTATAATTTTCTTTA
TAGTAATACCAATTATAATTGGAGGATTTGGAAATTGATTAGTTCCTTTAATATTAGGAG
CCCCTGATATAGCCTTTCCTCGAATAAATAATATAAGTTTTTGAATACTACCTCCTTCAT
TGACTCTTCTATTATCAAGCTCAATAGTAGAAAATGGGGCAGGAACTGGGTGAACAGTTT
ATCCTCCTCTCTCTTCAGGAACAGCTCATGCTGGAGCTTCTGTTGATTTAGCTATTTTTT
CTCTTCATTTAGCTGGAATTTCCTCAATTTTAGGGGCAGTAAATTTTATTACAACTGTAA
TTAATATACGATCGTCAGGAATTACTTTAGATCGACTACCTTTATTTGTTTGATCTGTAG
TTATTACAGCTATCTTATTACTTCTTTCTCTTCCTGTTTTAGCTGGAGCTATTACTATGT
TAT---
--
>MK028288.1
------------------------------------TTAGCCACCCTGGTATATTTATTG
GGAATGACCAAATTTATAATGTAATTGTAACAGCTCATGCATTTATTATAATTTTCTTTA
TAGTAATACCAATTATAATTGGAGGATTTGGAAATTGATTAGTTCCTTTAATATTAGGAG
CCCCTGATATAGCCTTTCCTCGAATAAATAATATAAGTTTTTGAATACTACCTCCTTCAT
TGACTCTTCTATTATCAAGCTCAATAGTAGAAAATGGGGCAGGAACTGGGTGAACAGTTT
ATCCTCCTCTCTCTTCAGGAACAGCTCATGCTGGAGCTTCTGTTGATTTAGCTATTTTTT
CTCTTCATTTAGCTGGAATTTCCTCAATTTTAGGGGCAGTAAATTTTATTACAACTGTAA
TTAATATACGATCGTCAGGAATTACTTTAGATCGACTACCTTTATTTGTTTGATCTGTAG
TTATTACAGCTATCTTATTACTTCTTTCTCTTCCTGTTTTAGCTGGAGCTATTACTATGT
TATTAAC---
--
>MK028289.1
---------------------------------------GCCACCCTGGTATATTTATTG
GGAATGACCAAATTTATAATGTAATTGTAACAGCTCATGCATTTATTATAATTTTCTTTA
TAGTAATACCAATTATAATTGGAGGATTTGGAAATTGATTAGTTCCTTTAATATTAGGAG
CCCCTGATATAGCCTTTCCTCGAATAAATAATATAAGTTTTTGAATACTACCTCCTTCAT
TGACTCTTCTATTATCAAGCTCAATAGTAGAAAATGGGGCAGGAACTGGGTGAACAGTTT
ATCCTCCTCTCTCTTCAGGAACAGCTCATGCTGGAGCTTCTGTTGATTTAGCTATTTTTT
CTCTTCATTTAGCTGGAATTTCCTCAATTTTAGGGGCAGTAAATTTTATTACAACTGTAA
TTAATATACGATCGTCAGGAATTACTTTAGATCGACTACCTTTATTTGTTTGATCTGTAG
TTATTACAGCTATCTTATTACTTCTTTCTCTTCCTGTTTTAGCTGGAGCTATTACTATGT
TATTA---
--
>MK028290.1
-----------------------------------CTTAGCCACCCTGGTATATTTATTG
GGAATGACCAAATTTATAATGTAATTGTAACAGCTCATGCATTTATTATAATTTTCTTTA
TAGTAATACCAATTATAATTGGAGGATTTGGAAATTGATTAGTTCCTTTAATATTAGGAG
CCCCTGATATAGCCTTTCCTCGAATAAATAATATAAGTTTTTGAATACTACCTCCTTCAT
TGACTCTTCTATTATCAAGCTCAATAGTAGAAAATGGGGCAGGAACTGGGTGAACAGTTT
ATCCTCCTCTCTCTTCAGGAACAGCTCATGCTGGAGCTTCTGTTGATTTAGCTATTTTTT
CTCTTCATTTAGCTGGAATTTCCTCAATTTTAGGGGCAGTAAATTTTATTACAACTGTAA
TTAATATACGATCGTCAGGAATTACTTTAGATCGACTACCTTTATTTGTTTGATCTGTAG
TTATTACAGCTATCTTATTACTTCTTTCTCTTCCTGTTTTAGCTGGAGCTATTACTATGT
TATTAA--
--
>MK028291.1
------------------ATTTAATTCGTGCTGAACTTAGCCACCCTGGTATATTTATTG
GGAATGACCAAATTTATAATGTAATTGTAACAGCTCATGCATTTATTATAATTTTCTTTA
TAGTAATGCCAATTATAATTGGAGGATTTGGAAATTGATTAGTTCCTTTAATATTAGGAG
CCCCTGATATAGCTTTCCCTCGAATGAATAATATAAGTTTTTGAATACTACCTCCTTCAT
TGACTCTTCTATTATCAAGCTCAATAGTAGAAAATGGAGCAGGAACTGGATGAACAGTTT
ATCCTCCTCTTTCTTCAGGAACAGCTCATGCTGGAGCTTCTGTTGATTTAGCTATTTTTT
CTCTTCATTTAGCTGGAATTTCCTCAATTTTAGGGGCAGTAAATTTTATTACAACTGTAA
TTAATATACGATCGTCAGGGATTACTTTAGATCGACTACCCTTATTTGTTTGATCTGTAG
TTATTACAGCTATCTTATTACTTCTTTCTCTTCCTGTTTTAGCTGGAGCTATTACTATAT

TATTAACAGACCGAAACTTAAATAC-----------------------------------
--
>MK028292.1
----------------AATTTTAATTCGTGCTGAACTTAGCCACCCTGGTATATTTATTG
GGAATGACCAAATTTATAATGTAATTGTAACAGCTCATGCATTTATTATAATTTTCTTTA
TAGTAATACCAATTATAATTGGAGGATTTGGAAATTGATTAGTTCCTTTAATATTAGGAG
CCCCTGATATAGCTTTTCCTCGAATAAATAATATAAGTTTCTGAATACTACCTCCTTCAT
TAACTCTACTATTATCAAGCTCAATAGTAGAAAATGGAGCAGGAACTGGATGAACAGTTT
ATCCTCCTCTCTCTTCAGGAACAGCTCATGCTGGGGCTTCTGTTGATTTAGCTATTTTTT
CTCTTCATTTAGCTGGAATTTCCTCAATTTTAGGAGCAGTAAATTTTATTACAACTGTAA
TTAATATACGATCATCAGGAATTACTTTAGATCGACTACCCTTATTTGTTTGATCTGTAG
TTATTACAGCTATCTTATTACTTCTTTCTCTTCCTGTTTTAGCTGGAGCTATTACTATAT
TATA--
--
>MK028293.1
----AACTTCTCTAAGAATTTTAATTCGTGCTGAACTTAGCCACCCTGGTATATTTATTG
GGAATGACCAAATTTATAATGTAATTGTAACAGCTCATGCATTTATTATAATTTTCTTTA
TAGTAATACCAATTATAATTGGAGGATTTGGAAATTGATTAGTTCCTTTAATATTAGGAG
CCCCTGATATAGCCTTTCCTCGAATAAATAATATAAGTTTTTGAATACTACCTCCTTCAT
TGACTCTTCTATTATCAAGCTCAATAGTAGAAAATGGGGCAGGAACTGGGTGAACAGTTT
ATCCTCCTCTCTCTTCAGGAACAGCTCATGCTGGAGCTTCTGTTGATTTAGCTATTTTTT
CTCTTCATTTAGCTGGAATTTCCTCAATTTTAGGGGCAGTAAATTTTATTACAACTGTAA
TTAATATACGATCGTCAGGAATTACTTTAGATCGACTACCTTTATTTGTTTGATCTGTAG
TTATTACAGCTATCTTATTACTTCTTTCTCTTCCTGTTTTAGCTGGAGCTATTACTATGT
TATTAACAGACCGAAAC---
--
>MK028294.1
--ATTTATTG
GGAATGACCAAATTTATAATGTAATTGTAACAGCTCATGCATTTATTATAATTTTCTTTA
TAGTAATACCAATTATAATTGGAGGATTTGGAAATTGATTAGTTCCTTTAATATTAGGAG
CCCCTGATATAGCTTTTCCTCGAATAAATAATATAAGTTTCTGAATACTACCTCCTTCAT
TAACTCTACTATTATCAAGCTCAATAGTAGAAAATGGAGCAGGAACTGGATGAACAGTTT
ATCCTCCTCTCTCTTCAGGAACAGCTCATGCTGGGGCTTCTGTTGATTTAGCTATTTTTT
CTCTTCATTTAGCTGGAATTTCCTCAATTTTAGGAGCAGTAAATTTTATTACAACTGTAA
TTAATATACGATCATCAGGAATTACTTTAGATCGACTACCCTTATTTGTTTGATCTGTAG
TTATTACAGCTATCTTATTACTTCTTTCTCTTCCTGTTTTAGCTGGAGCTAT--------

--
>MK028295.1
---TATATTTATTG
GGAATGACCAAATTTATAATGTAATTGTAACAGCTCATGCATTTATTATAATTTTCTTTA
TAGTAATACCAATTATAATTGGAGGATTTGGAAATTGATTAGTTCCTTTAATATTAGGAG
CCCCTGATATAGCTTTCCCTCGAATGAATAATATAAGTTTTTGAATACTACCTCCTTCAT
TGACTCTTCTATTATCAAGCTCAATAGTAGAAAATGGAGCAGGAACTGGGTGAACAGTTT
ATCCTCCTCTCTCTTCAGGAACAGCTCATGCTGGAGCTTCTGTTGATTTAGCTATTTTTT
CTCTTCATTTAGCTGGAATTTCCTCAATTTTAGGAGCAGTAAATTTTATTACAACTGTAA
TTAATATACGATCGTCAGGAATTACTTTAGATCGACTACCCTTATTTGTTTGATCTGTAG
TTATTACAGCTATCTTATTACTTCTTTCTCTTCCTGTTTTAGCTGGAGCTATTACTATAT
TATTAACAGACCGAAAC---
--
>MK028296.1
TCGGAACTTCTCTAAGAATTTTAATTCGTGCTGAACTTAGCCACCCTGGTATATTTATTG
GGAATGACCAAATTTATAATGTAATTGTAACAGCTCATGCATTTATTATAATTTTCTTTA
TAGTAATACCAATTATAATTGGAGGATTTGGAAATTGATTAGTTCCTTTAATATTAGGAG
CCCCTGATATAGCCTTTCCTCGAATAAATAATATAAGTTTTTGAATACTACCTCCTTCAT
TGACTCTTCTATTATCAAGCTCAATAGTAGAAAATGGGGCAGGAACTGGGTGAACAGTTT
ATCCTCCTCTCTCTTCAGGAACAGCTCATGCTGGAGCTTCTGTTGATTTAGCTATTTTTT

```
CTCTTCATTTAGCTGGAATTTCCTCAATTTTAGGGGCAGTAAATTTTATTACAACTGTAA
TTAATATACGATCGTCAGGAATTACTTTAGATCGACTACCTTTATTTGTTTGATCTGTAG
TTATTACAGCTATCTTATTACTTCTTTCTCTTCCTGTTTTAGCTGGAGCTATTACTATGT
TATTAACAGACCGAAACTTAAATACATCTTTCTTTGACCCAATCGGAGG-----------
--
>MK028297.1
-----------------------------------------------GGTATATTTATTG
GGAATGACCAAATTTATAATGTAATTGTAACAGCTCATGCATTTATTATAATTTTCTTTA
TAGTAATGCCAATTATAATTGGAGGATTTGGAAATTGATTAGTTCCTTTAATATTAGGAG
CCCCTGATATAGCTTTCCCTCGAATGAATAATATAAGTTTTTGAATACTACCTCCTTCAT
TGACTCTTCTATTATCAAGCTCAATAGTAGAAAATGGAGCAGGAACTGGATGAACAGTTT
ATCCTCCTCTTTCTTCAGGAACAGCTCATGCTGGAGCTTCTGTTGATTTAGCTATTTTTT
CTCTTCATTTAGCTGGAATTTCCTCAATTTTAGGGGCAGTAAATTTTATTACAACTGTAA
TTAATATACGATCGTCAGGGATTACTTTAGATCGACTACCCTTATTTGTTTGATCTGTAG
TTATTACAGCTATCTTATTACTTCTTTCTCTTCCTGTTTTAGCTGGAGCTATTACTATAT
TATTAACAGACCGAAACTTAAATA------------------------------------
--
>MK028298.1
TCGGAACTTCTCTAAGAATTTTAATTCGTGCTGAACTTAGCCACCCTGGTATATTTATTG
GGAATGACCAAATTTATAATGTAATTGTAACAGCTCATGCATTTATTATAATTTTCTTTA
TAGTAATACCAATTATAATTGGAGGATTTGGAAATTGATTAGTTCCTTTAATATTAGGAG
CCCCTGATATAGCCTTTCCTCGAATAAATAATATAAGTTTTTGAATACTACCTCCTTCAT
TGACTCTTCTATTATCAAGCTCAATAGTAGAAAATGGGGCAGGAACTGGGTGAACAGTTT
ATCCTCCTCTCTCTTCAGGAACAGCTCATGCTGGAGCTTCTGTTGATTTAGCTATTTTTT
CTCTTCATTTAGCTGGAATTTCCTCAATTTTAGGGGCAGTAAATTTTATTACAACTGTAA
TTAATATACGATCGTCAGGAATTACTTTAGATCGACTACCTTTATTTGTTTGATCTGTAG
TTATTACAGCTATCTTATTACTTCTTTCTCTTCCTGTTTTAGCTGGAGCTATTACTATGT
TATTAACAGACCGAAACTTAAATACATCTTTCTTT-------------------------
--
>MK028299.1
TCGGAACTTCTCTAAGAATTTTAATTCGTGCTGAACTTAGCCACCCTGGTATATTTATTG
GGAATGACCAAATTTATAATGTAATTGTAACAGCTCATGCATTTATTATAATTTTCTTTA
TAGTAATGCCAATTATAATTGGAGGATTTGGAAATTGATTAGTTCCTTTAATATTAGGAG
CCCCTGATATAGCTTTCCCTCGAATGAATAATATAAGTTTTTGAATACTACCTCCTTCAT
TGACTCTTCTATTATCAAGCTCAATAGTAGAAAATGGAGCAGGAACTGGATGAACAGTTT
ATCCTCCTCTTTCTTCAGGAACAGCTCATGCTGGAGCTTCTGTTGATTTAGCTATTTTTT
CTCTTCATTTAGCTGGAATTTCCTCAATTTTAGGGGCAGTAAATTTTATTACAACTGTAA
TTAATATACGATCGTCAGGAATTACTTTAGATCGACTACCCTTATTTGTTTGATCTGTAG
TTATTACAGCTATCTTATTACTTCTTTCTCTTCCTGTTTTAGCTGGAGCTATTACTATAT
TATTAACAGACCGAAACTTAAATACATCTTT-----------------------------
--
>MK028300.1
--------TCTCTAAGAATTTTAATTCGTGCTGAACTTAGCCACCCTGGTATATTTATTG
GGAATGACCAAATTTATAATGTAATTGTAACAGCTCATGCATTTATTATAATTTTCTTTA
TAGTAATGCCAATTATAATTGGAGGATTTGGAAATTGATTAGTTCCTTTAATATTAGGAG
CCCCTGATATAGCTTTCCCTCGAATGAATAATATAAGTTTTTGAATACTACCTCCTTCAT
TGACTCTTCTATTATCAAGCTCAATAGTAGAAAATGGAGCAGGAACTGGATGAACAGTTT
ATCCTCCTCTTTCTTCAGGAACAGCTCATGCTGGAGCTTCTGTTGATTTAGCTATTTTTT
CTCTTCATTTAGCTGGAATTTCCTCAATTTTAGGGGCAGTAAATTTTATTACAACTGTAA
TTAATATACGATCGTCAGGAATTACTTTAGATCGACTACCCTTATTTGTTTGATCTGTAG
TTATTACAGCTATCTTATTACTTCTTTCTCTTCCTGTTTTAGCTGGAGCTATTACTATAT
TATTAACAGACCGAAACTTAAATACAT---------------------------------
--
>MK300216.1
TCGGAACTTCTCTAAGAATTTTAATTCGTGCTGAACTTAGCCACCCTGGTATATTTATTG
GGAATGACCAAATTTATAATGTAATTGTAACAGCTCATGCATTTATTATAATTTTCTTTA
TAGTAATACCAATTATAATTGGAGGATTTGGAAATTGATTAGTTCCTTTAATATTAGGAG
```

CCCCTGATATAGCTTTCCCTCGAATAAATAATATAAGTTTTTGAATACTACCTCCTTCAT
TGACTCTTCTATTATCAAGCTCAATAGTAGAAAATGGGGCAGGAACTGGGTGAACAGTTT
ATCCTCCTCTCTCTTCAGGAACAGCTCATGCTGGAGCTTCTGTTGATTTAGCTATTTTTT
CTCTTCATTTAGCTGGAATTTCCTCAATTTTAGGGGCAGTAAATTTTATTACAACTGTAA
TTAATATACGATCGTCAGGGATTACTTTAGATCGACTACCCTTATTTGTTTGATCTGTAG
TTATTACAGCTATCTTATTACTTCTTTCTCTTCCTGTTTTAGCTGGAGCTATTACTATAT
TATTAACAGACCGAAACTTAAATACATCTTTCTTTGATCCAATCGGAGGAGGAGACCCTA
TT
>MK300217.1
TCGGAACTTCTCTAAGAATTTTAATTCGTGCTGAACTTAGCCACCCTGGTATATTTATTG
GGAATGACCAAATTTATAATGTAATTGTAACAGCTCATGCATTTATTATAATTTTCTTTA
TAGTAATGCCAATTATAATTGGAGGATTTGGAAATTGATTAGTTCCTTTAATATTAGGAG
CCCCTGATATAGCTTTCCCTCGAATGAATAATATAAGTTTTTGAATACTACCTCCTTCAT
TGACTCTTCTATTATCAAGCTCAATAGTAGAAAATGGAGCAGGAACTGGGTGAACAGTTT
ATCCTCCTCTCTCTTCAGGAACAGCTCATGCTGGGGCTTCTGTTGATTTAGCTATTTTTT
CTCTTCATTTAGCTGGAATTTCCTCAATTTTAGGGGCAGTAAATTTTATTACAACTGTAA
TTAATATACGATCGTCAGGGATTACTTTAGATCGACTACCCTTATTTGTTTGATCTGTAG
TTATTACAGCTATCTTATTACTTCTTTCTCTTCCTGTTTTAGCTGGAGCTATTACTATAT
TATTAACAGACCGAAACTTAAATACATCTTTCTTTGATCCAATCGGAGGGGGAGACCCTA
TT
>MK300218.1
TCGGAACTTCTCTAAGAATTTTAATTCGTGCTGAACTTAGCCACCCTGGTATATTTATTG
GGAATGACCAAATTTATAATGTAATTGTAACAGCTCATGCATTTATTATAATTTTCTTTA
TAGTAATACCAATTATAATTGGAGGATTTGGAAATTGATTAGTTCCTTTAATATTAGGAG
CCCCTGATATAGCTTTCCCTCGAATAAATAATATAAGTTTTTGAATACTACCTCCTTCAT
TGACTCTTCTATTATCAAGCTCAATAGTAGAAAATGGAGCAGGAACTGGGTGAACAGTTT
ATCCTCCTCTCTCTTCAGGAACAGCTCATGCTGGAGCTTCTGTTGATTTAGCTATTTTTT
CTCTTCATTTAGCTGGAATTTCCTCAATTTTAGGGGCAGTAAATTTTATTACAACTGTAA
TTAATATACGATCGTCAGGGATTACTTTAGATCGACTACCCTTATTTGTTTGATCTGTAG
TTATTACAGCTATCTTATTACTTCTTTCTCTTCCTGTTTTAGCTGGAGCTATTACTATAT
TATTAACAGACCGAAACTTAAATACATCTTTCTTTGATCCAATCGGAGGAGGAGACCCTA
TT
>MK300219.1
TCGGAACTTCTCTAAGAATTTTAATTCGTGCTGAACTTAGCCACCCTGGTATATTTATTG
GGAATGACCAAATTTACAATGTAATTGTAACAGCTCATGCATTTATTATAATTTTCTTTA
TAGTAATGCCAATTATAATTGGAGGATTTGGAAATTGATTAGTTCCTTTAATATTAGGAG
CCCCTGATATAGCTTTCCCTCGAATGAATAATATAAGTTTTTGAATACTACCTCCTTCAT
TGACTCTTCTATTATCAAGCTCAATAGTAGAAAATGGAGCAGGAACTGGGTGAACAGTTT
ATCCTCCTCTCTCTTCAGGAACAGCTCATGCTGGAGCTTCTGTTGATTTAGCTATTTTTT
CTCTTCATTTAGCTGGAATTTCCTCAATTTTAGGGGCAGTAAATTTTATTACAACTGTAA
TTAATATACGATCGTCAGGGATTACTTTAGATCGACTACCTTTATTTGTTTGATCTGTAG
TTATTACAGCTATCTTATTACTTCTTTCTCTTCCTGTTTTAGCTGGAGCTATTACTATAT
TATTAACAGACCGAAACTTAAATACATCTTTCTTTGATCCAATCGGAGGAGGAGACCCTA
TT
>MK300220.1
TCGGAACTTCTCTAAGAATTTTAATTCGTGCTGAACTTAGCCACCCTGGTATATTTATTG
GGAATGACCAAATTTATAATGTAATTGTAACAGCTCATGCATTTATTATAATTTTCTTTA
TAGTAATGCCAATTATAATTGGAGGATTTGGAAATTGATTAGTTCCTTTAATATTAGGAG
CCCCTGATATAGCTTTCCCTCGAATGAATAATATAAGTTTTTGAATACTACCTCCTTCAT
TGACTCTTCTATTATCAAGCTCAATAGTAGAAAATGGAGCAGGAACTGGGTGAACAGTTT
ATCCTCCTCTCTCTTCAGGAACAGCTCATGCTGGAGCTTCTGTTGATTTAGCTATTTTTT
CTCTTCATTTAGCTGGAATTTCCTCAATTTTAGGGGCAGTAAATTTTATTACAACTGTAA
TTAATATACGATCGTCAGGAATTACTTTAGATCGACTACCCTTATTTGTTTGATCTGTAG
TTATTACAGCTATTTTATTACTTCTTTCTCTCCCTGTTTTAGCTGGAGCTATTACTATAT
TATTAACAGACCGAAACTTAAATACATCTTTCTTTGATCCAATCGGAGGGGGAGACCCTA
TT
>MK300221.1

TCGGAACTTCTCTAAGAATTTTAATTCGTGCTGAACTTAGCCACCCTGGTATATTTATTG
GGAATGACCAAATTTATAATGTAATTGTAACAGCTCATGCATTTATTATAATTTTCTTTA
TAGTAATACCAATTATAATTGGAGGATTTGGAAATTGATTAGTTCCTTTAATATTAGGAG
CCCCTGATATAGCTTTCCCTCGAATGAATAATATAAGTTTTTGAATACTACCTCCTTCAT
TGACTCTTCTATTATCAAGCTCAATAGTAGAAAATGGAGCAGGAACTGGGTGAACAGTTT
ATCCTCCTCTCTCTTCAGGAACAGCTCATGCTGGAGCTTCTGTTGATTTAGCTATTTTTT
CTCTTCATTTAGCTGGAATTTCCTCAATTTTAGGGGCAGTAAATTTTATTACAACTGTAA
TTAATATACGATCGTCAGGGATTACTTTAGATCGACTACCTTTATTTGTTTGATCTGTAG
TTATTACAGCTATCTTATTACTTCTTTCTCTTCCTGTTTTAGCTGGAGCTATTACTATAT
TATTAACAGACCGAAACTTAAATACATCTTTCTTTGATCCAATCGGAGGAGGAGACCCTA
TT
>MK300222.1
TCGGAACTTCTCTAAGAATTTTAATTCGTGCTGAACTTAGCCACCCTGGTATATTTATTG
GGAATGACCAAATTTATAATGTAATTGTAACAGCTCATGCATTTATTATAATTTTCTTTA
TAGTAATACCAATTATAATTGGAGGATTTGGAAATTGATTAGTTCCTTTAATATTAGGAG
CCCCTGATATAGCTTTCCCTCGAATAAATAATATAAGTTTTTGAATACTACCTCCTTCAT
TGACTCTTCTATTATCAAGCTCAATAGTAGAAAATGGGGCAGGAACTGGGTGAACAGTTT
ATCCTCCTCTCTCTTCAGGAACAGCTCATGCTGGAGCTTCTGTTGATTTAGCTATTTTTT
CTCTTCATTTAGCTGGAATTTCCTCAATTTTAGGGGCAGTAAATTTTATTACAACTGTAA
TTAATATACGATCGTCAGGGATTACTTTAGATCGACTACCCTTATTTGTTTGATCTGTAG
TTATTACAGCTATCTTATTACTTCTTTCTCTTCCTGTTTTAGCTGGAGCTATTACTATAT
TATTAACAGACCGAAACTTAAATACATCTTTCTTTGACCCAATCGGAGGAGGAGACCCTA
TT
>MK300223.1
TCGGAACTTCTCTAAGAATTTTAATTCGTGCTGAACTTAGCCACCCTGGTATATTTATTG
GGAATGACCAAATTTATAATGTAATTGTAACAGCTCATGCATTTATTATAATTTTCTTTA
TAGTAATGCCAATTATAATTGGAGGATTTGGAAATTGATTAGTTCCTTTAATATTAGGAG
CCCCTGATATAGCTTTCCCTCGAATGAATAATATAAGTTTTTGAATACTACCTCCTTCAT
TGACTCTTCTATTATCAAGCTCAATAGTAGAAAATGGAGCAGGAACTGGGTGAACAGTTT
ATCCTCCTCTCTCTTCAGGAACAGCTCATGCTGGAGCTTCTGTTGATTTAGCTATTTTTT
CTCTTCATTTAGCTGGAATTTCCTCAATTTTAGGGGCAGTAAATTTTATTACAACTGTAA
TTAATATACGATCGTCAGGGATTACTTTAGATCGACTACCCTTATTTGTTTGATCTGTAG
TTATTACAGCTATTTTATTACTTCTTTCTCTTCCTGTTTTAGCTGGAGCTATTACTATAT
TATTAACAGACCGAAACTTAAATACATCTTTCTTTGATCCAATCGGAGGGGGAGACCCTA
TT
>MK300224.1
TCGGAACTTCTCTAAGAATTTTAATTCGTGCTGAACTTAGCCACCCTGGTATATTTATTG
GGAATGACCAAATTTATAATGTAATTGTAACAGCTCATGCATTTATTATAATTTTCTTTA
TAGTAATGCCAATTATAATTGGAGGATTTGGAAATTGATTAGTTCCTTTAATATTAGGAG
CCCCTGATATAGCTTTCCCTCGAATGAATAATATAAGTTTTTGAATACTACCTCCTTCAT
TGACTCTTCTATTATCAAGCTCAATAGTAGAAAATGGGGCAGGAACTGGATGAACAGTTT
ATCCTCCTCTCTCTTCAGGAACAGCTCATGCTGGAGCTTCTGTTGATTTAGCTATTTTTT
CTCTTCATTTAGCTGGAATTTCCTCAATTTTAGGGGCAGTAAATTTTATTACAACTGTAA
TTAATATACGATCGTCAGGGATTACTTTAGATCGACTACCCTTATTTGTTTGATCTGTAG
TTATTACAGCTATCTTATTACTTCTTTCTCTTCCTGTTTTAGCTGGAGCTATTACTATAT
TATTAACAGACCGAAACTTAAATACATCTTTCTTTGATCCAATCGGAGGGGGAGACCCTA
TT
>MK300226.1
TCGGAACTTCTCTAAGAATTTTAATTCGTGCTGAACTTAGCCACCCTGGTATATTTATTG
GGAATGACCAAATTTATAATGTAATTGTAACAGCTCATGCATTTATTATAATTTTCTTTA
TAGTAATGCCAATTATAATTGGAGGATTTGGAAATTGATTAGTTCCTTTAATATTAGGAG
CCCCTGATATAGCTTTCCCTCGAATGAATAATATAAGTTTTTGAATACTACCTCCTTCAT
TGACTCTTCTATTATCAAGCTCAATAGTAGAAAATGGAGCAGGAACTGGGTGAACAGTTT
ATCCTCCTCTCTCTTCAGGAACAGCTCATGCTGGAGCTTCTGTTGATTTAGCTATTTTTT
CTCTTCATTTAGCTGGAATTTCCTCAATTTTAGGGGCAGTAAATTTTATTACAACTGTAA
TTAATATACGATCGTCAGGGATTACTTTAGATCGGCTACCCTTATTTGTTTGATCTGTAG
TTATTACAGCTATCTTATTACTTCTTTCTCTTCCTGTTTTAGCTGGAGCTATTACTATAT

TATTAACAGACCGAAACTTAAATACATCTTTCTTTGATCCAATCGGAGGGGGAGACCCTA
TT
>MK300227.1
TCGGAACTTCTCTAAGAATTTTAATTCGTGCTGAACTTAGCCACCCTGGTATATTTATTG
GGAATGACCAAATTTATAATGTAATTGTAACAGCTCATGCATTTATTATAATTTTCTTTA
TAGTAATGCCAATTATAATTGGAGGATTTGGAAATTGATTAGTTCCTTTAATATTAGGAG
CCCCTGATATAGCTTTCCCTCGAATGAATAATATAAGTTTTTGAATACTACCTCCTTCAT
TGACTCTTCTATTATCAAGCTCAATAGTAGAAAATGGAGCAGGAACTGGGTGAACAGTTT
ATCCTCCTCTCTCTTCAGGAACAGCTCATGCTGGAGCTTCTGTTGATTTAGCTATTTTTT
CTCTTCATTTAGCTGGAATTTCCTCAATTTTAGGGGCAGTAAATTTTATTACAACTGTAA
TTAATATACGATCATCAGGGATTACTTTAGATCGACTACCCTTATTTGTTTGATCTGTAG
TTATTACAGCTATCTTATTACTTCTTTCTCTTCCTGTTTTAGCTGGAGCTATTACTATAT
TATTAACAGACCGAAACTTAAATACATCTTTCTTTGATCCAATCGGAGGGGGAGACCCTA
TT
>MK300228.1
TCGGAACTTCTCTAAGAATTTTAATTCGAGCTGAACTTAGCCACCCTGGTATATTTATTG
GGAATGACCAAATTTATAATGTAATTGTAACAGCTCATGCATTTATTATAATTTTCTTTA
TAGTAATGCCAATTATAATTGGAGGATTTGGAAATTGATTAGTTCCTTTAATATTAGGAG
CCCCTGATATAGCTTTCCCTCGAATGAATAATATAAGTTTTTGAATACTACCTCCTTCAT
TGACTCTTCTATTATCAAGCTCAATAGTAGAAAATGGAGCAGGAACTGGGTGAACAGTTT
ATCCTCCTCTCTCTTCAGGAACAGCTCATGCTGGAGCTTCTGTTGATTTAGCTATTTTTT
CTCTTCATTTAGCTGGAATTTCCTCAATTTTAGGGGCAGTAAATTTTATTACAACTGTAA
TTAATATACGATCTTCAGGGATTACTTTAGATCGACTACCCTTATTTGTTTGATCTGTAG
TAATTACAGCTATCTTATTACTTCTTTCTCTTCCTGTTTTAGCTGGAGCTATTACTATAT
TATTAACAGATCGAAACTTAAATACATCATTCTTTGATCCAATCGGAGGAGGAGACCCTA
TT
>MK300229.1
TCGGAACTTCTCTAAGAATTTTAATTCGTGCTGAACTTAGCCACCCTGGTATATTTATTG
GGAATGACCAAATTTATAATGTAATTGTAACAGCTCATGCATTTATTATAATTTTCTTTA
TAGTAATGCCAATTATAATTGGAGGATTTGGAAATTGATTAGTTCCTTTAATATTAGGAG
CCCCTGATATAGCTTTCCCTCGAATGAATAATATAAGTTTTTGAATACTACCTCCTTCAT
TGACTCTTCTATTATCAAGCTCAATAGTAGAAAATGGAGCAGGAACTGGGTGAACAGTTT
ATCCTCCTCTCTCTTCAGGAACAGCTCATGCTGGAGCTTCTGTTGATTTAGCTATTTTTT
CTCTTCATTTAGCTGGAATTTCCTCAATTTTAGGGGCAGTAAATTTTATTACAACTGTAA
TTAATATACGATCGTCAGGGATTACTTTAGATCGACTACCCTTATTTGTTTGATCTGTAG
TTATTACAGCTATCTTATTACTTCTTTCTCTTCCTGTTTTAGCCGGAGCTATTACTATAT
TATTAACAGACCGAAACTTAAATACATCTTTCTTTGATCCAGCAGGAGGAGGTGATCCAA
TT
>KM613046.1
TCGGAACTTCTCTAAGAATTTTAATTCGTGCTGAACTTAGCCACCCTGGTATATTTATTG
GGAATGACCAAATTTATAATGTAATTGTAACAGCTCATGCATTTATTATAATTTTCTTTA
TAGTAATACCAATTATAATTGGAGGATTTGGAAATTGATTAGTTCCTTTAATATTAGGAG
CCCCTGATATAGCCTTTCCTCGAATAAATAATATAAGTTTTTGAATACTACCTCCTTCAT
TGACTCTTCTATTATCAAGCTCAATAGTAGAAAATGGAGCAGGAACTGGGTGAACAGTGT
ATCCTCCTCTCTCTTCAGGAACAGCTCATGCTGGAGCTTCTGTTGATTTAGCTATTTTTT
CTCTTCATTTAGCTGGAATTTCCTCAATTTTAGGGGCAGTAAATTTTATTACAACTGTAA
TTAATATACGATCGTCAGGAATTACTTTAGACCGACTACCTTTATTTGTTTGATCTGTAG
TTATTACAGCTATCTTATTACTTCTTTCTCTTCCTGTTTTAGCTGGAGCTATTACTATGT
TATTAACAGACCGAAACTTAAATACATCTTTCTTCGATCCAATCGGAGGAGGAGATCCTA
TT
>KM613047.1
TCGGAACTTCTCTAAGAATTTTAATTCGTGCTGAACTTAGCCACCCTGGTATATTTATTG
GGAATGACCAAATTTATAATGTAATTGTAACAGCTCATGCATTTATTATAATTTTCTTTA
TAGTAATACCAATTATAATTGGAGGATTTGGAAATTGATTAGTTCCTTTAATATTAGGAG
CCCCTGATATAGCCTTTCCTCGAATAAATAATATAAGTTTTTGAATACTACCTCCTTCAT
TGACTCTTCTATTATCAAGCTCAATAGTAGAAAATGGAGCAGGAACTGGGTGAACAGTGT
ATCCTCCTCTCTCTTCAGGAACAGCTCATGCTGGAGCTTCTGTTGATTTAGCTATTTTTT

CTCTTCATTTAGCTGGAATTTCCTCAATTTTAGGGGCAGTAAATTTTATTACAACTGTAA
TTAATATACGATCGTCAGGAATTACTTTAGACCGACTACCTTTATTTGTTTGATCTGTAG
TTATTACAGCTATCTTATTACTTCTTTCTCTTCCTGTTTTAGCTGGAGCTATTACTATGT
TATTAACAGACCGAAACTTAAATACATCTTTCTTCGATCCAATCGGAGGAGGAGATCCTA
TT
>KM613048.1
TCGGAACTTCTCTAAGAATTTTAATTCGTGCTGAACTTAGCCACCCTGGTATATTTATTG
GGAATGACCAAATTTATAATGTAATTGTAACAGCTCATGCATTTATTATAATTTTCTTTA
TAGTAATGCCAATTATAATTGGAGGATTTGGAAATTGATTAGTTCCTTTAATATTAGGAG
CCCCTGATATAGCTTTCCCTCGAATGAATAATATAAGTTTTTGAATACTACCTCCTTCAT
TGACTCTTCTATTATCAAGCTCAATAGTAGAAAATGGGGCAGGAACTGGGTGAACAGTTT
ATCCTCCTCTCTCTTCAGGAACAGCTCATGCTGGAGCTTCTGTTGATTTAGCTATTTTTT
CTCTTCATTTAGCTGGAATTTCCTCAATTTTAGGGGCAGTAAATTTTATTACAACTGTAA
TTAATATGCGATCGTCAGGGATTACTTTAGATCGACTACCCTTATTTGTTTGATCTGTAG
TTATTACAGCTATCTTATTACTTCTTTCTCTTCCTGTTTTAGCTGGAGCTATTACTATAT
TATTAACAGACCGAAACTTAAATACATCTTTCTTTGATCCAATCGGAGGGGGAGACCCTA
TT
>KM613049.1
TCGGAACTTCTCTAAGAATTTTAATTCGTGCTGAACTTAGCCACCCTGGTATATTTATTG
GGAATGACCAAATTTATAATGTAATTGTAACAGCTCATGCATTTATTATAATTTTCTTTA
TAGTAATACCAATTATAATTGGAGGATTTGGAAATTGATTAGTTCCTTTAATATTAGGAG
CCCCTGATATAGCCTTTCCTCGAATAAATAATATAAGTTTTTGAATACTACCTCCTTCAT
TGACTCTTCTATTATCAAGCTCAATAGTAGAAAATGGAGCAGGAACTGGGTGAACAGTGT
ATCCTCCTCTCTCTTCAGGAACAGCTCATGCTGGAGCTTCTGTTGATTTAGCTATTTTTT
CTCTTCATTTAGCTGGAATTTCCTCAATTTTAGGGGCAGTAAATTTTATTACAACTGTAA
TTAATATACGATCGTCAGGAATTACTTTAGACCGACTACCTTTATTTGTTTGATCTGTAG
TTATTACAGCTATCTTATTACTTCTTTCTCTTCCTGTTTTAGCTGGAGCTATTACTATGT
TATTAACAGACCGAAACTTAAATACATCTTTCTTCGATCCAATCGGAGGAGGAGATCCTA
TT
>KM613050.1
TCGGAACTTCTCTAAGAATTTTAATTCGTGCTGAACTTAGCCACCCTGGTATATTTATTG
GGAATGACCAAATTTATAATGTAATTGTAACAGCTCATGCATTTATTATAATTTTCTTTA
TAGTAATACCAATTATAATTGGAGGATTTGGAAATTGATTAGTTCCTTTAATATTAGGAG
CCCCTGATATAGCCTTTCCTCGAATAAATAATATAAGTTTTTGAATACTACCTCCTTCAT
TGACTCTTCTATTATCAAGCTCAATAGTAGAAAATGGAGCAGGAACTGGGTGAACAGTGT
ATCCTCCTCTCTCTTCAGGAACAGCTCATGCTGGAGCTTCTGTTGATTTAGCTATTTTTT
CTCTTCATTTAGCTGGAATTTCCTCAATTTTAGGGGCAGTAAATTTTATTACAACTGTAA
TTAATATACGATCGTCAGGAATTACTTTAGACCGACTACCTTTATTTGTTTGATCTGTAG
TTATTACAGCTATCTTATTACTTCTTTCTCTTCCTGTTTTAGCTGGAGCTATTACTATGT
TATTAACAGACCGAAACTTAAATACATCTTTCTTCGATCCAATCGGAGGAGGAGATCCTA
TT
>KM613051.1
TCGGAACTTCTCTAAGAATTTTAATTCGTGCTGAACTTAGCCACCCTGGTATATTTATTG
GGAATGACCAAATTTATAATGTAATTGTAACAGCTCATGCATTTATTATAATTTTCTTTA
TAGTAATGCCAATTATAATTGGAGGATTTGGAAATTGATTAGTTCCTTTAATATTAGGAG
CCCCTGATATAGCTTTCCCTCGAATGAATAATATAAGTTTTTGAATACTACCTCCTTCAT
TGACTCTTCTATTATCAAGCTCAATAGTAGAAAATGGAGCAGGAACTGGGTGAACAGTTT
ATCCTCCTCTCTCTTCAGGAACAGCTCATGCTGGAGCTTCTGTTGATTTAGCTATTTTTT
CTCTTCATTTAGCTGGAATTTCCTCAATTTTAGGGGCAGTAAATTTTATTACAACTGTAA
TTAATATACGATCGTCAGGAATTACTTTAGATCGACTACCCTTATTTGTTTGATCTGTAG
TTATTACAGCTATCTTATTACTTCTTTCTCTTCCTGTTTTAGCTGGAGCTATTACTATAT
TATTAACAGACCGAAACTTAAATACATCTTTCTTTGATCCAATCGGAGGGGGAGACCCTA
TT
>KM613052.1
TCGGAACTTCTCTAAGAATTTTAATTCGTGCTGAACTTAGCCACCCTGGTATATTTATTG
GGAATGACCAAATTTATAATGTAATTGTAACAGCTCATGCATTTATTATAATTTTCTTTA
TAGTAATACCAATTATAATTGGAGGATTTGGAAATTGATTAGTTCCTTTAATATTAGGAG

CCCCTGATATAGCCTTTCCTCGAATAAATAATATAAGTTTTTGAATACTACCTCCTTCAT
TGACTCTTCTATTATCAAGCTCAATAGTAGAAAATGGGGCAGGAACTGGGTGAACAGTTT
ATCCTCCTCTCTCTTCAGGAACAGCTCATGCTGGAGCTTCTGTTGATTTAGCTATTTTTT
CTCTTCATTTAGCTGGAATTTCCTCAATTTTAGGGGCAGTAAATTTTATTACAACTGTAA
TTAATATACGATCGTCAGGAATTACTTTAGACCGACTACCTTTATTTGTTTGATCTGTAG
TTATTACAGCTATCTTATTACTTCTTTCTCTTCCTGTTTTAGCTGGAGCTATTACTATGT
TATTAACAGACCGAAACTTAAATACATCTTTCTTTGATCCAATCGGAGGAGGAGATCCTA
TT
>KM613053.1
TCGGAACTTCTCTAAGAATTTTAATTCGTGCTGAACTTAGCCACCCTGGTATATTTATTG
GGAATGACCAAATTTATAATGTAATTGTAACAGCTCATGCATTTATTATAATTTTCTTTA
TAGTAATACCAATTATAATTGGAGGATTTGGAAATTGATTAGTTCCTTTAATATTAGGAG
CCCCTGATATAGCCTTTCCTCGAATAAATAATATAAGTTTTTGAATACTACCTCCTTCAT
TGACTCTTCTATTATCAAGCTCAATAGTAGAAAATGGGGCAGGAACTGGGTGAACAGTTT
ATCCTCCTCTCTCTTCAGGAACAGCTCATGCTGGAGCTTCTGTTGATTTAGCTATTTTTT
CTCTTCATTTAGCTGGAATTTCCTCAATTTTAGGGGCAGTAAATTTTATTACAACTGTAA
TTAATATACGATCGTCAGGAATTACTTTAGACCGACTACCTTTATTTGTTTGATCTGTAG
TTATTACAGCTATCTTATTACTTCTTTCTCTTCCTGTTTTAGCTGGAGCTATTACTATGT
TATTAACAGACCGAAACTTAAATACATCTTTCTTTGATCCAATCGGAGGAGGAGATCCTA
TT
>KM613054.1
TCGGAACTTCTCTAAGAATTTTAATTCGTGCTGAACTTAGCCACCCTGGTATATTTATTG
GGAATGACCAAATTTATAATGTAATTGTAACAGCTCATGCATTTATTATAATTTTCTTTA
TAGTAATACCAATTATAATTGGAGGATTTGGAAATTGATTAGTTCCTTTAATATTAGGAG
CCCCTGATATAGCCTTTCCTCGAATAAATAATATAAGTTTTTGAATACTACCTCCTTCAT
TGACTCTTCTATTATCAAGCTCAATAGTAGAAAATGGAGCAGGAACTGGGTGAACAGTGT
ATCCTCCTCTCTCTTCAGGAACAGCTCATGCTGGAGCTTCTGTTGATTTAGCTATTTTTT
CTCTTCATTTAGCTGGAATTTCCTCAATTTTAGGGGCAGTAAATTTTATTACAACTGTAA
TTAATATACGATCGTCAGGAATTACTTTAGACCGACTACCTTTATTTGTTTGATCTGTAG
TTATTACAGCTATCTTATTACTTCTTTCTCTTCCTGTTTTAGCTGGAGCTATTACTATGT
TATTAACAGACCGAAACTTAAATACATCTTTCTTCGATCCAATCGGAGGAGGAGATCCTA
TT
>KM613055.1
TCGGAACTTCTCTAAGAATTTTAATTCGTGCTGAACTTAGCCACCCTGGTATATTTATTG
GGAATGACCAAATTTATAATGTAATTGTAACAGCTCATGCATTTATTATAATTTTCTTTA
TAGTAATACCAATTATAATTGGAGGATTTGGAAATTGATTAGTTCCTTTAATATTAGGAG
CCCCTGATATAGCCTTTCCTCGAATAAATAATATAAGTTTTTGAATACTACCTCCTTCAT
TGACTCTTCTATTATCAAGCTCAATAGTAGAAAATGGAGCAGGAACTGGGTGAACAGTGT
ATCCTCCTCTCTCTTCAGGAACAGCTCATGCTGGAGCTTCTGTTGATTTAGCTATTTTTT
CTCTTCATTTAGCTGGAATTTCCTCAATTTTAGGGGCAGTAAATTTTATTACAACTGTAA
TTAATATACGATCGTCAGGAATTACTTTAGACCGACTACCTTTATTTGTTTGATCTGTAG
TTATTACAGCTATCTTATTACTTCTTTCTCTTCCTGTTTTAGCTGGAGCTATTACTATGT
TATTAACAGACCGAAACTTAAATACATCTTTCTTTGATCCAATCGGAGGAGGAGATCCTA
TT
>KM613056.1
TCGGAACTTCTCTAAGAATTTTAATTCGTGCTGAACTTAGCCACCCTGGTATATTTATTG
GGAATGACCAAATTTATAATGTAATTGTAACAGCTCATGCATTTATTATAATTTTCTTTA
TAGTAATACCAATTATAATTGGAGGATTTGGAAATTGATTAGTTCCTTTAATATTAGGAG
CCCCTGATATAGCCTTTCCTCGAATAAATAATATAAGTTTTTGAATACTACCTCCTTCAT
TGACTCTTCTATTATCAAGCTCAATAGTAGAAAATGGAGCAGGAACTGGGTGAACAGTGT
ATCCTCCTCTCTCTTCAGGAACAGCTCATGCTGGAGCTTCTGTTGATTTAGCTATTTTTT
CTCTTCATTTAGCTGGAATTTCCTCAATTTTAGGGGCAGTAAATTTTATTACAACTGTAA
TTAATATACGATCGTCAGGAATTACTTTAGACCGACTACCTTTATTTGTTTGATCTGTAG
TTATTACAGCTATCTTATTACTTCTTTCTCTTCCTGTTTTAGCTGGAGCTATTACTATGT
TATTAACAGACCGAAACTTAAATACATCTTTCTTCGATCCAATCGGAGGAGGAGATCCTA
TT
>KM613057.1

TCGGAACTTCTCTAAGAATTTTAATTCGTGCTGAACTTAGCCACCCTGGTATATTTATTG
GGAATGACCAAATTTATAATGTAATTGTAACAGCTCATGCATTTATTATAATTTTCTTTA
TAGTAATACCAATTATAATTGGAGGATTTGGAAATTGATTAGTTCCTTTAATATTAGGAG
CCCCTGATATAGCCTTTCCTCGAATAAATAATATAAGTTTTTGAATACTACCTCCTTCAT
TGACTCTTCTATTATCAAGCTCAATAGTAGAAAATGGGGCAGGAACTGGGTGAACAGTTT
ATCCTCCTCTCTCTTCAGGAACAGCTCATGCTGGAGCTTCTGTTGATTTAGCTATTTTTT
CTCTTCATTTAGCTGGAATTTCCTCAATTTTAGGGGCAGTAAATTTTATTACAACTGTAA
TTAATATACGATCGTCAGGAATTACTTTAGACCGACTACCTTTATTTGTTTGATCTGTAG
TTATTACAGCTATCTTATTACTTCTTTCTCTTCCTGTTTTAGCTGGAGCTATTACTATGT
TATTAACAGACCGAAACTTAAATACATCTTTCTTTGATCCAATCGGAGGAGGAGATCCTA
TT
>KM613058.1
TCGGAACTTCTCTAAGAATTTTAATTCGTGCTGAACTTAGCCACCCTGGTATATTTATTG
GGAATGACCAAATTTATAATGTAATTGTAACAGCTCATGCATTTATTATAATTTTCTTTA
TAGTAATACCAATTATAATTGGAGGATTTGGAAATTGATTAGTTCCTTTAATATTAGGAG
CCCCTGATATAGCCTTTCCTCGAATAAATAATATAAGTTTTTGAATACTACCTCCTTCAT
TGACTCTTCTATTATCAAGCTCAATAGTAGAAAATGGGGCAGGAACTGGGTGAACAGTTT
ATCCTCCTCTCTCTTCAGGAACAGCTCATGCTGGAGCTTCTGTTGATTTAGCTATTTTTT
CTCTTCATTTAGCTGGAATTTCCTCAATTTTAGGGGCAGTAAATTTTATTACAACTGTAA
TTAATATACGATCGTCAGGAATTACTTTAGACCGACTACCTTTATTTGTTTGATCTGTAG
TTATTACAGCTATCTTATTACTTCTTTCTCTTCCTGTTTTAGCTGGAGCTATTACTATGT
TATTAACAGACCGAAACTTAAATACATCTTTCTTTGATCCAATCGGAGGAGGAGATCCTA
TT
>KM613059.1
TCGGAACTTCTCTAAGAATTTTAATTCGTGCTGAACTTAGCCACCCTGGTATATTTATTG
GGAATGACCAAATTTATAATGTAATTGTAACAGCTCATGCATTTATTATAATTTTCTTTA
TAGTAATACCAATTATAATTGGAGGATTTGGAAATTGATTAGTTCCTTTAATATTAGGAG
CCCCTGATATAGCCTTTCCTCGAATAAATAATATAAGTTTTTGAATACTACCTCCTTCAT
TGACTCTTCTATTATCAAGCTCAATAGTAGAAAATGGGGCAGGAACTGGGTGAACAGTTT
ATCCTCCTCTCTCTTCAGGAACAGCTCATGCTGGAGCTTCTGTTGATTTAGCTATTTTTT
CTCTTCATTTAGCTGGAATTTCCTCAATTTTAGGGGCAGTAAATTTTATTACAACTGTAA
TTAATATACGATCGTCAGGAATTACTTTAGACCGACTACCTTTATTTGTTTGATCTGTAG
TTATTACAGCTATCTTATTACTTCTTTCTCTTCCTGTTTTAGCTGGAGCTATTACTATGT
TATTAACAGACCGAAACTTAAATACATCTTTCTTTGATCCAATCGGAGGAGGAGATCCTA
TT
>KM613060.1
TCGGAACTTCTCTAAGAATTTTAATTCGTGCTGAACTTAGCCACCCTGGTATATTTATTG
GGAATGACCAAATTTATAATGTAATTGTAACAGCTCATGCATTTATTATAATTTTCTTTA
TAGTAATACCAATTATAATTGGAGGATTTGGAAATTGATTAGTTCCTTTAATATTAGGAG
CCCCTGATATAGCCTTTCCTCGAATAAATAATATAAGTTTTTGAATACTACCTCCTTCAT
TGACTCTTCTATTATCAAGCTCAATAGTAGAAAATGGGGCAGGAACTGGGTGAACAGTTT
ATCCTCCTCTCTCTTCAGGAACAGCTCATGCTGGAGCTTCTGTTGATTTAGCTATTTTTT
CTCTTCATTTAGCTGGAATTTCCTCAATTTTAGGGGCAGTAAATTTTATTACAACTGTAA
TTAATATACGATCGTCAGGAATTACTTTAGACCGACTACCTTTATTTGTTTGATCTGTAG
TTATTACAGCTATCTTATTACTTCTTTCTCTTCCTGTTTTAGCTGGAGCTATTACTATGT
TATTAACAGACCGAAACTTAAATACATCTTTCTTTGATCCAATCGGAGGAGGAGATCCTA
TT
>KM613061.1
TCGGAACTTCTCTAAGAATTTTAATTCGTGCTGAACTTAGCCACCCTGGTATATTTATTG
GGAATGACCAAATTTATAATGTAATTGTAACAGCTCATGCATTTATTATAATTTTCTTTA
TAGTAATACCAATTATAATTGGAGGATTTGGAAATTGATTAGTTCCTTTAATATTAGGAG
CCCCTGATATAGCCTTTCCTCGAATAAATAATATAAGTTTTTGAATACTACCTCCTTCAT
TGACTCTTCTATTATCAAGCTCAATAGTAGAAAATGGAGCAGGAACTGGGTGAACAGTGT
ATCCTCCTCTCTCTTCAGGAACAGCTCATGCTGGAGCTTCTGTTGATTTAGCTATTTTTT
CTCTTCATTTAGCTGGAATTTCCTCAATTTTAGGGGCAGTAAATTTTATTACAACTGTAA
TTAATATACGATCGTCAGGAATTACTTTAGACCGACTACCTTTATTTGTTTGATCTGTAG
TTATTACAGCTATCTTATTACTTCTTTCTCTTCCTGTTTTAGCTGGAGCTATTACTATGT

TATTAACAGACCGAAACTTAAATACATCTTTCTTCGATCCAATCGGAGGAGGAGATCCTA
TT
>KM613062.1
TCGGAACTTCTCTAAGAATTTTAATTCGTGCTGAACTTAGCCACCCTGGTATATTTATTG
GGAATGACCAAATTTATAATGTAATTGTAACAGCTCATGCATTTATTATAATTTTCTTTA
TAGTAATACCAATTATAATTGGAGGATTTGGAAATTGATTAGTTCCTTTAATATTAGGAG
CCCCTGATATAGCCTTTCCTCGAATAAATAATATAAGTTTTTGAATACTACCTCCTTCAT
TGACTCTTCTATTATCAAGCTCAATAGTAGAAAATGGGGCAGGAACTGGGTGAACAGTTT
ATCCTCCTCTCTCTTCAGGAACAGCTCATGCTGGAGCTTCTGTTGATTTAGCTATTTTTT
CTCTTCATTTAGCTGGAATTTCCTCAATTTTAGGGGCAGTAAATTTTATTACAACTGTAA
TTAATATACGATCGTCAGGAATTACTTTAGACCGACTACCTTTATTTGTTTGATCTGTAG
TTATTACAGCTATCTTATTACTTCTTTCTCTTCCTGTTTTAGCTGGAGCTATTACTATGT
TATTAACAGACCGAAACTTAAATACATCTTTCTTTGATCCAATCGGAGGAGGAGATCCTA
TT
>KM613063.1
TCGGAACTTCTCTAAGAATTTTAATTCGTGCTGAACTTAGCCACCCTGGTATATTTATTG
GGAATGACCAAATTTATAATGTAATTGTAACAGCTCATGCATTTATTATAATTTTCTTTA
TAGTAATACCAATTATAATTGGAGGATTTGGAAATTGATTAGTTCCTTTAATATTAGGAG
CCCCTGATATAGCCTTTCCTCGAATAAATAATATAAGTTTTTGAATACTACCTCCTTCAT
TGACTCTTCTATTATCAAGCTCAATAGTAGAAAATGGAGCAGGAACTGGGTGAACAGTGT
ATCCTCCTCTCTCTTCAGGAACAGCTCATGCTGGAGCTTCTGTTGATTTAGCTATTTTTT
CTCTTCATTTAGCTGGAATTTCCTCAATTTTAGGGGCAGTAAATTTTATTACAACTGTAA
TTAATATACGATCGTCAGGAATTACTTTAGACCGACTACCTTTATTTGTTTGATCTGTAG
TTATTACAGCTATCTTATTACTTCTTTCTCTTCCTGTTTTAGCTGGAGCTATTACTATGT
TATTAACAGACCGAAACTTAAATACATCTTTCTTCGATCCAATCGGAGGAGGAGATCCTA
TT
>KM613064.1
TCGGAACTTCTCTAAGAATTTTAATTCGTGCTGAACTTAGCCACCCTGGTATATTTATTG
GGAATGACCAAATTTATAATGTAATTGTAACAGCTCATGCATTTATTATAATTTTCTTTA
TAGTAATACCAATTATAATTGGAGGATTTGGAAATTGATTAGTTCCTTTAATATTAGGAG
CCCCTGATATAGCCTTTCCTCGAATAAATAATATAAGTTTTTGAATACTACCTCCTTCAT
TGACTCTTCTATTATCAAGCTCAATAGTAGAAAATGGAGCAGGAACTGGGTGAACAGTGT
ATCCTCCTCTCTCTTCAGGAACAGCTCATGCTGGAGCTTCTGTTGATTTAGCTATTTTTT
CTCTTCATTTAGCTGGAATTTCCTCAATTTTAGGGGCAGTAAATTTTATTACAACTGTAA
TTAATATACGATCGTCAGGAATTACTTTAGACCGACTACCTTTATTTGTTTGATCTGTAG
TTATTACAGCTATCTTATTACTTCTTTCTCTTCCTGTTTTAGCTGGAGCTATTACTATGT
TATTAACAGACCGAAACTTAAATACATCTTTCTTCGATCCAATCGGAGGAGGAGATCCTA
TT
>KM613065.1
TCGGAACTTCTCTAAGAATTTTAATTCGTGCTGAACTTAGCCACCCTGGTATATTTATTG
GGAATGACCAAATTTATAATGTAATTGTAACAGCTCATGCATTTATTATAATTTTCTTTA
TAGTAATACCAATTATAATTGGAGGATTTGGAAATTGATTAGTTCCTTTAATATTAGGAG
CCCCTGATATAGCCTTTCCTCGAATAAATAATATAAGTTTTTGAATACTACCTCCTTCAT
TGACTCTTCTATTATCAAGCTCAATAGTAGAAAATGGAGCAGGAACTGGGTGAACAGTGT
ATCCTCCTCTCTCTTCAGGAACAGCTCATGCTGGAGCTTCTGTTGATTTAGCTATTTTTT
CTCTTCATTTAGCTGGAATTTCCTCAATTTTAGGGGCAGTAAATTTTATTACAACTGTAA
TTAATATACGATCGTCAGGAATTACTTTAGACCGACTACCTTTATTTGTTTGATCTGTAG
TTATTACAGCTATCTTATTACTTCTTTCTCTTCCTGTTTTAGCTGGAGCTATTACTATGT
TATTAACAGACCGAAACTTAAATACATCTTTCTTCGATCCAATCGGAGGAGGAGATCCTA
TT
>KM613066.1
TCGGAACTTCTCTAAGAATTTTAATTCGTGCTGAACTTAGCCACCCTGGTATATTTATTG
GGAATGACCAAATTTATAATGTAATTGTAACAGCTCATGCATTTATTATAATTTTCTTTA
TAGTAATACCAATTATAATTGGAGGATTTGGAAATTGATTAGTTCCTTTAATATTAGGAG
CCCCTGATATAGCCTTTCCTCGAATAAATAATATAAGTTTTTGAATACTACCTCCTTCAT
TGACTCTTCTATTATCAAGCTCAATAGTAGAAAATGGAGCAGGAACTGGGTGAACAGTGT
ATCCTCCTCTCTCTTCAGGAACAGCTCATGCTGGAGCTTCTGTTGATTTAGCTATTTTTT

CTCTTCATTTAGCTGGAATTTCCTCAATTTTAGGGGCAGTAAATTTTATTACAACTGTAA
TTAATATACGATCGTCAGGAATTACTTTAGACCGACTACCTTTATTTGTTTGATCTGTAG
TTATTACAGCTATCTTATTACTTCTTTCTCTTCCTGTTTTAGCTGGAGCTATTACTATGT
TATTAACAGACCGAAACTTAAATACATCTTTCTTCGATCCAATCGGAGGAGGAGATCCTA
TT
>KM613067.1
TCGGAACTTCTCTAAGAATTTTAATTCGTGCTGAACTTAGCCACCCTGGTATATTTATTG
GGAATGACCAAATTTATAATGTAATTGTAACAGCTCATGCATTTATTATAATTTTCTTTA
TAGTAATACCAATTATAATTGGAGGATTTGGAAATTGATTAGTTCCTTTAATATTAGGAG
CCCCTGATATAGCCTTTCCTCGAATAAATAATATAAGTTTTTGAATACTACCTCCTTCAT
TGACTCTTCTATTATCAAGCTCAATAGTAGAAAATGGGGCAGGAACTGGGTGAACAGTTT
ATCCTCCTCTCTCTTCAGGAACAGCTCATGCTGGAGCTTCTGTTGATTTAGCTATTTTTT
CTCTTCATTTAGCTGGAATTTCCTCAATTTTAGGGGCAGTAAATTTTATTACAACTGTAA
TTAATATACGATCGTCAGGAATTACTTTAGATCGACTACCTTTATTTGTTTGATCTGTAG
TTATTACAGCTATCTTATTACTTCTTTCTCTTCCTGTTTTAGCTGGAGCTATTACTATGT
TATTAACAGACCGAAACTTAAATACATCTTTCTTTGATCCAATCGGAGGAGGAGATCCTA
TT
>KM613068.1
TCGGAACTTCTCTAAGAATTTTAATTCGTGCTGAACTTAGCCACCCTGGTATATTTATTG
GGAATGACCAAATTTATAATGTAATTGTAACAGCTCATGCATTTATTATAATTTTCTTTA
TAGTAATACCAATTATAATTGGAGGATTTGGAAATTGATTAGTTCCTTTAATATTAGGAG
CCCCTGATATAGCCTTTCCTCGAATAAATAATATAAGTTTTTGAATACTACCTCCTTCAT
TGACTCTTCTATTATCAAGCTCAATAGTAGAAAATGGGGCAGGAACTGGGTGAACAGTTT
ATCCTCCTCTCTCTTCAGGAACAGCTCATGCTGGAGCTTCTGTTGATTTAGCTATTTTTT
CTCTTCATTTAGCTGGAATTTCCTCAATTTTAGGGGCAGTAAATTTTATTACAACTGTAA
TTAATATACGATCGTCAGGAATTACTTTAGACCGACTACCTTTATTTGTTTGATCTGTAG
TTATTACAGCTATCTTATTACTTCTTTCTCTTCCTGTTTTAGCTGGAGCTATTACTATGT
TATTAACAGACCGAAACTTAAATACATCTTTCTTTGATCCAATCGGAGGAGGAGATCCTA
TT
>KM613069.1
TCGGAACTTCTCTAAGAATTTTAATTCGTGCTGAACTTAGCCACCCTGGTATATTTATTG
GGAATGACCAAATTTATAATGTAATTGTAACAGCTCATGCATTTATTATAATTTTCTTTA
TAGTAATACCAATTATAATTGGAGGATTTGGAAATTGATTAGTTCCTTTAATATTAGGAG
CCCCTGATATAGCCTTTCCTCGAATAAATAATATAAGTTTTTGAATACTACCTCCTTCAT
TGACTCTTCTATTATCAAGCTCAATAGTAGAAAATGGGGCAGGAACTGGGTGAACAGTTT
ATCCTCCTCTCTCTTCAGGAACAGCTCATGCTGGAGCTTCTGTTGATTTAGCTATTTTTT
CTCTTCATTTAGCTGGAATTTCCTCAATTTTAGGGGCAGTAAATTTTATTACAACTGTAA
TTAATATACGATCGTCAGGAATTACTTTAGACCGACTACCTTTATTTGTTTGATCTGTAG
TTATTACAGCTATCTTATTACTTCTTTCTCTTCCTGTTTTAGCTGGAGCTATTACTATGT
TATTAACAGACCGAAACTTAAATACATCTTTCTTTGATCCAATCGGAGGAGGAGATCCTA
TT
>KM613070.1
TCGGAACTTCTCTAAGAATTTTAATTCGTGCTGAACTTAGCCACCCTGGTATATTTATTG
GGAATGACCAAATTTATAATGTAATTGTAACAGCTCATGCATTTATTATAATTTTCTTTA
TAGTAATACCAATTATAATTGGAGGATTTGGAAATTGATTAGTTCCTTTAATATTAGGAG
CCCCTGATATAGCCTTTCCTCGAATAAATAATATAAGTTTTTGAATACTACCTCCTTCAT
TGACTCTTCTATTATCAAGCTCAATAGTAGAAAATGGGGCAGGAACTGGGTGAACAGTTT
ATCCTCCTCTCTCTTCAGGAACAGCTCATGCTGGAGCTTCTGTTGATTTAGCTATTTTTT
CTCTTCATTTAGCTGGAATTTCCTCAATTTTAGGGGCAGTAAATTTTATTACAACTGTAA
TTAATATACGATCGTCAGGAATTACTTTAGACCGACTACCTTTATTTGTTTGATCTGTAG
TTATTACAGCTATCTTATTACTTCTTTCTCTTCCTGTTTTAGCTGGAGCTATTACTATGT
TATTAACAGACCGAAACTTAAATACATCTTTCTTTGATCCAATCGGAGGAGGAGATCCTA
TT
>KM613071.1
TCGGAACTTCTCTAAGAATTTTAATTCGTGCTGAACTTAGCCACCCTGGTATATTTATTG
GGAATGACCAAATTTATAATGTAATTGTAACAGCTCATGCATTTATTATAATTTTCTTTA
TAGTAATACCAATTATAATTGGAGGATTTGGAAATTGATTAGTTCCTTTAATATTAGGAG

CCCCTGATATAGCCTTTCCTCGAATAAATAATATAAGTTTTTGAATACTACCTCCTTCAT
TGACTCTTCTATTATCAAGCTCAATAGTAGAAAATGGGGCAGGAACTGGGTGAACAGTTT
ATCCTCCTCTCTCTTCAGGAACAGCTCATGCTGGAGCTTCTGTTGATTTAGCTATTTTTT
CTCTTCATTTAGCTGGAATTTCCTCAATTTTAGGGGCAGTAAATTTTATTACAACTGTAA
TTAATATACGATCGTCAGGAATTACTTTAGACCGACTACCTTTATTTGTTTGATCTGTAG
TTATTACAGCTATCTTATTACTTCTTTCTCTTCCTGTTTTAGCTGGAGCTATTACTATGT
TATTAACAGACCGAAACTTAAATACATCTTTCTTTGATCCAATCGGAGGAGGAGATCCTA
TT
>KM613072.1
TCGGAACTTCTCTAAGAATTTTAATTCGTGCTGAACTTAGCCACCCTGGTATATTTATTG
GGAATGACCAAATTTATAATGTAATTGTAACAGCTCATGCATTTATTATAATTTTCTTTA
TAGTAATACCAATTATAATTGGAGGATTTGGAAATTGATTAGTTCCTTTAATATTAGGAG
CCCCTGATATAGCCTTTCCTCGAATAAATAATATAAGTTTTTGAATACTACCTCCTTCAT
TGACTCTTCTATTATCAAGCTCAATAGTAGAAAATGGGGCAGGAACTGGGTGAACAGTTT
ATCCTCCTCTCTCTTCAGGAACAGCTCATGCTGGAGCTTCTGTTGATTTAGCTATTTTTT
CTCTTCATTTAGCTGGAATTTCCTCAATTTTAGGGGCAGTAAATTTTATTACAACTGTAA
TTAATATACGATCGTCAGGAATTACTTTAGATCGACTACCTTTATTTGTTTGATCTGTAG
TTATTACAGCTATCTTATTACTTCTTTCTCTTCCTGTTTTAGCTGGAGCTATTACTATGT
TATTAACAGACCGAAACTTAAATACATCTTTCTTTGATCCAATCGGAGGAGGAGATCCTA
TT
>KM613073.1
TCGGAACTTCTCTAAGAATTTTAATTCGTGCTGAACTTAGCCACCCTGGTATATTTATTG
GGAATGACCAAATTTATAATGTAATTGTAACAGCTCATGCATTTATTATAATTTTCTTTA
TAGTAATACCAATTATAATTGGAGGATTTGGAAATTGATTAGTTCCTTTAATATTAGGAG
CCCCTGATATAGCCTTTCCTCGAATAAATAATATAAGTTTTTGAATACTACCTCCTTCAT
TGACTCTTCTATTATCAAGCTCAATAGTAGAAAATGGGGCAGGAACTGGGTGAACAGTTT
ATCCTCCTCTCTCTTCAGGAACAGCTCATGCTGGAGCTTCTGTTGATTTAGCTATTTTTT
CTCTTCATTTAGCTGGAATTTCCTCAATTTTAGGGGCAGTAAATTTTATTACAACTGTAA
TTAATATACGATCGTCAGGAATTACTTTAGATCGACTACCTTTATTTGTTTGATCTGTAG
TTATTACAGCTATCTTATTACTTCTTTCTCTTCCTGTTTTAGCTGGAGCTATTACTATGT
TATTAACAGACCGAAACTTAAATACATCTTTCTTTGATCCAATCGGAGGAGGAGATCCTA
TT
>KM613074.1
TCGGAACTTCTCTAAGAATTTTAATTCGTGCTGAACTTAGCCACCCTGGTATATTTATTG
GGAATGACCAAATTTATAATGTAATTGTAACAGCTCATGCATTTATTATAATTTTCTTTA
TAGTAATGCCAATTATAATTGGAGGATTTGGAAATTGATTAGTTCCTTTAATATTAGGAG
CCCCTGATATAGCTTTCCCTCGAATGAATAATATAAGTTTTTGAATACTACCTCCTTCAT
TGACTCTTCTATTATCAAGCTCAATAGTAGAAAATGGGGCAGGAACTGGGTGAACAGTTT
ATCCTCCTCTCTCTTCAGGAACAGCTCATGCTGGAGCTTCTGTTGATTTAGCTATTTTTT
CTCTTCATTTAGCTGGAATTTCCTCAATTTTAGGGGCAGTAAATTTTATTACAACTGTGA
TTAATATGCGATCGTCAGGGATTACTTTAGATCGACTACCCTTATTTGTTTGATCTGTAG
TTATTACAGCTATCTTATTACTTCTTTCTCTTCCTGTTTTAGCTGGAGCTATTACTATAT
TATTAACAGACCGAAACTTAAATACATCTTTCTTTGATCCAATCGGAGGGGGAGACCCTA
TT
>KM613075.1
TCGGAACTTCTCTAAGAATTTTAATTCGTGCTGAACTTAGCCACCCTGGTATATTTATTG
GGAATGACCAAATTTATAATGTAATTGTAACAGCTCATGCATTTATTATAATTTTCTTTA
TAGTAATACCAATTATAATTGGAGGATTTGGAAATTGATTAGTTCCTTTAATATTAGGAG
CCCCTGATATAGCCTTTCCTCGAATAAATAATATAAGTTTTTGAATACTACCTCCTTCAT
TGACTCTTCTATTATCAAGCTCAATAGTAGAAAATGGGGCAGGAACTGGGTGAACAGTTT
ATCCTCCTCTCTCTTCAGGAACAGCTCATGCTGGAGCTTCTGTTGATTTAGCTATTTTTT
CTCTTCATTTAGCTGGAATTTCCTCAATTTTAGGGGCAGTAAATTTTATTACAACTGTAA
TTAATATACGATCGTCAGGAATTACTTTAGACCGACTACCTTTATTTGTTTGATCTGTAG
TTATTACAGCTATCTTATTACTTCTTTCTCTTCCTGTTTTAGCTGGAGCTATTACTATGT
TATTAACAGACCGAAACTTAAATACATCTTTCTTTGATCCAATCGGAGGAGGAGATCCTA
TT
>KM613076.1

TCGGAACTTCTCTAAGAATTTTAATTCGTGCTGAACTTAGCCACCCTGGTATATTTATTG
GGAATGACCAAATTTATAATGTAATTGTAACAGCTCATGCATTTATTATAATTTTCTTTA
TAGTAATACCAATTATAATTGGAGGATTTGGAAATTGATTAGTTCCTTTAATATTAGGAG
CCCCTGATATAGCCTTTCCTCGAATAAATAATATAAGTTTTTGAATACTACCTCCTTCAT
TGACTCTTCTATTATCAAGCTCAATAGTAGAAAATGGGGCAGGAACTGGGTGAACAGTTT
ATCCTCCTCTCTCTTCAGGAACAGCTCATGCTGGAGCTTCTGTTGATTTAGCTATTTTTT
CTCTTCATTTAGCTGGAATTTCCTCAATTTTAGGGGCAGTAAATTTTATTACAACTGTAA
TTAATATACGATCGTCAGGAATTACTTTAGATCGACTACCTTTATTTGTTTGATCTGTAG
TTATTACAGCTATCTTATTACTTCTTTCTCTTCCTGTTTTAGCTGGAGCTATTACTATGT
TATTAACAGACCGAAACTTAAATACATCTTTCTTTGATCCAATCGGAGGAGGAGATCCTA
TT
>KM613077.1
TCGGAACTTCTCTAAGAATTTTAATTCGTGCTGAACTTAGCCACCCTGGTATATTTATTG
GGAATGACCAAATTTATAATGTAATTGTAACAGCTCATGCATTTATTATAATTTTCTTTA
TAGTAATGCCAATTATAATTGGAGGATTTGGAAATTGATTAGTTCCTTTAATATTAGGAG
CCCCTGATATAGCTTTCCCTCGAATGAATAATATAAGTTTTTGAATACTACCTCCTTCAT
TGACTCTTCTATTATCAAGCTCAATAGTAGAAAATGGGGCAGGAACTGGGTGAACAGTTT
ATCCTCCTCTCTCTTCAGGAACAGCTCATGCTGGAGCTTCTGTTGATTTAGCTATTTTTT
CTCTTCATTTAGCTGGAATTTCCTCAATTTTAGGGGCAGTAAATTTTATTACAACTGTGA
TTAATATGCGATCGTCAGGGATTACTTTAGATCGACTACCCTTATTTGTTTGATCTGTAG
TTATTACAGCTATCTTATTACTTCTTTCTCTTCCTGTTTTAGCTGGAGCTATTACTATAT
TATTAACAGACCGAAACTTAAATACATCTTTCTTTGATCCAATCGGAGGGGGAGACCCTA
TT
>KM613078.1
TCGGAACTTCTCTAAGAATTTTAATTCGTGCTGAACTTAGCCACCCTGGTATATTTATTG
GGAATGACCAAATTTATAATGTAATTGTAACAGCTCATGCATTTATTATAATTTTCTTTA
TAGTAATACCAATTATAATTGGAGGATTTGGAAATTGATTAGTTCCTTTAATATTAGGAG
CCCCTGATATAGCCTTTCCTCGAATAAATAATATAAGTTTTTGAATACTACCTCCTTCAT
TGACTCTTCTATTATCAAGCTCAATAGTAGAAAATGGGGCAGGAACTGGGTGAACAGTTT
ATCCTCCTCTCTCTTCAGGAACAGCTCATGCTGGAGCTTCTGTTGATTTAGCTATTTTTT
CTCTTCATTTAGCTGGAATTTCCTCAATTTTAGGGGCAGTAAATTTTATTACAACTGTAA
TTAATATACGATCGTCAGGAATTACTTTAGACCGACTACCTTTATTTGTTTGATCTGTAG
TTATTACAGCTATCTTATTACTTCTTTCTCTTCCTGTTTTAGCTGGAGCTATTACTATGT
TATTAACAGACCGAAACTTAAATACATCTTTCTTTGATCCAATCGGAGGAGGAGATCCTA
TT
>KM613079.1
TCGGAACTTCTCTAAGAATTTTAATTCGTGCTGAACTTAGCCACCCTGGTATATTTATTG
GGAATGACCAAATTTATAATGTAATTGTAACAGCTCATGCATTTATTATAATTTTCTTTA
TAGTAATACCAATTATAATTGGAGGATTTGGAAATTGATTAGTTCCTTTAATATTAGGAG
CCCCTGATATAGCCTTTCCTCGAATAAATAATATAAGTTTTTGAATACTACCTCCTTCAT
TGACTCTTCTATTATCAAGCTCAATAGTAGAAAATGGGGCAGGAACTGGGTGAACAGTTT
ATCCTCCTCTCTCTTCAGGAACAGCTCATGCTGGAGCTTCTGTTGATTTAGCTATTTTTT
CTCTTCATTTAGCTGGAATTTCCTCAATTTTAGGGGCAGTAAATTTTATTACAACTGTAA
TTAATATACGATCGTCAGGAATTACTTTAGACCGACTACCTTTATTTGTTTGATCTGTAG
TTATTACAGCTATCTTATTACTTCTTTCTCTTCCTGTTTTAGCTGGAGCTATTACTATGT
TATTAACAGACCGAAACTTAAATACATCTTTCTTTGATCCAATCGGAGGAGGAGATCCTA
TT
>KM613080.1
TCGGAACTTCTCTAAGAATTTTAATTCGTGCTGAACTTAGCCACCCTGGTATATTTATTG
GGAATGACCAAATTTATAATGTAATTGTAACAGCTCATGCATTTATTATAATTTTCTTTA
TAGTAATACCAATTATAATTGGAGGATTTGGAAATTGATTAGTTCCTTTAATATTAGGAG
CCCCTGATATAGCCTTTCCTCGAATAAATAATATAAGTTTTTGAATACTACCTCCTTCAT
TGACTCTTCTATTATCAAGCTCAATAGTAGAAAATGGGGCAGGAACTGGGTGAACAGTTT
ATCCTCCTCTCTCTTCAGGAACAGCTCATGCTGGAGCTTCTGTTGATTTAGCTATTTTTT
CTCTTCATTTAGCTGGAATTTCCTCAATTTTAGGGGCAGTAAATTTTATTACAACTGTAA
TTAATATACGATCGTCAGGAATTACTTTAGACCGACTACCTTTATTTGTTTGATCTGTAG
TTATTACAGCTATCTTATTACTTCTTTCTCTTCCTGTTTTAGCTGGAGCTATTACTATGT

TATTAACAGACCGAAACTTAAATACATCTTTCTTTGATCCAATCGGAGGAGGAGATCCTA
TT
>KM613081.1
TCGGAACTTCTCTAAGAATTTTAATTCGTGCTGAACTTAGCCACCCTGGTATATTTATTG
GGAATGACCAAATTTATAATGTAATTGTAACAGCTCATGCATTTATTATAATTTTCTTTA
TAGTAATACCAATTATAATTGGAGGATTTGGAAATTGATTAGTTCCTTTAATATTAGGAG
CCCCTGATATAGCCTTTCCTCGAATAAATAATATAAGTTTTTGAATACTACCTCCTTCAT
TGACTCTTCTATTATCAAGCTCAATAGTAGAAAATGGGGCAGGAACTGGGTGAACAGTTT
ATCCTCCTCTCTCTTCAGGAACAGCTCATGCTGGAGCTTCTGTTGATTTAGCTATTTTTT
CTCTTCATTTAGCTGGAATTTCCTCAATTTTAGGGGCAGTAAATTTTATTACAACTGTAA
TTAATATACGATCGTCAGGAATTACTTTAGACCGACTACCTTTATTTGTTTGATCTGTAG
TTATTACAGCTATCTTATTACTTCTTTCTCTTCCTGTTTTAGCTGGAGCTATTACTATGT
TATTAACAGACCGAAACTTAAATACATCTTTCTTTGATCCAATCGGAGGAGGAGATCCTA
TT
>KM613082.1
TCGGAACTTCTCTAAGAATTTTAATTCGTGCTGAACTTAGCCACCCTGGTATATTTATTG
GGAATGACCAAATTTATAATGTAATTGTAACAGCTCATGCATTTATTATAATTTTCTTTA
TAGTAATACCAATTATAATTGGAGGATTTGGAAATTGATTAGTTCCTTTAATATTAGGAG
CCCCTGATATAGCCTTTCCTCGAATAAATAATATAAGTTTTTGAATACTACCTCCTTCAT
TGACTCTTCTATTATCAAGCTCAATAGTAGAAAATGGGGCAGGAACTGGGTGAACAGTTT
ATCCTCCTCTCTCTTCAGGAACAGCTCATGCTGGAGCTTCTGTTGATTTAGCTATTTTTT
CTCTTCATTTAGCTGGAATTTCCTCAATTTTAGGGGCAGTAAATTTTATTACAACTGTAA
TTAATATACGATCGTCAGGAATTACTTTAGACCGACTACCTTTATTTGTTTGATCTGTAG
TTATTACAGCTATCTTATTACTTCTTTCTCTTCCTGTTTTAGCTGGAGCTATTACTATGT
TATTAACAGACCGAAACTTAAATACATCTTTCTTTGATCCAATCGGAGGAGGAGATCCTA
TT
>MF371160.1
-----------------------------------CTTAGCCACCCTGGTATATTTATTG
GGAATGACCAAATTTATAATGTAATTGTAACAGCTCATGCATTTATTATAATTTTCTTTA
TAGTAATACCAATTATAATTGGAGGATTTGGAAATTGATTAGTTCCTTTAATATTAGGAG
CCCCTGATATAGCCTTTCCTCGAATAAATAATATAAGTTTTTGAATACTACCTCCTTCAT
TGACTCTTCTATTATCAAGCTCAATAGTAGAAAATGGGGCAGGAACTGGGTGAACAGTTT
ATCCTCCTCTCTCTTCAGGAACAGCTCATGCTGGAGCTTCTGTTGATTTAGCTATTTTTT
CTCTTCATTTAGCTGGAATTTCCTCAATTTTAGGGGCAGTAAATTTTATTACAACTGTAA
TTAATATACGATCGTCAGGAATTACTTTAGATCGACTACCTTTATTTGTTTGATCTGTAG
TTATTACAGCTATCTTATTACTTCTTTCTCTTCCTGTTTTAGCTGGAGCTATTACTATGT
TATTA---
--
>MF371161.1
TCGGAACTTCTCTAAGAATTTTAATTCGTGCTGAACTTAGCCACCCTGGTATATTTATTG
GGAATGACCAAATTTATAATGTAATTGTAACAGCTCATGCATTTATTATAATTTTCTTTA
TAGTAATACCAATTATAATTGGAGGATTTGGAAATTGATTAGTTCCTTTAATATTAGGAG
CCCCTGATATAGCCTTTCCTCGAATAAATAATATAAGTTTTTGAATACTACCTCCTTCAT
TGACTCTTCTATTATCAAGCTCAATAGTAGAAAATGGGGCAGGAACTGGGTGAACAGTTT
ATCCTCCTCTCTCTTCAGGAACAGCTCATGCTGGAGCTTCTGTTGATTTAGCTATTTTTT
CTCTTCATTTAGCTGGAATTTCCTCAATTTTAGGGGCAGTAAATTTTATTACAACTGTAA
TTAATATACGATCGTCAGGAATTACTTTAGATCGACTACCTTTATTTGTTTGATCTGTAG
TTATTACAGCTATCTTATTACTTCTTTCTCTTCCTGTTTTAGCTGGAGCTATTACTATGT
TATTAACAGACCGAAACTTAAATACATCTTTCTTTGATCCAATCGGAGGAGGAGATCCTA
TT
>MF371162.1
--------------------------------------AGCCACCCTGGTATATTTATTG
GGAATGACCAAATTTATAATGTAATTGTAACAGCTCATGCATTTATTATAATTTTCTTTA
TAGTAATACCAATTATAATTGGAGGATTTGGAAATTGATTAGTTCCTTTAATATTAGGAG
CCCCTGATATAGCCTTTCCTCGAATAAATAATATAAGTTTTTGAATACTACCTCCTTCAT
TGACTCTTCTATTATCAAGCTCAATAGTAGAAAATGGGGCAGGAACTGGGTGAACAGTTT
ATCCTCCTCTCTCTTCAGGAACAGCTCATGCTGGAGCTTCTGTTGATTTAGCTATTTTTT

CTCTTCATTTAGCTGGAATTTCCTCAATTTTAGGGGCAGTAAATTTTATTACAACTGTAA
TTAATATACGATCGTCAGGAATTACTTTAGATCGACTACCTTTATTTGTTTGATCTGTAG
TTATTACAGCTATCTTATTACTTCTTTCTCTTCCTGTTTTAGCTGGAGCTATTACTATGT
TATTA---
--
>MF371163.1
-----------------------------------CTTAGCCACCCTGGTATATTTATTG
GGAATGACCAAATTTATAATGTAATTGTAACAGCTCATGCATTTATTATAATTTTCTTTA
TAGTAATACCAATTATAATTGGAGGATTTGGAAATTGATTAGTTCCTTTAATATTAGGAG
CCCCTGATATAGCCTTTCCTCGAATAAATAATATAAGTTTTTGAATACTACCTCCTTCAT
TGACTCTTCTATTATCAAGCTCAATAGTAGAAAATGGGGCAGGAACTGGGTGAACAGTTT
ATCCTCCTCTCTCTTCAGGAACAGCTCATGCTGGAGCTTCTGTTGATTTAGCTATTTTTT
CTCTTCATTTAGCTGGAATTTCCTCAATTTTAGGGGCAGTAAATTTTATTACAACTGTAA
TTAATATACGATCGTCAGGAATTACTTTAGATCGACTACCTTTATTTGTTTGATCTGTAG
TTATTACAGCTATCTTATTACTTCTTTCTCTTCCTGTTTTAGCTGGAGCTATTACTATGT
TATTA---
--
>MF371164.1
-----------------------------GCTGAACTTAGCCACCCTGGTATATTTATTG
GGAATGACCAAATTTATAATGTAATTGTAACAGCTCATGCATTTATTATAATTTTCTTTA
TAGTAATGCCAATTATAATTGGAGGATTTGGAAATTGATTAGTTCCTTTAATATTAGGAG
CCCCTGATATAGCTTTCCCTCGAATGAATAATATAAGTTTTTGAATACTACCTCCTTCAT
TGACTCTTCTATTATCAAGCTCAATAGTAGAAAATGGAGCAGGAACTGGATGAACAGTTT
ATCCTCCTCTTTCTTCAGGAACAGCTCATGCTGGAGCTTCTGTTGATTTAGCTATTTTTT
CTCTTCATTTAGCTGGAATTTCCTCAATTTTAGGGGCAGTAAATTTTATTACAACTGTAA
TTAATATACGATCGTCAGGGATTACTTTAGATCGACTACCCTTATTTGTTTGATCTGTAG
TTATTACAGCTATCTTATTACTTCTTTCTCTTCCTGTTTTAGCTGGAGCTATTACTATAT
TATTA---
--
>MF371165.1
--------------------------------------AGCCACCCTGGTATATTTATTG
GGAATGACCAAATTTATAATGTAATTGTAACAGCTCATGCATTTATTATAATTTTCTTTA
TAGTAATGCCAATTATAATTGGAGGATTTGGAAATTGATTAGTTCCTTTAATATTAGGAG
CCCCTGATATAGCTTTCCCTCGAATGAATAATATAAGTTTTTGAATACTACCTCCTTCAT
TGACTCTTCTATTATCAAGCTCAATAGTAGAAAATGGAGCAGGAACTGGATGAACAGTTT
ATCCTCCTCTTTCTTCAGGAACAGCTCATGCTGGAGCTTCTGTTGATTTAGCTATTTTTT
CTCTTCATTTAGCTGGAATTTCCTCAATTTTAGGGGCAGTAAATTTTATTACAACTGTAA
TTAATATACGATCGTCAGGGATTACTTTAGATCGACTACCCTTATTTGTTTGATCTGTAG
TTATTACAGCTATCTTATTACTTCTTTCTCTTCCTGTTTTAGCTGGAGCTATTACTATAT
TATTAACA--
--
>MF371166.1
-----------------------------------CTTAGCCACCCTGGTATATTTATTG
GGAATGACCAAATTTATAATGTAATTGTAACAGCTCATGCATTTATTATAATTTTCTTTA
TAGTAATGCCAATTATAATTGGAGGATTTGGAAATTGATTAGTTCCTTTAATATTAGGAG
CCCCTGATATAGCTTTCCCTCGAATGAATAATATAAGTTTTTGAATACTACCTCCTTCAT
TGACTCTTCTATTATCAAGCTCAATAGTAGAAAATGGAGCAGGAACTGGATGAACAGTTT
ATCCTCCTCTTTCTTCAGGAACAGCTCATGCTGGAGCTTCTGTTGATTTAGCTATTTTTT
CTCTTCATTTAGCTGGAATTTCCTCAATTTTAGGGGCAGTAAATTTTATTACAACTGTAA
TTAATATACGATCGTCAGGGATTACTTTAGATCGACTACCCTTATTTGTTTGATCTGTAG
TTATTACAGCTATCTTATTACTTCTTTCTCTTCCTGTTTTAGCTGGAGCTATTACTATAT
TATTA---
--
>MF371167.1
--------------------------------------AACCACCCTGGTATATTTATTG
GGAATGACCAAATTTATAATGTAATTGTAACAGCTCATGCATTTATTATAATTTTCTTTA
TAGTAATACCAATTATAATTGGAGGATTTGGAAATTGATTAGTTCCTTTAATATTAGGAG

CCCCTGATATAGCTTTCCCTCGAATGAATAATATAAGTTTTTGAATACTACCTCCTTCAT
TGACTCTTCTATTATCAAGCTCAATAGTAGAAAATGGAGCAGGAACTGGGTGAACAGTTT
ATCCTCCTCTCTCTTCAGGAACAGCTCATGCTGGAGCTTCTGTTGATTTAGCTATTTTTT
CTCTTCATTTAGCTGGAATTTCCTCAATTTTAGGAGCAGTAAATTTTATTACAACTGTAA
TTAATATACGATCGTCAGGAATTACTTTAGATCGACTACCCTTATTTGTTTGATCTGTAG
TTATTACAGCTATCTTATTACTTCTTTCTCTTCCTGTTTTAGCTGGAGCTATTACTATAT
TATTA---
--
>MF371168.1
ACGGAACTTCTCTAAGAATTTTAATTCGTGCTGAACTTAGCCACCCTGGTATATTTATTG
GGAATGACCAAATTTATAATGTAATTGTAACAGCTCATGCATTTATTATAATTTTCTTTA
TAGTAATACCAATTATAATTGGAGGATTTGGAAATTGATTAGTTCCTTTAATATTAGGAG
CCCCTGATATAGCTTTCCCTCGAATGAATAATATAAGTTTTTGAATACTACCTCCTTCAT
TGACTCTTCTATTATCAAGCTCAATAGTAGAAAATGGAGCAGGAACTGGGTGAACAGTTT
ATCCTCCTCTCTCTTCAGGAACAGCTCATGCTGGAGCTTCTGTTGATTTAGCTATTTTTT
CTCTTCATTTAGCTGGAATTTCCTCAATTTTAGGAGCAGTAAATTTTATTACAACTGTAA
TTAATATACGATCGTCAGGAATTACTTTAGATCGACTACCCTTATTTGTTTGATCTGTAG
TTATTACAGCTATCTTATTACTTCTTTCTCTTCCTGTTTTAGCTGGAGCTATTACTATAT
TATTAACAGACCGAAACTTAAATACATCTTTCTTTGACCCAATCGGA-------------
--
>MF371169.1
---------------------------------------AACCACCCTGGTATATTTATTG
GGAATGACCAAATTTATAATGTAATTGTAACAGCTCATGCATTTATTATAATTTTCTTTA
TAGTAATACCAATTATAATTGGAGGATTTGGAAATTGATTAGTTCCTTTAATATTAGGAG
CCCCTGATATAGCTTTCCCTCGAATGAATAATATAAGTTTTTGAATACTACCTCCTTCAT
TGACTCTTCTATTATCAAGCTCAATAGTAGAAAATGGAGCAGGAACTGGGTGAACAGTTT
ATCCTCCTCTCTCTTCAGGAACAGCTCATGCTGGAGCTTCTGTTGATTTAGCTATTTTTT
CTCTTCATTTAGCTGGAATTTCCTCAATTTTAGGAGCAGTAAATTTTATTACAACTGTAA
TTAATATACGATCGTCAGGAATTACTTTAGATCGACTACCCTTATTTGTTTGATCTGTAG
TTATTACAGCTATCTTATTACTTCTTTCTCTTCCTGTTTTAGCTGGAGCTATTACTATAT
TATTA---
--
>MF371170.1
------------------------------------CTTAGCCACCCTGGTATATTTATTG
GGAATGACCAAATTTATAATGTAATTGTAACAGCTCATGCATTTATTATAATTTTCTTTA
TAGTAATACCAATTATAATTGGAGGATTTGGAAATTGATTAGTTCCTTTAATATTAGGAG
CCCCTGATATAGCCTTTCCTCGAATAAATAATATAAGTTTTTGAATACTACCTCCTTCAT
TGACTCTTCTATTATCAAGCTCAATAGTAGAAAATGGAGCAGGAACTGGGTGAACAGTTT
ATCCTCCTCTCTCTTCAGGAACAGCTCATGCTGGAGCTTCTGTTGATTTAGCTATTTTTT
CTCTTCATTTAGCTGGAATTTCCTCAATTTTAGGGGCAGTAAATTTTATTACAACTGTAA
TTAATATACGATCGTCAGGAATTACTTTAGATCGACTACCCTTATTTGTTTGATCTGTAG
TTATTACAGCTATCTTATTACTTCTTTCTCTTCCTGTTTTAGCTGGAGCTATTACTATAT
TATTA---
--
>MF371171.1
------------------------------------CTTAGCCACCCTGGTATATTTATTG
GGAATGACCAAATTTATAATGTAATTGTAACAGCTCATGCATTTATTATAATTTTCTTTA
TAGTAATACCAATTATAATTGGAGGATTTGGAAATTGATTAGTTCCTTTAATATTAGGAG
CCCCTGATATAGCCTTTCCTCGAATAAATAATATAAGTTTTTGAATACTACCTCCTTCAT
TGACTCTTCTATTATCAAGCTCAATAGTAGAAAATGGAGCAGGAACTGGGTGAACAGTTT
ATCCTCCTCTCTCTTCAGGAACAGCTCATGCTGGAGCTTCTGTTGATTTAGCTATTTTTT
CTCTTCATTTAGCTGGAATTTCCTCAATTTTAGGGGCAGTAAATTTTATTACAACTGTAA
TTAATATACGATCGTCAGGAATTACTTTAGATCGACTACCCTTATTTGTTTGATCTGTAG
TTATTACAGCTATCTTATTACTTCTTTCTCTTCCTGTTTTAGCTGGAGCTATTACTATGT
TATTA---
--
>MF371172.1

TCGGAACTTCTCTAAGAATTTTAATTCGTGCTGAACTTAGCCACCCTGGTATATTTATTG
GGAATGACCAAATTTATAATGTAATTGTAACAGCTCATGCATTTATTATAATTTTCTTTA
TAGTAATACCAATTATAATTGGAGGATTTGGAAATTGATTAGTTCCTTTAATATTAGGAG
CCCCTGATATAGCCTTTCCTCGAATAAATAATATAAGTTTTTGAATACTACCTCCTTCAT
TGACTCTTCTATTATCAAGCTCAATAGTAGAAAATGGAGCAGGAACTGGGTGAACAGTTT
ATCCTCCTCTCTCTTCAGGAACAGCTCATGCTGGAGCTTCTGTTGATTTAGCTATTTTTT
CTCTTCATTTAGCTGGAATTTCCTCAATTTTAGGGGCAGTAAATTTTATTACAACTGTAA
TTAATATACGATCGTCAGGAATTACTTTAGATCGACTACCCTTATTTGTTTGATCTGTAG
TTATTACAGCTATCTTATTACTTCTTTCTCTTCCTGTTTTAGCTGGAGCTATTACTATGT
TATTAACAGACCGAAACTTAAATACATCTTTC----------------------------
--
>MF371173.1
--------------------------------------AGCCACCCTGGTATATTTATTG
GGAATGACCAAATTTATAACGTAATTGTAACAGCTCATGCATTTATTATAATTTTCTTTA
TAGTAATGCCAATTATAATTGGAGGATTTGGAAATTGATTAGTTCCTTTAATATTAGGAG
CCCCTGATATAGCTTTCCCTCGAATGAATAATATAAGTTTTTGAATACTACCTCCTTCAT
TGACTCTTCTATTATCAAGCTCAATAGTAGAAAATGGAGCAGGAACTGGATGAACAGTTT
ATCCTCCTCTTTCTTCAGGAACAGCTCATGCTGGAGCTTCTGTTGATTTAGCTATTTTTT
CTCTTCATTTAGCTGGAATTTCCTCAATTTTAGGGGCAGTAAATTTTATTACAACTGTAA
TTAATATACGATCGTCAGGGATTACTTTAGATCGACTACCCTTATTTGTTTGATCTGTAG
TTATTACAGCTATCTTATTACTTCTTTCTCTTCCTGTTTTAGCTGGAGCTATTACTATAT
TATTA---
--
>MF371174.1
TCGGAACTTCTCTAAGAATTTTAATTCGTGCTGAACTTAGCCACCCTGGTATATTTATTG
GGAATGACCAAATTTATAATGTAATTGTAACAGCTCATGCATTTATTATAATTTTCTTTA
TAGTAATGCCAATTATAATTGGAGGATTTGGAAATTGATTAGTTCCTTTAATATTAGGAG
CCCCTGATATAGCTTTCCCTCGAATGAATAATATAAGTTTTTGAATACTACCTCCTTCAT
TGACTCTTCTATTATCAAGCTCAATAGTAGAAAATGGGGCAGGAACTGGGTGAACAGTTT
ATCCTCCTCTCTCTTCAGGAACAGCTCATGCTGGAGCTTCTGTTGATTTAGCTATTTTTT
CTCTTCATTTAGCTGGAATTTCCTCAATTTTAGGGGCAGTAAATTTTATTACAACTGTGA
TTAATATGCGATCGTCAGGGATTACTTTAGATCGACTACCCTTATTTGTTTGATCTGTAG
TTATTACAGCTATCTTATTACTTCTTTCTCTTCCTGTTTTAGCTGGAGCTATTACTATAT
TATTAACAGACCGAAACTTAAATACATCTTTCTTTGATCCAATCGGAGGGGGAGACCCTA
TT
>1-1
TCGGAACTTCTCTAAGAATTTTAATTCGTGCTGAACTTAGCCACCCTGGTATATTTATTG
GGAATGACCAAATTTATAATGTAATTGTAACAGCTCATGCATTTATTATAATTTTCTTTA
TAGTAATGCCAATTATAATTGGAGGATTTGGAAATTGATTAGTTCCTTTAATATTAGGAG
CCCCTGATATAGCTTTCCCTCGAATGAATAATATAAGTTTTTGAATACTACCTCCTTCAT
TGACTCTTCTATTATCAAGCTCAATAGTAGAAAATGGGGCAGGAACTGGGTGAACAGTTT
ATCCTCCTCTCTCTTCAGGAACAGCTCATGCTGGAGCTTCTGTTGATTTAGCTATTTTTT
CTCTTCATTTAGCTGGAATTTCCTCAATTTTAGGGGCAGTAAATTTTATTACAACTGTGA
TTAATATACGATCGTCAGGGATTACTTTAGATCGACTACCCTTATTTGTTTGATCTGTAG
TTATTACAGCTATCTTATTACTTCTTTCTCTTCCTGTTTTAGCTGGAGCTATTACTATAT
TATTAACAGACCGAAACTTAAATACATCTTTCTTTGATCCAATCGGAGGGGGAGACCCTA
TT
>1-2
TCGGAACTTCTCTAAGAATTTTAATTCGTGCTGAACTTAGCCACCCTGGTATATTTATTG
GGAATGACCAAATTTATAATGTAATTGTAACAGCTCATGCATTTATTATAATTTTCTTTA
TAGTAATGCCAATTATAATTGGAGGATTTGGAAATTGATTAGTTCCTTTAATATTAGGAG
CCCCTGATATAGCTTTCCCTCGAATGAATAATATAAGTTTTTGAATACTACCTCCTTCAT
TGACTCTTCTATTATCAAGCTCAATAGTAGAAAATGGGGCAGGAACTGGGTGAACAGTTT
ATCCTCCTCTCTCTTCAGGAACAGCTCATGCTGGAGCTTCTGTTGATTTAGCTATTTTTT
CTCTTCATTTAGCTGGAATTTCCTCAATTTTAGGGGCAGTAAATTTTATTACAACTGTGA
TTAATATACGATCGTCAGGGATTACTTTAGATCGACTACCCTTATTTGTTTGATCTGTAG
TTATTACAGCTATCTTATTACTTCTTTCTCTTCCTGTTTTAGCTGGAGCTATTACTATAT

TATTAACAGACCGAAACTTAAATACATCTTTCTTTGATCCAATCGGAGGGGGAGACCCTA
TT
>1-3
TCGGAACTTCTCTAAGAATTTTAATTCGTGCTGAACTTAGCCACCCTGGTATATTTATTG
GGAATGACCAAATTTATAATGTAATTGTAACAGCTCATGCATTTATTATAATTTTCTTTA
TAGTAATGCCAATTATAATTGGAGGATTTGGAAATTGATTAGTTCCTTTAATATTAGGAG
CCCCTGATATAGCTTTCCCTCGAATGAATAATATAAGTTTTTGAATACTACCTCCTTCAT
TGACTCTTCTATTATCAAGCTCAATAGTAGAAAATGGAGCAGGAACTGGATGAACAGTTT
ATCCTCCTCTTTCTTCAGGAACAGCTCATGCTGGAGCTTCTGTTGATTTAGCTATTTTTT
CTCTTCATTTAGCTGGAATTTCCTCAATTTTAGGGGCAGTAAATTTTATTACAACTGTAA
TTAATATACGATCGTCAGGGATTACTTTAGATCGACTACCCTTATTTGTTTGATCTGTAG
TTATTACAGCTATCTTATTACTTCTTTCTCTTCCTGTTTTAGCTGGAGCTATTACTATAT
TATTAACAGACCGAAACTTAAATACATCTTTCTTTGATCCAATCGGAGGGGGAGACCCTA
TT
>3-3
TCGGAACTTCTCTAAGAATTTTAATTCGTGCTGAACTTAGCCACCCTGGTATATTTATTG
GGAATGACCAAATTTATAATGTAATTGTAACAGCTCATGCATTTATTATAATTTTCTTTA
TAGTAATGCCAATTATAATTGGAGGATTTGGAAATTGATTAGTTCCTTTAATATTAGGAG
CCCCTGATATAGCTTTCCCTCGAATGAATAATATAAGTTTTTGAATACTACCTCCTTCAT
TGACTCTTCTATTATCAAGCTCAATAGTAGAAAATGGGGCAGGAACTGGGTGAACAGTTT
ATCCTCCTCTCTCTTCAGGAACAGCTCATGCTGGAGCTTCTGTTGATTTAGCTATTTTTT
CTCTTCATTTAGCTGGAATTTCCTCAATTTTAGGGGCAGTAAATTTTATTACAACTGTGA
TTAATATGCGATCGTCAGGGATTACTTTAGATCGACTACCCTTATTTGTTTGATCTGTAG
TTATTACAGCTATCTTATTACTTCTTTCTCTTCCTGTTTTAGCTGGAGCTATTACTATAT
TATTAACATACCGAAACTTAAATACATCTTTCTTTGATCCAATCGGAGGGGGAGACCCTA
TT
>4-1
TCGGAACTTCTCTAAGAATTTTAATTCGTGCTGAACTTAGCCACCCTGGTATATTTATTG
GGAATGACCAAATTTATAATGTAATTGTAACAGCTCATGCATTTATTATAATTTTCTTTA
TAGTAATGCCAATTATAATTGGAGGATTTGGAAATTGATTAGTTCCTTTAATATTAGGAG
CCCCTGATATAGCTTTCCCTCGAATGAATAATATAAGTTTTTGAATACTACCTCCTTCAT
TGACTCTTCTATTATCAAGCTCAATAGTAGAAAATGGGGCAGGAACTGGGTGAACAGTTT
ATCCTCCTCTCTCTTCAGGAACAGCTCATGCTGGAGCTTCTGTTGATTTAGCTATTTTTT
CTCTTCATTTAGCTGGAATTTCCTCAATTTTAGGGGCAGTAAATTTTATTACAACTGTGA
TTAATATGCGATCGTCAGGGATTACTTTAGATCGACTACCCTTATTTGTTTGATCTGTAG
TTATTACAGCTATCTTATTACTTCTTTCTCTTCCTGTTTTAGCTGGAGCTATTACTATAT
TATTAACAGACCGAAACTTAAATACATCTTTCTTTGATCCAATCGGAGGGGGAGACCCTA
TT
>4-2
TCGGAACTTCTCTAAGAATTTTAATTCGTGCTGAACTTAGCCACCCTGGTATATTTATTG
GGAATGACCAAATTTATAATGTAATTGTAACAGCTCATGCATTTATTATAATTTTCTTTA
TAGTAATGCCAATTATAATTGGAGGATTTGGAAATTGATTAGTTCCTTTAATATTAGGAG
CCCCTGATATAGCTTTCCCTCGAATGAATAATATAAGTTTTTGAATACTACCTCCTTCAT
TGACTCTTCTATTATCAAGCTCAATAGTAGAAAATGGGGCAGGAACTGGGTGAACAGTTT
ATCCTCCTCTCTCTTCAGGAACAGCTCATGCTGGAGCTTCTGTTGATTTAGCTATTTTTT
CTCTTCATTTAGCTGGAATTTCCTCAATTTTAGGGGCAGTAAATTTTATTACAACTGTGA
TTAATATGCGATCGTCAGGGATTACTTTAGATCGACTACCCTTATTTGTTTGATCTGTAG
TTATTACAGCTATCTTATTACTTCTTTCTCTTCCTGTTTTAGCTGGAGCTATTACTATAT
TATTAACAGACCGAAACTTAAATACATCTTTCTTTGATCCAATCGGAGGGGGAGACCCTA
TT
>4-3
TCGGAACTTCTCTAAGAATTTTAATTCGTGCTGAACTTAGCCACCCTGGTATATTTATTG
GGAATGACCAAATTTATAATGTAATTGTAACAGCTCATGCATTTATTATAATTTTCTTTA
TAGTAATGCCAATTATAATTGGAGGATTTGGAAATTGATTAGTTCCTTTAATATTAGGAG
CCCCTGATATAGCTTTCCCTCGAATGAATAATATAAGTTTTTGAATACTACCTCCTTCAT
TGACTCTTCTATTATCAAGCTCAATAGTAGAAAATGGGGCAGGAACTGGGTGAACAGTTT
ATCCTCCTCTCTCTTCAGGAACAGCTCATGCTGGAGCTTCTGTTGATTTAGCTATTTTTT

CTCTTCATTTAGCTGGAATTTCCTCAATTTTAGGGGCAGTAAATTTTATTACAACTGTGA
TTAATATGCGATCGTCAGGGATTACTTTAGATCGACTACCCTTATTTGTTTGATCTGTAG
TTATTACAGCTATCTTATTACTTCTTTCTCTTCCTGTTTTAGCTGGAGCTATTACTATAT
TATTAACAGACCGAAACTTAAATACATCTTTCTTTGATCCAATCGGAGGGGGAGACCCTA
TT
>6-1
TCGGAACTTCTCTAAGAATTTTAATTCGTGCTGAACTTAGCCACCCTGGTATATTTATTG
GGAATGACCAAATTTATAATGTAATTGTAACAGCTCATGCATTTATTATAATTTTCTTTA
TAGTAATGCCAATTATAATTGGAGGATTTGGAAATTGATTAGTTCCTTTAATATTAGGAG
CCCCTGATATAGCTTTCCCTCGAATGAATAATATAAGTTTTTGAATACTACCTCCTTCAT
TGACTCTTCTATTATCAAGCTCAATAGTAGAAAATGGAGCAGGAACTGGATGAACAGTTT
ATCCTCCTCTTTCTTCAGGAACAGCTCATGCTGGAGCTTCTGTTGATTTAGCTATTTTTT
CTCTTCATTTAGCTGGAATTTCCTCAATTTTAGGGGCAGTAAATTTTATTACAACTGTAA
TTAATATACGATCGTCAGGGATTACTTTAGATCGACTACCCTTATTTGTTTGATCTGTAG
TTATTACAGCTATCTTATTACTTCTTTCTCTTCCTGTTTTAGCTGGAGCTATTACTATAT
TATTAACAGACCGAAACTTAAATACATCTTTCTTTGATCCAATCGGAGGGGGAGACCCTA
TT
>6-2
TCGGAACTTCTCTAAGAATTTTAATTCGTGCTGAACTTAGCCACCCTGGTATATTTATTG
GGAATGACCAAATTTATAATGTAATTGTAACAGCTCATGCATTTATTATAATTTTCTTTA
TAGTAATGCCAATTATAATTGGAGGATTTGGAAATTGATTAGTTCCTTTAATATTAGGAG
CCCCTGATATAGCTTTCCCTCGAATGAATAATATAAGTTTTTGAATACTACCTCCTTCAT
TGACTCTTCTATTATCAAGCTCAATAGTAGAAAATGGGGCAGGAACTGGGTGAACAGTTT
ATCCTCCTCTCTCTTCAGGAACAGCTCATGCTGGAGCTTCTGTTGATTTAGCTATTTTTT
CTCTTCATTTAGCTGGAATTTCCTCAATTTTAGGGGCAGTAAATTTTATTACAACTGTGA
TTAATATGCGATCGTCAGGGATTACTTTAGATCGACTACCCTTATTTGTTTGATCTGTAG
TTATTACAGCTATCTTATTACTTCTTTCTCTTCCTGTTTTAGCTGGAGCTATTACTATAT
TATTAACAGACCGAAACTTAAATACATCTTTCTTTGATCCAATCGGAGGGGGAGACCCTA
TT
>6-3
TCGGAACTTCTCTAAGAATTTTAATTCGTGCTGAACTTAGCCACCCTGGTATATTTATTG
GGAATGACCAAATTTATAATGTAATTGTAACAGCTCATGCATTTATTATAATTTTCTTTA
TAGTAATGCCAATTATAATTGGAGGATTTGGAAATTGATTAGTTCCTTTAATATTAGGAG
CCCCTGATATAGCTTTCCCTCGAATGAATAATATAAGTTTTTGAATACTACCTCCTTCAT
TGACTCTTCTATTATCAAGCTCAATAGTAGAAAATGGGGCAGGAACTGGGTGAACAGTTT
ATCCTCCTCTCTCTTCAGGAACAGCTCATGCTGGAGCTTCTGTTGATTTAGCTATTTTTT
CTCTTCATTTAGCTGGAATTTCCTCAATTTTAGGGGCAGTAAATTTTATTACAACTGTGA
TTAATATGCGATCGTCAGGGATTACTTTAGATCGACTACCCTTATTTGTTTGATCTGTAG
TTATTACAGCTATCTTATTACTTCTTTCTCTTCCTGTTTTAGCTGGAGCTATTACTATAT
TATTAACAGACCGAAACTTAAATACATCTTTCTTTGATCCAATCGGAGGGGGAGACCCTA
TT
>10-1
TCGGAACTTCTCTAAGAATTTTAATTCGTGCTGAACTTAGCCACCCTGGTATATTTATTG
GGAATGACCAAATTTATAATGTAATTGTAACAGCTCATGCATTTATTATAATTTTCTTTA
TAGTAATGCCAATTATAATTGGAGGATTTGGAAATTGATTAGTTCCTTTAATATTAGGAG
CCCCTGATATAGCTTTCCCTCGAATGAATAATATAAGTTTTTGAATACTACCTCCTTCAT
TGACTCTTCTATTATCAAGCTCAATAGTAGAAAATGGGGCAGGAACTGGGTGAACAGTTT
ATCCTCCTCTCTCTTCAGGAACAGCTCATGCTGGAGCTTCTGTTGATTTAGCTATTTTTT
CTCTTCATTTAGCTGGAATTTCCTCAATTTTAGGGGCAGTAAATTTTATTACAACTGTGA
TTAATATGCGATCGTCAGGGATTACTTTAGATCGACTACCCTTATTTGTTTGATCTGTAG
TTATTACAGCTATCTTATTACTTCTTTCTCTTCCTGTTTTAGCTGGAGCTATTACTATAT
TATTAACAGACCGAAACTTAAATACATCTTTCTTTGATCCAATCGGAGGGGGAGACCCTA
TT
>10-2
TCGGAACTTCTCTAAGAATTTTAATTCGTGCTGAACTTAGCCACCCTGGTATATTTATTG
GGAATGACCAAATTTATAATGTAATTGTAACAGCTCATGCATTTATTATAATTTTCTTTA
TAGTAATGCCAATTATAATTGGAGGATTTGGAAATTGATTAGTTCCTTTAATATTAGGAG

CCCCTGATATAGCTTTCCCTCGAATGAATAATATAAGTTTTTGAATACTACCTCCTTCAT
TGACTCTTCTATTATCAAGCTCAATAGTAGAAAATGGGGCAGGAACTGGGTGAACAGTTT
ATCCTCCTCTCTCTTCAGGAACAGCTCATGCTGGAGCTTCTGTTGATTTAGCTATTTTTT
CTCTTCATTTAGCTGGAATTTCCTCAATTTTAGGGGCAGTAAATTTTATTACAACTGTGA
TTAATATGCGATCGTCAGGGATTACTTTAGATCGACTACCCTTATTTGTTTGATCTGTAG
TTATTACAGCTATCTTATTACTTCTTTCTCTTCCTGTTTTAGCTGGAGCTATTACTATAT
TATTAACAGACCGAAACTTAAATACATCTTTCTTTGATCCAATCGGAGGGGGAGACCCTA
TT
>10-3
TCGGAACTTCTCTAAGAATTTTAATTCGTGCTGAACTTAGCCACCCTGGTATATTTATTG
GGAATGACCAAATTTATAATGTAATTGTAACAGCTCATGCATTTATTATAATTTTCTTTA
TAGTAATGCCAATTATAATTGGAGGATTTGGAAATTGATTAGTTCCTTTAATATTAGGAG
CCCCTGATATAGCTTTCCCTCGAATGAATAATATAAGTTTTTGAATACTACCTCCTTCAT
TGACTCTTCTATTATCAAGCTCAATAGTAGAAAATGGGGCAGGAACTGGGTGAACAGTTT
ATCCTCCTCTCTCTTCAGGAACAGCTCATGCTGGAGCTTCTGTTGATTTAGCTATTTTTT
CTCTTCATTTAGCTGGAATTTCCTCAATTTTAGGGGCAGTAAATTTTATTACAACTGTGA
TTAATATGCGATCGTCAGGGATTACTTTAGATCGACTACCCTTATTTGTTTGATCTGTAG
TTATTACAGCTATCTTATTACTTCTTTCTCTTCCTGTTTTAGCTGGAGCTATTACTATAT
TATTAACAGACCGAAACTTAAATACATCTTTCTTTGATCCAATCGGAGGGGGAGACCCTA
TT
>13-1
TCGGAACTTCTCTAAGAATTTTAATTCGTGCTGAACTTAGCCACCCTGGTATATTTATTG
GGAATGACCAAATTTATAATGTAATTGTAACAGCTCATGCATTTATTATAATTTTCTTTA
TAGTAATGCCAATTATAATTGGAGGATTTGGAAATTGATTAGTTCCTTTAATATTAGGAG
CCCCTGATATAGCTTTCCCTCGAATGAATAATATAAGTTTTTGAATACTACCTCCTTCAT
TGACTCTTCTATTATCAAGCTCAATAGTAGAAAATGGAGCAGGAACTGGATGAACAGTTT
ATCCTCCTCTTTCTTCAGGAACAGCTCATGCTGGAGCTTCTGTTGATTTAGCTATTTTTT
CTCTTCATTTAGCTGGAATTTCCTCAATTTTAGGGGCAGTAAATTTTATTACAACTGTAA
TTAATATACGATCGTCAGGGATTACTTTAGATCGACTACCCTTATTTGTTTGATCTGTAG
TTATTACAGCTATCTTATTACTTCTTTCTCTTCCTGTTTTAGCTGGAGCTATTACTATAT
TATTAACAGACCGAAACTTAAATACATCTTTCTTTGATCCAATCGGAGGGGGAGACCCTA
TT
>13-2
TCGGAACTTCTCTAAGAATTTTAATTCGTGCTGAACTTAGCCACCCTGGTATATTTATTG
GGAATGACCAAATTTATAATGTAATTGTAACAGCTCATGCATTTATTATAATTTTCTTTA
TAGTAATGCCAATTATAATTGGAGGATTTGGAAATTGATTAGTTCCTTTAATATTAGGAG
CCCCTGATATAGCTTTCCCTCGAATGAATAATATAAGTTTTTGAATACTACCTCCTTCAT
TGACTCTTCTATTATCAAGCTCAATAGTAGAAAATGGGGCAGGAACTGGGTGAACAGTTT
ATCCTCCTCTCTCTTCAGGAACAGCTCATGCTGGAGCTTCTGTTGATTTAGCTATTTTTT
CTCTTCATTTAGCTGGAATTTCCTCAATTTTAGGGGCAGTAAATTTTATTACAACTGTGA
TTAATATGCGATCGTCAGGGATTACTTTAGATCGACTACCCTTATTTGTTTGATCTGTAG
TTATTACAGCTATCTTATTACTTCTTTCTCTTCCTGTTTTAGCTGGAGCTATTACTATAT
TATTAACAGACCGAAACTTAAATACATCTTTCTTTGATCCAATCGGAGGGGGAGACCCTA
TT
>13-3
TCGGAACTTCTCTAAGAATTTTAATTCGTGCTGAACTTAGCCACCCTGGTATATTTATTG
GGAATGACCAAATTTATAATGTAATTGTAACAGCTCATGCATTTATTATAATTTTCTTTA
TAGTAATGCCAATTATAATTGGAGGATTTGGAAATTGATTAGTTCCTTTAATATTAGGAG
CCCCTGATATAGCTTTCCCTCGAATGAATAATATAAGTTTTTGAATACTACCTCCTTCAT
TGACTCTTCTATTATCAAGCTCAATAGTAGAAAATGGGGCAGGAACTGGGTGAACAGTTT
ATCCTCCTCTCTCTTCAGGAACAGCTCATGCTGGAGCTTCTGTTGATTTAGCTATTTTTT
CTCTTCATTTAGCTGGAATTTCCTCAATTTTAGGGGCAGTAAATTTTATTACAACTGTGA
TTAATATGCGATCGTCAGGGATTACTTTAGATCGACTACCCTTATTTGTTTGATCTGTAG
TTATTACAGCTATCTTATTACTTCTTTCTCTTCCTGTTTTAGCTGGAGCTATTACTATAT
TATTAACAGACCGAAACTTAAATACATCTTTCTTTGATCCAATCGGAGGGGGAGACCCTA
TT
>13-4

TCGGAACTTCTCTAAGAATTTTAATTCGTGCTGAACTTAGCCACCCTGGTATATTTATTG
GGAATGACCAAATTTATAATGTAATTGTAACAGCTCATGCATTTATTATAATTTTCTTTA
TAGTAATGCCAATTATAATTGGAGGATTTGGAAATTGATTAGTTCCTTTAATATTAGGAG
CCCCTGATATAGCTTTCCCTCGAATGAATAATATAAGTTTTTGAATACTACCTCCTTCAT
TGACTCTTCTATTATCAAGCTCAATAGTAGAAAATGGGGCAGGAACTGGGTGAACAGTTT
ATCCTCCTCTCTCTTCAGGAACAGCTCATGCTGGAGCTTCTGTTGATTTAGCTATTTTTT
CTCTTCATTTAGCTGGAATTTCCTCAATTTTAGGGGCAGTAAATTTTATTACAACTGTGA
TTAATATGCGATCGTCAGGGATTACTTTAGATCGACTACCCTTATTTGTTTGATCTGTAG
TTATTACAGCTATCTTATTACTTCTTTCTCTTCCTGTTTTAGCTGGAGCTATTACTATAT
TATTAACAGACCGAAACTTAAATACATCTTTCTTTGATCCAATCGGAGGGGGAGACCCTA
TT
>13-5
TCGGAACTTCTCTAAGAATTTTAATTCGTGCTGAACTTAGCCACCCTGGTATATTTATTG
GGAATGACCAAATTTATAATGTAATTGTAACAGCTCATGCATTTATTATAATTTTCTTTA
TAGTAATGCCAATTATAATTGGAGGATTTGGAAATTGATTAGTTCCTTTAATATTAGGAG
CCCCTGATATAGCTTTCCCTCGAATGAATAATATAAGTTTTTGAATACTACCTCCTTCAT
TGACTCTTCTATTATCAAGCTCAATAGTAGAAAATGGAGCAGGAACTGGATGAACAGTTT
ATCCTCCTCTTTCTTCAGGAACAGCTCATGCTGGGGCTTCTGTTGATTTAGCTATTTTTT
CTCTTCATTTAGCTGGAATTTCCTCAATTTTAGGGGCAGTAAATTTTATTACAACTGTAA
TTAATATACGATCGTCAGGGATTACTTTAGATCGACTACCCTTATTTGTTTGATCTGTAG
TTATTACAGCTATCTTATTACTTCTTTCTCTTCCTGTTTTAGCTGGAGCTATTACTATAT
TATTAACAGACCGAAACTTAAATACATCTTTCTTTGATCCAATCGGAGGGGGAGACCCTA
TT
>13-6
TCGGAACTTCTCTAAGAATTTTAATTCGTGCTGAACTTAGCCACCCTGGTATATTTATTG
GGAATGACCAAATTTATAATGTAATTGTAACAGCTCATGCATTTATTATAATTTTCTTTA
TAGTAATGCCAATTATAATTGGAGGATTTGGAAATTGATTAGTTCCTTTAATATTAGGAG
CCCCTGATATAGCTTTCCCTCGAATGAATAATATAAGTTTTTGAATACTACCTCCTTCAT
TGACTCTTCTATTATCAAGCTCAATAGTAGAAAATGGAGCAGGAACTGGATGAACAGTTT
ATCCTCCTCTTTCTTCAGGAACAGCTCATGCTGGAGCTTCTGTTGATTTAGCTATTTTTT
CTCTTCATTTAGCTGGAATTTCCTCAATTTTAGGGGCAGTAAATTTTATTACAACTGTAA
TTAATATACGATCGTCAGGGATTACTTTAGATCGACTACCCTTATTTGTTTGATCTGTAG
TTATTACAGCTATCTTATTACTTCTTTCTCTTCCTGTTTTAGCTGGAGCTATTACTATAT
TATTAACATACCGAAACTTAAATACATCTTTCTTTGATCCAATCGGAGGGGGAGACCCTA
TT
>13-7
TCGGAACTTCTCTAAGAATTTTAATTCGTGCCGAACTTAGCCACCCTGGTATATTTATTG
GGAATGACCAAATTTATAATGTAATTGTAACAGCTCATGCATTTATTATAATTTTCTTTA
TAGTAATNCCAATTATAATTGGAGGATTTGGAAATTGATTAGTTCCTTTAATATTAGGAG
CCCCTGATATAGCTTTCCCTCGAATGAATAATATAAGTTTTTGAATACTACCTCCTTCAT
TGACTCTTCTATTATCAAGCTCAATAGTAGAAAATGGGGCAGGAACTGGGTGAACAGTTT
ATCCTCCTCTCTCTTCAGGAACAGCTCATGCTGGAGCTTCTGTTGATTTAGCTATTTTTT
CTCTTCATTTAGCTGGAATTTCCTCAATTTTAGGGGCAGTAAATTTTATTACAACTGTGA
TTAATATGCGATCGTCAGGGATTACTTTAGATCGACTACCCTTATTTGTTTGATCTGTAG
TTATTACAGCTATCTTATTACTTCTTTCTCTTCCTGTTTTAGCTGGAGCTATTACTATAT
TATTAACAGACCGAAACTTAAATACATCTTTCTTTGATCCAATCGGAGGGGGAGACCCTA
TT
>13-8
TCGGAACTTCTCTAAGAATTTTAATTCGTGCTGAACTTAGCCACCCTGGTATATTTATTG
GGAATGACCAAATTTATAATGTAATTGTAACAGCTCATGCATTTATTATAATTTTCTTTA
TAGTAATGCCAATTATAATTGGAGGATTTGGAAATTGATTAGTTCCTTTAATATTAGGAG
CCCCTGATATAGCTTTCCCTCGAATGAATAATATAAGTTTTTGAATACTACCTCCTTCAT
TGACTCTTCTATTATCAAGCTCAATAGTAGAAAATGGAGCAGGAACTGGATGAACAGTTT
ATCCTCCTCTTTCTTCAGGAACAGCTCATGCTGGAGCTTCTGTTGATTTAGCTATTTTTT
CTCTTCATTTAGCTGGAATTTCCTCAATTTTAGGGGCAGTAAATTTTATTACAACTGTAA
TTAATATACGATCGTCAGGGATTACTTTAGATCGACTACCCTTATTTGTTTGATCTGTAG
TTATTACAGCTATCTTATTACTTCTTTCTCTTCCTGTTTTAGCTGGAGCTATTACTATAT

TATTAACAGACCGAAACTTAAATACATCTTTCTTTGATCCAATCGGAGGGGGAGACCCTA
TT
>13-9
TCGGAACTTCTCTAAGAATTTTAATTCGTGCTGAACTTAGCCACCCTGGTATATTTATTG
GGAATGACCAAATTTATAATGTAATTGTAACAGCTCATGCATTTATTATAATTTTCTTTA
TAGTAATGCCAATTATAATTGGAGGATTTGGAAATTGATTAGTTCCTTTAATATTAGGAG
CCCCTGATATAGCTTTCCCTCGAATGAATAATATAAGTTTTTGAATACTACCTCCTTCAT
TGACTCTTCTATTATCAAGCTCAATAGTAGAAAATGGGGCAGGAACTGGGTGAACAGTTT
ATCCTCCTCTCTCTTCAGGAACAGCTCATGCTGGAGCTTCTGTTGATTTAGCTATTTTTT
CTCTTCATTTAGCTGGAATTTCCTCAATTTTAGGGGCAGTAAATTTTATTACAACTGTGA
TTAATATGCGATCGTCAGGGATTACTTTAGATCGACTACCCTTATTTGTTTGATCTGTAG
TTATTACAGCTATCTTATTACTTCTTTCTCTTCCTGTTTTAGCTGGAGCTATTACTATAT
TATTAACAGACCGAAACTTAAATACATCTTTCTTTGATCCAATCGGAGGGGGAGACCCTA
TT
>13-10
TCGGAACTTCTCTAAGAATTTTAATTCGTGCTGAACTTAGCCACCCTGGTATATTTATTG
GGAATGACCAAATTTATAATGTAATTGTAACAGCTCATGCATTTATTATAATTTTCTTTA
TAGTAATGCCAATTATAATTGGAGGATTTGGAAATTGATTAGTTCCTTTAATATTAGGAG
CCCCTGATATAGCTTTCCCTCGAATGAATAATATAAGTTTTTGAATACTACCTCCTTCAT
TGACTCTTCTATTATCAAGCTCAATAGTAGAAAATGGAGCAGGAACTGGATGAACAGTTT
ATCCTCCTCTTTCTTCAGGAACAGCTCATGCTGGAGCTTCTGTTGATTTAGCTATTTTTT
CTCTTCATTTAGCTGGAATTTCCTCAATTTTAGGGGCAGTAAATTTTATTACAACTGTAA
TTAATATACGATCGTCAGGGATTACTTTAGATCGACTACCCTTATTTGTTTGATCTGTAG
TTATTACAGCTATCTTATTACTTCTTTCTCTTCCTGTTTTAGCTGGAGCTATTACTATAT
TATTAACAGACCGAAACTTAAATACATCTTTCTTTGATCCAATCGGAGGGGGAGACCCTA
TT
>13-11
TCGGAACTTCTCTAAGAATTTTAATTCGTGCTGAACTTAGCCACCCTGGTATATTTATTG
GGAATGACCAAATTTATAATGTAATTGTAACAGCTCATGCATTTATTATAATTTTCTTTA
TAGTAATGCCAATTATAATTGGAGGATTTGGAAATTGATTAGTTCCTTTAATATTAGGAG
CCCCTGATATAGCTTTCCCTCGAATGAATAATATAAGTTTTTGAATACTACCTCCTTCAT
TGACTCTTCTATTATCAAGCTCAATAGTAGAAAATGGGGCAGGAACTGGGTGAACAGTTT
ATCCTCCTCTCTCTTCAGGAACAGCTCATGCTGGAGCTTCTGTTGATTTAGCTATTTTTT
CTCTTCATTTAGCTGGAATTTCCTCAATTTTAGGGGCAGTAAATTTTATTACAACTGTGA
TTAATATGCGATCGTCAGGGATTACTTTAGATCGACTACCCTTATTTGTTTGATCTGTAG
TTATTACAGCTATCTTATTACTTCTTTCTCTTCCTGTTTTAGCTGGAGCTATTACTATAT
TATTAACAGACCGAAACTTAAATACATCTTTCTTTGATCCAATTGGAGGGGGAGACCCTA
TT
>13-15
TCGGAACTTCTCTAAGAATTTTAATTCGTGCTGAACTTAGCCACCCTGGTATATTTATTG
GGAATGACCAAATTTATAATGTAATTGTAACAGCTCATGCATTTATTATAATTTTCTTTA
TAGTAATACCAATTATAATTGGAGGATTTGGAAATTGATTAGTTCCTTTAATATTAGGAG
CCCCTGATATAGCCTTTCCTCGAATAAATAATATAAGTTTTTGAATACTACCTCCTTCAT
TGACTCTTCTATTATCAAGCTCAATAGTAGAAAATGGGGCAGGAACTGGGTGAACAGTTT
ATCCTCCTCTCTCTTCAGGAACAGCTCATGCTGGAGCTTCTGTTGATTTAGCTATTTTTT
CTCTTCATTTAGCTGGAATTTCCTCAATTTTAGGGGCAGTAAATTTTATTACAACTGTAA
TTAATATACGATCGTCAGGAATTACTTTAGATCGACTACCTTTATTTGTTTGATCTGTAG
TTATTACAGCTATCTTATTACTTCTTTCTCTTCCTGTTTTAGCTGGAGCTATTACTATGT
TATTAACAGACCGAAACTTAAATACATCTTTCTTTGATCCAATCGGAGGAGGAGATCCTA
TT
>13-16
TCGGAACTTCTCTAAGAATTTTAATTCGTGCTGAACTTAGCCACCCTGGTATATTTATTG
GGAATGACCAAATTTATAATGTAATTGTAACAGCTCATGCATTTATTATAATTTTCTTTA
TAGTAATACCAATTATAATTGGAGGATTTGGAAATTGATTAGTTCCTTTAATATTAGGAG
CCCCTGATATAGCCTTTCCTCGAATAAATAATATAAGTTTTTGAATACTACCTCCTTCAT
TGACTCTTCTATTATCAAGCTCAATAGTAGAAAATGGGGCAGGAACTGGGTGAACAGTTT
ATCCTCCTCTCTCTTCAGGAACAGCTCATGCTGGAGCTTCTGTTGATTTAGCTATTTTTT

CTCTTCATTTAGCTGGAATTTCCTCAATTTTAGGGGCAGTAAATTTTATTACAACTGTAA
TTAATATACGATCGTCAGGAATTACTTTAGATCGACTACCTTTATTTGTTTGATCTGTAG
TTATTACAGCTATCTTATTACTTCTTTCTCTTCCTGTTTTAGCTGGAGCTATTACTATGT
TATTAACAGACCGAAACTTAAATACATCTTTCTTTGATCCAATCGGAGGAGGAGATCCTA
TT
>17-1
TCGGAACTTCTCTAAGAATTTTAATTCGTGCTGAACTTAGCCACCCTGGTATATTTATTG
GGAATGACCAAATTTATAATGTAATTGTAACAGCTCATGCATTTATTATAATTTTCTTTA
TAGTAATGCCAATTATAATTGGAGGATTTGGAAATTGATTAGTTCCTTTAATATTAGGAG
CCCCTGATATAGCTTTCCCTCGAATGAATAATATAAGTTTTTGAATACTACCTCCTTCAT
TGACTCTTCTATTATCAAGCTCAATAGTAGAAAATGGGGCAGGAACTGGGTGAACAGTTT
ATCCTCCTCTCTCTTCAGGAACAGCTCATGCTGGAGCTTCTGTTGATTTAGCTATTTTTT
CTCTTCATTTAGCTGGAATTTCCTCAATTTTAGGGGCAGTAAATTTTATTACAACTGTGA
TTAATATGCGATCGTCAGGGATTACTTTAGATCGACTACCCTTATTTGTTTGATCTGTAG
TTATTACAGCTATCTTATTACTTCTTTCTCTTCCTGTTTTAGCTGGAGCTATTACTATAT
TATTAACAGACCGAAACTTAAATACATCTTTCTTTGATCCAATCGGAGGGGGGGACCCTA
TT
>17-2
TCGGAACTTCTCTAAGAATTTTAATTCGTGCTGAACTTAGCCACCCTGGTATATTTATTG
GGAATGACCAAATTTATAATGTAATTGTAACAGCTCATGCATTTATTATAATTTTCTTTA
TAGTAATACCAATTATAATTGGAGGATTTGGAAATTGATTAGTTCCTTTAATATTAGGAG
CCCCTGATATAGCCTTTCCTCGAATAAATAATATAAGTTTTTGAATACTACCTCCTTCAT
TGACTCTTCTATTATCAAGCTCAATAGTAGAAAATGGGGCAGGAACTGGGTGAACAGTTT
ATCCTCCTCTCTCTTCAGGAACAGCTCATGCTGGAGCTTCTGTTGATTTAGCTATTTTTT
CTCTTCATTTAGCTGGAATTTCCTCAATTTTAGGGGCAGTAAATTTTATTACAACTGTAA
TTAATATACGATCGTCAGGAATTACTTTAGATCGACTACCTTTATTTGTTTGATCTGTAG
TTATTACAGCTATCTTATTACTTCTTTCTCTTCCTGTTTTAGCTGGAGCTATTACTATGT
TATTAACAGACCGAAACTTAAATACATCTTTCTTTGATCCAATCGGAGGAGGAGATCCTA
TT
>17-3
TCGGAACTTCTCTAAGAATTTTAATTCGTGCTGAACTTAGCCACCCTGGTATATTTATTG
GGAATGACCAAATTTATAATGTAATTGTAACAGCTCATGCATTTATTATAATTTTCTTTA
TAGTAATGCCAATTATAATTGGAGGATTTGGAAATTGATTAGTTCCTTTAATATTAGGAG
CCCCTGATATAGCTTTCCCTCGAATGAATAATATAAGTTTTTGAATACTACCTCCTTCAT
TGACTCTTCTATTATCAAGCTCAATAGTAGAAAATGGGGCAGGAACTGGGTGAACAGTTT
ATCCTCCTCTCTCTTCAGGAACAGCTCATGCTGGAGCTTCTGTTGATTTAGCTATTTTTT
CTCTTCATTTAGCTGGAATTTCCTCAATTTTAGGGGCAGTAAATTTTATTACAACTGTGA
TTAATATGCGATCGTCAGGGATTACTTTAGATCGACTACCCTTATTTGTTTGATCTGTAG
TTATTACAGCTATCTTATTACTTCTTTCTCTTCCTGTTTTAGCTGGAGCTATTACTATAT
TATTAACAGACCGAAACTTAAATACATCTTTCTTTGATCCAATCGGAGGGGGGGACCCTA
TT
>18-1
TCGGAACTTCTCTAAGAATTTTAATTCGTGCTGAACTTAGCCACCCTGGTATATTTATTG
GGAATGACCAAATTTATAATGTAATTGTAACAGCTCATGCATTTATTATAATTTTCTTTA
TAGTAATGCCAATTATAATTGGAGGATTTGGAAATTGATTAGTTCCTTTAATATTAGGAG
CCCCTGATATAGCTTTCCCTCGAATGAATAATATAAGTTTTTGAATACTACCTCCTTCAT
TGACTCTTCTATTATCAAGCTCAATAGTAGAAAATGGGGCAGGAACTGGGTGAACAGTTT
ATCCTCCTCTCTCTTCAGGAACAGCTCATGCTGGAGCTTCTGTTGATTTAGCTATTTTTT
CTCTTCATTTAGCTGGAATTTCCTCAATTTTAGGGGCAGTAAATTTTATTACAACTGTGA
TTAATATGCGATCGTCAGGGATTACTTTAGATCGACTACCCTTATTTGTTTGATCTGTAG
TTATTACAGCTATCTTATTACTTCTTTCTCTTCCTGTTTTAGCTGGAGCTATTACTATAT
TATTAACAGACCGAAACTTAAATACATCTTTCTTTGATCCAATCGGAGGGGGAGACCCTA
TT
>18-2
TCGGAACTTCTCTAAGAATTTTAATTCGTGCTGAACTTAGCCACCCTGGTATATTTATTG
GGAATGACCAAATTTATAATGTAATTGTAACAGCTCATGCATTTATTATAATTTTCTTTA
TAGTAATGCCAATTATAATTGGAGGATTTGGAAATTGATTAGTTCCTTTAATATTAGGAG

CCCCTGATATAGCTTTCCCTCGAATGAATAATATAAGTTTTTGAATACTACCTCCTTCAT
TGACTCTTCTATTATCAAGCTCAATAGTAGAAAATGGGGCAGGAACTGGGTGAACAGTTT
ATCCTCCTCTCTCTTCAGGAACAGCTCATGCTGGAGCTTCTGTTGATTTAGCTATTTTTT
CTCTTCATTTAGCTGGAATTTCCTCAATTTTAGGGGCAGTAAATTTTATTACAACTGTGA
TTAATATGCGATCGTCAGGGATTACTTTAGATCGACTACCCTTATTTGTTTGATCTGTAG
TTATTACAGCTATCTTATTACTTCTTTCTCTTCCTGTTTTAGCTGGAGCTATTACTATAT
TATTAACAGACCGAAACTTAAATACATCTTTCTTTGATCCAATCGGAGGGGGAGACCCTA
TT
>18-3
TCGGAACTTCTCTAAGAATTTTAATTCGTGCTGAACTTAGCCACCCTGGTATATTTATTG
GGAATGACCAAATTTATAATGTAATTGTAACAGCTCATGCATTTATTATAATTTTCTTTA
TAGTAATGCCAATTATAATTGGAGGATTTGGAAATTGATTAGTTCCTTTAATATTAGGAG
CCCCTGATATAGCTTTCCCTCGAATGAATAATATAAGTTTTTGAATACTACCTCCTTCAT
TGACTCTTCTATTATCAAGCTCAATAGTAGAAAATGGGGCAGGAACTGGGTGAACAGTTT
ATCCTCCTCTCTCTTCAGGAACAGCTCATGCTGGAGCTTCTGTTGATTTAGCTATTTTTT
CTCTTCATTTAGCTGGAATTTCCTCAATTTTAGGGGCAGTAAATTTTATTACAACTGTGA
TTAATATGCGATCGTCAGGGATTACTTTAGATCGACTACCCTTATTTGTTTGATCTGTAG
TTATTACAGCTATCTTATTACTTCTTTCTCTTCCTGTTTTAGCTGGAGCTATTACTATAT
TATTAACAGACCGAAACTTAAATACATCTTTCTTTGATCCAATCGGAGGGGGAGACCCTA
TT
>21-1
TCGGAACTTCTCTAAGAATTTTAATTCGTGCTGAACTTAGCCACCCTGGTATATTTATTG
GGAATGACCAAATTTATAATGTAATTGTAACAGCTCATGCATTTATTATAATTTTCTTTA
TAGTAATGCCAATTATAATTGGAGGATTTGGAAATTGATTAGTTCCTTTAATATTAGGAG
CCCCTGATATAGCTTTCCCTCGAATGAATAATATAAGTTTTTGAATACTACCTCCTTCAT
TGACTCTTCTATTATCAAGCTCAATAGTAGAAAATGGGGCAGGAACTGGGTGAACAGTTT
ATCCTCCTCTCTCTTCAGGAACAGCTCATGCTGGAGCTTCTGTTGATTTAGCTATTTTTT
CTCTTCATTTAGCTGGAATTTCCTCAATTTTAGGGGCAGTAAATTTTATTACAACTGTGA
TTAATATACGATCGTCAGGGATTACTTTAGATCGACTACCCTTATTTGTTTGATCTGTAG
TTATTACAGCTATCTTATTACTTCTTTCTCTTCCTGTTTTAGCTGGAGCTATTACTATAT
TATTAACAGACCGAAACTTAAATACATCTTTCTTTGATCCAATCGGAGGGGGAGACCCTA
TT
>21-2
TCGGAACTTCTCTAAGAATTTTAATTCGTGCTGAACTTAGCCACCCTGGTATATTTATTG
GGAATGACCAAATTTATAATGTAATTGTAACAGCTCATGCATTTATTATAATTTTCTTTA
TAGTAATGCCAATTATAATTGGAGGATTTGGAAATTGATTAGTTCCTTTAATATTAGGAG
CCCCTGATATAGCTTTCCCTCGAATGAATAATATAAGTTTTTGAATACTACCTCCTTCAT
TGACTCTTCTATTATCAAGCTCAATAGTAGAAAATGGGGCAGGAACTGGGTGAACAGTTT
ATCCTCCTCTCTCTTCAGGAACAGCTCATGCTGGAGCTTCTGTTGATTTAGCTATTTTTT
CTCTTCATTTAGCTGGAATTTCCTCAATTTTAGGGGCAGTAAATTTTATTACAACTGTGA
TTAATATGCGATCGTCAGGGATTACTTTAGATCGACTACCCTTATTTGTTTGATCTGTAG
TTATTACAGCTATCTTATTACTTCTTTCTCTTCCTGTTTTAGCTGGAGCTATTACTATAT
TATTAACAGACCGAAACTTAAATACATCTTTCTTTGATCCAATCGGAGGGGGAGACCCTA
TT
>21-3
TCGGAACTTCTCTAAGAATTTTAATTCGTGCTGAACTTAGCCACCCTGGTATATTTATTG
GGAATGACCAAATTTATAATGTAATTGTAACAGCTCATGCATTTATTATAATTTTCTTTA
TAGTAATTCCAATTATAATTGGAGGATTTGGAAATTGATTAGTTCCTTTAATATTAGGAG
CCCCTGATATAGCTTTCCCTCGAATGAATAATATAAGTTTTTGAATACTACCTCCTTCAT
TGACTCTTCTATTATCAAGCTCAATAGTAGAAAATGGGGCAGGAACTGGGTGAACAGTTT
ATCCTCCTCTCTCTTCAGGAACAGCTCATGCTGGAGCTTCTGTTGATTTAGCTATTTTTT
CTCTTCATTTAGCTGGAATTTCCTCAATTTTAGGGGCAGTAAATTTTATTACAACTGTGA
TTAATATGCGATCGTCAGGGATTACTTTAGATCGACTACCCTTATTTGTTTGATCTGTAG
TTATTACAGCTATCTTATTACTTCTTTCTCTTCCTGTTTTAGCTGGAGCTATTACTATAT
TATTAACAGACCGAAACTTAAATACATCTTTCTTTGATCCAATCGGAGGGGGAGACCCTA
TT
>21-4

TCGGAACTTCTCTAAGAATTTTAATTCGTGCTGAACTTAGCCACCCTGGTATATTTATTG
GGAATGACCAAATTTATAATGTAATTGTAACAGCTCATGCATTTATTATAATTTTCTTTA
TAGTAATGCCAATTATAATTGGAGGATTTGGAAATTGATTAGTTCCTTTAATATTAGGAG
CCCCTGATATAGCTTTCCCTCGAATGAATAATATAAGTTTTTGAATACTACCTCCTTCAT
TGACTCTTCTATTATCAAGCTCAATAGTAGAAAATGGGGCAGGAACTGGGTGAACAGTTT
ATCCTCCTCTCTCTTCAGGAACAGCTCATGCTGGAGCTTCTGTTGATTTAGCTATTTTTT
CTCTTCATTTAGCTGGAATTTCCTCAATTTTAGGGGCAGTAAATTTTATTACAACTGTGA
TTAATATACGATCGTCAGGGATTACTTTAGATCGACTACCCTTATTTGTTTGATCTGTAG
TTATTACAGCTATCTTATTACTTCTTTCTCTTCCTGTTTTAGCTGGAGCTATTACTATAT
TATTAACAGACCGAAACTTAAATACATCTTTCTTTGATCCAATCGGAGGGGGAGACCCTA
TT
>21-5
TCGGAACTTCTCTAAGAATTTTAATTCGTGCTGAACTTAGCCACCCTGGTATATTTATTG
GGAATGACCAAATTTATAATGTAATTGTAACAGCTCATGCATTTATTATAATTTTCTTTA
TAGTAATGCCAATTATAATTGGAGGATTTGGAAATTGATTAGTTCCTTTAATATTAGGAG
CCCCTGATATAGCTTTCCCTCGAATGAATAATATAAGTTTTTGAATACTACCTCCTTCAT
TGACTCTTCTATTATCAAGCTCAATAGTAGAAAATGGAGCAGGAACTGGATGAACAGTTT
ATCCTCCTCTTTCTTCAGGAACAGCTCATGCTGGAGCTTCTGTTGATTTAGCTATTTTTT
CTCTTCATTTAGCTGGAATTTCCTCAATTTTAGGGGCAGTAAATTTTATTACAACTGTAA
TTAATATACGATCGTCAGGGATTACTTTAGATCGACTACCCTTATTTGTTTGATCTGTAG
TTATTACAGCTATCTTATTACTTCTTTCTCTTCCTGTTTTAGCTGGAGCTATTACTATAT
TATTAACAGACCGAAACTTAAATACATCTTTCTTTGATCCAATCGGAGGAGGAGACCCTA
TT
>21-6
TCGGAACTTCTCTAAGAATTTTAATTCGTGCTGAACTTAGCCACCCTGGTATATTTATTG
GGAATGACCAAATTTATAATGTAATTGTAACAGCTCATGCATTTATTATAATTTTCTTTA
TAGTAATGCCAATTATAATTGGAGGATTTGGAAATTGATTAGTTCCTTTAATATTAGGAG
CCCCTGATATAGCTTTCCCTCGAATGAATAATATAAGTTTTTGAATACTACCTCCTTCAT
TGACTCTTCTATTATCAAGCTCAATAGTAGAAAATGGGGCAGGAACTGGGTGAACAGTTT
ATCCTCCTCTCTCTTCAGGAACAGCTCATGCTGGAGCTTCTGTTGATTTAGCTATTTTTT
CTCTTCATTTAGCTGGAATTTCCTCAATTTTAGGGGCAGTAAATTTTATTACAACTGTGA
TTAATATGCGATCGTCAGGGATTACTTTAGATCGACTACCCTTATTTGTTTGATCTGTAG
TTATTACAGCTATCTTATTACTTCTTTCTCTTCCTGTTTTAGCTGGAGCTATTACTATAT
TATTAACAGACCGAAACTTAAATACATCTTTCTTTGATCCAATCGGAGGGGGAGACCCTA
TT
>21-7
TCGGAACTTCTCTAAGAATTTTAATTCGTGCTGAACTTAGCCACCCTGGTATATTTATTG
GGAATGACCAAATTTATAATGTAATTGTAACAGCTCATGCATTTATTATAATTTTCTTTA
TAGTAATGCCAATTATAATTGGAGGATTTGGAAATTGATTAGTTCCTTTAATATTAGGAG
CCCCTGATATAGCTTTCCCTCGAATGAATAATATAAGTTTTTGAATACTACCTCCTTCAT
TGACTCTTCTATTATCAAGCTCAATAGTAGAAAATGGGGCAGGAACTGGGTGAACAGTTT
ATCCTCCTCTCTCTTCAGGAACAGCTCATGCTGGAGCTTCTGTTGATTTAGCTATTTTTT
CTCTTCATTTAGCTGGAATTTCCTCAATTTTAGGGGCAGTAAATTTTATTACAACTGTGA
TTAATATACGATCGTCAGGGATTACTTTAGATCGACTACCCTTATTTGTTTGATCTGTAG
TTATTACAGCTATCTTATTACTTCTTTCTCTTCCTGTTTTAGCTGGAGCTATTACTATAT
TATTAACAGACCGAAACTTAAATACATCTTTCTTTGATCCAATCGGAGGGGGAGACCCTA
TT
>21-8
TCGGAACTTCTCTAAGAATTTTAATTCGTGCTGAACTTAGCCACCCTGGTATATTTATTG
GGAATGACCAAATTTATAATGTAATTGTAACAGCTCATGCATTTATTATAATTTTCTTTA
TAGTAATGCCAATTATAATTGGAGGATTTGGAAATTGATTAGTTCCTTTAATATTAGGAG
CCCCTGATATAGCTTTCCCTCGAATGAATAATATAAGTTTTTGAATACTACCTCCTTCAT
TGACTCTTTTATTATCAAGCTCAATAGTAGAAAATGGGGCAGGAACTGGGTGAACAGTTT
ATCCTCCTCTCTCTTCAGGAACAGCTCATGCTGGAGCTTCTGTTGATTTAGCTATTTTTT
CTCTTCATTTAGCTGGAATTTCCTCAATTTTAGGGGCAGTAAATTTTATTACAACTGTGA
TTAATATGCGATCGTCAGGGATTACTTTAGATCGACTACCCTTATTTGTTTGATCTGTAG
TTATTACAGCTATCTTATTACTTCTTTCTCTTCCTGTTTTAGCTGGAGCTATTACTATAT

TATTAACAGACCGAAACTTAAATACATCTTTCTTTGATCCAATCGGAGGGGGAGACCCTA
TT
>21-9
TCGGAACTTCTCTAAGAATTTTAATTCGTGCTGAACTTAGCCACCCTGGTATATTTATTG
GGAATGACCAAATTTATAATGTAATTGTAACAGCTCATGCATTTATTATAATTTTCTTTA
TAGTAATGCCAATTATAATTGGAGGATTTGGAAATTGATTAGTTCCTTTAATATTAGGAG
CCCCTGATATAGCTTTCCCTCGAATGAATAATATAAGTTTTTGAATACTACCTCCTTCAT
TGACTCTTCTATTATCAAGCTCAATAGTAGAAAATGGGGCAGGAACTGGGTGAACAGTTT
ATCCTCCTCTCTCTTCAGGAACAGCTCATGCTGGAGCTTCTGTTGATTTAGCTATTTTTT
CTCTTCATTTAGCTGGAATTTCCTCAATTTTAGGGGCAGTAAATTTTATTACAACTGTGA
TTAATATACGATCGTCAGGGATTACTTTAGATCGACTACCCTTATTTGTTTGATCTGTAG
TTATTACAGCTATCTTATTACTTCTTTCTCTTCCTGTTTTAGCTGGAGCTATTACTATAT
TATTAACAGACCGAAACTTAAATACATCTTTCTTTGATCCAATCGGAGGGGGAGACCCTA
TT
>21-10
TCGGAACTTCTCTAAGAATTTTAATTCGTGCTGAACTTAGCCACCCTGGTATATTTATTG
GGAATGACCAAATTTATAATGTAATTGTAACAGCTCATGCATTTATTATAATTTTCTTTA
TAGTAATGCCAATTATAATTGGAGGATTTGGAAATTGATTAGTTCCTTTAATATTAGGAG
CCCCTGATATAGCTTTCCCTCGAATGAATAATATAAGTTTTTGAATACTACCTCCTTCAT
TGACTCTTCTATTATCAAGCTCAATAGTAGAAAATGGGGCAGGAACTGGGTGAACAGTTT
ATCCTCCTCTCTCTTCAGGAACAGCTCATGCTGGAGCTTCTGTTGATTTAGCTATTTTTT
CTCTTCATTTAGCTGGAATTTCCTCAATTTTAGGGGCAGTAAATTTTATTACAACTGTGA
TTAATATGCGATCGTCAGGGATTACTTTAGATCGACTACCCTTATTTGTTTGATCTGTAG
TTATTACAGCTATCTTATTACTTCTTTCTCTTCCTGTTTTAGCTGGAGCTATTACTATAT
TATTAACAGACCGAAACTTAAATACATCTTTCTTTGATCCAATCGGAGGGGGAGACCCTA
TT
>21-13
TCGGAACTTCTCTAAGAATTTTAATTCGTGCTGAACTTAGCCACCCTGGTATATTTATTG
GGAATGACCAAATTTATAATGTAATTGTAACAGCTCATGCATTTATTATAATTTTCTTTA
TAGTAATGCCAATTATAATTGGAGGATTTGGAAATTGATTAGTTCCTTTAATATTAGGAG
CCCCTGATATAGCTTTCCCTCGAATGAATAATATAAGTTTTTGAATACTACCTCCTTCAT
TGACTCTTCTATTATCAAGCTCAATAGTAGAAAATGGGGCAGGAACTGGGTGAACAGTTT
ATCCTCCTCTCTCTTCAGGAACAGCTCATGCTGGAGCTTCTGTTGATTTAGCTATTTTTT
CTCTTCATTTAGCTGGAATTTCCTCAATTTTAGGGGCAGTAAATTTTATTACAACTGTGA
TTAATATGCGATCGTCAGGGATTACTTTAGATCGACTACCCTTATTTGTTTGATCTGTAG
TTATTACAGCTATCTTATTACTTCTTTCTCTTCCTGTTTTAGCTGGAGCTATTACTATAT
TATTAACAGACCGAAACTTAAATACATCTTTCTTTGATCCAATCGGAGGGGGAGACCCTA
TT
>21-14
TCGGAACTTCTCTAAGAATTTTAATTCGTGCTGAACTTAGCCACCCTGGTATATTTATTG
GGAATGACCAAATTTATAATGTAATTGTAACAGCTCATGCATTTATTATAATTTTCTTTA
TAGTAATGCCAATTATAATTGGAGGATTTGGAAATTGATTAGTTCCTTTAATATTAGGAG
CCCCTGATATAGCTTTCCCTCGAATGAATAATATAAGTTTTTGAATACTACCTCCTTCAT
TGACTCTTCTATTATCAAGCTCAATAGTAGAAAATGGGGCAGGAACTGGGTGAACAGTTT
ATCCTCCTCTCTCTTCAGGAACAGCTCATGCTGGAGCTTCTGTTGATTTAGCTATTTTTT
CTCTTCATTTAGCTGGAATTTCCTCAATTTTAGGGGCAGTAAATTTTATTACAACTGTGA
TTAATATGCGATCGTCAGGGATTACTTTAGATCGACTACCCTTATTTGTTTGATCTGTAG
TTATTACAGCTATCTTATTACTTCTTTCTCTTCCTGTTTTAGCTGGAGCTATTACTATAT
TATTAACAGACCGAAACTTAAATACATCTTTCTTTGATCCAATCGGAGGGGGAGACCCTA
TT
>21-15
TCGGAACTTCTCTAAGAATTTTAATTCGTGCTGAACTTAGCCACCCTGGTATATTTATTG
GGAATGACCAAATTTATAATGTAATTGTAACAGCTCATGCATTTATTATAATTTTCTTTA
TAGTAATGCCAATTATAATTGGAGGATTTGGAAATTGATTAGTTCCTTTAATATTAGGAG
CCCCTGATATAGCTTTCCCTCGAATGAATAATATAAGTTTTTGAATACTACCTCCTTCAT
TGACTCTTCTATTATCAAGCTCAATAGTAGAAAATGGGGCAGGAACTGGGTGAACAGTTT
ATCCTCCTCTCTCTTCAGGAACAGCTCATGCTGGAGCTTCTGTTGATTTAGCTATTTTTT

CTCTTCATTTAGCTGGAATTTCCTCAATTTTAGGGGCAGTAAATTTTATTACAACTGTGA
TTAATATGCGATCGTCAGGGATTACTTTAGATCGACTACCCTTATTTGTTTGATCTGTAG
TTATTACAGCTATCTTATTACTTCTTTCTCTTCCTGTTTTAGCTGGAGCTATTACTATAT
TATTAACAGACCGAAACTTAAATACATCTTTCTTTGATCCAATCGGAGGGGGAGACCCTA
TT
>21-16
TCGGAACTTCTCTAAGAATTTTAATTCGTGCTGAACTTAGCCACCCTGGTATATTTATTG
GGAATGACCAAATTTATAATGTAATTGTAACAGCTCATGCATTTATTATAATTTTCTTTA
TAGTAATGCCAATTATAATTGGAGGATTTGGAAATTGATTAGTTCCTTTAATATTAGGAG
CCCCTGATATAGCTTTCCCTCGAATGAATAATATAAGTTTTTGAATACTACCTCCTTCAT
TGACTCTTCTATTATCAAGCTCAATAGTAGAAAATGGGGCAGGAACTGGGTGAACAGTTT
ATCCTCCTCTCTCTTCAGGAACAGCTCATGCTGGAGCTTCTGTTGATTTAGCTATTTTTT
CTCTTCATTTAGCTGGAATTTCCTCAATTTTAGGGGCAGTAAATTTTATTACAACTGTGA
TTAATATACGATCGTCAGGGATTACTTTAGATCGACTACCCTTATTTGTTTGATCTGTAG
TTATTACAGCTATCTTATTACTTCTTTCTCTTCCTGTTTTAGCTGGAGCTATTACTATAT
TATTAACAGACCGAAACTTAAATACATCTTTCTTTGATCCAATCGGAGGGGGAGACCCTA
TT
>22-1
TCGGAACTTCTCTAAGAATTTTAATTCGTGCTGAACTTAGCCACCCTGGTATATTTATTG
GGAATGACCAAATTTATAATGTAATTGTAACAGCTCATGCATTTATTATAATTTTCTTTA
TAGTAATGCCAATTATAATTGGAGGATTTGGAAATTGATTAGTTCCTTTAATATTAGGAG
CCCCTGATATAGCTTTCCCTCGAATGAATAATATAAGTTTTTGAATACTACCTCCTTCAT
TGACTCTTCTATTATCAAGCTCAATAGTAGAAAATGGGGCAGGAACTGGGTGAACAGTTT
ATCCTCCTCTCTCTTCAGGAACAGCTCATGCTGGAGCTTCTGTTGATTTAGCTATTTTTT
CTCTTCATTTAGCTGGAATTTCCTCAATTTTAGGGGCAGTAAATTTTATTACAACTGTGA
TTAATATGCGATCGTCAGGGATTACTTTAGATCGACTACCCTTATTTGTTTGATCTGTAG
TTATTACAGCTATCTTATTACTTCTTTCTCTTCCTGTTTTAGCTGGAGCTATTACTATAT
TATTAACAGACCGAAACTTAAATACATCTTTCTTTGATCCAATCGGAGGGGGAGACCCTA
TT
>22-2
TCGGAACTTCTCTAAGAATTTTAATTCGTGCTGAACTTAGCCACCCTGGTATATTTATTG
GGAATGACCAAATTTATAATGTAATTGTAACAGCTCATGCATTTATTATAATTTTCTTTA
TAGTAATGCCAATTATAATTGGAGGATTTGGAAATTGATTAGTTCCTTTAATATTAGGAG
CCCCTGATATAGCTTTCCCTCGAATGAATAATATAAGTTTTTGAATACTACCTCCTTCAT
TGACTCTTCTATTATCAAGCTCAATAGTAGAAAATGGGGCAGGAACTGGGTGAACAGTTT
ATCCTCCTCTCTCTTCAGGAACAGCTCATGCTGGAGCTTCTGTTGATTTAGCTATTTTTT
CTCTTCATTTAGCTGGAATTTCCTCAATTTTAGGGGCAGTAAATTTTATTACAACTGTGA
TTAATATGCGATCGTCAGGGATTACTTTAGATCGACTACCCTTATTTGTTTGATCTGTAG
TTATTACAGCTATCTTATTACTTCTTTCTCTTCCTGTTTTAGCTGGAGCTATTACTATAT
TATTAACAGACCGAAACTTAAATACATCTTTCTTTGATCCAATCGGAGGGGGAGACCCTA
TT
>22-3
TCGGAACTTCTCTAAGAATTTTAATTCGTGCTGAACTTAGCCACCCTGGTATATTTATTG
GGAATGACCAAATTTATAATGTAATTGTAACAGCTCATGCATTTATTATAATTTTCTTTA
TAGTAATGCCAATTATAATTGGAGGATTTGGAAATTGATTAGTTCCTTTAATATTAGGAG
CCCCTGATATAGCTTTCCCTCGAATGAATAATATAAGTTTTTGAATACTACCTCCTTCAT
TGACTCTTCTATTATCAAGCTCAATAGTAGAAAATGGGGCAGGAACTGGGTGAACAGTTT
ATCCTCCTCTCTCTTCAGGAACAGCTCATGCTGGAGCTTCTGTTGATTTAGCTATTTTTT
CTCTTCATTTAGCTGGAATTTCCTCAATTTTAGGGGCAGTAAATTTTATTACAACTGTGA
TTAATATACGATCGTCAGGGATTACTTTAGATCGACTACCCTTATTTGTTTGATCTGTAG
TTATTACAGCTATCTTATTACTTCTTTCTCTTCCTGTTTTAGCTGGAGCTATTACTATAT
TATTAACAGACCGAAACTTAAATACATCTTTCTTTGATCCAATCGGAGGGGGAGACCCTA
TT
>22-4
TCGGAACTTCTCTAAGAATTTTAATTCGTGCTGAACTTAGCCACCCTGGTATATTTATTG
GGAATGACCAAATTTATAATGTAATTGTAACAGCTCATGCATTTATTATAATTTTCTTTA
TAGTAATGCCAATTATAATTGGAGGATTTGGAAATTGATTAGTTCCTTTAATATTAGGAG

CCCCTGATATAGCTTTCCCTCGAATGAATAATATAAGTTTTTGAATACTACCTCCTTCAT
TGACTCTTCTATTATCAAGCTCAATAGTAGAAAATGGGGCAGGAACTGGGTGAACAGTTT
ATCCTCCTCTCTCTTCAGGAACAGCTCATGCTGGAGCTTCTGTTGATTTAGCTATTTTTT
CTCTTCATTTAGCTGGAATTTCCTCAATTTTAGGGGCAGTAAATTTTATTACAACTGTGA
TTAATATACGATCGTCAGGGATTACTTTAGATCGACTACCCTTATTTGTTTGATCTGTAG
TTATTACAGCTATCTTATTACTTCTTTCTCTTCCTGTTTTAGCTGGAGCTATTACTATAT
TATTAACAGACCGAAACTTAAATACATCTTTCTTTGATCCAATCGGAGGGGGAGACCCTA
TT
>22-5
TCGGAACTTCTCTAAGAATTTTAATTCGTGCTGAACTTAGCCACCCTGGTATATTTATTG
GGAATGACCAAATTTATAATGTAATTGTAACAGCTCATGCATTTATTATAATTTTCTTTA
TAGTAATGCCAATTATAATTGGAGGATTTGGAAATTGATTAGTTCCTTTAATATTAGGAG
CCCCTGATATAGCTTTCCCTCGAATGAATAATATAAGTTTTTGAATACTACCTCCTTCAT
TGACTCTTCTATTATCAAGCTCAATAGTAGAAAATGGGGCAGGAACTGGGTGAACAGTTT
ATCCTCCTCTCTCTTCAGGAACAGCTCATGCTGGAGCTTCTGTTGATTTAGCTATTTTTT
CTCTTCATTTAGCTGGAATTTCCTCAATTTTAGGGGCAGTAAATTTTATTACAACTGTGA
TTAATATACGATCGTCAGGGATTACTTTAGATCGACTACCCTTATTTGTTTGATCTGTAG
TTATTACAGCTATCTTATTACTTCTTTCTCTTCCTGTTTTAGCTGGAGCTATTACTATAT
TATTAACAGACCGAAACTTAAATACATCTTTCTTTGATCCAATCGGAGGGGGAGACCCTA
TT
>22-6
TCGGAACTTCTCTAAGAATTTTAATTCGTGCTGAACTTAGCCACCCTGGTATATTTATTG
GGAATGACCAAATTTATAATGTAATTGTAACAGCTCATGCATTTATTATAATTTTCTTTA
TAGTAATGCCAATTATAATTGGAGGATTTGGAAATTGATTAGTTCCTTTAATATTAGGAG
CCCCTGATATAGCTTTCCCTCGAATGAATAATATAAGTTTTTGAATACTACCTCCTTCAT
TGACTCTTCTATTATCAAGCTCAATAGTAGAAAATGGGGCAGGAACTGGGTGAACAGTTT
ATCCTCCTCTCTCTTCAGGAACAGCTCATGCTGGAGCTTCTGTTGATTTAGCTATTTTTT
CTCTTCATTTAGCTGGAATTTCCTCAATTTTAGGGGCAGTAAATTTTATTACAACTGTGA
TTAATATGCGATCGTCAGGGATTACTTTAGATCGACTACCCTTATTTGTTTGATCTGTAG
TTATTACAGCTATCTTATTACTTCTTTCTCTTCCTGTTTTAGCTGGAGCTATTACTATAT
TATTAACAGACCGAAACTTAAATACATCTTTCTTTGATCCAATCGGAGGGGGAGACCCTA
TT
>22-7
TCGGAACTTCTCTAAGAATTTTAATTCGTGCTGAACTTAGCCACCCTGGTATATTTATTG
GGAATGACCAAATTTATAATGTAATTGTAACAGCTCATGCATTTATTATAATTTTCTTTA
TAGTAATGCCAATTATAATTGGAGGATTTGGAAATTGATTAGTTCCTTTAATATTAGGAG
CCCCTGATATAGCTTTCCCTCGAATGAATAATATAAGTTTTTGAATACTACCTCCTTCAT
TGACTCTTCTATTATCAAGCTCAATAGTAGAAAATGGGGCAGGAACTGGGTGAACAGTTT
ATCCTCCTCTCTCTTCAGGAACAGCTCATGCTGGAGCTTCTGTTGATTTAGCTATTTTTT
CTCTTCATTTAGCTGGAATTTCCTCAATTTTAGGGGCAGTAAATTTTATTACAACTGTGA
TTAATATACGATCGTCAGGGATTACTTTAGATCGACTACCCTTATTTGTTTGATCTGTAG
TTATTACAGCTATCTTATTACTTCTTTCTCTTCCTGTTTTAGCTGGAGCTATTACTATAT
TATTAACAGACCGAAACTTAAATACATCTTTCTTTGATCCAATCGGAGGGGGAGACCCTA
TT
>22-8
TCGGAACTTCTCTAAGAATTTTAATTCGTGCTGAACTTAGCCACCCTGGTATATTTATTG
GGAATGACCAAATTTATAATGTAATTGTAACAGCTCATGCATTTATTATAATTTTCTTTA
TAGTAATGCCAATTATAATTGGAGGATTTGGAAATTGATTAGTTCCTTTAATATTAGGAG
CCCCTGATATAGCTTTCCCTCGAATGAATAATATAAGTTTTTGAATACTACCTCCTTCAT
TGACTCTTCTATTATCAAGCTCAATAGTAGAAAATGGGGCAGGAACTGGGTGAACAGTTT
ATCCTCCTCTCTCTTCAGGAACAGCTCATGCTGGAGCTTCTGTTGATTTAGCTATTTTTT
CTCTTCATTTAGCTGGAATTTCCTCAATTTTAGGGGCAGTAAATTTTATTACAACTGTGA
TTAATATGCGATCGTCAGGGATTACTTTAGATCGACTACCCTTATTTGTTTGATCTGTAG
TTATTACAGCTATCTTATTACTTCTTTCTCTTCCTGTTTTAGCTGGAGCTATTACTATAT
TATTAACAGACCGAAACTTAAATACATCTTTCTTTGATCCAATCGGAGGGGGAGACCCTA
TT
>22-9

TCGGAACTTCTCTAAGAATTTTAATTCGTGCTGAACTTAGCCACCCTGGTATATTTATTG
GGAATGACCAAATTTATAATGTAATTGTAACAGCTCATGCATTTATTATAATTTTCTTTA
TAGTAATGCCAATTATAATTGGAGGATTTGGAAATTGATTAGTTCCTTTAATATTAGGAG
CCCCTGATATAGCTTTCCCTCGAATGAATAATATAAGTTTTTGAATACTACCTCCTTCAT
TGACTCTTCTATTATCAAGCTCAATAGTAGAAAATGGGGCAGGAACTGGGTGAACAGTTT
ATCCTCCTCTCTCTTCAGGAACAGCTCATGCTGGAGCTTCTGTTGATTTAGCTATTTTTT
CTCTTCATTTAGCTGGAATTTCCTCAATTTTAGGGGCAGTAAATTTTATTACAACTGTGA
TTAATATGCGATCGTCAGGGATTACTTTAGATCGACTACCCTTATTTGTTTGATCTGTAG
TTATTACAGCTATCTTATTACTTCTTTCTCTTCCTGTTTTAGCTGGAGCTATTACTATAT
TATTAACAGACCGAAACTTAAATACATCTTTCTTTGATCCAATCGGAGGGGGAGACCCTA
TT
>22-10
TCGGAACTTCTCTAAGAATTTTAATTCGTGCTGAACTTAGCCACCCTGGTATATTTATTG
GGAATGACCAAATTTATAATGTAATTGTAACAGCTCATGCATTTATTATAATTTTCTTTA
TAGTAATGCCAATTATAATTGGAGGATTTGGAAATTGATTAGTTCCTTTAATATTAGGAG
CCCCTGATATAGCTTTCCCTCGAATGAATAATATAAGTTTTTGAATACTACCTCCTTCAT
TGACTCTTCTATTATCAAGCTCAATAGTAGAAAATGGGGCAGGAACTGGGTGAACAGTTT
ATCCTCCTCTCTCTTCAGGAACAGCTCATGCTGGAGCTTCTGTTGATTTAGCTATTTTTT
CTCTTCATTTAGCTGGAATTTCCTCAATTTTAGGGGCAGTAAATTTTATTACAACTGTGA
TTAATATGCGATCGTCAGGGATTACTTTAGATCGACTACCCTTATTTGTTTGATCTGTAG
TTATTACAGCTATCTTATTACTTCTTTCTCTTCCTGTTTTAGCTGGAGCTATTACTATAT
TATTAACAGACCGAAACTTAAATACATCTTTCTTTGATCCAATCGGAGGGGGAGACCCTA
TT
>22-11
TCGGAACTTCTCTAAGAATTTTAATTCGTGCTGAACTTAGCCACCCTGGTATATTTATTG
GGAATGACCAAATTTATAATGTAATTGTAACAGCTCATGCATTTATTATAATTTTCTTTA
TAGTAATGCCAATTATAATTGGAGGATTTGGAAATTGATTAGTTCCTTTAATATTAGGAG
CCCCTGATATAGCTTTCCCTCGAATGAATAATATAAGTTTTTGAATACTACCTCCTTCAT
TGACTCTTCTATTATCAAGCTCAATAGTAGAAAATGGGGCAGGAACTGGGTGAACAGTTT
ATCCTCCTCTCTCTTCAGGAACAGCTCATGCTGGAGCTTCTGTTGATTTAGCTATTTTTT
CTCTTCATTTAGCTGGAATTTCCTCAATTTTAGGGGCAGTAAATTTTATTACAACTGTGA
TTAATATACGATCGTCAGGGATTACTTTAGATCGACTACCCTTATTTGTTTGATCTGTAG
TTATTACAGCTATCTTATTACTTCTTTCTCTTCCTGTTTTAGCTGGAGCTATTACTATAT
TATTAACAGACCGAAACTTAAATACATCTTTCTTTGATCCAATCGGAGGGGGAGACCCTA
TT
>22-12
TCGGAACTTCTCTAAGAATTTTAATTCGTGCTGAACTTAGCCACCCTGGTATATTTATTG
GGAATGACCAAATTTATAATGTAATTGTAACAGCTCATGCATTTATTATAATTTTCTTTA
TAGTAATGCCAATTATAATTGGAGGATTTGGAAATTGATTAGTTCCTTTAATATTAGGAG
CCCCTGATATAGCTTTCCCTCGAATGAATAATATAAGTTTTTGAATACTACCTCCTTCAT
TGACTCTTCTATTATCAAGCTCAATAGTAGAAAATGGGGCAGGAACTGGGTGAACAGTTT
ATCCTCCTCTCTCTTCAGGAACAGCTCATGCTGGAGCTTCTGTTGATTTAGCTATTTTTT
CTCTTCATTTAGCTGGAATTTCCTCAATTTTAGGGGCAGTAAATTTTATTACAACTGTGA
TTAATATGCGATCGTCAGGGATTACTTTAGATCGACTACCCTTATTTGTTTGATCTGTAG
TTATTACAGCTATCTTATTACTTCTTTCTCTTCCTGTTTTAGCTGGAGCTATTACTATAT
TATTAACAGACCGAAACTTAAATACATCTTTCTTTGATCCAATCGGAGGGGGAGACCCTA
TT
>22-13
TCGGAACTTCTCTAAGAATTTTAATTCGTGCTGAACTTAGCCACCCTGGTATATTTATTG
GGAATGACCAAATTTATAATGTAATTGTAACAGCTCATGCATTTATTATAATTTTCTTTA
TAGTAATGCCAATTATAATTGGAGGATTTGGAAATTGATTAGTTCCTTTAATATTAGGAG
CCCCTGATATAGCTTTCCCTCGAATGAATAATATAAGTTTTTGAATACTACCTCCTTCAT
TGACTCTTCTATTATCAAGCTCAATAGTAGAAAATGGGGCAGGAACTGGGTGAACAGTTT
ATCCTCCTCTCTCTTCAGGAACAGCTCATGCTGGAGCTTCTGTTGATTTAGCTATTTTTT
CTCTTCATTTAGCTGGAATTTCCTCAATTTTAGGGGCAGTAAATTTTATTACAACTGTGA
TTAATATACGATCGTCAGGGATTACTTTAGATCGACTACCCTTATTTGTTTGATCTGTAG
TTATTACAGCTATCTTATTACTTCTTTCTCTTCCTGTTTTAGCTGGAGCTATTACTATAT

TATTAACAGACCGAAACTTAAATACATCTTTCTTTGATCCAATCGGAGGGGGAGACCCTA
TT
>22-14
TCGGAACTTCTCTAAGAATTTTAATTCGTGCTGAACTTAGCCACCCTGGTATATTTATTG
GGAATGACCAAATTTATAATGTAATTGTAACAGCTCATGCATTTATTATAATTTTCTTTA
TAGTAATGCCAATTATAATTGGAGGATTTGGAAATTGATTAGTTCCTTTAATATTAGGAG
CCCCTGATATAGCTTTCCCTCGAATGAATAATATAAGTTTTTGAATACTACCTCCTTCAT
TGACTCTTCTATTATCAAGCTCAATAGTAGAAAATGGGGCAGGAACTGGGTGAACAGTTT
ATCCTCCTCTCTCTTCAGGAACAGCTCATGCTGGAGCTTCTGTTGATTTAGCTATTTTTT
CTCTTCATTTAGCTGGAATTTCCTCAATTTTAGGGGCAGTAAATTTTATTACAACTGTGA
TTAATATACGATCGTCAGGGATTACTTTAGATCGACTACCCTTATTTGTTTGATCTGTAG
TTATTACAGCTATCTTATTACTTCTTTCTCTTCCTGTTTTAGCTGGAGCTATTACTATAT
TATTAACAGACCGAAACTTAAATACATCTTTCTTTGATCCAATCGGAGGGGGAGACCCTA
TT
>22-15
TCGGAACTTCTCTAAGAATTTTAATTCGTGCTGAACTTAGCCACCCTGGTATATTTATTG
GGAATGACCAAATTTATAATGTAATTGTAACAGCTCATGCATTTATTATAATTTTCTTTA
TAGTAATGCCAATTATAATTGGAGGATTTGGAAATTGATTAGTTCCTTTAATATTAGGAG
CCCCTGATATAGCTTTCCCTCGAATGAATAATATAAGTTTTTGAATACTACCTCCTTCAT
TGACTCTTCTATTATCAAGCTCAATAGTAGAAAATGGGGCAGGAACTGGGTGAACAGTTT
ATCCTCCTCTCTCTTCAGGAACAGCTCATGCTGGAGCTTCTGTTGATTTAGCTATTTTTT
CTCTTCATTTAGCTGGAATTTCCTCAATTTTAGGGGCAGTAAATTTTATTACAACTGTGA
TTAATATACGATCGTCAGGGATTACTTTAGATCGACTACCCTTATTTGTTTGATCTGTAG
TTATTACAGCTATCTTATTACTTCTTTCTCTTCCTGTTTTAGCTGGAGCTATTACTATAT
TATTAACAGACCGAAACTTAAATACATCTTTCTTTGATCCAATCGGAGGGGGAGACCCTA
TT
>22-16
TCGGAACTTCTCTAAGAATTTTAATTCGTGCTGAACTTAGCCACCCTGGTATATTTATTG
GGAATGACCAAATTTATAATGTAATTGTAACAGCTCATGCATTTATTATAATTTTCTTTA
TAGTAATGCCAATTATAATTGGAGGATTTGGAAATTGATTAGTTCCTTTAATATTAGGAG
CCCCTGATATAGCTTTCCCTCGAATGAATAATATAAGTTTTTGAATACTACCTCCTTCAT
TGACTCTTCTATTATCAAGCTCAATAGTAGAAAATGGGGCAGGAACTGGGTGAACAGTTT
ATCCTCCTCTCTCTTCAGGAACAGCTCATGCTGGAGCTTCTGTTGATTTAGCTATTTTTT
CTCTTCATTTAGCTGGAATTTCCTCAATTTTAGGGGCAGTAAATTTTATTACAACTGTGA
TTAATATGCGATCGTCAGGGATTACTTTAGATCGACTACCCTTATTTGTTTGATCTGTAG
TTATTACAGCTATCTTATTACTTCTTTCTCTTCCTGTTTTAGCTGGAGCTATTACTATAT
TATTAACAGACCGAAACTTAAATACATCTTTCTTTGATCCAATCGGAGGGGGAGACCCTA
TT
>23-1
TCGGAACTTCTCTAAGAATTTTAATTCGTGCTGAACTTAGCCACCCTGGTATATTTATTG
GGAATGACCAAATTTATAATGTAATTGTAACAGCTCATGCATTTATTATAATTTTCTTTA
TAGTAATGCCAATTATAATTGGAGGATTTGGAAATTGATTAGTTCCTTTAATATTAGGAG
CCCCTGATATAGCTTTCCCTCGAATGAATAATATAAGTTTTTGAATACTACCTCCTTCAT
TGACTCTTCTATTATCAAGCTCAATAGTAGAAAATGGGGCAGGAACTGGGTGAACAGTTT
ATCCTCCTCTCTCTTCAGGAACAGCTCATGCTGGAGCTTCTGTTGATTTAGCTATTTTTT
CTCTTCATTTAGCTGGAATTTCCTCAATTTTAGGGGCAGTAAATTTTATTACAACTGTGA
TTAATATGCGATCGTCAGGGATTACTTTAGATCGACTACCCTTATTTGTTTGATCTGTAG
TTATTACAGCTATCTTATTACTTCTTTCTCTTCCTGTTTTAGCTGGAGCTATTACTATAT
TATTAACAGACCGAAACTTAAATACATCTTTCTTTGATCCAATCGGAGGGGGAGACCCTA
TT
>23-2
TCGGAACTTCTCTAAGAATTTTAATTCGTGCTGAACTTAGCCACCCTGGTATATTTATTG
GGAATGACCAAATTTATAATGTAATTGTAACAGCTCATGCATTTATTATAATTTTCTTTA
TAGTAATGCCAATTATAATTGGAGGATTTGGAAATTGATTAGTTCCTTTAATATTAGGAG
CCCCTGATATAGCTTTCCCTCGAATGAATAATATAAGTTTTTGAATACTACCTCCTTCAT
TGACTCTTCTATTATCAAGCTCAATAGTAGAAAATGGGGCAGGAACTGGGTGAACAGTTT
ATCCTCCTCTCTCTTCAGGAACAGCTCATGCTGGAGCTTCTGTTGATTTAGCTATTTTTT

CTCTTCATTTAGCTGGAATTTCCTCAATTTTAGGGGCAGTAAATTTTATTACAACTGTGA
TTAATATGCGATCGTCAGGGATTACTTTAGATCGACTACCCTTATTTGTTTGATCTGTAG
TTATTACAGCTATCTTATTACTTCTTTCTCTTCCTGTTTTAGCTGGAGCTATTACTATAT
TATTAACAGACCGAAACTTAAATACATCTTTCTTTGATCCAATCGGAGGGGGAGACCCTA
TT
>23-3
TCGGAACTTCTCTAAGAATTTTAATTCGTGCTGAACTTAGCCACCCTGGTATATTTATTG
GGAATGACCAAATTTATAATGTAATTGTAACAGCTCATGCATTTATTATAATTTTCTTTA
TAGTAATGCCAATTATAATTGGAGGATTTGGAAATTGATTAGTTCCTTTAATATTAGGAG
CCCCTGATATAGCTTTCCCTCGAATGAATAATATAAGTTTTTGAATACTACCTCCTTCAT
TGACTCTTCTATTATCAAGCTCAATAGTAGAAAATGGGGCAGGAACTGGGTGAACAGTTT
ATCCTCCTCTCTCTTCAGGAACAGCTCATGCTGGAGCTTCTGTTGATTTAGCTATTTTTT
CTCTTCATTTAGCTGGAATTTCCTCAATTTTAGGGGCAGTAAATTTTATTACAACTGTGA
TTAATATGCGATCGTCAGGGATTACTTTAGATCGACTACCCTTATTTGTTTGATCTGTAG
TTATTACAGCTATCTTATTACTTCTTTCTCTTCCTGTTTTAGCTGGAGCTATTACTATAT
TATTAACAGACCGAAACTTAAATACATCTTTCTTTGATCCAATCGGAGGGGGAGACCCTA
TT
>27-1
TCGGAACTTCTCTAAGAATTTTAATTCGTGCTGAACTTAGCCACCCTGGTATATTTATTG
GGAATGACCAAATTTATAATGTAATTGTAACAGCTCATGCATTTATTATAATTTTCTTTA
TAGTAATGCCAATTATAATTGGAGGATTTGGAAATTGATTAGTTCCTTTAATATTAGGAG
CCCCTGATATAGCTTTCCCTCGAATGAATAATATAAGTTTTTGAATACTACCTCCTTCAT
TGACTCTTCTATTATCAAGCTCAATAGTAGAAAATGGGGCAGGAACTGGGTGAACAGTTT
ATCCTCCTCTCTCTTCAGGAACAGCTCATGCTGGAGCTTCTGTTGATTTAGCTATTTTTT
CTCTTCATTTAGCTGGAATTTCCTCAATTTTAGGGGCAGTAAATTTTATTACAACTGTGA
TTAATATACGATCGTCAGGGATTACTTTAGATCGACTACCCTTATTTGTTTGATCTGTAG
TTATTACAGCTATCTTATTACTTCTTTCTCTTCCTGTTTTAGCTGGAGCTATTACTATAT
TATTAACAGACCGAAACTTAAATACATCTTTCTTTGATCCAATCGGAGGGGGAGACCCTA
TT
>27-2
TCGGAACTTCTCTAAGAATTTTAATTCGTGCTGAACTTAGCCACCCTGGTATATTTATTG
GGAATGACCAAATTTATAATGTAATTGTAACAGCTCATGCATTTATTATAATTTTCTTTA
TAGTAATGCCAATTATAATTGGAGGATTTGGAAATTGATTAGTTCCTTTAATATTAGGAG
CCCCTGATATAGCTTTCCCTCGAATGAATAATATAAGTTTTTGAATACTACCTCCTTCAT
TGACTCTTCTATTATCAAGCTCAATAGTAGAAAATGGGGCAGGAACTGGGTGAACAGTTT
ATCCTCCTCTCTCTTCAGGAACAGCTCATGCTGGAGCTTCTGTTGATTTAGCTATTTTTT
CTCTTCATTTAGCTGGAATTTCCTCAATTTTAGGGGCAGTAAATTTTATTACAACTGTGA
TTAATATACGATCGTCAGGGATTACTTTAGATCGACTACCCTTATTTGTTTGATCTGTAG
TTATTACAGCTATCTTATTACTTCTTTCTCTTCCTGTTTTAGCTGGAGCTATTACTATAT
TATTAACAGACCGAAACTTAAATACATCTTTCTTTGATCCAATCGGAGGGGGAGACCCTA
TT
>27-3
TCGGAACTTCTCTAAGAATTTTAATTCGTGCTGAACTTAGCCACCCTGGTATATTTATTG
GGAATGACCAAATTTATAATGTAATTGTAACAGCTCATGCATTTATTATAATTTTCTTTA
TAGTAATGCCAATTATAATTGGAGGATTTGGAAATTGATTAGTTCCTTTAATATTAGGAG
CCCCTGATATAGCTTTCCCTCGAATGAATAATATAAGTTTTTGAATACTACCTCCTTCAT
TGACTCTTCTATTATCAAGCTCAATAGTAGAAAATGGGGCAGGAACTGGGTGAACAGTTT
ATCCTCCTCTCTCTTCAGGAACAGCTCATGCTGGAGCTTCTGTTGATTTAGCTATTTTTT
CTCTTCATTTAGCTGGAATTTCCTCAATTTTAGGGGCAGTAAATTTTATTACAACTGTGA
TTAATATACGATCGTCAGGGATTACTTTAGATCGACTACCCTTATTTGTTTGATCTGTAG
TTATTACAGCTATCTTATTACTTCTTTCTCTTCCTGTTTTAGCTGGAGCTATTACTATAT
TATTAACAGACCGAAACTTAAATACATCTTTCTTTGATCCAATCGGAGGGGGAGACCCTA
TT
>29-1
TCGGAACTTCTCTAAGAATTTTAATTCGTGCTGAACTTAGCCACCCTGGTATATTTATTG
GGAATGACCAAATTTATAATGTAATTGTAACAGCTCATGCATTTATTATAATTTTCTTTA
TAGTAATGCCAATTATAATTGGAGGATTTGGAAATTGATTAGTTCCTTTAATATTAGGAG

CCCCTGATATAGCTTTCCCTCGAATGAATAATATAAGTTTTTGAATACTACCTCCTTCAT
TGACTCTTCTATTATCAAGCTCAATAGTAGAAAATGGGGCAGGAACTGGGTGAACAGTTT
ATCCTCCTCTCTCTTCAGGAACAGCTCATGCTGGAGCTTCTGTTGATTTAGCTATTTTTT
CTCTTCATTTAGCTGGAATTTCCTCAATTTTAGGGGCAGTAAATTTTATTACAACTGTGA
TTAATATACGATCGTCAGGGATTACTTTAGATCGACTACCCTTATTTGTTTGATCTGTAG
TTATTACAGCTATCTTATTACTTCTTTCTCTTCCTGTTTTAGCTGGAGCTATTACTATAT
TATTAACAGACCGAAACTTAAATACATCTTTCTTTGATCCAATCGGAGGGGGAGACCCTA
TT
>29-2
TCGGAACTTCTCTAAGAATTTTAATTCGTGCTGAACTTAGCCACCCTGGTATATTTATTG
GGAATGACCAAATTTATAATGTAATTGTAACAGCTCATGCATTTATTATAATTTTCTTTA
TAGTAATGCCAATTATAATTGGAGGATTTGGAAATTGATTAGTTCCTTTAATATTAGGAG
CCCCTGATATAGCTTTCCCTCGAATGAATAATATAAGTTTTTGAATACTACCTCCTTCAT
TGACTCTTCTATTATCAAGCTCAATAGTAGAAAATGGGGCAGGAACTGGGTGAACAGTTT
ATCCTCCTCTCTCTTCAGGAACAGCTCATGCTGGAGCTTCTGTTGATTTAGCTATTTTTT
CTCTTCATTTAGCTGGAATTTCCTCAATTTTAGGGGCAGTAAATTTTATTACAACTGTGA
TTAATATACGATCGTCAGGGATTACTTTAGATCGACTACCCTTATTTGTTTGATCTGTAG
TTATTACAGCTATCTTATTACTTCTTTCTCTTCCTGTTTTAGCTGGAGCTATTACTATAT
TATTAACAGACCGAAACTTAAATACATCTTTCTTTGATCCAATCGGAGGGGGAGACCCTA
TT
>30-1
TCGGAACTTCTCTAAGAATTTTAATTCGTGCTGAACTTAGCCACCCTGGTATATTTATTG
GGAATGACCAAATTTATAATGTAATTGTAACAGCTCATGCATTTATTATAATTTTCTTTA
TAGTAATGCCAATTATAATTGGAGGATTTGGAAATTGATTAGTTCCTTTAATATTAGGAG
CCCCTGATATAGCTTTCCCTCGAATGAATAATATAAGTTTTTGAATACTACCTCCTTCAT
TGACTCTTCTATTATCAAGCTCAATAGTAGAAAATGGGGCAGGAACTGGGTGAACAGTTT
ATCCTCCTCTCTCTTCAGGAACAGCTCATGCTGGAGCTTCTGTTGATTTAGCTATTTTTT
CTCTTCATTTAGCTGGAATTTCCTCAATTTTAGGGGCAGTAAATTTTATTACAACTGTGA
TTAATATGCGATCGTCAGGGATTACTTTAGATCGACTACCCTTATTTGTTTGATCTGTAG
TTATTACAGCTATCTTATTACTTCTTTCTCTTCCTGTTTTAGCTGGAGCTATTACTATAT
TATTAACAGACCGAAACTTAAATACATCTTTCTTTGATCCAATCGGAGGGGGAGACCCTA
TT
>30-2
TCGGAACTTCTCTAAGAATTTTAATTCGTGCTGAACTTAGCCACCCTGGTATATTTATTG
GGAATGACCAAATTTATAATGTAATTGTAACAGCTCATGCATTTATTATAATTTTCTTTA
TAGTAATGCCAATTATAATTGGAGGATTTGGAAATTGATTAGTTCCTTTAATATTAGGAG
CCCCTGATATAGCTTTCCCTCGAATGAATAATATAAGTTTTTGAATACTACCTCCTTCAT
TGACTCTTCTATTATCAAGCTCAATAGTAGAAAATGGGGCAGGAACTGGGTGAACAGTTT
ATCCTCCTCTCTCTTCAGGAACAGCTCATGCTGGAGCTTCTGTTGATTTAGCTATTTTTT
CTCTTCATTTAGCTGGAATTTCCTCAATTTTAGGGGCAGTAAATTTTATTACAACTGTGA
TTAATATGCGATCGTCAGGGATTACTTTAGATCGACTACCCTTATTTGTTTGATCTGTAG
TTATTACAGCTATCTTATTACTTCTTTCTCTTCCTGTTTTAGCTGGAGCTATTACTATAT
TATTAACAGACCGAAACTTAAATACATCTTTCTTTGATCCAATCGGAGGGGGAGACCCTA
TT
>30-3
TCGGAACTTCTCTAAGAATTTTAATTCGTGCTGAACTTAGCCACCCTGGTATATTTATTG
GGAATGACCAAATTTATAATGTAATTGTAACAGCTCATGCATTTATTATAATTTTCTTTA
TAGTAATGCCAATTATAATTGGAGGATTTGGAAATTGATTAGTTCCTTTAATATTAGGAG
CCCCTGATATAGCTTTCCCTCGAATGAATAATATAAGTTTTTGAATACTACCTCCTTCAT
TGACTCTTCTATTATCAAGCTCAATAGTAGAAAATGGGGCAGGAACTGGGTGAACAGTTT
ATCCTCCTCTCTCTTCAGGAACAGCTCATGCTGGAGCTTCTGTTGATTTAGCTATTTTTT
CTCTTCATTTAGCTGGAATTTCCTCAATTTTAGGGGCAGTAAATTTTATTACAACTGTGA
TTAATATGCGATCGTCAGGGATTACTTTAGATCGACTACCCTTATTTGTTTGATCTGTAG
TTATTACAGCTATCTTATTACTTCTTTCTCTTCCTGTTTTAGCTGGAGCTATTACTATAT
TATTAACAGACCGAAACTTAAATACATCTTTCTTTGATCCAATCGGAGGGGGAGACCCTA
TT
>31-1

TCGGAACTTCTCTAAGAATTTTAATTCGTGCTGAACTTAGCCACCCTGGTATATTTATTG
GGAATGACCAAATTTATAATGTAATTGTAACAGCTCATGCATTTATTATAATTTTCTTTA
TAGTAATGCCAATTATAATTGGAGGATTTGGAAATTGATTAGTTCCTTTAATATTAGGAG
CCCCTGATATAGCTTTCCCTCGAATGAATAATATAAGTTTTTGAATACTACCTCCTTCAT
TGACTCTTCTATTATCAAGCTCAATAGTAGAAAATGGGGCAGGAACTGGGTGAACAGTTT
ATCCTCCTCTCTCTTCAGGAACAGCTCATGCTGGAGCTTCTGTTGATTTAGCTATTTTTT
CTCTTCATTTAGCTGGAATTTCCTCAATTTTAGGGGCAGTAAATTTTATTACAACTGTGA
TTAATATGCGATCGTCAGGGATTACTTTAGATCGACTACCCTTATTTGTTTGATCTGTAG
TTATTACAGCTATCTTATTACTTCTTTCTCTTCCTGTTTTAGCTGGAGCTATTACTATAT
TATTAACAGACCGAAACTTAAATACATCTTTCTTTGATCCAATCGGAGGGGGAGACCCTA
TT
>31-2
TCGGAACTTCTCTAAGAATTTTAATTCGTGCTGAACTTAGCCACCCTGGTATATTTATTG
GGAATGACCAAATTTATAATGTAATTGTAACAGCTCATGCATTTATTATAATTTTCTTTA
TAGTAATACCAATTATAATTGGAGGATTTGGAAATTGATTAGTTCCTTTAATATTAGGAG
CCCCTGATATAGCCTTTCCTCGAATAAATAATATAAGTTTTTGAATACTACCTCCTTCAT
TGACTCTTCTATTATCAAGCTCAATAGTAGAAAATGGGGCAGGAACTGGGTGAACAGTTT
ATCCTCCTCTCTCTTCAGGAACAGCTCATGCTGGAGCTTCTGTTGATTTAGCTATTTTTT
CTCTTCATTTAGCTGGAATTTCCTCAATTTTAGGGGCAGTAAATTTTATTACAACTGTAA
TTAATATACGATCGTCAGGAATTACTTTAGATCGACTACCTTTATTTGTTTGATCTGTAG
TTATTACAGCTATCTTATTACTTCTTTCTCTTCCTGTTTTAGCTGGAGCTATTACTATGT
TATTAACAGACCGAAACTTAAATACATCTTTCTTTGATCCAATCGGAGGAGGAGATCCTA
TT
>31-3
TCGGAACTTCTCTAAGAATTTTAATTCGTGCTGAACTTAGCCACCCTGGTATATTTATTG
GGAATGACCAAATTTATAATGTAATTGTAACAGCTCATGCATTTATTATAATTTTCTTTA
TAGTAATGCCAATTATAATTGGAGGATTTGGAAATTGATTAGTTCCTTTAATATTAGGAG
CCCCTGATATAGCTTTCCCTCGAATGAATAATATAAGTTTTTGAATACTACCTCCTTCAT
TGACTCTTCTATTATCAAGCTCAATAGTAGAAAATGGGGCAGGAACTGGGTGAACAGTTT
ATCCTCCTCTCTCTTCAGGAACAGCTCATGCTGGAGCTTCTGTTGATTTAGCTATTTTTT
CTCTTCATTTAGCTGGAATTTCCTCAATTTTAGGGGCAGTAAATTTTATTACAACTGTGA
TTAATATGCGATCGTCAGGGATTACTTTAGATCGACTACCCTTATTTGTTTGATCTGTAG
TTATTACAGCTATCTTATTACTTCTTTCTCTTCCTGTTTTAGCTGGAGCTATTACTATAT
TATTAACATACCGAAACTTAAATACATCTTTCTTTGATCCAATCGGAGGGGGAGACCCTA
TT
>34-1
TCGGAACTTCTCTAAGAATTTTAATTCGTGCTGAACTTAGCCACCCTGGTATATTTATTG
GGAATGACCAAATTTATAATGTAATTGTAACAGCTCATGCATTTATTATAATTTTCTTTA
TAGTAATGCCAATTATAATTGGAGGATTTGGAAATTGATTAGTTCCTTTAATATTAGGAG
CCCCTGATATAGCTTTCCCTCGAATGAATAATATAAGTTTTTGAATACTACCTCCTTCAT
TGACTCTTCTATTATCAAGCTCAATAGTAGAAAATGGAGCAGGAACTGGATGAACAGTTT
ATCCTCCTCTTTCTTCAGGAACAGCTCATGCTGGAGCTTCTGTTGATTTAGCTATTTTTT
CTCTTCATTTAGCTGGAATTTCCTCAATTTTAGGGGCAGTAAATTTTATTACAACTGTAA
TTAATATACGATCGTCAGGGATTACTTTAGATCGACTACCCTTATTTGTTTGATCTGTAG
TTATTACAGCTATCTTATTACTTCTTTCTCTTCCTGTTTTAGCTGGAGCTATTACTATAT
TATTAACAGACCGAAACTTAAATACATCTTTCTTTGATCCAATCGGAGGGGGAGACCCTA
TT
>34-2
TCGGAACTTCTCTAAGAATTTTAATTCGTGCTGAACTTAGCCACCCTGGTATATTTATTG
GGAATGACCAAATTTATAATGTAATTGTAACAGCTCATGCATTTATTATAATTTTCTTTA
TAGTAATGCCAATTATAATTGGAGGATTTGGAAATTGATTAGTTCCTTTAATATTAGGAG
CCCCTGATATAGCTTTCCCTCGAATGAATAATATAAGTTTTTGAATACTACCTCCTTCAT
TGACTCTTCTATTATCAAGCTCAATAGTAGAAAATGGGGCAGGAACTGGGTGAACAGTTT
ATCCTCCTCTCTCTTCAGGAACAGCTCATGCTGGAGCTTCTGTTGATTTAGCTATTTTTT
CTCTTCATTTAGCTGGAATTTCCTCAATTTTAGGGGCAGTAAATTTTATTACAACTGTGA
TTAATATGCGATCGTCAGGGATTACTTTAGATCGACTACCCTTATTTGTTTGATCTGTAG
TTATTACAGCTATCTTATTACTTCTTTCTCTTCCTGTTTTAGCTGGAGCTATTACTATAT

TATTAACAGACCGAAACTTAAATACATCTTTCTTTGATCCAATCGGAGGGGGAGACCCTA
TT
>34-3
TCGGAACTTCTCTAAGAATTTTAATTCGTGCTGAACTTAGCCACCCTGGTATATTTATTG
GGAATGACCAAATTTATAATGTAATTGTAACAGCTCATGCATTTATTATAATTTTCTTTA
TAGTAATGCCAATTATAATTGGAGGATTTGGAAATTGATTAGTTCCTTTAATATTAGGAG
CCCCTGATATAGCTTTCCCTCGAATGAATAATATAAGTTTTTGAATACTACCTCCTTCAT
TGACTCTTCTATTATCAAGCTCAATAGTAGAAAATGGGGCAGGAACTGGGTGAACAGTTT
ATCCTCCTCTCTCTTCAGGAACAGCTCATGCTGGAGCTTCTGTTGATTTAGCTATTTTTT
CTCTTCATTTAGCTGGAATTTCCTCAATTTTAGGGGCAGTAAATTTTATTACAACTGTGA
TTAATATGCGATCGTCAGGGATTACTTTAGATCGACTACCCTTATTTGTTTGATCTGTAG
TTATTACAGCTATCTTATTACTTCTTTCTCTTCCTGTTTTAGCTGGAGCTATTACTATAT
TATTAACAGACCGAAACTTAAATACATCTTTCTTTGATCCAATCGGAGGGGGAGACCCTA
TT
>34-4
TCGGAACTTCTCTAAGAATTTTAATTCGTGCTGAACTTAGCCACCCTGGTATATTTATTG
GGAATGACCAAATTTATAATGTAATTGTAACAGCTCATGCATTTATTATAATTTTCTTTA
TAGTAATGCCAATTATAATTGGAGGATTTGGAAATTGATTAGTTCCTTTAATATTAGGAG
CCCCTGATATAGCTTTCCCTCGAATGAATAATATAAGTTTTTGAATACTACCTCCTTCAT
TGACTCTTCTATTATCAAGCTCAATAGTAGAAAATGGGGCAGGAACTGGGTGAACAGTTT
ATCCTCCTCTCTCTTCAGGAACAGCTCATGCTGGAGCTTCTGTTGATTTAGCTATTTTTT
CTCTTCATTTAGCTGGAATTTCCTCAATTTTAGGGGCAGTAAATTTTATTACAACTGTGA
TTAATATGCGATCGTCAGGGATTACTTTAGATCGACTACCCTTATTTGTTTGATCTGTAG
TTATTACAGCTATCTTATTACTTCTTTCTCTTCCTGTTTTAGCTGGAGCTATTACTATAT
TATTAACAGACCGAAACTTAAATACATCTTTCTTTGATCCAATCGGAGGGGGAGACCCTA
TT
>34-5
TCGGAACTTCTCTAAGAATTTTAATTCGTGCTGAACTTAGCCACCCTGGTATATTTATTG
GGAATGACCAAATTTATAATGTAATTGTAACAGCTCATGCATTTATTATAATTTTCTTTA
TAGTAATGCCAATTATAATTGGAGGATTTGGAAATTGATTAGTTCCTTTAATATTAGGAG
CCCCTGATATAGCTTTCCCTCGAATGAATAATATAAGTTTTTGAATACTACCTCCTTCAT
TGACTCTTCTATTATCAAGCTCAATAGTAGAAAATGGGGCAGGAACTGGGTGAACAGTTT
ATCCTCCTCTCTCTTCAGGAACAGCTCATGCTGGAGCTTCTGTTGATTTAGCTATTTTTT
CTCTTCATTTAGCTGGAATTTCCTCAATTTTAGGGGCAGTAAATTTTATTACAACTGTGA
TTAATATGCGATCGTCAGGGATTACTTTAGATCGACTACCCTTATTTGTTTGATCTGTAG
TTATTACAGCTATCTTATTACTTCTTTCTCTTCCTGTTTTAGCTGGAGCTATTACTATAT
TATTAACAGACCGAAACTTAAATACATCTTTCTTTGATCCAATCGGAGGGGGAGACCCTA
TT
>34-6
TCGGAACTTCTCTAAGAATTTTAATTCGTGCTGAACTTAGCCACCCTGGTATATTTATTG
GGAATGACCAAATTTATAATGTAATTGTAACAGCTCATGCATTTATTATAATTTTCTTTA
TAGTAATGCCAATTATAATTGGAGGATTTGGAAATTGATTAGTTCCTTTAATATTAGGAG
CCCCTGATATAGCTTTCCCTCGAATGAATAATATAAGTTTTTGAATACTACCTCCTTCAT
TGACTCTTCTATTATCAAGCTCAATAGTAGAAAATGGAGCAGGAACTGGATGAACAGTTT
ATCCTCCTCTTTCTTCAGGAACAGCTCATGCTGGAGCTTCTGTTGATTTAGCTATTTTTT
CTCTTCATTTAGCTGGAATTTCCTCAATTTTAGGGGCAGTAAATTTTATTACAACTGTAA
TTAATATACGATCGTCAGGGATTACTTTAGATCGACTACCCTTATTTGTTTGATCTGTAG
TTATTACAGCTATCTTATTACTTCTTTCTCTTCCTGTTTTAGCTGGAGCTATTACTATAT
TATTAACAGACCGAAACTTAAATACATCTTTCTTTGATCCAATCGGAGGGGGAGACCCTA
TT
>34-7
TCGGAACTTCTCTAAGAATTTTAATTCGTGCTGAACTTAGCCACCCTGGTATATTTATTG
GGAATGACCAAATTTATAATGTAATTGTAACAGCTCATGCATTTATTATAATTTTCTTTA
TAGTAATGCCAATTATAATTGGAGGATTTGGAAATTGATTAGTTCCTTTAATATTAGGAG
CCCCTGATATAGCTTTCCCTCGAATGAATAATATAAGTTTTTGAATACTACCTCCTTCAT
TGACTCTTCTATTATCAAGCTCAATAGTAGAAAATGGGGCAGGAACTGGGTGAACAGTTT
ATCCTCCTCTCTCTTCAGGAACAGCTCATGCTGGAGCTTCTGTTGATTTAGCTATTTTTT

CTCTTCATTTAGCTGGAATTTCCTCAATTTTAGGGGCAGTAAATTTTATTACAACTGTGA
TTAATATGCGATCGTCAGGGATTACTTTAGATCGACTACCCTTATTTGTTTGATCTGTAG
TTATTACAGCTATCTTATTACTTCTTTCTCTTCCTGTTTTAGCTGGAGCTATTACTATAT
TATTAACAGACCGAAACTTAAATACATCTTTCTTTGATCCAATCGGAGGGGGAGACCCTA
TT
>34-8
TCGGAACTTCTCTAAGAATTTTAATTCGTGCTGAACTTAGCCACCCTGGTATATTTATTG
GGAATGACCAAATTTATAATGTAATTGTAACAGCTCATGCATTTATTATAATTTTCTTTA
TAGTAATGCCAATTATAATTGGAGGATTTGGAAATTGATTAGTTCCTTTAATATTAGGAG
CCCCTGATATAGCTTTCCCTCGAATGAATAATATAAGTTTTTGAATACTACCTCCTTCAT
TGACTCTTCTATTATCAAGCTCAATAGTAGAAAATGGAGCAGGAACTGGATGAACAGTTT
ATCCTCCTCTTTCTTCAGGAACAGCTCATGCTGGAGCTTCTGTTGATTTAGCTATTTTTT
CTCTTCATTTAGCTGGAATTTCCTCAATTTTAGGGGCAGTAAATTTTATTACAACTGTAA
TTAATATACGATCGTCAGGGATTACTTTAGATCGACTACCCTTATTTGTTTGATCTGTAG
TTATTACAGCTATCTTATTACTTCTTTCTCTTCCTGTTTTAGCTGGAGCTATTACTATAT
TATTAACAGACCGAAACTTAAATACATCTTTCTTTGATCCAATCGGAGGGGGAGACCCTA
TT
>34-9
TCGGAACTTCTCTAAGAATTTTAATTCGTGCTGAACTTAGCCACCCTGGTATATTTATTG
GGAATGACCAAATTTATAATGTAATTGTAACAGCTCATGCACTTATCATAATTTTCTTTA
TAGTAATGCCAATTATAATTGGAGGATTTGGAAATTGATTAGTTCCTTTAATATTAGGAG
CCCCTGATATAGCTTTCCCTCGAATGAATAATATAAGTTTTTGAATACTACCTCCTTCAT
TGACTCTTCTATTATCAAGCTCAATAGTAGAAAATGGAGCAGGAACTGGATGAACAGTTT
ATCCTCCTCTTTCTTCAGGAACAGCTCATGCTGGAGCTTCTGTTGATTTAGCTATTTTTT
CTCTTCATTTAGCTGGAATTTCCTCAATTTTAGGGGCAGTAAATTTTATTACAACTGTAA
TTAATATACGATCGTCAGGGATTACTTTAGATCGACTACCCTTATTTGTTTGATCTGTAG
TTATTACAGCTATCTTATTACTTCTTTCTCTTCCTGTTTTAGCTGGAGCTATTACTATAT
TATTAACAGACCGAAACTTAAATACATCTTTCTTTGATCCAATCGGAGGGGGAGACCCTA
TT
>34-10
TCGGAACTTCTCTAAGAATTTTAATTCGTGCTGAACTTAGCCACCCTGGTATATTTATTG
GGAATGACCAAATTTATAATGTAATTGTAACAGCTCATGCATTTATTATAATTTTCTTTA
TAGTAATGCCAATTATAATTGGAGGATTTGGAAATTGATTAGTTCCTTTAATATTAGGAG
CCCCTGATATAGCTTTCCCTCGAATGAATAATATAAGTTTTTGAATACTACCTCCTTCAT
TGACTCTTCTATTATCAAGCTCAATAGTAGAAAATGGGGCAGGAACTGGGTGAACAGTTT
ATCCTCCTCTCTCTTCAGGAACAGCTCATGCTGGAGCTTCTGTTGATTTAGCTATTTTTT
CTCTTCATTTAGCTGGAATTTCCTCAATTTTAGGGGCAGTAAATTTTATTACAACTGTGA
TTAATATGCGATCGTCAGGGATTACTTTAGATCGACTACCCTTATTTGTTTGATCTGTAG
TTATTACAGCTATCTTATTACTTCTTTCTCTTCCTGTTTTAGCTGGAGCTATTACTATAT
TATTAACAGACCGAAACTTAAATACATCTTTCTTTGATCCAATCGGAGGGGGAGACCCTA
TT
>34-11
TCGGAACTTCTCTAAGAATTTTAATTCGTGCTGAACTTAGCCACCCTGGTATATTTATTG
GGAATGACCAAATTTATAATGTAATTGTAACAGCTCATGCATTTATTATAATTTTCTTTA
TAGTAATGCCAATTATAATTGGAGGATTTGGAAATTGATTAGTTCCTTTAATATTAGGAG
CCCCTGATATAGCTTTCCCTCGAATGAATAATATAAGTTTTTGAATACTACCTCCTTCAT
TGACTCTTCTATTATCAAGCTCAATAGTAGAAAATGGGGCAGGAACTGGGTGAACAGTTT
ATCCTCCTCTCTCTTCAGGAACAGCTCATGCTGGAGCTTCTGTTGATTTAGCTATTTTTT
CTCTTCATTTAGCTGGAATTTCCTCAATTTTAGGGGCAGTAAATTTTATTACAACTGTGA
TTAATATGCGATCGTCAGGGATTACTTTAGATCGACTACCCTTATTTGTTTGATCTGTAG
TTATTACAGCTATCTTATTACTTCTTTCTCTTCCTGTTTTAGCTGGAGCTATTACTATAT
TATTAACAGACCGAAACTTAAATACATCTTTCTTTGATCCAATCGGAGGGGGAGACCCTA
TT
>34-12
TCGGAACTTCTCTAAGAATTTTAATTCGTGCTGAACTTAGCCACCCTGGTATATTTATTG
GGAATGACCAAATTTATAATGTAATTGTAACAGCTCATGCATTTATTATAATTTTCTTTA
TAGTAATGCCAATTATAATTGGAGGATTTGGAAATTGATTAGTTCCTTTAATATTAGGAG

CCCCTGATATAGCTTTCCCTCGAATGAATAATATAAGTTTTTGAATACTACCTCCTTCAT
TGACTCTTCTATTATCAAGCTCAATAGTAGAAAATGGGGCAGGAACTGGGTGAACAGTTT
ATCCTCCTCTCTCTTCAGGAACAGCTCATGCTGGAGCTTCTGTTGATTTAGCTATTTTTT
CTCTTCATTTAGCTGGAATTTCCTCAATTTTAGGGGCAGTAAATTTTATTACAACTGTGA
TTAATATGCGATCGTCAGGGATTACTTTAGATCGACTACCCTTATTTGTTTGATCTGTAG
TTATTACAGCTATCTTATTACTTCTTTCTCTTCCTGTTTTAGCTGGAGCTATTACTATAT
TATTAACAGACCGAAACTTAAATACATCTTTCTTTGATCCAATCGGAGGGGGAGACCCTA
TT
>34-13
TCGGAACTTCTCTAAGAATTTTAATTCGTGCTGAACTTAGCCACCCTGGTATATTTATTG
GGAATGACCAAATTTATAATGTAATTGTAACAGCTCATGCATTTATTATAATTTTCTTTA
TAGTAATGCCAATTATAATTGGAGGATTTGGAAATTGATTAGTTCCTTTAATATTAGGAG
CCCCTGATATAGCTTTCCCTCGAATGAATAATATAAGTTTTTGAATACTACCTCCTTCAT
TGACTCTTCTATTATCAAGCTCAATAGTAGAAAATGGAGCAGGAACTGGATGAACAGTTT
ATCCTCCTCTTTCTTCAGGAACAGCTCATGCTGGAGCTTCTGTTGATTTAGCTATTTTTT
CTCTTCATTTAGCTGGAATTTCCTCAATTTTAGGGGCAGTAAATTTTATTACAACTGTAA
TTAATATACGATCGTCAGGGATTACTTTAGATCGACTACCCTTATTTGTTTGATCTGTAG
TTATTACAGCTATCTTATTACTTCTTTCTCTTCCTGTTTTAGCTGGAGCTATTACTATAT
TATTAACAGACCGAAACTTAAATACATCTTTCTTTGATCCAATCGGAGGGGGAGACCCTA
TT
>34-14
TCGGAACTTCTCTAAGAATTTTAATTCGTGCTGAACTTAGCCACCCTGGTATATTTATTG
GGAATGACCAAATTTATAATGTAATTGTAACAGCTCATGCATTTATTATAATTTTCTTTA
TAGTAATGCCAATTATAATTGGAGGATTTGGAAATTGATTAGTTCCTTTAATATTAGGAG
CCCCTGATATAGCTTTCCCTCGAATGAATAATATAAGTTTTTGAATACTACCTCCTTCAT
TGACTCTTCTATTATCAAGCTCAATAGTAGAAAATGGAGCAGGAACTGGATGAACAGTTT
ATCCTCCTCTTTCTTCAGGAACAGCTCATGCTGGAGCTTCTGTTGATTTAGCTATTTTTT
CTCTTCATTTAGCTGGAATTTCCTCAATTTTAGGGGCAGTAAATTTTATTACAACTGTAA
TTAATATACGATCGTCAGGGATTACTTTAGATCGACTACCCTTATTTGTTTGATCTGTAG
TTATTACAGCTATCTTATTACTTCTTTCTCTTCCTGTTTTAGCTGGAGCTATTACTATAT
TATTAACAGACCGAAACTTAAATACATCTTTCTTTGATCCAATCGGAGGGGGAGACCCTA
TT
>34-15
TCGGAACTTCTCTAAGAATTTTAATTCGTGCTGAACTTAGCCACCCTGGTATATTTATTG
GGAATGACCAAATTTATAATGTAATTGTAACAGCTCATGCATTTATTATAATTTTCTTTA
TAGTAATGCCAATTATAATTGGAGGATTTGGAAATTGATTAGTTCCTTTAATATTAGGAG
CCCCTGATATAGCTTTCCCTCGAATGAATAATATAAGTTTTTGAATACTACCTCCTTCAT
TGACTCTTCTATTATCAAGCTCAATAGTAGAAAATGGGGCAGGAACTGGGTGAACAGTTT
ATCCTCCTCTCTCTTCAGGAACAGCTCATGCTGGAGCTTCTGTTGATTTAGCTATTTTTT
CTCTTCATTTAGCTGGAATTTCCTCAATTTTAGGGGCAGTAAATTTTATTACAACTGTGA
TTAATATGCGATCGTCAGGGATTACTTTAGATCGACTACCCTTATTTGTTTGATCTGTAG
TTATTACAGCTATCTTATTACTTCTTTCTCTTCCTGTTTTAGCTGGAGCTATTACTATAT
TATTAACAGACCGAAACTTAAATACATCTTTCTTTGATCCAATCGGAGGGGGAGACCCTA
TT
>36-1
TCGGAACTTCTCTAAGAATTTTAATTCGTGCTGAACTTAGCCACCCTGGTATATTTATTG
GGAATGACCAAATTTATAATGTAATTGTAACAGCTCATGCATTTATTATAATTTTCTTTA
TAGTAATGCCAATTATAATTGGAGGATTTGGAAATTGATTAGTTCCTTTAATATTAGGAG
CCCCTGATATAGCTTTCCCTCGAATGAATAATATAAGTTTTTGAATACTACCTCCTTCAT
TGACTCTTCTATTATCAAGCTCAATAGTAGAAAATGGGGCAGGAACTGGGTGAACAGTTT
ATCCTCCTCTCTCTTCAGGAACAGCTCATGCTGGAGCTTCTGTTGATTTAGCTATTTTTT
CTCTTCATTTAGCTGGAATTTCCTCAATTTTAGGGGCAGTAAATTTTATTACAACTGTGA
TTAATATACGATCGTCAGGGATTACTTTAGATCGACTACCCTTATTTGTTTGATCTGTAG
TTATTACAGCTATCTTATTACTTCTTTCTCTTCCTGTTTTAGCTGGAGCTATTACTATAT
TATTAACAGACCGAAACTTAAATACATCTTTCTTTGATCCAATCGGAGGGGGAGACCCTA
TT
>36-2

TCGGAACTTCTCTAAGAATTTTAATTCGTGCTGAACTTAGCCACCCTGGTATATTTATTG
GGAATGACCAAATTTATAATGTAATTGTAACAGCTCATGCATTTATTATAATTTTCTTTA
TAGTAATGCCAATTATAATTGGAGGATTTGGAAATTGATTAGTTCCTTTAATATTAGGAG
CCCCTGATATAGCTTTCCCTCGAATGAATAATATAAGTTTTTGAATACTACCTCCTTCAT
TGACTCTTCTATTATCAAGCTCAATAGTAGAAAATGGGGCAGGAACTGGGTGAACAGTTT
ATCCTCCTCTCTCTTCAGGAACAGCTCATGCTGGAGCTTCTGTTGATTTAGCTATTTTTT
CTCTTCATTTAGCTGGAATTTCCTCAATTTTAGGGGCAGTAAATTTTATTACAACTGTGA
TTAATATGCGATCGTCAGGGATTACTTTAGATCGACTACCCTTATTTGTTTGATCTGTAG
TTATTACAGCTATCTTATTACTTCTTTCTCTTCCTGTTTTAGCTGGAGCTATTACTATAT
TATTAACAGACCGAAACTTAAATACATCTTTCTTTGATCCAATCGGAGGGGGAGACCCTA
TT
>36-3
TCGGAACTTCTCTAAGAATTTTAATTCGTGCTGAACTTAGCCACCCTGGTATATTTATTG
GGAATGACCAAATTTATAATGTAATTGTAACAGCTCATGCATTTATTATAATTTTCTTTA
TAGTAATGCCAATTATAATTGGAGGATTTGGAAATTGATTAGTTCCTTTAATATTAGGAG
CCCCTGATATAGCTTTCCCTCGAATGAATAATATAAGTTTTTGAATACTACCTCCTTCAT
TGACTCTTCTATTATCAAGCTCAATAGTAGAAAATGGGGCAGGAACTGGGTGAACAGTTT
ATCCTCCTCTCTCTTCAGGAACAGCTCATGCTGGAGCTTCTGTTGATTTAGCTATTTTTT
CTCTTCATTTAGCTGGAATTTCCTCAATTTTAGGGGCAGTAAATTTTATTACAACTGTGA
TTAATATGCGATCGTCAGGGATTACTTTAGATCGACTACCCTTATTTGTTTGATCTGTAG
TTATTACAGCTATCTTATTACTTCTTTCTCTTCCTGTTTTAGCTGGAGCTATTACTATAT
TATTAACAGACCGAAACTTAAATACATCTTTCTTTGATCCAATCGGAGGGGGAGACCCTA
TT
>66-1
TCGGAACTTCTCTAAGAATTTTAATTCGTGCTGAACTTAGCCACCCTGGTATATTTATTG
GGAATGACCAAATTTATAATGTAATTGTAACAGCTCATGCATTTATTATAATTTTCTTTA
TAGTAATGCCAATTATAATTGGAGGATTTGGAAATTGATTAGTTCCTTTAATATTAGGAG
CCCCTGATATAGCTTTCCCTCGAATGAATAATATAAGTTTTTGAATACTACCTCCTTCAT
TGACTCTTCTATTATCAAGCTCAATAGTAGAAAATGGGGCAGGAACTGGGTGAACAGTTT
ATCCTCCTCTCTCTTCAGGAACAGCTCATGCTGGAGCTTCTGTTGATTTAGCTATTTTTT
CTCTTCATTTAGCTGGAATTTCCTCAATTTTAGGGGCAGTAAATTTTATTACAACTGTGA
TTAATATGCGATCGTCAGGGATTACTTTAGATCGACTACCCTTATTTGTTTGATCTGTAG
TTATTACAGCTATCTTATTACTTCTTTCTCTTCCTGTTTTAGCTGGAGCTATTACTATAT
TATTAACAGACCGAAACTTAAATACATCTTTCTTTGATCCAATCGGAGGGGGAGACCCTA
TT
>66-2
TCGGAACTTCTCTAAGAATTTTAATTCGTGCTGAACTTAGCCACCCTGGTATATTTATTG
GGAATGACCAAATTTATAATGTAATTGTAACAGCTCATGCATTTATTATAATTTTCTTTA
TAGTAATGCCAATTATAATTGGAGGATTTGGAAATTGATTAGTTCCTTTAATATTAGGAG
CCCCTGATATAGCTTTCCCTCGAATGAATAATATAAGTTTTTGAATACTACCTCCTTCAT
TGACTCTTCTATTATCAAGCTCAATAGTAGAAAATGGAGCAGGAACTGGATGAACAGTTT
ATCCTCCTCTTTCTTCAGGAACAGCTCATGCTGGAGCTTCTGTTGATTTAGCTATTTTTT
CTCTTCATTTAGCTGGAATTTCCTCAATTTTAGGGGCAGTAAATTTTATTACAACTGTAA
TTAATATACGATCGTCAGGGATTACTTTAGATCGACTACCCTTATTTGTTTGATCTGTAG
TTATTACAGCTATCTTATTACTTCTTTCTCTTCCTGTTTTAGCTGGAGCTATTACTATAT
TATTAACAGACCGAAACTTAAATACATCTTTCTTTGATCCAATCGGAGGGGGAGACCCTA
TT
>66-3
TCGGAACTTCTCTAAGAATTTTAATTCGTGCTGAACTTAGCCACCCTGGTATATTTATTG
GGAATGACCAAATTTATAATGTAATTGTAACAGCTCATGCATTTATTATAATTTTCTTTA
TAGTAATGCCAATTATAATTGGAGGATTTGGAAATTGATTAGTTCCTTTAATATTAGGAG
CCCCTGATATAGCTTTCCCTCGAATGAATAATATAAGTTTTTGAATACTACCTCCTTCAT
TGACTCTTCTATTATCAAGCTCAATAGTAGAAAATGGGGCAGGAACTGGGTGAACAGTTT
ATCCTCCTCTCTCTTCAGGAACAGCTCATGCTGGAGCTTCTGTTGATTTAGCTATTTTTT
CTCTTCATTTAGCTGGAATTTCCTCAATTTTAGGGGCAGTAAATTTTATTACAACTGTGA
TTAATATGCGATCGTCAGGGATTACTTTAGATCGACTACCCTTATTTGTTTGATCTGTAG
TTATTACAGCTATCTTATTACTTCTTTCTCTTCCTGTTTTAGCTGGAGCTATTACTATAT

TATTAACAGACCGAAACTTAAATACATCTTTCTTTGATCCAATCGGAGGGGGAGACCCTA
TT
>68-1
TCGGAACTTCTCTAAGAATTTTAATTCGTGCTGAACTTAGCCACCCTGGTATATTTATTG
GGAATGACCAAATTTATAATGTAATTGTAACAGCTCATGCATTTATTATAATTTTCTTTA
TAGTAATACCAATTATAATTGGAGGATTTGGAAATTGATTAGTTCCTTTAATATTAGGAG
CCCCTGATATAGCCTTTCCTCGAATAAATAATATAAGTTTTTGAATACTACCTCCTTCAT
TGACTCTTCTATTATCAAGCTCAATAGTAGAAAATGGGGCAGGAACTGGGTGAACAGTTT
ATCCTCCTCTCTCTTCAGGAACAGCTCATGCTGGAGCTTCTGTTGATTTAGCTATTTTTT
CTCTTCATTTAGCTGGAATTTCCTCAATTTTAGGGGCAGTAAATTTTATTACAACTGTAA
TTAATATACGATCGTCAGGAATTACTTTAGATCGACTACCTTTATTTGTTTGATCTGTAG
TTATTACAGCTATCTTATTACTTCTTTCTCTTCCTGTTTTAGCTGGAGCTATTACTATGT
TATTAACAGACCGAAACTTAAATACATCTTTCTTTGATCCAATCGGAGGAGGAGATCCTA
TT
>68-2
TCGGAACTTCTCTAAGAATTTTAATTCGTGCTGAACTTAGCCACCCTGGTATATTTATTG
GGAATGACCAAATTTATAATGTAATTGTAACAGCTCATGCATTTATTATAATTTTCTTTA
TAGTAATACCAATTATAATTGGAGGATTTGGAAATTGATTAGTTCCTTTAATATTAGGAG
CCCCTGATATAGCCTTTCCTCGAATAAATAATATAAGTTTTTGAATACTACCTCCTTCAT
TGACTCTTCTATTATCAAGCTCAATAGTAGAAAATGGGGCAGGAACTGGGTGAACAGTTT
ATCCTCCTCTCTCTTCAGGAACAGCTCATGCTGGAGCTTCTGTTGATTTAGCTATTTTTT
CTCTTCATTTAGCTGGAATTTCCTCAATTTTAGGGGCAGTAAATTTTATTACAACTGTAA
TTAATATACGATCGTCAGGAATTACTTTAGATCGACTACCTTTATTTGTTTGATCTGTAG
TTATTACAGCTATCTTATTACTTCTTTCTCTTCCTGTTTTAGCTGGAGCTATTACTATGT
TATTAACAGACCGAAACTTAAATACATCTTTCTTTGATCCAATCGGAGGAGGAGATCCTA
TT
>68-3
TCGGAACTTCTCTAAGAATTTTAATTCGTGCTGAACTTAGCCACCCTGGTATATTTATTG
GGAATGACCAAATTTATAATGTAATTGTAACAGCTCATGCATTTATTATAATTTTCTTTA
TAGTAATGCCAATTATAATTGGAGGATTTGGAAATTGATTAGTTCCTTTAATATTAGGAG
CCCCTGATATAGCTTTCCCTCGAATGAATAATATAAGTTTTTGAATACTACCTCCTTCAT
TGACTCTTCTATTATCAAGCTCAATAGTAGAAAATGGGGCAGGAACTGGGTGAACAGTTT
ATCCTCCTCTCTCTTCAGGAACAGCTCATGCTGGAGCTTCTGTTGATTTAGCTATTTTTT
CTCTTCATTTAGCTGGAATTTCCTCAATTTTAGGGGCAGTAAATTTTATTACAACTGTGA
TTAATATGCGATCGTCAGGGATTACTTTAGATCGACTACCCTTATTTGTTTGATCTGTAG
TTATTACAGCTATCTTATTACTTCTTTCTCTTCCTGTTTTAGCTGGAGCTATTACTATAT
TATTAACAGACCGAAACTTAAATACATCTTTCTTTGATCCAATCGGAGGGGGAGACCCTA
TT
>74-1
TCGGAACTTCTCTAAGAATTTTAATTCGTGCTGAACTTAGCCACCCTGGTATATTTATTG
GGAATGACCAAATTTATAATGTAATTGTAACAGCTCATGCATTTATTATAATTTTCTTTA
TAGTAATGCCAATTATAATTGGAGGATTTGGAAATTGATTAGTTCCTTTAATATTAGGAG
CCCCTGATATAGCTTTCCCTCGAATGAATAATATAAGTTTTTGAATACTACCTCCTTCAT
TGACTCTTCTATTATCAAGCTCAATAGTAGAAAATGGGGCAGGAACTGGGTGAACAGTTT
ATCCTCCTCTCTCTTCAGGAACAGCTCATGCTGGAGCTTCTGTTGATTTAGCTATTTTTT
CTCTTCATTTAGCTGGAATTTCCTCAATTTTAGGGGCAGTAAATTTTATTACAACTGTGA
TTAATATGCGATCGTCAGGGATTACTTTAGATCGACTACCCTTATTTGTTTGATCTGTAG
TTATTACAGCTATCTTATTACTTCTTTCTCTTCCTGTTTTAGCTGGAGCTATTACTATAT
TATTAACAGACCGAAACTTAAATACATCTTTCTTTGATCCAATCGGAGGGGGAGACCCTA
TT
>74-3
TCGGAACTTCTCTAAGAATTTTAATTCGTGCTGAACTTAGCCACCCTGGTATATTTATTG
GGAATGACCAAATTTATAATGTAATTGTAACAGCTCATGCATTTATTATAATTTTCTTTA
TAGTAATGCCAATTATAATTGGAGGATTTGGAAATTGATTAGTTCCTTTAATATTAGGAG
CCCCTGATATAGCTTTCCCTCGAATGAATAATATAAGTTTTTGAATACTACCTCCTTCAT
TGACTCTTCTATTATCAAGCTCAATAGTAGAAAATGGGGCAGGAACTGGGTGAACAGTTT
ATCCTCCTCTCTCTTCAGGAACAGCTCATGCTGGAGCTTCTGTTGATTTAGCTATTTTTT

CTCTTCATTTAGCTGGAATTTCCTCAATTTTAGGGGCAGTAAATTTTATTACAACTGTGA
TTAATATGCGATCGTCAGGGATTACTTTAGATCGACTACCCTTATTTGTTTGATCTGTAG
TTATTACAGCTATCTTATTACTTCTTTCTCTTCCTGTTTTAGCTGGAGCTATTACTATAT
TATTAACAGACCGAAACTTAAATACATCTTTCTTTGATCCAATCGGAGGGGGAGACCCTA
TT
>76-1
TCGGAACTTCTCTAAGAATTTTAATTCGTGCTGAACTTAGCCACCCTGGTATATTTATTG
GGAATGACCAAATTTATAATGTAATTGTAACAGCTCATGCATTTATTATAATTTTCTTTA
TAGTAATGCCAATTATAATTGGAGGATTTGGAAATTGATTAGTTCCTTTAATATTAGGAG
CCCCTGATATAGCTTTCCCTCGAATGAATAATATAAGTTTTTGAATACTACCTCCTTCAT
TGACTCTTCTATTATCAAGCTCAATAGTAGAAAATGGGGCAGGAACTGGGTGAACAGTTT
ATCCTCCTCTCTCTTCAGGAACAGCTCATGCTGGAGCTTCTGTTGATTTAGCTATTTTTT
CTCTTCATTTAGCTGGAATTTCCTCAATTTTAGGGGCAGTAAATTTTATTACAACTGTGA
TTAATATACGATCGTCAGGGATTACTTTAGATCGACTACCCTTATTTGTTTGATCTGTAG
TTATTACAGCTATCTTATTACTTCTTTCTCTTCCTGTTTTAGCTGGAGCTATTACTATAT
TATTAACAGACCGAAACTTAAATACATCTTTCTTTGATCCAATCGGAGGAGGAGATCCTA
TT
>79-2
TCGGAACTTCTCTAAGAATTTTAATTCGTGCTGAACTTAGCCACCCTGGTATATTTATTG
GGAATGACCAAATTTATAATGTAATTGTAACAGCTCATGCATTTATTATAATTTTCTTTA
TAGTAATGCCAATTATAATTGGAGGATTTGGAAATTGATTAGTTCCTTTAATATTAGGAG
CCCCTGATATAGCTTTCCCTCGAATGAATAATATAAGTTTTTGAATACTACCTCCTTCAT
TGACTNTTCTATTATCAAGCTCAATAGTAGAAAATGGGGCAGGAACTGGGTGAACAGTTT
ATCCTCCTCTCTCTTCAGGAACAGCTCATGCTGGAGCTTCTGTTGATTTAGCTATTTTTT
CTNTTCATTTAGCTGGAATTTCCTCAATTTTAGGGGCAGTAAATTTTATTACAACTGTGA
TTAATATGCGATCGTCAGGGATTACTTTAGATCGACTACCCTTATTTGTTTGATCTGTAG
TTATTACAGCTATCTTATTACTTCTTTCTCTTCCTGTTTTAGCTGGAGCTATTACTATAT
TATTAACAGACCGAAACTTAAATACATCTTTCTTTGATCCAATCGGAGGGGGAGACCCTA
TT
>79-3
TCGGAACTTCTCTAAGAATTTTAATTCGTGCTGAACTTAGCCACCCTGGTATATTTATTG
GGAATGACCAAATTTATAATGTAATTGTAACAGCTCATGCATTTATTATAATTTTCTTTA
TAGTAATACCAATTATAATTGGAGGATTTGGAAATTGATTAGTTCCTTTAATATTAGGAG
CCCCTGATATAGCCTTTCCTCGAATAAATAATATAAGTTTTTGAATACTACCTCCTTCAT
TGACTCTTCTATTATCAAGCTCAATAGTAGAAAATGGGGCAGGAACTGGGTGAACAGTTT
ATCCTCCTCTCTCTTCAGGAACAGCTCATGCTGGAGCTTCTGTTGATTTAGCTATTTTTT
CTCTTCATTTAGCTGGAATTTCCTCAATTTTAGGGGCAGTAAATTTTATTACAACTGTAA
TTAATATACGATCGTCAGGAATTACTTTAGATCGACTACCTTTATTTGTTTGATCTGTAG
TTATTACAGCTATCTTATTACTTCTTTCTCTTCCTGTTTTAGCTGGAGCTATTACTATGT
TATTAACAGACCGAAACTTAAATACATCTTTCTTTGATCCAATCGGAGGAGGAGATCCTA
TT
>82-1
TCGGAACTTCTCTAAGAATTTTAATTCGTGCTGAACTTAGCCACCCTGGTATATTTATTG
GGAATGACCAAATTTATAATGTAATTGTAACAGCTCATGCATTTATTATAATTTTCTTTA
TAGTAATGCCAATTATAATTGGAGGATTTGGAAATTGATTAGTTCCTTTAATATTAGGAG
CCCCTGATATAGCTTTCCCTCGAATGAATAATATAAGTTTTTGAATACTACCTCCTTCAT
TGACTCTTCTATTATCAAGCTCAATAGTAGAAAATGGGGCAGGAACTGGGTGAACAGTTT
ATCCTCCTCTCTCTTCAGGAACAGCTCATGCTGGAGCTTCTGTTGATTTAGCTATTTTTT
CTCTTCATTTAGCTGGAATTTCCTCAATTTTAGGGGCAGTAAATTTTATTACAACTGTGA
TTAATATGCGATCGTCAGGGATTACTTTAGATCGACTACCCTTATTTGTTTGATCTGTAG
TTATTACAGCTATCTTATTACTTCTTTCTCTTCCTGTTTTAGCTGGAGCTATTACTATAT
TATTAACAGACCGAAACTTAAATACATCTTTCTTTGATCCAATCGGAGGGGGAGACCCTA
TT
>82-2
TCGGAACTTCTCTAAGAATTTTAATTCGTGCTGAACTTAGCCACCCTGGTATATTTATTG
GGAATGACCAAATTTATAATGTAATTGTAACAGCTCATGCATTTATTATAATTTTCTTTA
TAGTAATGCCAATTATAATTGGAGGATTTGGAAATTGATTAGTTCCTTTAATATTAGGAG

CCCCTGATATAGCTTTCCCTCGAATGAATAATATAAGTTTTTGAATACTACCTCCTTCAT
TGACTNTTCTATTATCAAGCTCAATAGTAGAAAATGGGGCAGGAACTGGGTGAACAGTTT
ATCCTCCTCTCTCTTCAGGAACAGCTCATGCTGGAGCTTCTGTTGATTTAGCTATTTTTT
CTCTTCATTTAGCTGGAATTTCCTCAATTTTAGGGGCAGTAAATTTTATTACAACTGTGA
TTAATATGCGATCGTCAGGGATTACTTTAGATCGACTACCCTTATTTGTTTGATCTGTAG
TTATTACAGCTATCTTATTACTTCTTTCTCTTCCTGTTTTAGCTGGAGCTATTACTATAT
TATTAACAGACCGAAACTTAAATACATCTTTCTTTGATCCAATCGGAGGGGGAGACCCTA
TT
